# Total Synthesis of Ophiorrhine A, G and Ophiorrhiside E Featuring a Bioinspired Intramolecular Diels–Alder Cycloaddition**

**DOI:** 10.1002/anie.202209135

**Published:** 2022-08-10

**Authors:** Wei Cao, Yingchao Dou, Cyrille Kouklovsky, Guillaume Vincent

**Affiliations:** ^1^ Institut de Chimie Moléculaire et des Matériaux d'Orsay Université Paris-Saclay CNRS 91405 Orsay France

**Keywords:** Biomimetic Synthesis, Diels–Alder Reaction, Indolopyridones, Monoterpene Indole Alkaloids, Total Synthesis

## Abstract

We report the first total synthesis of the monoterpene indole alkaloids ophiorrhine A via a late stage bioinspired intramolecular Diels–Alder cycloaddition to form the intricate bridged and spirannic polycyclic system. Several strategies were investigated to construct the indolopyridone moiety of ophiorrhiside E, the postulated biosynthetic precursor of ophiorrhine A. Eventually, the Friedel–Crafts‐type coupling of N‐methyl indolyl‐acetamide with a secologanin‐derived acid chloride delivered ophiorrhine G. Cyclodehydration of a protected form of the latter was followed by the desired spontaneous intramolecular Diels–Alder cycloaddition of protected ophiorrhiside E leading to ophiorrhine A.

## Graphical abstract

A late stage bioinspired intramolecular Diels–Alder cycloaddition of the indolopyridone of ophiorrhiside E was deployed to access the spirocyclic azabicyclic[2.2.2]octanone ring system of ophiorrhiside A and achieve its total synthesis. The indolopyridone of ophiorrhiside E was synthesized by the cyclodehydration of ophiorrhine G which was formed by the acylation of N‐methyl indolylacetamide by a secologanin derivative.

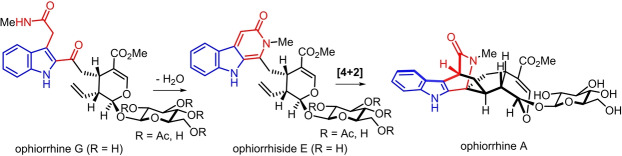

## Introduction

**Scheme 1:**
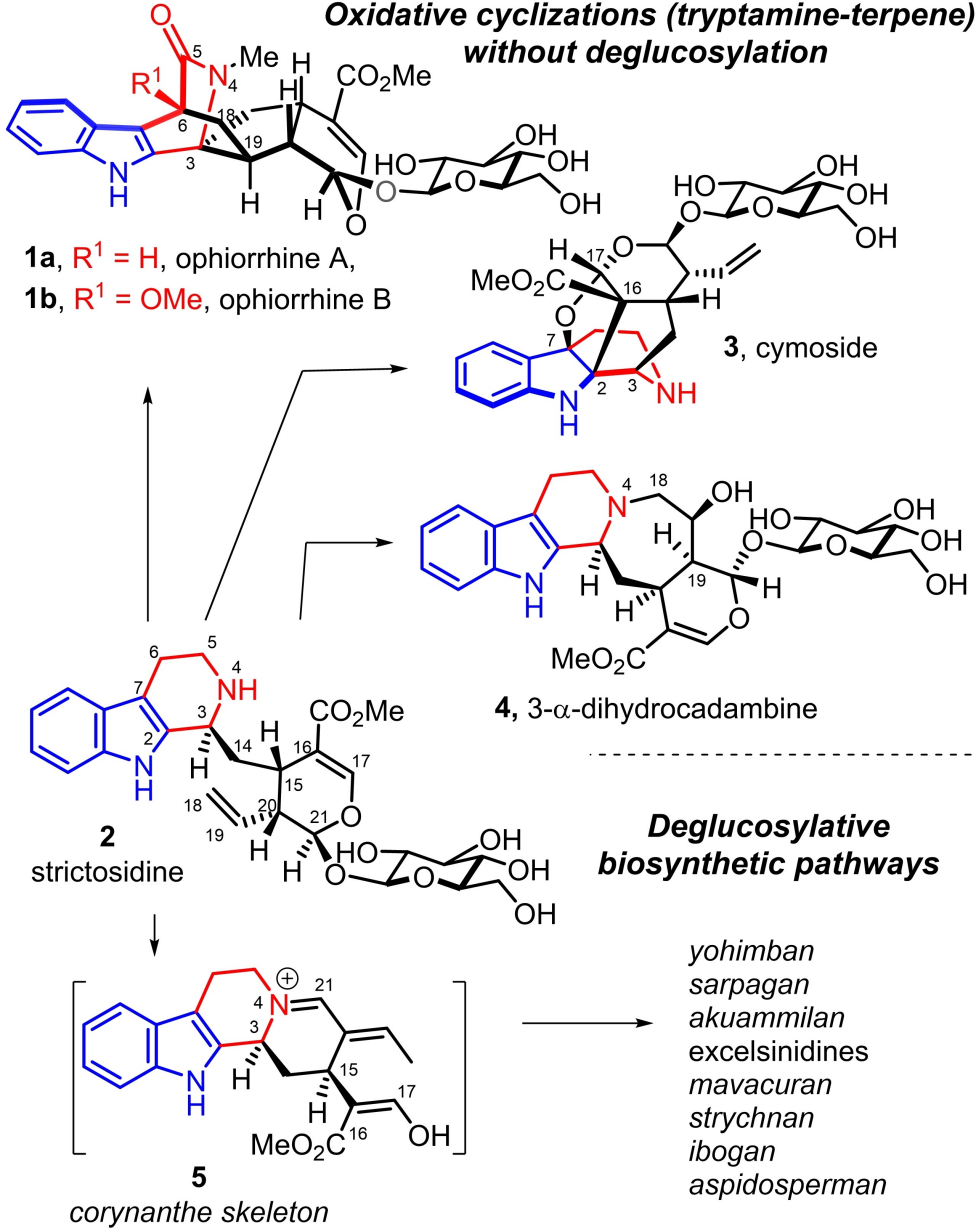
Biosynthetic oxidative cyclization pathways from strictosidine into glycosidic monoterpene indole alkaloids.

Ophiorrhine A and B (**1 a**, **b**) are monoterpene indole alkaloids isolated from *Ophiorrhiza japonica* and display an intricate polycyclic‐fused structure which was secured by single crystal X‐ray diffraction with a highly unusual bridged‐spirocyclic ring system (Scheme 1).^[1]^ These natural products exhibit in vitro immunosuppressive activity.

The monoterpene indole alkaloids which comprise over 3000 natural products are biosynthetically derived from strictosidine (**2**) as their common precursor.^[2]^ Ophiorrhines A,B are among the few monoterpene indole alkaloids which are biosynthetically produced by an oxidative cyclization between the tryptamine part and one of the double bond of the terpene part without deglucosylation such as cymoside (**3**) or 3‐α‐dihydrocadambine (**4**).^[3]^ These pathways are in contrast with the main one which involves a deglucosylation event of strictosidine which is usually followed by the condensation of the thus released aldehyde with the secondary amine N4 to give birth to the corynanthe skeleton of **5** and eventually to a large diversity of sub‐families of monoterpene indole alkaloids.^[2]^

**Scheme 2:**
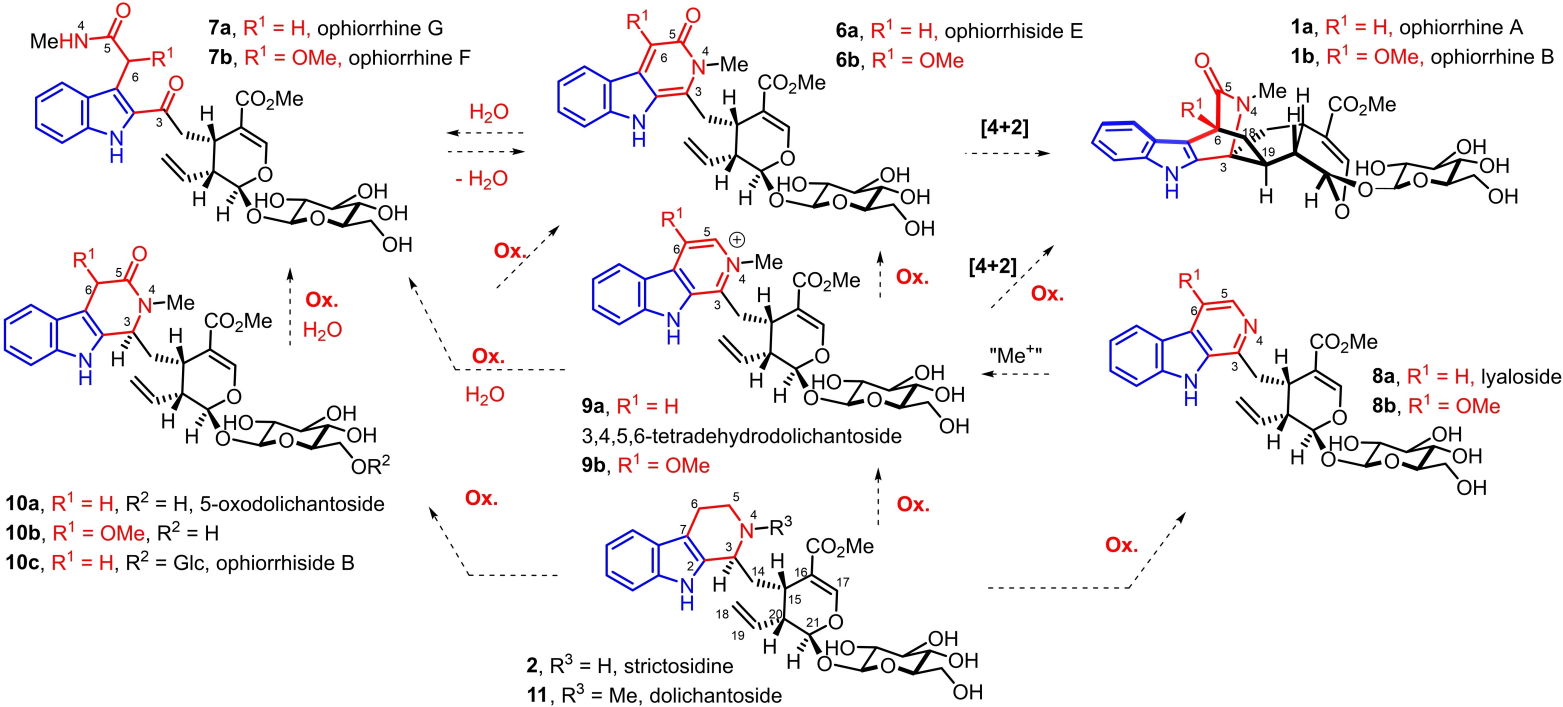
Postulated biosynthesis of ophiorrhines A,B and related monoterpene indole alkaloids from Ophiorrhiza genus.

Obviously, the biosynthesis of the azabicyclic[2.2.2]octanone central core of ophiorrhines A,B involves the intramolecular [4+2] Diels–Alder cycloaddition^[4]^ between the indolopyridone moiety and the C18=C19 terminal alkene of ophiorriside E (**6 a**) from *Ophiorrhiza trichocarpon* or its 6‐methoxy analogue **6 b** (Scheme 2).^[5]^ The recently discovered ophiorrhine G and F (**7 a**, **b**) from also *Ophiorrhiza japonica* appeared to be hydrolyzed forms of the indolopyridone moiety of the latters (**6 a**, **b**).^[6]^ Of importance, several related natural products isolated from the genus *Ophiorrhiza* ^[7]^ possess oxidation forms of the C‐piperidine ring of strictosidine,[^5^, ^8^] the biosynthetic precursor of the monoterpene indole alkaloids.

For instance, lyaloside (**8 a**)[^5^, ^8a^] and 3,4,5,6‐tetradehydrodolichantoside (**9 a**)[^5^, ^8b^] display respectively a pyridine and a N‐methyl pyridinium, while 5‐oxodolichantoside (**10 a**)[^1^, ^8c^] and ophiorrhiside B (**10 c**)^[5]^ present a N‐methylpiperidone. The precise biosynthetic interconnections between these compounds have yet to be determined.^[9]^ Among others postulates, the indolopyridone of ophiorrhiside E (**6 a**) could be produced by oxidation of either the N‐methylpyridinium of 3,4,5,6‐tetradehydrodolichantoside (**9 a**) or the piperidone of **10 a** or by intramolecular condensation of the N‐methyl amide of ophiorrhine G (**7 a**) onto its ketone.

Nevertheless, these biosynthetic hypotheses offer inspiration for organic chemists.

**Scheme 3:**
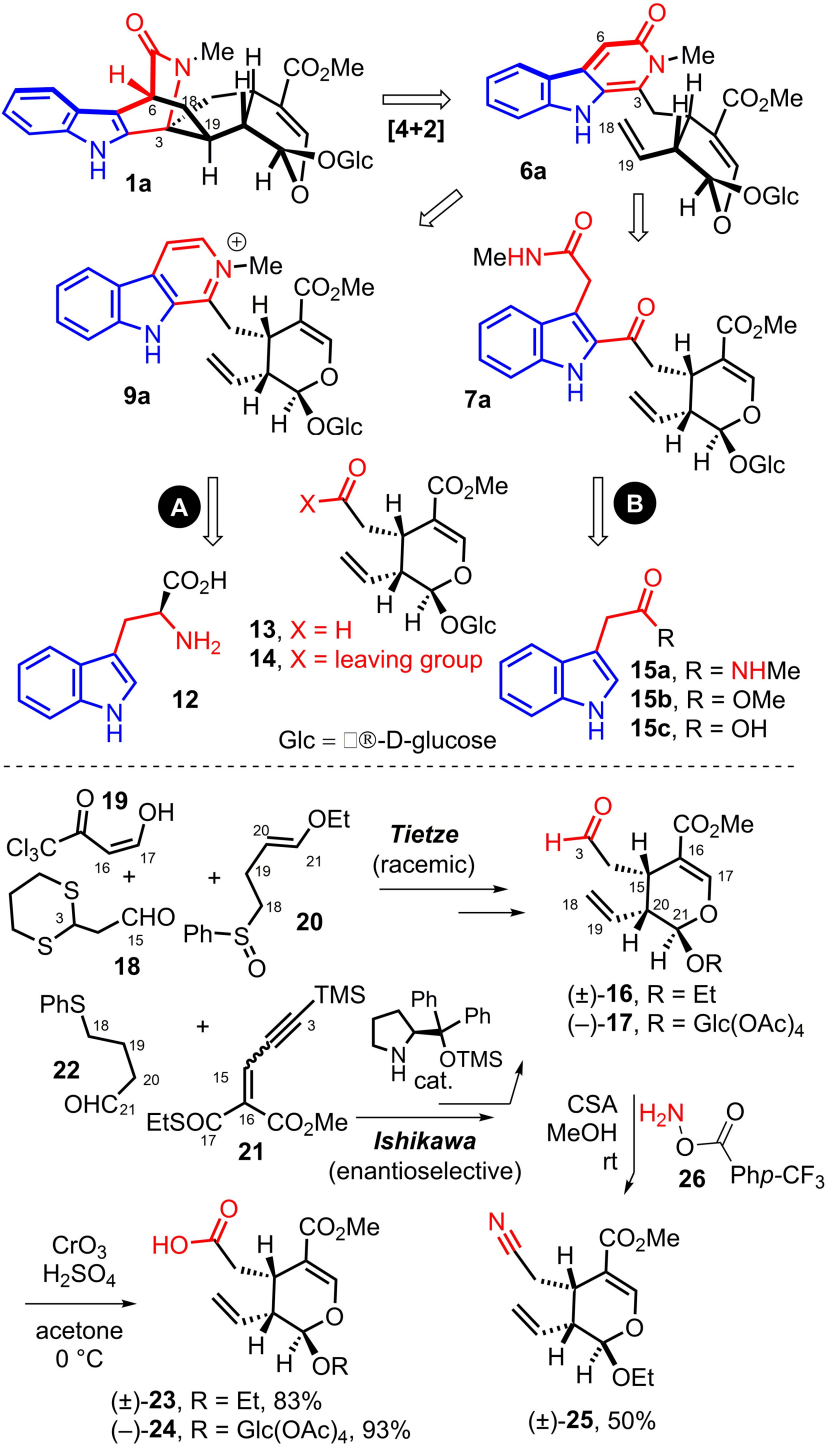
Retrosynthesis of ophiorrhine A (Glc=β‐d‐glucose).

In the context of our recent syntheses of monoterpene indole alkaloids^[10]^ including cymoside^[11]^ by bioinspired oxidative couplings, we proposed to access ophiorrhine A (**1**) through a bioinspired Diels–Alder cycloaddition from ophiorrhiside E (**6 a**, Scheme 3).^[12]^ It would represent a divergent total synthesis approach from strictosidine or secologanin in complement to the syntheses of cymoside and 3‐α‐dihydrocadambine.[^11^, ^13^]

## Results and Discussion

The key indolopyridone moiety of **6a** would be obtained through oxidation of the N‐methylpyridinium of **9 a** (Scheme 3, A) which would be synthesized from the oxidative decarboxylation of the Pictet–Spengler product of L‐tryptophan (**12**) and secologanin (**13**) followed by N‐methylation of the resulting pyridine of lyaloside (**8 a**). Alternatively, the indolopyridone of **6 a** is envisioned to be obtained via a cyclodehydration of ophiorrhine G (**7 a**) which would arise from the acylation of indolylacetic acid or its derivatives **15 a**–**c** with a carboxylic acid derivative **14** of secologanin (Scheme 3, B).

In the course of our study, we prepared both racemic secologanin aglycon ethyl ether (±)‐**16** and enantiopure tetra‐acetylated secologanin (−)‐**17**.^[14]^ The former was prepared according to Tietze via a hetero Diels–Alder cycloaddition between enol ether **20** and the enal derived from the condensation of formyl‐ketone **19** and aldehyde **18**.^[14a]^ In the other hand, the synthesis of (−)‐**17** relied on the work of Ishikawa via an organocatalytic enantioselective Michael addition of aldehyde **22** onto **21**.^[14b]^ Oxidation of the aldehyde of (±)‐**16** and (−)‐**17** led to the corresponding carboxylic acids (±)‐**23** and (−)‐**24**.^[15]^ Secologanin aglycone (±)‐**16** could also be converted into nitrile (±)‐**25** in 1 step with O‐benzoylhydroxylamine **26** in presence of camphorsulphonic acid (CSA).^[16]^

For each approaches, the feasibility to access the key indolopyridone moiety was first tested with a less complex substrate in lieu of the corresponding secologanin derivative.

**Scheme 4:**
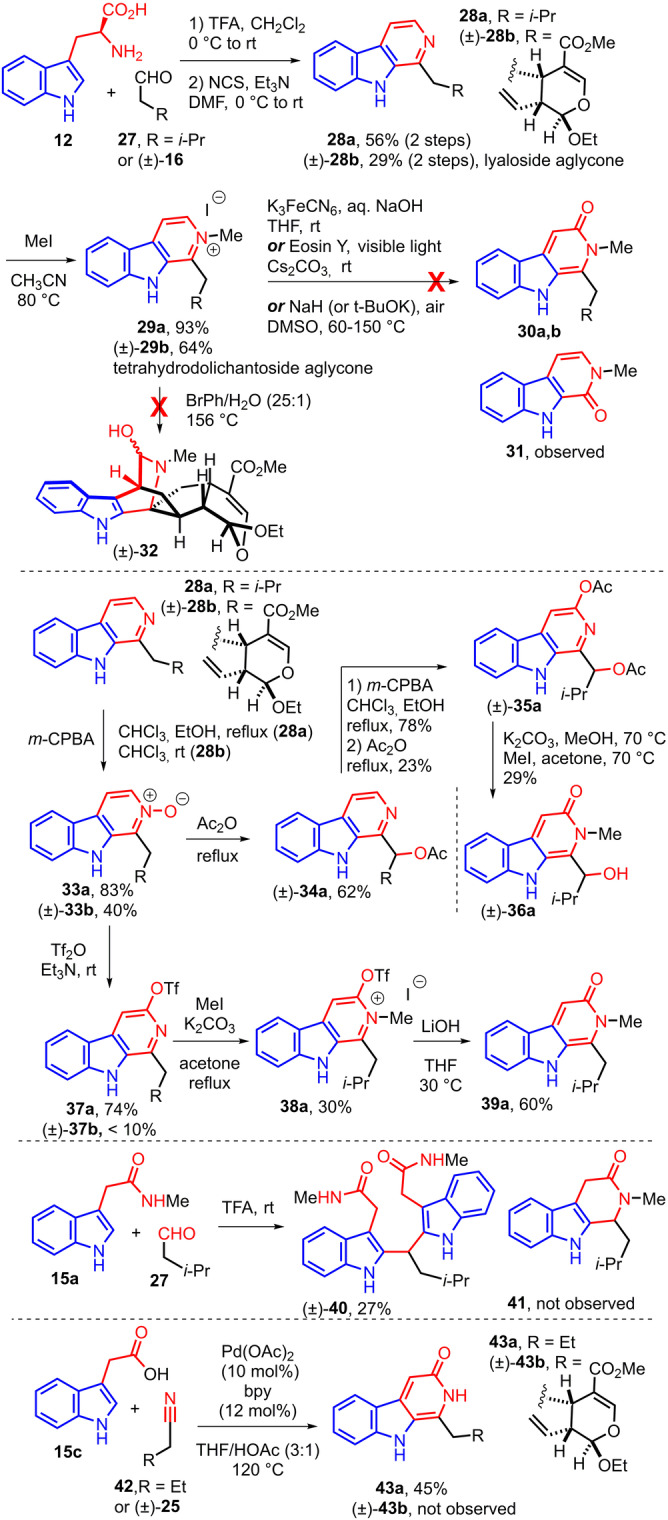
Attempts towards the synthesis of the indolopyridone moiety.

The indolopyridine approach (Scheme 3, A) involved the Pictet–Spengler reaction of L‐tryptophan **12** with *iso*‐valeraldehyde **27** or secologanin ethyl ether aglycone (±)‐**16** in trifluoroacetic acid (TFA, Scheme 4). It was followed by a decarboxylation and aromatization sequence mediated by N‐chlorosuccinimide (NCS) with triethylamine to produce the pyridine ring of **28 a** in 56 % yield and of lyaloside ethyl ether aglycone (±)‐**28 b** in 29 % yield.^[17]^

The corresponding N‐methylpyridinium salts **29 a** and 3,4,5,6‐tetrahydrodolichantoside aglycone ethyl ether (±)‐**29 b** were then obtained in 93 % and 64 % yields via reaction of the pyridine of **28 a**, **b** with excess of iodomethane at 80 °C in acetonitrile. We then pursued the pivotal oxidation of the indolopyrdinium ring into the desired indolopyridone. Known procedures for the oxidation of N‐methylpyridinium salts into pyridones^[18]^ were screened on **29 a** such as the use of potassium ferricyanide^[18a]^ or of Eosin Y as a photoredox catalyst under visible light irradiation^[18b]^ or heating in air with a strong base in dimethyl sulfoxide (DMSO).^[18c]^ Unfortunately, none of these conditions could allow to form the desired indolopyridones **30 a**, **b** with oxidation at the 5‐position. Only pyridone **31** could be observed resulting of oxidation at the more activated 3‐position followed by a C−C bond cleavage.

Therefore, we attempted to perform the intramolecular [4+2] cycloaddition at an earlier stage (Scheme 4). The N‐methyl pyridinium of lyaloside ethyl ether aglycone (**29 b**) was heated in a solution of bromobenzene and water (25 : 1) at 156 °C in order to perform a Bradsher cycloaddition.^[19]^ However, mainly starting material could be detected.

We envisioned that the formation of a pyridine‐N‐oxide and its reaction with anhydride acetic could lead the desired indolopyridone moiety via addition of an acetate to an N‐acetoxypyridinium intermediate.^[20]^ Indeed, reaction of pyridines **28 a** and (±)‐**28 b** with meta‐chloroperbenzoic acid (*m*‐CPBA) led respectively to pyridine N‐oxides **33 a** and (±)‐**33 b**. Heating of **33 a** in acetic anhydride (Ac_2_O) led to undesired α‐acetoxy pyridine (±)‐**34 a**.^[21]^ From (±)‐**34 a**, it was possible to effect the desired transformation via the same sequence of oxidation of the pyridine and then reflux in anhydride acetic to obtain acetoxypyridine (±)‐**35 a** which reaction with potassium carbonate and methyl iodide led to indolopyridone (±)‐**36 a**. However, this approach is not straightforward since an undesired alcohol is present on the substituent of the pyridone.

Nevertheless, switching acetic anhydride by triflic anhydride (Tf_2_O) allowed to convert selectively pyridine N‐oxide **33 a** at room temperature to the desired α‐triflyloxylpyridine **37 a** in 74 % yield.^[22]^ Methylation into **38 a** and treatment with lithium hydroxide led with delight to N‐methylpyridone **39 a**. However, we were disappointed that this sequence could not be implemented to the more complex secologanin‐derived pyridine N‐oxide (±)‐**33 b** since its treatment with triflic anhydride led to a complex mixture with less than 10 % of (±)‐**37 b**.

We then turned our attention towards the reaction of indolylacetic acid derivatives **15 a**–**c** with carbonyl reagents according to the alternative retrosynthesis (Scheme 3, B).

Without surprised the acid‐mediated reaction between *iso*‐valeraldehyde aldehyde **27** and indolylacetamide **15 a** ^[23]^ did not lead to indolopiperidone **41** but to the double addition of the indole of **15 a** to the carbonyl of **27** (Scheme 4).

Very interestingly, the palladium catalyzed reaction of indolylocarboxylic acid **15 c** with butyronitrile **42** led to indolopyridone **43 a** with bipyridine as ligand (bpy).^[24]^ Unfortunately, the same reaction with secologanin‐derived nitrile (±)‐**34 b** did not succeeded (Scheme 4).

**Scheme 5:**
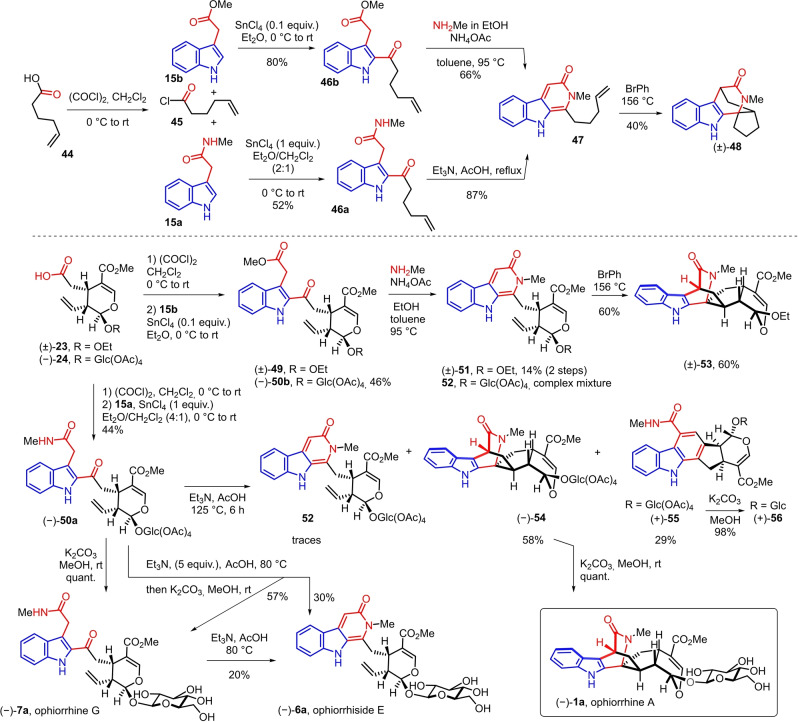
Total synthesis of ophiorrhine A via a cyclodehydration/Diels–Alder cycloaddition from protected ophiorrhine G.

Eventually direct acylation of **15 a**, **b** with a carboxylic acid into a 2‐acylindole was considered (Scheme 5). As a test substrate, we selected 5‐hexenoic acid **44** which leads to a suitable substrate model for the intramolecular Diels–Alder cycloaddition.

After optimization, we were able to perform the Friedel–Crafts‐type coupling of indolyl acetic acid methyl ester **15 b** with 5‐hexenoic acid chloride **45** in presence of a catalytic amount of tin tetrachloride to furnish **46 b** in 80 % yield.^[25]^ The same acylation reaction from indolylacetamide **15 a** required a stoichiometric amount of tin tetrachloride and delivered the 2‐acylated indole **46 a** in 52 % yield.

Both **46 a** and **46 b** could be converted efficiently into the expected indolopyridone **47**, either via reaction of methyl ester **46 b** with excess of an ethanolic solution of methylamine and ammonium acetate at 95 °C in toluene or by cyclodehydration of keto‐amide **46 a** with triethylamine in reflux of acetic acid.[^26^, ^27^] In the literature, only one example of a Diels–Alder cycloaddition of indolopyridone is reported with electron‐poor dienophiles^[28]^ while our present approach involves an electron‐rich dienophile. The aromatic character of the 2‐pyridone moiety part via tautomeric equilibrium necessitates to supply a high activation energy such as a high temperature to accomplish the [4+2] cycloaddition.^[29]^ Nevertheless, it has been shown that the presence of a N‐alkyl substituent could enable the cycloaddition of 2‐pyridones. In addition, in these harsh reaction conditions, avoiding the retro Diels–Alder reaction of the cycloadduct with extrusion of methyl isocyanate is another challenge to overcome.[^28^, ^29^] Rewardingly, the key intramolecular Diels–Alder cycloaddition was achieved via heating indolopyridone **47** in bromobenzene at 156 °C leading to azabicyclo[2.2.2]octenone skeleton of (±)‐**48** as one diastereoisomer which relative stereochemistry could not be determined.

Having demonstrated the proof of principle with a simplified carboxylic acid, we then aimed to implement our strategy to the secologanin template (Scheme 5). Based on the results on the model substrate, indolyl acetic acid methyl ester **15 b** seemed to be a more suitable substrate for the acylation than the corresponding amide **15 a**. Thus, the acid of secologanin aglycon ethyl ether (±)‐**23 a** was converted into the corresponding acid chloride and the Friedel–Crafts‐type reaction with **15 b** in presence of a catalytic amount of tin tetrachloride followed by treatment with methylamine allowed to obtain ophiorrhiside E aglycone ethyl ether (±)‐**51** in 14 % over two steps. Heating of the latter in bromobenzene at 156 °C delivered stereoselectively ophiorrhine A aglycon ethyl ether (±)‐**53 a** in 60 %.^[30]^ Unfortunately, the same sequence could not be applied to protected secologanin acid (−)‐**24**. While the acylation of **15 b** furnished (−)‐**50 b** in 46 %, the reaction of the latter with methyl amine could not deliver protected ophiorrhiside E (−)‐**52**, only decomposition or partial deacetylation of protected sugar were observed. Therefore, we believed that starting the sequence from a substrate already containing the acetamide functionality would prevent this problem. Rewardingly, reaction of **15 a** with the acid chloride of protected secologanin acid (−)‐**24** with a stoichiometric amount of tin tetrachloride allowed us to obtain, in 44 % yield, (−)‐**50 a**, which deacetylation with potassium carbonate in methanol could yield quantitatively ophiorrhine G **7 a**.^[31]^

Formation of the key indolopyridone motif was sought by cyclodehydration of 2‐(2‐acyl‐3‐indolyl)‐acetamide (−)‐**50 a** with triethylamine in acetic acid according to the conditions developed on the model substrate **46 a**. Thus, (−)‐**50 a** was submitted to heating at 125 °C with a large excess of triethylamine in acetic acid for 6 h. Surprisingly, only traces of indolopyridone **52** were observed in these conditions. To our delight, the successful cyclodehydration was followed by the spontaneous bioinspired diastereoselective intramolecular Diels–Alder cycloaddition of transient **52** to deliver the desired spirocyclic azabicyclic[2.2.2]octanone (−)‐**54** in 58 % yield. Finally, the total synthesis of ophiorrhine A (−)‐**1 a** was achieved via quantitative methanolysis of the four acetate of the glucose moiety of (−)‐**54**.^[31]^

**Scheme 6:**
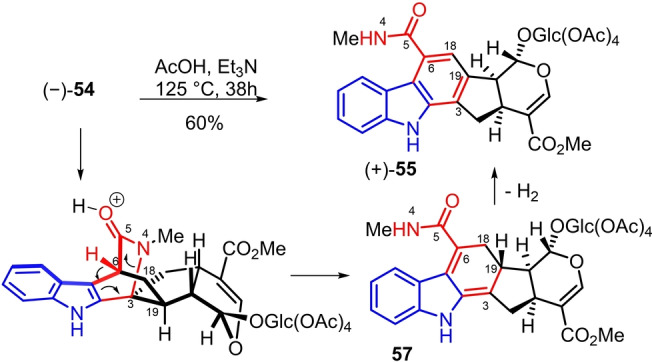
Formation of carbazole (+)‐**55** via aromatizing ring opening of the bicyclo[2.2.2]template of ophiorrhine A.

Intriguingly, during the dehydrative intramolecular condensation/Diels–Alder sequence, carbazole (+)‐**55** was also observed which could be methanolysed into (+)‐**56** in 29 % over 2 steps. We demonstrated that carbazole (+)‐**55** could be formed from azabicyclic[2.2.2]octanone (−)‐**54** in reflux of acetic acid and triethylamine (Scheme 6). The benzamide part of (+)‐**55** probably raised from the ring opening of the azabicyclic[2.2.2]octanone via cleavage of the N4−C3 bond followed by aromatizing C18−C19 dehydrogenation of **57**. This remarkable bond reorganization delivers an original strictosidine‐derived skeleton which is not a natural product or yet to be discovered from natural sources.^[32]^ This mechanism is in contrast with the anticipated retro Diels–Alder reaction from (−)‐**54** with extrusion of methyl isocyanate which was not observed in our case.[^28^, ^29^]

In order to form indolopyridone **52** and prevent the spontaneous Diels–Alder cycloaddition, the cyclodehydration had to be performed at a lower temperature (80 °C) after which, removal of the four acetates with methanol and potassium carbonate led to ophiorrhiside E **6 a** in 30 % over two steps (Scheme 5). At 80 °C, the conversion of (−)‐**50 a** into **52** is modest since 57 % of ophiorrhine G **7 a** was also isolated. Eventually, ophiorrhiside E **6 a** could also be obtained in 20 % via cyclodehydration at 80 °C of ophiorrhine G **7 a**.^[31]^

## Conclusion

In conclusion, we performed the first total synthesis of ophiorrhines A and G, as well as ophiorrhiside E. Ophiorrhine A and its spirocyclic ring systems were accessed through a bioinspired intramolecular Diels–Alder cycloaddition between the pyridone moiety and terminal alkene of protected ophiorrhiside E. The latter was obtained through acylation of N‐methyl indolylacetamide by a secologanin derivative followed by the cyclodehydration of the resulting protected ophiorrhine G.

## Conflict of interest

The authors declare no conflict of interest.

1

## Supporting information

As a service to our authors and readers, this journal provides supporting information supplied by the authors. Such materials are peer reviewed and may be re‐organized for online delivery, but are not copy‐edited or typeset. Technical support issues arising from supporting information (other than missing files) should be addressed to the authors.

Supporting Information

## Data Availability

The data that support the findings of this study are available from the corresponding author upon reasonable request.
