# Supplementary material for: Total Synthesis of Ophiorrhine A, G and Ophiorrhiside E Featuring a Bioinspired Intramolecular Diels–Alder Cycloaddition
Source: Angew Chem Int Ed Engl. 2022 Aug 10;61(38):e202209135. doi: 10.1002/anie.202209135 (PMC9543224; doi:10.1002/anie.202209135)
Supplement: Supplementary file 1 — Supporting Information [file ANIE-61-0-s001.pdf]

Angewandte Chemie
Eine Zeitschrift der Gesellschaft Deutscher Chemiker

Supporting Information

Total Synthesis of Ophiorrhine A, G and Ophiorrhiside E Featuring a Bioinspired Intramolecular Diels–Alder Cycloaddition

*W. Cao, Y. Dou, C. Kouklovsky, G. Vincent**

Supporting Information

Total Synthesis of Ophiorrhine A, G and Ophiorrhiside E Featuring a Bioinspired Intramolecular Diels-Alder Cycloaddition

*Wei Cao, Yingchao Dou, Cyrille Kouklovsky, and Guillaume Vincent**

Institut de Chimie Moléculaire et des Matériaux d'Orsay
Université Paris-Saclay, CNRS
91405 Orsay France.
guillaume.vincent@universite-paris-saclay.fr

Contents

1. General information 3

2. Optimizations of reaction conditions 4

3. Experimental procedures and data of all compounds. 9

4. Comparison of ^{1}H, ^{13}C NMR data and optical rotations for natural and synthetic products .. 34

5. NMR spectra of all compounds 40

6. References 97

1. General information

All reactions were carried out in anhydrous solvents under an inert argon atmosphere unless otherwise stated. Tetrahydrofuran (THF) and diethyl ether (Et_2O) were distilled from sodium-benzophenone before use. Dichloromethane (CH_2Cl_2) and toluene (PhMe) were distilled from calcium hydride before use. Methanol (MeOH) and acetone were distilled from calcium hydride and stored under argon atmosphere. All others commercial reagent-grade chemicals and solvents were used directly without further treatment unless noted. Reactions were monitored with analytical thin-layer chromatography (TLC) on silica gel 60 F254 plates and visualized under UV (254 nm) and/or by staining with phosphomolybdic acid hydrate solution in EtOH followed by heating or with $KMnO_4$ in a K_2CO_3 and NaOH aqueous solution. Flash chromatography were performed on silica gel 60 [63-200 µm]) as stationary phase. Preparative thin-layer chromatography (prep. TLC) were performed on silica gel 60 F254 plates. NMR spectra were recorded on Bruker AVANCE I 250 (250 MHz for ^{1}H), Bruker AVANCE I 300 (300 MHz for ^{1}H and 75 MHz for ^{13}C), Bruker AVANCE I 360 (360 MHz for ^{1}H and 90 MHz for ^{13}C) and Bruker AVANCE I 400 (400 MHz for ^{1}H and 100 MHz for ^{13}C) instruments, at 295 K. Chemical shifts were reported in part per million relative to residual peak ($CDCl_3$: ^{1}H δ 7.26 ppm, ^{13}C δ 77.16 ppm; CD_3OD: ^{1}H δ 3.31 ppm, ^{13}C δ 49.0 ppm). The mentioned abbreviations were as follows: s (singlet), d (doublet), t (triplet), q (quartet), m (multiplet), br (broad). High-Resolution mass spectra (HRMS) were measured on a Bruker Daltonics MicrOTOF-Q instrument by means of Electrospray Ionization (ESI) technic, with accurate masses reported for molecular ion $[M+H]^+$ or $[M+Na]^+$. $[\alpha]_D$ were recorded on an Anton Paar MCP150 polarimeter.

2. Optimizations of reaction conditions

1.1 Attempts towards the oxidation of N-methyl pyridinium **29** into pyridone **30**

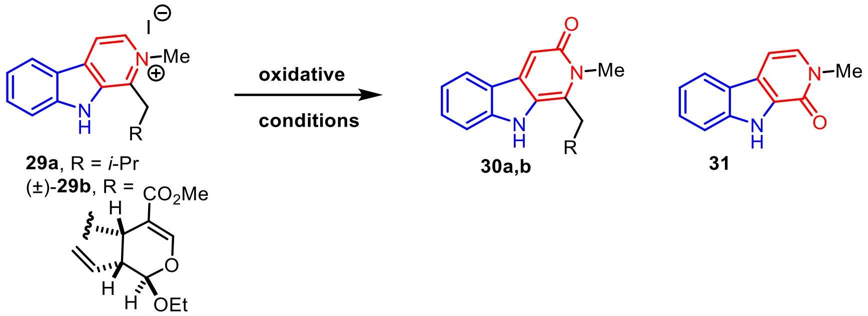

***Table S1**. Attempts with $K_3Fe(CN)_6$*

Entry	Substrate	Oxidative Conditions	Yield of 30[a]
1	**29a**	2 eq. $K_3Fe(CN)_6$, 3 equiv. NaOH, THF/H_2O (2/1), r.t., 12h	N.D.
2	**29a**	4 eq. $K_3Fe(CN)_6$, 6 equiv. NaOH, THF/H_2O (2/1), 40 °C, overnight	N.D.
3	**29a**	3 eq. $K_3Fe(CN)_6$, 0.5 mL H_2SO_4 (60%), r.t., 4h	N.D.
4	**29b**	11 eq. $K_3Fe(CN)_6$, 11 equiv. NaOH, THF/H_2O (2/1), r.t., 12h	Complex mixture

Reaction conditions: 0.025 mmol of **29** and 1.0 mL solvent were used.
a: isolated yield.
N.D. = No detected.

***Table S2.** Attempts with a base in air.*

Entry	Substrate	Oxidative Conditions	Yield of 30[a]
1	**29a**	2 eq. *t*-BuOK, DMSO, r.t., air, 24 h	N.D.
2	**29a**	2 eq. *t*-BuOK, DMSO, 60 °C, air, 72 h	N.D.
3	**29a**	4 eq. Et_3N, DMSO, 150 °C, air, 3 h	N.R.
4[b]	**29a**	4 eq. NaH, DMSO, 150 °C, air, 7 h	N.D (74% of **31**)

Reaction conditions: 0.01 mmol of **29** and 0.5 mL solvent were used.
a: isolated yield.
N.D. = No detected.
N.R. = No reaction.

***Table S3.** Attempts with a photoredox catalyst.*

Entry	Substrate	Oxidative Conditions	Yield of 30[a]
1	**29a**	0.1 eq. 2-hydroxybenzaldehyde, 1.5 eq. K_3PO_4, DMSO, air, r.t., LED light, 72h	N.R.
2	**29a**	0.02 eq. EosinY, 1.5 eq. Cs_2CO_3, DMSO, air, r.t., LED light, 24h	N.D. (10% of **31**)
3	**29a**	0.02 eq. EosinY, 1.5 eq. Cs_2CO_3, THF, air, r.t., LED light, 24h	N.D. (10% of **31**)
4	**29b**	0.02 eq. EosinY, 1.5 eq Cs_2CO_3, THF, air, r.t., LED light, 24h	Complex mixture

Reaction conditions: 0.05 mmol of **29** and 1.0 mL solvent were used.
a: isolated yield.
N.D. = No detected.
N.R. = No reaction.

***Table S4.** Attempts in electrochemical conditions.*

Entry	Substrate	Oxidative Conditions	Yield of 30[a]
1	**29a**	Graphite (anode) and Pt (cathode), 20 mA O_2, 1.5 eq Cs_2CO_3, 5 eq. Bu_4NBF_4, MeCN/H_2O (4/1)	N.R.
2	**29a**	Graphite (anode) and Pt (cathode), 20 mA O_2, 5 eq. KI, 1.5 eq Cs_2CO_3, MeCN/H_2O (4/1)	N.R.

Reaction conditions: 0.15 mmol of **29a** and 3.0 mL solvent were used.
a: isolated yield.
N.R. = No reaction.

1.2 Conversion of pyridine **28** into triflyloxypyridine **37** via pyridine N-oxide **33**

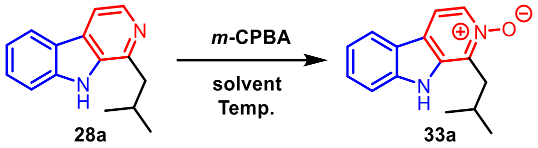

Table S5. *Optimization of the oxidation of pyridine **28a** into pyridine N-oxide **33a**.*

Entry	Solvent	Temp.	Eq. *m*-CPBA	Yield of 33a[a]
1	$CHCl_3$	rt	3.0	62%
2	CH_2Cl_2	rt	3.0	46%
3	$CHCl_3$:EtOH (1:1)	reflux	3.0	75%
4[b]	$CHCl_3$:EtOH (1:1)	reflux	5.0	83%

Reaction Conditions: **28a** (0.1 mmol) in solvent (c = 0.1M), overnight.
a. Isolated yields.
b. 1.0 mmol of **28a** was used.

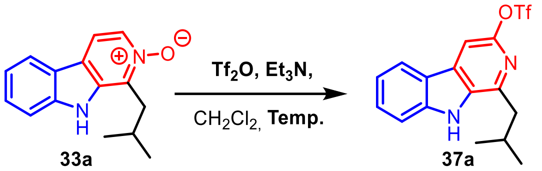

Table S6. *Optimization of synthesis of triflyloxypyridine **37a** from pyridine N-oxide **33a** with Tf_2O.*

Entry	Eq. Tf_2O	Eq. Et_3N	Temp.	Yield of 37a[a]
1	1.1	2.2	- 78 °C	23%
2	5	10	- 78 °C	25%
3	5	10	0 °C	38%
4	5	10	r.t.	74%
5	1.1	2.2	r.t.	27%
6	3	6	r.t.	59%
7	7	14	r.t.	26%

Reaction Conditions: **33a** (0.1 mmol) and 1 mL of CH_2Cl_2 were used.
a. Isolated yields.

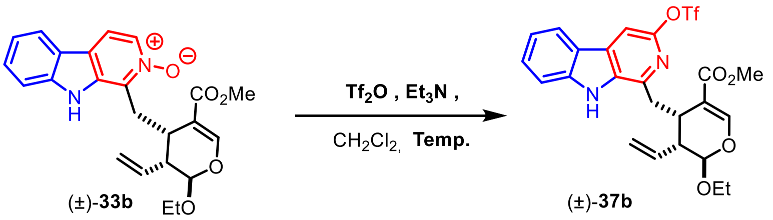

Table S7. *Optimization of synthesis of triflyloxypyridine **37b** from pyridine N-oxide **33b** with Tf_2O*

Entry	Eq. Tf_2O	Eq. Et_3N	Temp.	Yield of 37b[a]
1	1.2	2.4	r.t.	trace
2	5	10	r.t.	trace
3	5	10	0 °C	7%

Reaction Conditions: **33b** (0.025 mmol) and 1 mL of CH_2Cl_2 were used.
a. Isolated yields.

1.3 Synthesis of hydroxypyridine **43a** via A Pd-catalyzed coupling of indolyacetic acid with a nitrile

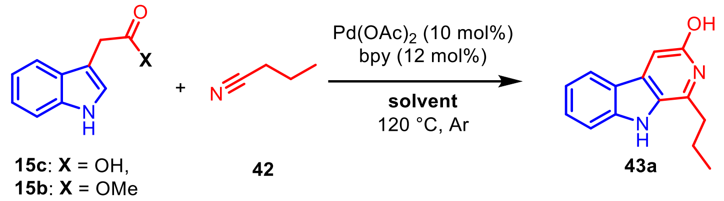

***Table S8**. Optimization of Pd-catalyzed synthesis of **43a** from **15b/c** and **42**.*

Entry	15b or 15c, eq.	Solvent	Yield of 43a[c]
1	**15b**, 0.67	THF/HOAc （3:1）	17%
2	**15c**, 0.67	THF/HOAc （3:1）	22%
3	**15c**, 5.00	THF/HOAc （3:1）	45%
4	**15c**, 5.00	THF	5%
5	**15c**, 5.00	NMF	trace
6[a]	**15c**, 5.00	THF	trace
6[a]	**15c**, 5.00	NMF	trace
8[b]	**15c**, 5.00	THF/HOAc （3:1）	N.D.

Reaction conditions: 0.6 mmol of **42**, 0.06 mmol of $Pd(OAc)_2$, 0.072 mmol of bpy and 1.6 mL solvent were used.
a: 10 % TFA was used.
b: (*E*)-*N*,3,7-trimethyloct-6-en-1-imine was used instead of nitrile **42**.
c: isolated yield.
N.D. = No detected.

1.4 Acylation of indolylacetic acid derivatives with a carboxylic acid

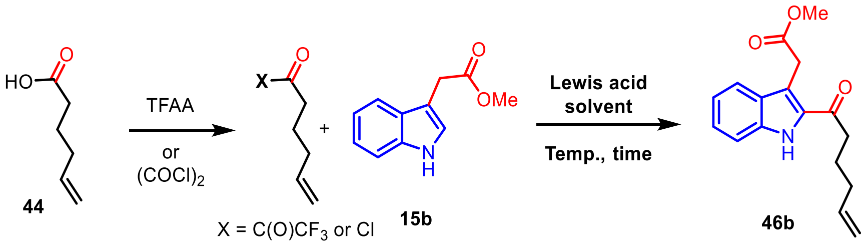

***Table S9.** Optimization of the acylation of indolylacetic acid methyl ester **15b** with 5-hexenoic acid **44**.*

Entry	X	Lewis acid	Solvent	Temp.	Time	Yield of 46b[a]
1	$C(O)CF_3$	3 eq. $ZnCl_2$	CH_2Cl_2	r.t.	24 h	11%
2	$C(O)CF_3$	3 eq. $BF_3{\cdot}OEt_2$	CH_2Cl_2	r.t.	24 h	12%
3	Cl	0.1 eq. $Bi(OTf)_3$	$MeNO_2$	0 °C	40 min	decomposed
4	Cl	5.0 eq. $TiCl_4$	Et_2O	0 °C to r.t.	overnight	Trace
5	Cl	2.0 eq. $TiCl_4$	Et_2O	0 °C to r.t.	overnight	17%
6	Cl	1.0 eq. $TiCl_4$	Et_2O	0 °C to r.t.	overnight	35%
7	Cl	0.5 eq. $TiCl_4$	Et_2O	0 °C to r.t.	overnight	35%
8	Cl	0.3 eq. $TiCl_4$	Et_2O	0 °C to r.t.	overnight	56%
9	Cl	0.1 eq. $TiCl_4$	Et_2O	0 °C to r.t.,	overnight	59%
10	Cl	0.05 eq. $TiCl_4$	Et_2O	0 °C to r.t.,	overnight	56%
11	Cl	0.1 eq. $TiCl_4$	Et_2O	0 °C to r.t.	overweekend	63%
12	Cl	0.03 eq. $SnCl_4$	Et_2O	0 °C to r.t.	overnight	42%
13	Cl	0.05 eq. $SnCl_4$	Et_2O	0 °C to r.t.	overnight	49%
14	Cl	0.07 eq. $SnCl_4$	Et_2O	0 °C to r.t.	overnight	56%
15	Cl	0.09 eq. $SnCl_4$	Et_2O	0 °C to r.t.	overnight	56%
16	Cl	0.1 eq. $SnCl_4$	Et_2O	0 °C to r.t.	overnight	80%
17	Cl	0.3 eq. $SnCl_4$	Et_2O	0 °C to r.t.	overnight	73%
18	Cl	0.5 eq. $SnCl_4$	Et_2O	0 °C to r.t.	overnight	63%
19	Cl	1.0 eq. $SnCl_4$	Et_2O	0 °C to r.t.	overnight	56%

Reaction conditions (entries 1-2): 0.6 mmol of **15b**, 0.5 mmol of carboxylic acid, 0.65 mmol of trifluoroacetic anhydride and 1 mL solvent were used.

Reaction conditions (entries 3-19): 0.1 mmol of **15b**, 0.12 mmol of carboxylic acid, 0.156 mmol of oxalyl chloride and 1 mL solvent were used.
a: Isolated yield.

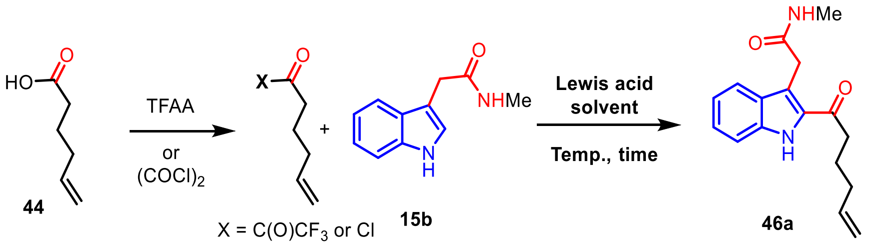

Table S10. *Optimization of the acylation of indolylacetamide* ***15a*** *with 5-hexenoic acid* ***44****.*

Entry	X	Lewis acid	Solvent	Temp.	Time	Yield of 46a[a]
1	C(O)CF$_3$	3 eq. $ZnCl_2$	CH_2Cl_2	r.t.	24 h	trace
2	C(O)CF$_3$	3 eq. $BF_3{\cdot}OEt_2$	CH_2Cl_2	r.t.	24 h	trace
3	Cl	0.1 eq. Bi(OTf)$_3$	$MeNO_2$	0 °C	overweekend	N.D.
4	Cl	3.0 eq. $SnCl_4$	Et_2O	0 °C to r.t.	overnight	decomposed
5	Cl	2.5 eq. $SnCl_4$	Et_2O : CH_2Cl_2 (3:1)	0 °C to r.t.	4 h	24%
6	Cl	2.0 eq. $SnCl_4$	Et_2O : CH_2Cl_2 (3:1)	0 °C to r.t.	4 h	10%
7	Cl	1.5 eq. $SnCl_4$	Et_2O : CH_2Cl_2 (3:1)	0 °C to r.t.	4 h	14%
8	Cl	1.0 eq. $SnCl_4$	Et_2O : CH_2Cl_2 (3:1)	0 °C to r.t.	4 h	45%
9	Cl	0.5 eq. $SnCl_4$	Et_2O : CH_2Cl_2 (3:1)	0 °C to r.t.	4 h	38%
10	Cl	1.0 eq. $SnCl_4$	Et_2O	0 °C to r.t.	4 h	10%
11	Cl	1.0 eq. $SnCl_4$	CH_2Cl_2	0 °C to r.t.	4 h	7%
12	Cl	1.0 eq. $SnCl_4$	Et_2O : CH_2Cl_2 (2:1)	0 °C to r.t.	4 h	52%
13	Cl	1.0 eq. $SnCl_4$	Et_2O : CH_2Cl_2 (1:1)	0 °C to r.t.	4 h	52%
14	Cl	1.0 eq. $SnCl_4$	Et_2O : CH_2Cl_2 (1:2)	0 °C to r.t.	4 h	14%
15	Cl	1.0 eq. $SnCl_4$	Et_2O : CH_2Cl_2 (1:3)	0 °C to r.t.	4 h	7%
16	Cl	1.0 eq. $SnCl_4$	Et_2O : CH_2Cl_2 (3:1)	0 °C to r.t.	overnight	45%
17	Cl	2.5 eq. $SnCl_4$	Et_2O : CH_2Cl_2 (3:1)	0 °C to r.t.	4 h	24%
18	Cl	2.0 eq. $SnCl_4$	Et_2O : CH_2Cl_2 (3:1)	0 °C to r.t.	4 h	10%
19	Cl	1.5 eq. $SnCl_4$	Et_2O : CH_2Cl_2 (3:1)	0 °C to r.t.	4 h	14%
20	Cl	1.0 eq. $SnCl_4$	Et_2O : CH_2Cl_2 (3:1)	0 °C to r.t.	4 h	45%
21	Cl	0.5 eq. $SnCl_4$	Et_2O : CH_2Cl_2 (3:1)	0 °C to r.t.	4 h	38%

Reaction conditions (entries 1-2): 0.6 mmol of **15a**, 0.5 mmol of carboxylic acid, 0.65 mmol of trifluoroacetic anhydride and 1 mL solvent were used.
Reaction conditions (entries 3-21): 0.12 mmol of **15a**, 0.10 mmol of carboxylic acid, 0.13 mmol of oxalyl chloride and 1 mL solvent were used.
a: Isolated yield.
N.D. = No Detected.

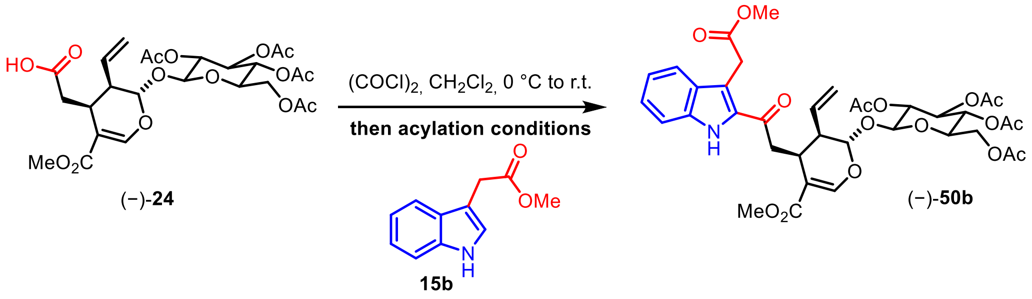

Table S11. *Optimization of acylation of indolylacetic acid methyl ester* ***15b*** *with* ***24****.*

Entry	Conditions	Yield of 50b[a]	Recovered 24[a]
1	$TiCl_4$ (0.1 eq.), Et_2O, 0 °C to r.t., overnight	16%	70%
2	$SnCl_4$ (0.1 eq.), Et_2O, 0 °C to r.t., overnight	27%	56%
3	$SnCl_4$ (0.05 eq.), Et_2O, 0 °C to r.t., overnight	27%	38%

4[b]	$SnCl_4$ (0.1 eq.), Et_2O, 0 °C to r.t., 2 h	47%	14%

Reaction conditions: 0.12 mmol of **15b**, 0.1 mmol of carboxylic acid, 0.156 mmol of oxalyl chloride and 1 mL solvent were used.

a: isolated yield.

b: 0.2 mmol of oxalyl chloride was used.

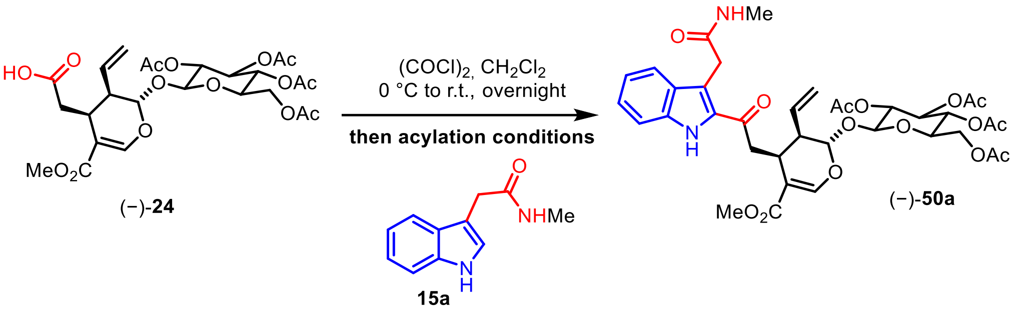

Table S12. *Optimization of acylation of indolylacetamide* ***15a*** *with* ***24***.

Entry	Conditions	Yield of 50a[a]
1	1.0 eq. $SnCl_4$, Et_2O : CH_2Cl_2 (1:1), 0 °C to r.t., 4 h	9%
2	1.0 eq. $SnCl_4$, Et_2O : CH_2Cl_2 (1:1), 0 °C to r.t., 24 h	33%
3	1.2 eq. $SnCl_4$, Et_2O : CH_2Cl_2 (1:1), 0 °C to r.t., 4 h	6%
4	8.0 eq. $SnCl_4$, Et_2O : CH_2Cl_2 (1:1), 0 °C, 10 min	33%
5	1.0 eq. $SnCl_4$, Et_2O : CH_2Cl_2 (3:1), 0 °C to r.t., 24 h	40%
6	2.0 eq. $SnCl_4$, Et_2O : CH_2Cl_2 (3:1), -20 °C (5 h) to 0 °C (19 h)	44%
7	1.0 eq. $SnCl_4$, Et_2O : CH_2Cl_2 (4:1), 0 °C to r.t., 24h	44%
8	1.0 eq. $SnCl_4$, Et_2O, 0 °C to r.t., 24 h	33%
9	2.0 eq. $SnCl_4$, Et_2O : CH_2Cl_2 (4:1), 0 °C, 24 h	40%
10	2.0 eq. $SnCl_4$, Et_2O: CH_2Cl_2 (4:1), -20 °C, 24 h	30%

Reaction conditions: Entries 1 and 3-10: 0.08 mmol of **15a**, 0.04 mmol of carboxylic acid, 0.08 mmol of oxalyl chloride and 0.5 mL solvent were used. Entry 2: 0.16 mmol of **15a**, 0.08 mmol of carboxylic acid, 0.16 mmol of oxalyl chloride and 1.0 mL solvent were used.

a: Isolated yield.

1.5 Cyclodehydration of protected ophiorrhine G and tandem Diels-Alder cycloaddition into protected ophiorrhine A.

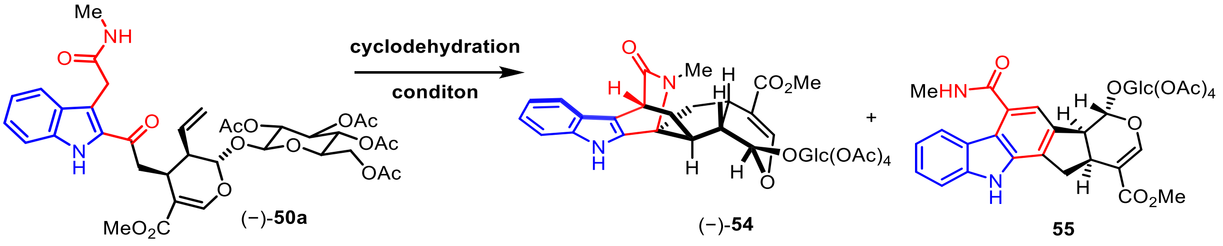

Table S13. *Optimization of the cyclodehydration/[4+2] cycloaddition of* ***50a*** *into* ***54***.

Entry	Cyclodehydration Conditions	Recovered 50a[a]	Yield of 54[a]	Yield of 55[a]
1	5 eq. Et_3N, AcOH, 80 °C, 3h	77%	0%	N.D.
2	5 eq. Et_3N AcOH, r.t. to 115 °C, 2 h	40%	20%	trace
3	45 eq. Et_3N (added in 3 times), AcOH， 115 °C, 3 h	30%	40%	trace
4	45 eq. Et_3N (added in 3 times), AcOH, 80 °C, 18 h	41%	8%	N.D.
5	45 eq. Et_3N, AcOH, 125 °C, 6 h	trace	58%	29%
6	200 eq. Et_3N, 200 eq. AcOH, 80 °C, 18h	50%	25%	N.D.
7	5 eq. Et_3N, AcOH, 80 °C, 43h	22%	45%	N.D.

a: Isolated yield.

3. Experimental procedures and data of all compounds.

Compound 28a: 1-isobutyl-9*H*-pyrido[3,4-*b*]indole

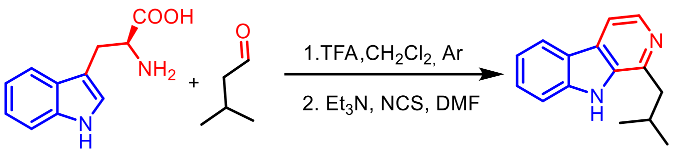

Inspired from a known a procedure.[1]
To a solution of the *L*-tryptophan **12** (1.02 g, 5.00 mmol) in CH_2Cl_2 (25 mL) at room temperature was added 3-methylbutanal **27** (1.07 mL, 10 mmol), and then TFA (1.48 mL, 20 mmol) dropwise at 0 ºC. The temperature was then allowed to warm up to room temperature and the reaction mixture was stirred at this temperature overnight. Then, the reaction mixture was concentrated under reduced pressure to remove TFA and CH_2Cl_2. The mixture was dissolved in 50 mL of DMF, followed by addition of NCS (1.4 g, 10.5 mmol) and Et_3N (1.75 mL, 12.5 mmol) dropwise at 0 ºC. The mixture was stirred at room temperature for 3 h and then quenched with water. The mixture was diluted with EtOAc and washed 3 times with a saturated aqueous solution of NaCl after which the organic layer was dried over Na_2SO_4, filtered and concentrated under reduced pressure. The residue was purified by column chromatography on silica gel (CH_2Cl_2/MeOH 25:1 to 20:1) to give 627 mg of **28a** as a colorless solid (56% yield).

R_f = 0.41 (Petroleum ether/ Ethyl acetate 1:1)
^{1}H NMR (360 MHz, MeOD) δ 8.16 (d, *J* = 5.4 Hz, 1H), 8.08 (dt, *J* = 8.1, 1.1 Hz, 1H), 7.86 (d, *J* = 5.4 Hz, 1H), 7.56 (dt, *J* = 8.1, 1.1 Hz, 1H), 7.50 (ddd, *J* = 8.1, 6.9, 1.1 Hz, 1H), 7.20 (ddd, *J* = 8.1, 6.9, 1.1 Hz, 1H), 2.98 (d, *J* = 7.5 Hz, 2H), 2.31 – 2.18 (m, 1H), 0.97 (d, *J* = 6.6 Hz, 6H).
^{13}C NMR (90 MHz, MeOD) δ 146.5, 142.5, 137.8, 136.3, 130.0, 129.4, 122.6, 120.6, 114.0, 112.8, 43.5, 30.2, 22.9 (2C).
HRMS (m/z): $[M + H]^+$ calcd. for $C_{15}H_{17}N_2^+$ 225.1386, found 225.1380.

Compound 29a: 1-isobutyl-2-methyl-9*H*-pyrido[3,4-*b*]indol-2-ium iodide

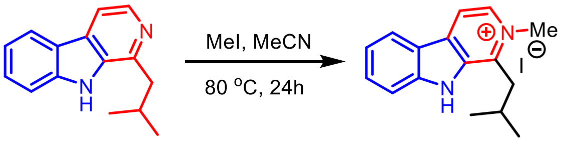

Inspired from a known procedure.[2]
To a solution of pyridine **28a** (224 mg, 1.00 mmol) in CH_3CN (2 mL) was added CH_3I (3.1 mL, 50 mmol) under argon. The mixture was heated at 80 °C for 21 h and then cooled to room temperature. Removal of the solvent under reduced pressure afforded the crude product, which was purified by recrystallization in an CH_3CN /EtOAc cosolvent system to give 340 mg of **29a** as a red solid (93% yield).

R_f = 0.30 (CH_2Cl_2/MeOH 10:1)
^{1}H NMR (360 MHz, MeOD) δ 8.54 – 8.46 (m, 2H), 8.38 (dt, *J* = 8.0, 1.0 Hz, 1H), 7.84 – 7.75 (m, 2H), 7.46 (ddd, *J* = 8.0, 6.5, 1.0 Hz, 1H), 4.48 (s, 3H), 3.43 (d, *J* = 7.7 Hz, 2H), 2.42 – 2.32 (m, 1H), 1.10 (d, *J* = 6.6 Hz, 6H).
^{13}C NMR (90 MHz, MeOD) δ 145.6, 144.3, 137.0, 136.1, 133.7, 133.2, 124.2, 123.1, 121.4, 116.9, 113.8, 45.5, 38.2, 29.7, 22.6 (2C).

HRMS (m/z): $[M]^+$ calcd. for $C_{16}H_{19}N_2^+$ 239.1543, found 239.1537.

Compound 31: 2-(2-(hex-5-enoyl)-1*H*-indol-3-yl)-*N*-methylacetamide

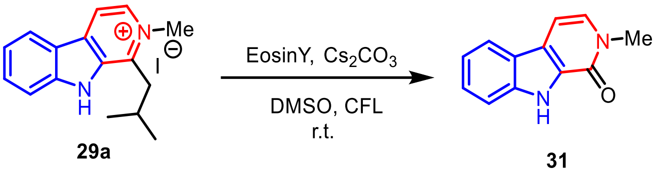

Inspired from a known procedure.[3]

To a solution of pyridinium **29a** (18.3 mg, 0.0500 mmol) in DMSO (1 mL) at room temperature was added EosinY (1 mg) and Cs_2CO_3 (24.0 mg, 0.075 mmol). The reaction mixture was stirred at this temperature under CFL irradiation for about 1 day. The reaction mixture was washed with a saturated aqueous solution of NaCl after which the organic layer was dried over Na_2SO_4, filtered and concentrated concentrated under reduced pressure. The residue was purified by preparative TLC (CH_2Cl_2/MeOH:10/1) to afforded **31** (3 mg) as colorless solid in 10% yield.

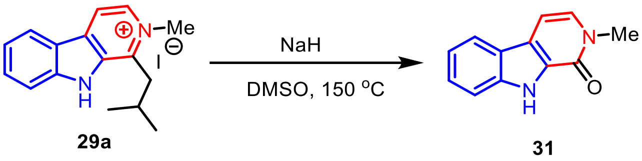

To a solution of pyridinium **29a** (5.0 mg, 0.0137 mmol) in DMSO (0.5 mL) was added NaH (1 mg, 4.0 eq) at room temperature. The reaction mixture was heated to 150 °C and stirred at this temperature overnight. The reaction mixture was cooled down to room temperature and diluted with 2 mL of CH_2Cl_2 and was washed with a saturated aqueous solution of NaCl. The organic layer was dried over Na_2SO_4, filtered and concentrated under reduced pressure. The residue was purified by preparative TLC (CH_2Cl_2/MeOH:10/1) to afford **31** (2 mg) as colorless solid in 74% yield.

R$_f$ = 0.50 (CH_2Cl_2/MeOH 10:1)

^{1}H NMR (360 MHz, MeOD) δ 7.99 (dt, *J* = 8.1, 1.0 Hz, 1H), 7.57 (dt, *J* = 8.1, 1.0 Hz, 1H), 7.47 (ddd, *J* = 8.1, 6.8, 1.0 Hz, 1H), 7.33 (d, *J* = 6.9 Hz, 1H), 7.22 (ddd, *J* = 8.1, 6.8, 1.0 Hz, 1H), 7.11 (d, *J* = 6.9 Hz, 1H), 3.73 (s, 3H).

^{13}C NMR (90 MHz, MeOD) δ 157.6, 141.3, 130.2, 128.1, 126.8, 123.5, 122.2, 121.1, 113.4, 102.8, 37.2.

HRMS (m/z): $[M + H]^+$ calcd. for $C_{12}H_{11}N_2O^+$ 199.0866, found 199.0863

$[M + Na]^+$ calcd. for $C_{12}H_{11}N_2NaO^+$ 221.0685, found 221.0683.

Compound (±)-28: methyl -4-((9*H*-pyrido[3,4-*b*]indol-1-yl)methyl)-2-ethoxy-3-vinyl-3,4-dihydro-2*H*-pyran-5-carboxylate

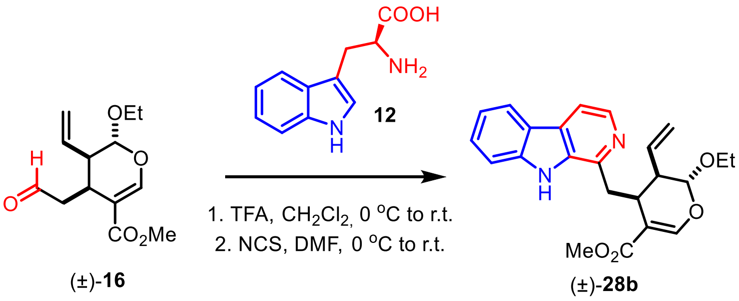

Inspired from a known procedure.[1]

To a solution of *L*-tryptophan **12** (46 mg, 0.228 mmol) in CH_2Cl_2 (3 mL) at room temperature was added (±)-**16** (29 mg, 0.114 mmol), which was prepared according our previous adaptation of Tietze procedures.[4] TFA (65 mg, 0.571 mmol) was then added dropwise at 0 °C under argon. Then, the temperature was allowed to warm up to room temperature and the reaction mixture stirred at this temperature overnight. The reaction mixture was concentrated under reduced pressure to remove TFA and CH_2Cl_2. The mixture was dissolved in 2 mL of DMF, followed by addition of NCS (64 mg, 0.479 mmol) and Et_3N (58 mg, 0.571 mmol) dropwise at 0 °C. Then the mixture was stirred at room temperature for 3 h and quenched with water. The mixture was diluted with EtOAc and washed 3 times with a saturated aqueous solution of NaCl. The organic phase was dried over Na_2SO_4, filtered and concentrated under reduced pressure. The residue was purified by preparative TLC on silica gel (CH_2Cl_2/MeOH 18:1) to give 13 mg of (±)-**28b** as a pale yellow solid (29% yield).

R_f = 0.50 (CH_2Cl_2/MeOH 10:1)
^{1}H NMR (300 MHz, $CDCl_3$) δ 11.04 (s, 1H), 8.27 (d, *J* = 5.4 Hz, 1H), 8.12 (dt, *J* = 8.0, 1.0 Hz, 1H), 7.86 (d, *J* = 5.4 Hz, 1H), 7.66 (dt, *J* = 8.0, 1.0 Hz, 1H), 7.63 (s, 1H), 7.57 (ddd, *J* = 8.0, 7.0, 1.0 Hz, 1H), 7.30 (ddd, *J* = 8.0, 7.0, 1.0 Hz, 1H, 1H), 6.02 (dt, *J* = 17.0, 10.3 Hz, 1H), 5.31 (d, *J* = 8.2 Hz, 1H), 4.85 (dd, *J* = 17.0, 1.0 Hz, 1H), 4.78 (dd, *J* = 10.3, 1.5 Hz, 1H), 3.97 (dd, *J* = 9.7, 7.1 Hz, 1H), 3.89 (s, 3H), 3.71 (dd, *J* = 9.7, 7.1 Hz, 1H), 3.54 – 3.45 (m, 1H), 3.40 – 3.31 (m, 2H), 2.61 – 2.52 (m, 1H), 1.24 (t, *J* = 7.1 Hz, 3H).
^{13}C NMR (75 MHz, $CDCl_3$) δ 169.5, 154.4, 144.3, 140.9, 136.3, 134.8, 129.0, 128.5, 121.8, 121.5, 119.9, 118.4, 113.7, 112.3, 109.3, 100.7, 66.0, 52.1, 46.3, 38.0, 35.0, 15.2.
HRMS (m/z): $[M + H]^+$ calcd. for $C_{23}H_{25}N_2O_4^+$ 393.1809, found 393.1791.

Compound (±)-29b: 2-ethoxy-5-(methoxycarbonyl)-3-vinyl-3,4-dihydro-2*H*-pyran-4-yl)methyl)-2-methyl-9*H*-pyrido[3,4-*b*]indol-2-ium iodide [2]

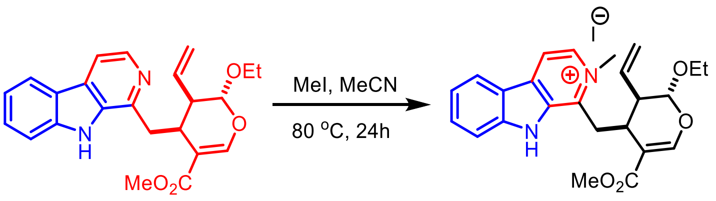

Inspired from a known procedure.[2]
To a solution of (±)-**28b** (12.6 mg, 0.0321 mmol) in CH_3CN (1.5 mL) was added CH_3I (229 mg, 1.607 mmol) under argon. The mixture was heated at 80 °C (heated by oil bath) for 21 h and then cooled to room temperature. Removal of the solvent under reduced pressure afforded the crude product, which was purified by preparative TLC (CH_2Cl_2/MeOH:10/1) to give 11 mg of (±)-**29b** as a red solid (64% yield).

R_f = 0.31 (CH_2Cl_2/MeOH 10:1)
^{1}H NMR (300 MHz, $CDCl_3$) δ 12.24 (s, 1H), 8.24 – 8.20 (m, 2H), 8.13 – 8.06 (m, 2H), 7.71 (t, *J* = 7.8 Hz, 1H), 7.43 (s, 1H), 7.40 (t, *J* = 7.8 Hz, 1H), 6.33 (d, *J* = 9.3 Hz, 1H), 5.90 (ddd, *J* = 17.0, 10.5, 7.8 Hz, 1H), 5.35 (d, *J* = 10.5 Hz, 1H), 5.32 (d, *J* = 17.0 Hz, 1H), 4.25 – 4.05 (m, 5H), 3.93 (t, *J* = 13.0 Hz, 1H), 3.12 – 2.99 (m, 2H), 2.78 (s, 3H), 2.66 – 2.55 (m, 1H), 1.32 (t, *J* = 7.1 Hz, 3H).
^{13}C NMR (75 MHz, $CDCl_3$) δ 167.5, 156.2, 143.9, 141.4, 135.6, 134.4, 134.1, 131.9, 131.1, 123.0, 122.2, 119.7, 119.2, 116.1, 114.2, 105.0, 99.7, 66.4, 50.9, 44.6, 44.3, 35.0, 32.2, 15.6.
HRMS (m/z): $[M]^+$ calcd. for $C_{24}H_{27}N_2O_4^+$ 407.1965, found 407.1965.

Compound 33a: 1-isobutyl-9*H*-pyrido[3,4-*b*]indole 2-oxide

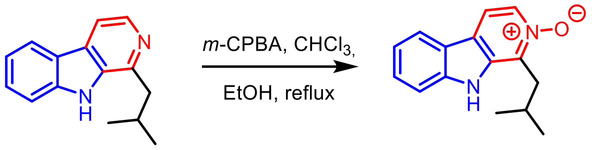

28a **33a**

Inspired from known procedures.[5]

A solution of pyridine **28a** (224 mg, 1.00 mmol) in 10 mL of $CHCl_3$\EtOH (1:1) was treated with *m*-CPBA (865 mg, 5.0 mmol) at room temperature after which the reaction mixture was heated to 80 °C overnight. Then, the reaction mixture was cooled down and a saturated aqueous solution of $NaHCO_3$ was added and stirred at 0 °C for about 10 min. The aqueous layer was extracted 3 times with CH_2Cl_2. The combined organic layers were dried with Na_2SO_4, filtered and concentrated under reduced pressure. The residue was purified by column chromatography on silica gel (Petroleum ether/ EtOAc 1:1 to CH_2Cl_2/MeOH 30:1) to give 166 mg of **33a** as a colorless solid (83% yield).

R_f = 0.66 (CH_2Cl_2/Methanol 10:1)

^{1}H NMR (360 MHz, MeOD) δ 8.11 (d, *J* = 6.7 Hz, 1H), 8.04 (d, *J* = 7.9 Hz, 1H), 7.94 (d, *J* = 6.7 Hz, 1H), 7.50 – 7.47 (m, 2H), 7.24 (t, *J* = 7.9 Hz, 1H), 3.15 (d, *J* = 6.9 Hz, 2H), 2.51-2.43 (m, 1H), 0.99 (d, *J* = 6.9 Hz, 6H).

^{13}C NMR (100 MHz, MeOD) δ 143.7, 138.8, 137.5, 132.0, 129.4, 124.0, 122.3, 121.8, 115.6, 112.9, 36.3, 27.7, 22.8.

HRMS (m/z): $[M + H]^+$ calcd. for $C_{15}H_{17}N_2O^+$ 241.1334, found 241.1330.

Compound (±)-34a: 2-methyl-1-(9*H*-pyrido[3,4-*b*]indol-1-yl)propyl acetate

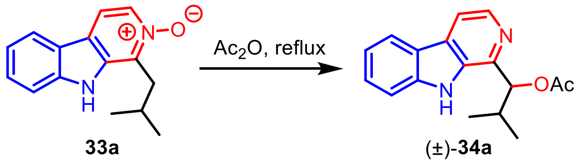

33a (±)-**34a**

Inspired from known procedures.[5]

A solution of pyridine N-oxide **33a** (15 mg, 0.0625 mmol) in acetic anhydride (3.5 mL) was refluxed at 165 °C for 3 h. Then, the reaction mixture was cooled down and a saturated aqueous solution of $NaHCO_3$ was added and the mixture was stirred at 0 °C for about 10 min. The resulting mixture was extracted 3 times with CH_2Cl_2. The combined organic layers were dried over Na_2SO_4, filtered and concentrated under reduced pressure The residue was purified by column chromatography on silica gel (CH_2Cl_2/MeOH 50:1 to 30:1) to give 11 mg of (±)-**34a** as a colorless solid (62% yield).

R_f = 0.32 (CH_2Cl_2/MeOH 30:1)

^{1}H NMR (250 MHz, MeOD) δ 8.24 (d, *J* = 5.3 Hz, 1H), 8.15 (dt, *J* = 8.0, 1.0 Hz, 1H), 8.01 (d, *J* = 5.3 Hz, 1H), 7.62 (dt, *J* = 8.0, 1.0 Hz, 1H), 7.55 (ddd, *J* = 8.0, 7.0, 1.0 Hz, 1H), 7.25 (ddd, *J* = 8.0, 7.0, 1.0 Hz, 1H), 5.90 (d, *J* = 8.6 Hz, 1H), 2.63 – 2.36 (m, 1H), 2.12 (s, 3H), 1.16 (d, *J* = 6.6 Hz, 3H), 0.81 (d, *J* = 6.6 Hz, 3H).

^{13}C NMR (62.5 MHz, MeOD) δ 172.3, 143.8, 142.8, 137.9, 135.1, 131.2, 129.8, 122.5, 122.1, 120.9, 115.4, 112.9, 80.7, 33.5, 20.7, 19.3, 18.8.

HRMS (m/z): $[M + H]^+$ calcd. for $C_{17}H_{19}N_2O_2^+$ 283.1441, found 283.1434.

Compound (±)-35a: 1-(1-acetoxy-2-methylpropyl)-9*H*-pyrido[3,4-*b*]indol-3-yl acetate

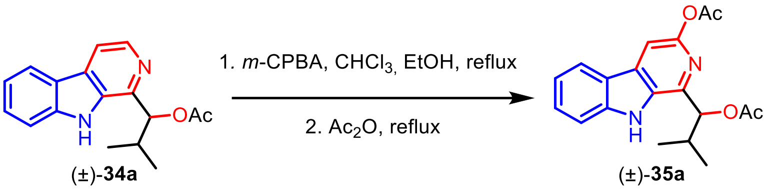

Inspired from known procedures.[5]

A solution of pyridine acetate (±)-**34a** (150 mg, 0.531 mmol) in $CHCl_3$\EtOH (1:1) 15 mL was treated with *m*-CPBA (450 mg, 2.65 mmol) at room temperature after which the reaction mixture was heated to 80 °C for about 18 h. Then, the reaction mixture was cooled down and a saturated aqueous solution of $NaHCO_3$ was added and the mixture was stirred at 0 °C for about 10 min. The resulting mixture was extracted 3 times with CH_2Cl_2. The combined organic layers were dried over Na_2SO_4, filtered and concentrated under reduced pressure. The residue was purified by column chromatography on silica gel (CH_2Cl_2/MeOH 50:1 to 30:1) to give 124 mg of 1-(1-acetoxy-2-methylpropyl)-9*H*-pyrido[3,4-*b*]indole 2-oxide as a colorless solid (78% yield).

A solution this pyridine N-oxide (120 mg, 0.402 mmol) in acetic anhydride (18 mL) was reflux at 165 °C for 3 h. Then the reaction mixture was cooled down and $NaHCO_3$ (aq.) was added and stirred at 0 °C for about 10 min. The resulting mixture was extracted 3 times with CH_2Cl_2. The combined organic layers were dried over Na_2SO_4, filtered and concentrated under reduced pressure. The residue was purified by column chromatography on silica gel (CH_2Cl_2/MeOH 50:1 to 30:1) to give 32 mg of (±)-**35a** as a colorless solid (23% yield).

***R**f* = 0.38 (CH_2Cl_2/MeOH 30:1)

^{1}H NMR (360 MHz, MeOD) δ 8.15 (dt, *J* = 8.0, 1.0 Hz, 1H), 7.73 (s, 1H), 7.65 – 7.54 (m, 2H), 7.25 (ddd, *J* = 8.0, 6.7, 1.0 Hz, 1H), 5.84 (d, *J* = 8.7 Hz, 1H), 2.54 – 2.44 (m, 1H), 2.36 (s, 3H), 2.13 (s, 3H), 1.15 (d, *J* = 6.8 Hz, 3H), 0.83 (d, *J* = 6.8 Hz, 3H).

^{13}C NMR (100 MHz, MeOD) δ 172.3, 171.7, 150.4, 144.0, 141.4, 134.6, 134.1, 130.3, 122.8, 122.1, 120.9, 113.1, 107.1, 80.3, 33.5, 21.0, 20.7, 19.3, 18.8.

HRMS (m/z): $[M + H]^+$ calcd. for $C_{19}H_{21}N_2O_2^+$ 341.1496, found 341.1483.

$[M + Na]^+$ calcd. for $C_{19}H_{20}N_2NaO_2^+$ 363.1315, found 363.1304.

Compound (±)-36a: 1-(1-hydroxy-2-methylpropyl)-2-methyl-2,9-dihydro-3*H*-pyrido[3,4-*b*]indol-3-one

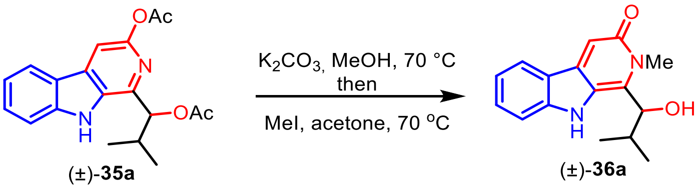

Inspired from a known procedure.[5b]

To a solution of acetoxypyridine (±)-**35a** (13 mg, 0.0382 mmol) in MeOH (3 mL) was added K_2CO_3 (131.3 mg, 0.95 mmol) at room temperature. Then the mixture was stirred at 70 °C for 2 h. Then, MeI (5 ml) and acetone (24 mL) were added into the mixture which was stirred at 70 °C overnight. The reaction mixture was then concentrated directly under reduced pressure, and was then purified by preparative TLC on silica gel (CH_2Cl_2) to give 3 mg of (±)-**36a** as a yellow solid (29% yield).

***R**f* = 0.12 (CH_2Cl_2)

^{1}H NMR (360 MHz, MeOD) δ 8.08 (dt, *J* = 8.0, 1.5 Hz, 1H), 7.53 – 7.44 (m, 2H), 7.32 (s, 1H), 7.15 (ddd, *J* = 8.0, 6.5, 1.5 Hz, 1H), 4.78 (d, *J* = 6.1 Hz, 1H), 3.98 (s, 3H), 2.41 – 2.24 (m, 1H), 1.01 (d, *J* = 6.8 Hz, 3H), 0.94 (d, *J* = 6.8 Hz, 3H).
^{13}C NMR (100 MHz, MeOD) δ 158.4, 144.1, 143.9, 135.2, 131.4, 129.6, 122.6, 122.2, 119.8, 112.7, 96.9, 79.1, 54.8, 35.5, 19.6, 18.0.
HRMS (m/z): $[M + H]^+$ calcd. for $C_{16}H_{19}N_2O_2^+$ 271.1441, found 271.1428.

Compound 37a: 1-isobutyl-9*H*-pyrido[3,4-*b*]indol-3-yl trifluoromethanesulfonate

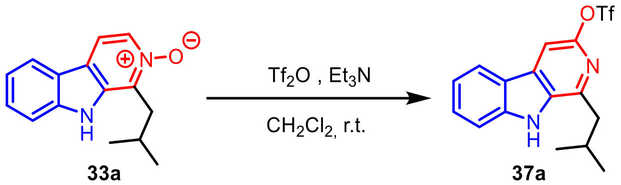

33a 37a

To a solution of pyridine N-oxide **33a** (24 mg, 0.100 mmol) in CH_2Cl_2 (1 mL), was added Tf_2O (28.2 mg, 0.5 mmol) and Et_3N (102 mg, 1 mmol) at room temperature. The reaction mixture was stirred at room temperature for about 30 min and was then quenched with water after which it was extracted 3 times with CH_2Cl_2. The combined organic layers were dried over Na_2SO_4, filtered and concentrated under reduced pressure. The residue was purified by column chromatography on silica gel (CH_2Cl_2) to give 28 mg of **37a** as a colorless oil (74% yield).

R$_f$ = 0.66 (CH_2Cl_2)
^{1}H NMR (400 MHz, $CDCl_3$) δ 8.22 (br, 1H), 8.09 (dt, *J* = 8.0, 1.0 Hz, 1H), 7.63 (s, 1H), 7.60 (ddd, *J* = 8.0, 6.5, 1.0 Hz, 1H), 7.54 (dt, *J* = 8.0, 1.0 Hz, 1H), 7.32 (ddd, *J* = 8.0, 6.5, 1.0 Hz, 1H), 2.90 (d, *J* = 7.3 Hz, 2H), 2.42 – 2.31 (m, 1H), 1.01 (d, *J* = 6.6 Hz, 6H).
^{13}C NMR (100 MHz, $CDCl_3$) δ 148.1, 143.6, 141.4, 134.6, 132.3, 129.6, 122.1, 121.6, 120.8, 119.0 (q, *J* = 320.0 Hz, CF_3), 112.0, 103.6, 42.5, 28.2, 22.7 (2C).
^{19}F NMR (235 MHz, $CDCl_3$) δ -72.79.
HRMS (m/z): $[M + H]^+$ calcd. for $C_{16}H_{16}F_3N_2O_3S^+$ 373.0828, found 373.0821.
$[M + Na]^+$ calcd. for $C_{16}H_{15}F_3N_2NaO_3S^+$ 395.0648, found 395.0641.

Compound 38a: 1-isobutyl-2-methyl-3-(((trifluoromethyl)sulfonyl)oxy)-9*H*-pyrido[3,4-*b*]indol-2-ium iodide

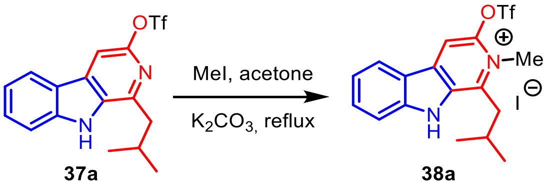

37a 38a

To a solution of triflyloxypyridine **37a** (15 mg, 0.0403 mmol) in acetone (5 mL) was added MeI (25 mL) and K_2CO_3 (12 mg, 0.088 mmol) at room temperature. Then, the mixture was stirred at 60 °C for 48 h. The reaction mixture was then directly concentrated under reduced pressure, and was purified by preparative TLC on silica gel (CH_2Cl_2/petroleum ether 1:1) to give 6 mg of **38a** as a solid (30% yield).

R$_f$ = 0.85 (CH_2Cl_2)
^{1}H NMR (300 MHz, $CDCl_3$) δ 8.10 (dt, *J* = 8.0, 0.9 Hz, 1H), 7.65 (s, 1H), 7.64 (ddd, *J* = 8.0, 7.1, 0.9 Hz, 1H), 7.47 (dt, *J* = 8.0, 0.9 Hz, 1H), 7.31 (ddd, *J* = 8.0, 7.1, 0.9 Hz, 1H), 4.11 (s, 3H), 3.18 (d, *J* = 6.8 Hz, 2H), 2.36-2.22 (m, 1H), 1.03 (d, *J* = 6.8 Hz, 6H).

^{13}C NMR (62.5 MHz, $CDCl_3$) δ 147.2, 143.8 (2C), 135.7, 133.4, 129.5, 121.9, 121.0, 120.4, 119.1 (q, *J* = 318.8 Hz, CF_3), 110.0, 103.4, 43.9, 32.6, 29.3, 22.5 (2C).
^{19}F NMR (235 MHz, $CDCl_3$) δ -72.84.
HRMS (m/z): $[M + H]^+$ calcd. for $C_{17}H_{18}F_3N_2O_3S^+$ 387.0985, found 387.0969.

Compound 39a: 1-isobutyl-2-methyl-2,9-dihydro-3*H*-pyrido[3,4-*b*]indol-3-one

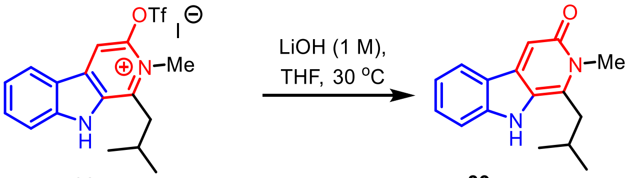

Inspired from a known procedure.[6]
A 1 M aqueous solution of LiOH (0.022 mL, 0.022 mmol,) was added to a solution of triflyloxypyridinium **38a** (5 mg, 0.00973 mmol,) in THF (0.5 mL). The mixture was stirred at 30 °C for about 20 h and the mixture was cooled to room temperature, followed by addition of water and careful acidification with a 1 M aqueous solution of HCl to pH 1–2. The resulting mixture was extracted 5 times with CH_2Cl_2 and the combined organic extracts were dried over $MgSO_4$. Removal of solvent under reduced pressure and purification by preparative TLC on silica gel (CH_2Cl_2/ MeOH 20:1) gave 1.5 mg of **39a** as a fluorescent yellow solid (60% yield).

$\boldsymbol{R_f}$ = 0.14 (CH_2Cl_2/ MeOH 20:1)
^{1}H NMR (400 MHz, $CDCl_3$) δ 7.93 (dt, *J* = 8.2, 1.2 Hz, 1H), 7.53 (ddd, *J* = 8.2, 7.3, 1.2 Hz, 1H), 7.20 (dt, *J* = 8.2, 1.2 Hz, 1H), 7.12 (ddd, *J* = 8.2, 7.3, 1.2 Hz, 1H), 7.01 (s, 1H), 3.80 (s, 3H), 3.04 (d, *J* = 7.4 Hz, 2H), 2.21 – 2.09 (m, 1H), 1.04 (d, *J* = 6.6 Hz, 6H).
^{13}C NMR (100 MHz, $CDCl_3$) δ 162.3, 147.1, 140.8, 131.6, 130.5, 126.7, 122.8, 120.9, 119.1, 108.9, 103.0, 39.1, 32.4, 30.5, 22.3 (2C).
HRMS (m/z): $[M + H]^+$ calcd. for $C_{16}H_{19}N_2O^+$ 255.1492, found 255.1486.
$[M + Na]^+$ calcd. for $C_{16}H_{18}N_2NaO^+$ 277.1311, found 277.1307.

Compound (±)-33b: 2-ethoxy-5-(methoxycarbonyl)-3-vinyl-3,4-dihydro-2*H*-pyran-4-yl)methyl)-9*H*-pyrido[3,4-*b*]indole 2-oxide

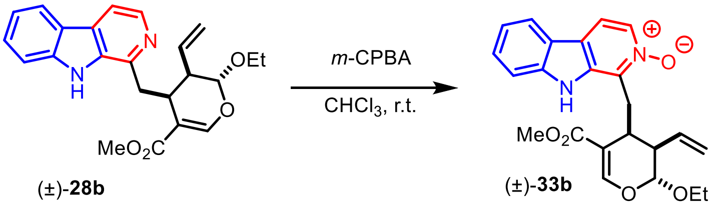

Inspired from known procedures.[5]
To solution of pyridine (±)-**28b** (12 mg, 0.0306 mmol) in $CHCl_3$ (2 mL) was added *m*-CPBA (25.8 mg, 0.15 mmol) at room temperature and the reaction was stirred at this temperature for 1 h. Then the reaction mixture was quenched with a saturated aqueous solution of $NaHCO_3$ and the mixture was stirred at 0 °C for about 10 min. The resulting mixture was extracted 3 times with CH_2Cl_2. The combined organic layers were dried with Na_2SO_4, filtered and concentrated under reduced pressure. The residue was purified by column chromatography on silica gel (CH_2Cl_2 to EtOAc to CH_2Cl_2/MeOH 10:1) to give 5 mg of (±)-**33b** as a colorless solid (40% yield).

$\boldsymbol{R_f}$ = 0.40 (CH_2Cl_2/MeOH 10:1)

^{1}H NMR (300 MHz, $CDCl_3$) δ 11.31 (s, 1H), 8.26 (d, *J* = 6.6 Hz, 1H), 8.01 (d, *J* = 7.8 Hz, 1H), 7.76 (d, *J* = 6.6 Hz, 1H), 7.66 (s, 1H), 7.65 (d, *J* = 7.8 Hz, 1H), 7.53 (t, *J* = 7.8 Hz, 1H), 7.30 (t, *J* = 7.8 Hz, 1H), 6.28 (dt, *J* = 17.0, 10.0 Hz, 1H), 5.35 (d, *J* = 8.8 Hz, 1H), 4.83 (d, *J* = 17.0 Hz 1H), 4.78 (d, *J* = 10.0 Hz, 1H), 4.16 (dd, *J* = 14.9, 10.2 Hz, 1H), 4.05 – 3.94 (m, 1H), 3.89 (s, 3H), 3.80 – 3.71 (m, 1H), 3.26 (dd, *J* = 10.1, 5.6 Hz, 1H), 3.02 (d, *J* = 14.9 Hz, 1H), 2.60 – 2.46 (m, 1H), 1.25 (t, *J* = 7.1 Hz, 3H).
^{13}C NMR (90 MHz, $CDCl_3$) δ 169.6, 155.0, 141.6, 136.3, 136.0, 133.3, 131.4, 127.6, 121.5, 120.9, 120.6, 120.5, 118.5, 114.3, 112.2, 108.6, 100.8, 66.1, 52.2, 45.8, 35.5, 27.7, 15.2.
HRMS (m/z): $[M + H]^+$ calcd. for $C_{23}H_{25}N_2O_5^+$ 409.1758, found 409.1749.

Compound (±)-37b: methyl -2-ethoxy-4-((3-(((trifluoromethyl)sulfonyl)oxy)-9*H*-pyrido[3,4-*b*]indol-1-yl)methyl)-3-vinyl-3,4-dihydro-2*H*-pyran-5-carboxylate

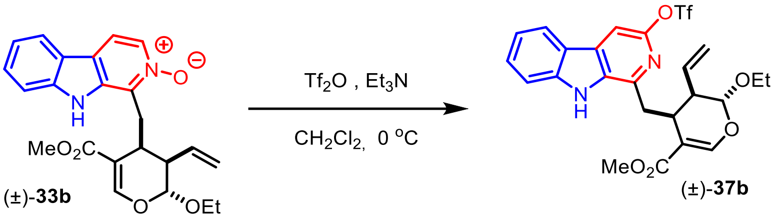

To a solution of pyridine N-oxide (±)-**33b** (10 mg, 0.0245 mmol) in CH_2Cl_2 (1 mL) was added Tf_2O (35 mg, 0.125 mmol) and Et_3N (25 mg, 0.25 mmol) at 0°C. Then the reaction mixture was stirred at 0 °C for about 5 min and was quenched with water. The aqueous layer was extracted with CH_2Cl_2 for 3 times. The combined organic layers were dried with Na_2SO_4, filtered and concentrated in vacuo. The residue was purified by column chromatography on silica gel (Petroleum ether/ EtOAc 10:1) to give 1 mg of (±)-**37b** as a colorless oil (7% yield).

R_f = 0.16 (Petroleum ether/ EtOAc 10:1)
^{1}H NMR (300 MHz, $CDCl_3$) δ 11.14 (s, 1H), 8.09 (dt, *J* = 7.9, 1.0 Hz, 1H), 7.70 – 7.55 (m, 3H), 7.59 (ddd, *J* = 7.9, 7.0, 1.0 Hz, 1H), 7.30 (ddd, *J* = 7.9, 7.0, 1.0 Hz, 1H), 5.90 (dt, *J* = 16.9, 9.6 Hz, 1H), 5.17 (d, *J* = 7.4 Hz, 1H), 4.97 (d, *J* = 16.9 Hz, 1H), 4.95 (dd, *J* = 9.6, 1.0 Hz, 1H), 4.00 – 3.91 (m, 1H), 3.90 (s, 3H), 3.76 – 3.65 (m, 1H), 3.50 (d, *J* = 14.2 Hz, 1H), 3.26 (dd, *J* = 10.2, 5.4 Hz, 1H), 3.12 (dd, *J* = 14.2, 10.2 Hz, 1H), 2.64 – 2.54 (m, 1H), 1.24 (d, *J* = 7.1 Hz, 3H).
^{13}C NMR (100 MHz, $CDCl_3$) δ 169.6, 154.5, 147.2, 143.0, 142.0, 134.9, 134.2, 132.1, 129.3, 126.9 (q, *J* = 310.0 Hz, CF_3), 122.1, 121.4, 120.3, 119.5, 112.6, 109.1, 104.1, 100.6, 65.6, 52.2, 46.0, 37.3, 34.4, 15.1.
^{19}F NMR (235 MHz, $CDCl_3$) δ -73.26.
HRMS (m/z): $[M + H]^+$ calcd. for $C_{24}H_{24}F_3N_2O_7S^+$ 541.1251, found 541.1235.
$[M + Na]^+$ calcd. for $C_{24}H_{23}F_3N_2NaO_7S^+$ 563.1070, found 563.1057.

Compound 15b: methyl 2-(1*H*-indol-3-yl)acetate[7]

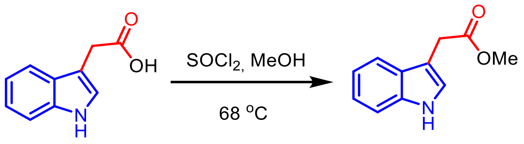

15c 15b

Prepared according to a known procedure.[7]

To a solution of indole-3-acetic acid **15c** (2.10 g, 12.0 mmol) in methanol (24 mL) under argon at 0 °C was slowly added $SOCl_2$ (4.28 g, 36 mmol). The resulting mixture was heated to 68 °C over 12 h. The reaction was poured into a saturated aqueous solution of $NaHCO_3$ (40 mL). The resulting mixture was extracted three times with EtOAc (3 × 50 mL) and the combined organic layers were dried over Na_2SO_4, filtered and concentrated under reduced pressure. The residue was purified by column chromatography on silica gel (Petroleum ether/ EtOAc 3:2) to give 2.064 g of **15b** as a colorless solid (91% yield).

R$_f$ = 0.59 (Petroleum ether/ EtOAc 3:2)
^{1}H NMR (360 MHz, $CDCl_3$) δ 8.12 (br, 1H), 7.63 (dt, *J* = 8.1, 1.0 Hz, 1H), 7.35 (dt, *J* = 8.1, 1.0 Hz, 1H), 7.21 (ddd, *J* = 8.1, 7.0, 1.0 Hz, 1H), 7.15 (ddd, *J* = 8.1, 7.0, 1.0 Hz, 1H), 7.13-7.11 (m, 1H), 3.80 (d, *J* = 0.9 Hz, 2H), 3.72 (s, 3H).
^{13}C NMR (90 MHz, $CDCl_3$) δ 172.7, 136.2, 127.3, 123.2, 122.3, 119.8, 119.0, 111.3, 108.5, 52.1, 31.3.
HRMS (m/z): $[M + H]^+$ calcd. for $C_{11}H_{12}NO_2^+$ 190.0863, found 190.0861.
$[M + Na]^+$ calcd. for $C_{11}H_{11}NNaO_2^+$ 212.0682, found 212.0680.

Compound 15a: 2-(1*H*-indol-3-yl)-*N*-methylacetamide[8]

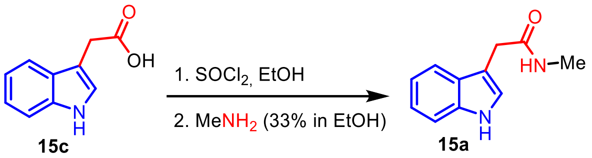

Prepared according to a known procedure.[8]
To a solution of indole-3-acetic acid **15c** (2.16 g, 12.33 mmol) in EtOH (24 mL) was added dropwise $SOCl_2$ (4.28 g, 36 mmol). The reaction was stirred to reflux for overnight. Upon completion of the reaction (monitoring by TLC), the mixture was concentrated to dryness and the crude material was directly treated for next step without isolation. The crude reaction mixture was dissolved in a solution of $MeNH_2$ (25 mL of 33% in EtOH). The mixture was stirred at 25 ºC for 24 h until ethyl 3-indoleacetate was fully consumed and was concentrated under reduced pressure. The crude product was purified by flash chromatography on silica gel (Petroleum ether/EtOAc 1:3) to give 2.264 g of **15a** as a colorless solid (98% yield).

R$_f$ = 0.13 (Petroleum ether/EtOAc 1:3)
^{1}H NMR (360 MHz, $CDCl_3$) δ 9.15 (br, 1H), 7.54 (dt, *J* = 8.0, 1.1 Hz, 1H), 7.41 (dt, *J* = 8.0, 1.1 Hz, 1H), 7.23 (ddd, *J* = 8.0, 7.0, 1.1 Hz, 1H), 7.14 (ddd, *J* = 8.0, 7.0, 1.1 Hz, 1H), 7.10 (d, *J* = 2.4 Hz, 1H), 5.89 (br, 1H), 3.74 (s, 2H), 2.71 (d, *J* = 4.8 Hz, 3H).
^{13}C NMR (90 MHz, $CDCl_3$) δ 172.8, 136.6, 127.1, 124.3, 122.5, 119.9, 118.6, 111.7, 108.5, 33.3, 26.5.
HRMS (m/z): $[M + H]^+$ calcd. for $C_{11}H_{13}N_2O^+$ 189.1022, found 189.1019.
$[M + Na]^+$ calcd. for $C_{11}H_{12}N_2NaO^+$ 211.0842, found 211.0840.

Compound (±)-40: 2,2'-((3-methylbutane-1,1-diyl)bis(1*H*-indole-2,3-diyl))bis(*N*-methylacetamide)

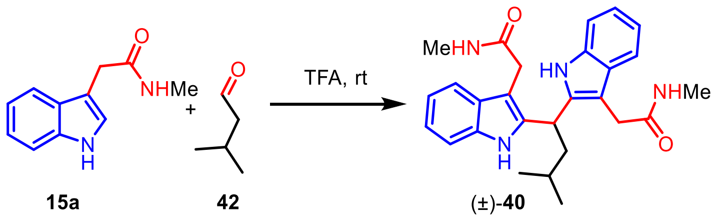

Inspired from a known procedure.[9]

2-(1*H*-indol-3-yl)-*N*-methylacetamide **15a** (18.8 mg, 0.100 mmol) and 3-methylbutanal **42** (9 mg, 0.1 mmol) were dissolved in TFA (1 mL) and the reaction was stirred for 40 min at room temperature. The solution was diluted with EtOAc and washed with a saturated aqueous solution of $NaHCO_3$. The organic layer was dried over Na_2SO_4, filtered and concentrated under reduced pressure. The residue was purified by preparative TCL on silica gel (CH_2Cl_2/MeOH 20:1) to give 6 mg of (±)-**40** as a colorless solid (27% yield).

R*f* = 0.22 (CH_2Cl_2/MeOH 20:1)
^{1}H NMR (300 MHz, $CDCl_3$) δ 10.22 (s, 2H), 7.45 (d, *J* = 8.0 Hz, 2H), 7.32 (d, *J* = 8.0 Hz, 2H), 7.17 – 7.02 (m, 4H), 5.91 (q, *J* = 4.8 Hz, 2H), 4.73 (t, *J* = 7.5 Hz, 1H), 3.84 (d, *J* = 16.0 Hz, 2H), 3.76 (d, *J* = 16.0 Hz, 2H), 2.66 (d, *J* = 4.8 Hz, 6H), 2.23 (t, *J* = 7.5 Hz, 2H), 1.60 – 1.47 (m, 1H), 0.96 (d, *J* = 6.6 Hz, 6H).
^{13}C NMR (100 MHz, $CDCl_3$) δ 173.3 (2C), 138.2 (2C), 135.8 (2C), 128.3 (2C), 121.9 (2C), 119.6 (2C), 117.5 (2C), 111.5 (2C), 103.9 (2C), 41.7, 34.9, 32.6 (2C), 26.7 (2C), 26.2, 22.8 (2C).
HRMS (m/z): $[M + H]^+$ calcd. for $C_{27}H_{33}N_4O_2^+$ 445.2598, found 445.2581.
$[M + Na]^+$ calcd. for $C_{27}H_{32}N_4NaO_2^+$ 467.2417, found 467.2402.

Compound 43a: 1-propyl-2,9-dihydro-3H-pyrido[3,4-b]indol-3-one

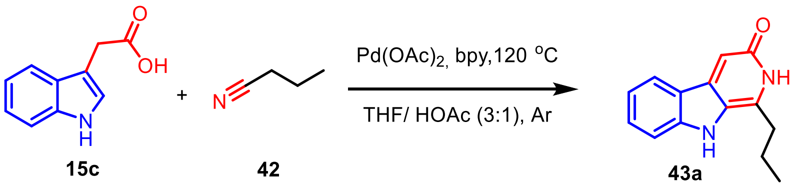

Inspired from a known procedure.[10]

2-(1*H*-indol-3-yl)acetic acid **15c** (525 mg, 3.00 mmol), butyronitrile (52 µL, 0.6 mmol), $Pd(OAc)_2$ (13.4 mg, 0.06 mmol), 2,2'-bipyridine (11.2 mg, 0.072 mmol) and HOAc/THF (v/v = 1/3, 1.6 mL) were placed in a sealed tube under argon atmosphere. The mixture was stirred at 120 °C overweekend. Then the mixture was cooled to room temperature, and a saturated aqueous solution of $NaHCO_3$ was added until no bubbles were generated. The resulting mixture was extracted five times with CH_2Cl_2. The combined organic layers were then dried over Na_2SO_4, filtered and concentrated under reduced pressure. The residue was purified by preparative TLC on silica gel (CH_2Cl_2/Methanol 10:1) to give 61 mg of **43a** as a colorless solid (45% yield).

R*f* = 0.38 (CH_2Cl_2/Methanol 10:1)
^{1}H NMR (300 MHz, MeOD) δ 7.97 (dt, *J* = 8.0, 1.0 Hz, 1H), 7.50 (ddd, *J* = 8.0, 7.1, 1.0 Hz, 1H), 7.35 (dt, *J* = 8.0, 1.0 Hz, 1H), 7.10 (ddd, *J* = 8.0, 7.1, 1.0 Hz, 1H), 6.93 (s, 1H), 2.93 (dd, *J* = 8.6, 6.7 Hz, 2H), 1.92 – 1.74 (m, 2H), 1.03 (t, *J* = 7.3 Hz, 3H).
^{13}C NMR (100 MHz, MeOD) δ 162.9, 147.1, 141.6, 133.0, 131.7, 127.3, 123.8, 121.7, 120.2, 112.4, 102.2, 32.3, 23.4, 13.9.
HRMS (m/z): $[M + H]^+$ calcd. for $C_{14}H_{15}N_2O_2^+$ 227.1179, found 227.1184.

Compound 26: *O*-(4-(trifluoromethyl)benzoyl)hydroxylamine[11]

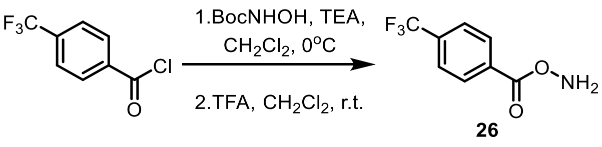

Prepared according to a known procedure.[11]

To a solution of *tert*-butyl *N*-hydroxycarbamate (402 mg, 3.02 mmol), Et_3N (420 µL, 3.02 mmol) in CH_2Cl_2 (5 mL) was added 4-trifluoromethylbenzoyl chloride (314 mg, 1.51 mmol) dropwise at 0 °C for 30 min and stirred at 0 °C for another 10 min. Then, the mixture was poured into a saturated aqueous solution of $NaHCO_3$. The resulting mixture was extracted twice with CH_2Cl_2. The combined organic phases were then dried over Na_2SO_4, filtered and concentrated under reduced pressure. The residue was then dissolved in dry CH_2Cl_2 (1 mL). Trifluoroacetic acid (2 ml) was then added dropwise at room temperature and the reaction mixture was stirred at 0 °C for another 30 min. Then, the mixture was poured into a saturated aqueous solution of $NaHCO_3$ and the resulting mixture was extracted twice with 25 mL of CH_2Cl_2. The combined organic layers were then dried over Na_2SO_4 and concentrated under reduced pressure. The residue was purified by column chromatography on silica gel (Petroleum ether/ EtOAc 5:1) to give 132 mg of **26** as a white solid (42% yield).

R_f = 0.31 (Petroleum ether/ Ethyl acetate 3:1)
^{1}H NMR (300 MHz, $CDCl_3$) δ 8.13 (d, *J* = 8.1 Hz, 2H), 7.72 (d, *J* = 8.1 Hz, 2H), 6.38 (s, 2H).
^{13}C NMR (90 MHz, $CDCl_3$) δ 166.4, 135.10 (q, *J* = 32.4 Hz), 131.3, 130.0, 125.8 (q, *J* = 3.6 Hz), 123.6 (q, *J* = 270.9 Hz)
^{19}F NMR (235 MHz, $CDCl_3$) δ -63.25.

Compound (±)-25: methyl -4-(cyanomethyl)-2-ethoxy-3-vinyl-3,4-dihydro-2*H*-pyran-5-carboxylate

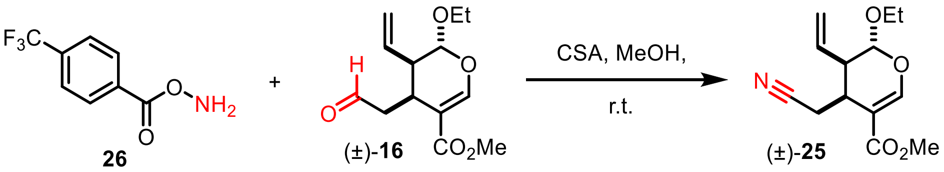

Inspired from a known procedure.[12]

A 3 mL vial was equipped with a magnetic stir bar and was charged with (±)-**16** (40 mg, 0.157 mmol), *O*-(4-(trifluoromethyl)benzoyl)hydroxylamine **26** (40 mg, 0.20 mmol, 1.2 equiv), and CSA (4 mg, 0.016 mmol, 0.1 equiv) in MeOH (2.0 mL). The mixture was then stirred at room temperature overnight, and was then concentrated under reduced pressure to remove MeOH. The residue was purified by preparative TCL on silica gel (Petroleum ether/ EtOAc 5:1) to give 20 mg of (±)-**25** as a white oil (50% yield).

R_f = 0.43 (Petroleum ether/ EtOAc 2:1)
^{1}H NMR (300 MHz, $CDCl_3$) δ 7.54 (d, *J* = 1.6 Hz, 1H), 5.61 (ddd, *J* = 17.0, 10.5, 8.5 Hz, 1H), 5.41 (dd, *J* = 17.0, 1.0 Hz, 1H), 5.34 (d, *J* = 10.5, 1.7 Hz, 1H), 5.13 (d, *J* = 4.5 Hz, 1H), 3.89 – 3.82 (m, 1H), 3.71 (s, 3H), 3.69 – 3.60 (m, 1H), 3.17 (dd, *J* = 16.6, 3.9 Hz, 1H), 3.12 – 3.04 (m, 1H), 2.82 – 2.75 (m, 1H), 2.42 (dd, *J* = 16.6, 9.4 Hz, 1H), 1.22 (t, *J* = 7.1 Hz, 3H).
^{13}C NMR (100 MHz, $CDCl_3$) δ 167.0, 154.1, 132.0, 121.6, 119.0, 106.0, 100.2, 65.2, 51.5, 44.5, 29.0, 18.0, 15.1.
HRMS (m/z): $[M + H]^+$ calcd. for $C_{13}H_{18}NO_4^+$ 252.1230, found 252.1226.
$[M + Na]^+$ calcd. for $C_{13}H_{17}NNaO_4^+$ 274.1050, found 274.1042.

Compound 46b: methyl-2-(2-(hex-5-enoyl)-1*H*-indol-3-yl)acetate

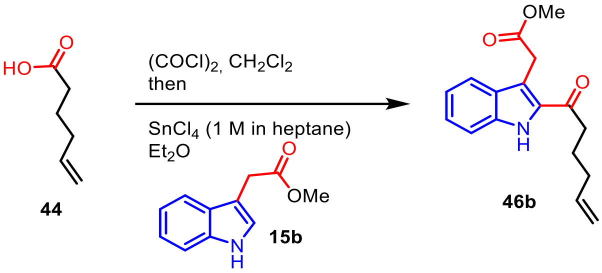

Inspired from known procedures.[13]

To a solution of hex-5-enoic acid **44** (13.7 mg, 0.120 mmol) in CH_2Cl_2 (0.5 mL) at 0 °C, was slowly added oxalyl chloride (19.8 mg, 0.156 mol). The resulting mixture was allowed to warm up to room temperature, while stirring overnight. The reaction was concentrated under reduced pressure and used for next step without any purification. Then the reaction mixture was dissolved in 1 mL of Et_2O, methyl 2-(1*H*-indol-3-yl)acetate **15b** (18.9 mg, 0.1 mmol) was added and then cooled to 0 °C. A solution of tin chloride (0.012 mL of 1 M in heptane) was added dropwise at 0 °C. The resulting mixture was allowed to warm up to room temperature and stirred at room temperature for overnight. The reaction mixture was quenched with a saturated aqueous KF solution and the resulting mixture was extracted 5 times with CH_2Cl_2. The combined organic layers were dried with Na_2SO_4 and concentrated under reduced pressure. The crude product was purified by preparative TLC on silica gel (Petroleum ether/EtOAc 7:3) to give 23 mg of **46b** as a white solid (80% yield).

R*f* = 0.60 (Petroleum ether/Ethyl acetate 7:3)
^{1}H NMR (360 MHz, $CDCl_3$) δ 9.29 (br, 1H), 7.67 (dt, *J* = 8.0, 1.0 Hz, 1H), 7.34 – 7.31 (m, 2H), 7.18-7.13 (m, 1H), 5.81 (ddt, *J* = 16.9, 10.2, 7.0 Hz, 1H), 5.09 – 4.96 (m, 2H), 4.15 (s, 2H), 3.74 (s, 3H), 2.80 (t, *J* = 7.0 Hz, 2H), 2.13 (q, *J* = 7.0 Hz, 2H), 1.78 (pent, *J* = 7.0 Hz, 2H).
^{13}C NMR (90 MHz, $CDCl_3$) δ 193.0, 171.8, 138.1, 135.9, 132.6, 128.3, 126.4, 120.9, 120.8, 115.4, 113.9, 112.5, 52.4, 39.4, 33.2, 31.3, 22.8.
HRMS (m/z): $[M + H]^+$ calcd. for $C_{17}H_{20}NO_3^+$ 286.1438, found 286.1428.
$[M + Na]^+$ calcd. for $C_{17}H_{19}NNaO_3^+$ 308.1257, found 308.1246.

Compound 46a: 2-(2-(hex-5-enoyl)-1*H*-indol-3-yl)-*N*-methylacetamide

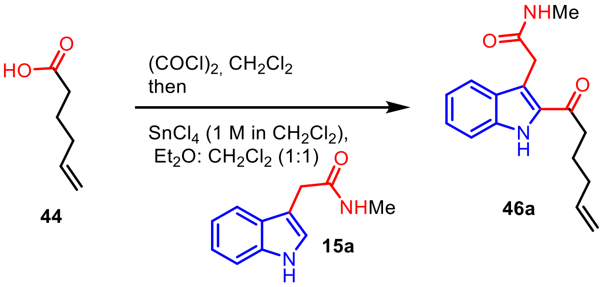

Inspired from known procedures.[13]

To a solution of hex-5-enoic acid **44** (11.4 mg, 0.100 mmol) in CH_2Cl_2 (0.5 mL) at 0 °C, oxalyl chloride (16.5 mg, 0.13 mmol) was added slowly and the resulting mixture was allowed to warm up to room temperature, while stirring overnight. The reaction mixture was concentrated under reduced pressure and used for next step without any purification. To the residue dissolved in 0.5 mL of Et_2O and 0.5 mL of CH_2Cl_2, **15a** (23 mg, 0.12 mmol) was added and the mixture was cooled to 0 °C. A solution of tin chloride (0.1 mL of 1 M in CH_2Cl_2) was added dropwise at 0 °C. The resulting mixture was allowed to warm up to room temperature and stirred at room temperature for 4 h. The reaction mixture was

quenched with a saturated aqueous solution of KF and the resulting mixture was extracted 5 times with CH_2Cl_2. The combined organic layers were dried over Na_2SO_4, filtered and concentrated under reduced pressure. The crude products were purified by preparative TLC on silica gel (CH_2Cl_2/MeOH 15:1) to give 15 mg of **46a** as a white solid (52% yield).

R*f* = 0.53 (CH_2Cl_2/Methanol 10:1)
^{1}H NMR (360 MHz, MeOD) δ 7.67 (d, *J* = 7.7 Hz, 1H), 7.44 (d, *J* = 7.7 Hz, 1H), 7.30 (t, *J* = 7.7 Hz, 1H), 7.10 (t, *J* = 7.7 Hz, 1H), 5.93 – 5.78 (m, 1H), 5.04 (d, *J* = 17.0 Hz, 1H), 4.98 (d, *J* = 10.0 Hz, 1H), 4.03 (s, 2H), 2.98 (t, *J* = 7.2 Hz, 2H), 2.68 (s, 3H), 2.21 – 2.10 (m, 2H), 1.88 – 1.75 (m, 2H).
^{13}C NMR (90 MHz, MeOD) δ 195.9, 174.4, 139.3, 138.1, 133.6, 129.2, 127.0, 121.6, 121.5, 116.7, 115.6, 113.5, 40.3, 34.3, 33.6, 26.5, 24.4.
HRMS (m/z): $[M + H]^+$ calcd. for $C_{17}H_{21}N_2O_2^+$ 285.1598, found 285.1589.
$[M + Na]^+$ calcd. for $C_{17}H_{20}N_2NaO_2^+$ 307.1417, found 307.1408.

Compound 47: 2-methyl-1-(pent-4-en-1-yl)-2,9-dihydro-3*H*-pyrido[3,4-b]indol-3-one

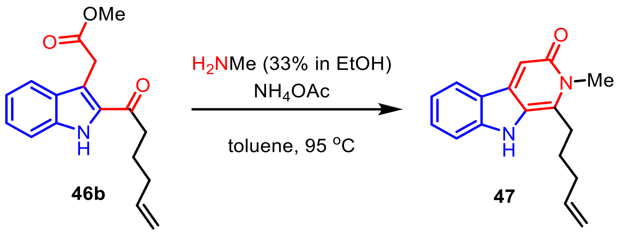

Inspired from known procedures.[14,15]
A solution of $MeNH_2$ (0.4 mL of 33% in EtOH, 3.36 mmol) was added to a solution of **46b** (16 mg, 0.0561 mmol) and NH_4OAc (13 mg, 0.168 mmol) in 1 mL of anhydrous toluene at room temperature. The reaction mixture was then heated to 95 °C and stirred overnight. After concentration under reduced pressure, the crude mixture was purified by preparative TLC on silica gel (CH_2Cl_2/Methanol 15:1) to give 10 mg of **47** as a fluorescent yellow solid (66% yield).

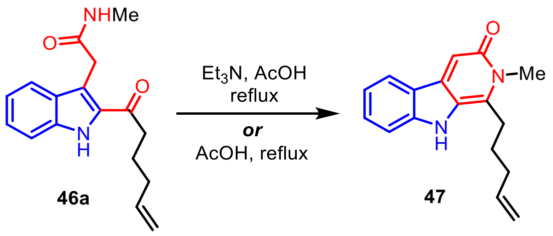

Inspired from known procedures.[14,15]
Et_3N (27 µL, 0.19 mmol) was added to a solution of **46a** (11 mg, 0.0387 mmol) in 1 mL of acetic acid and the reaction mixture was stirred at reflux for 1.5 h. The mixture was then cooled-down and then poured into water, and a solution of ammonium hydroxide was added until it reached pH ≥ 7. Then the resulting mixture was extracted with 5 times CH_2Cl_2. The combined organic layers were dried over Na_2SO_4, filtered and concentrated under reduced pressure. The crude products were purified by preparative TLC on silica gel (CH_2Cl_2/Methanol 15:1) to give 9 mg of **47** as a fluorescent yellow solid (87% yield).

A solution of **46a** (14 mg, 0.0492 mmol) in 1 mL of acetic acid was stirred at reflux for 1.5 h. The mixture was then cooled-down and then poured into water, and a solution of ammonium hydroxide was added until it reached pH ≥ 7. Then the resulting mixture was extracted with 5 times CH_2Cl_2. The

combined organic layers were dried over Na_2SO_4, filtered and concentrated under reduced pressure. The crude products were purified by preparative TLC on silica gel (CH_2Cl_2/Methanol 15:1) to give 11 mg of **47** as a fluorescent yellow solid (85% yield).

R$_f$ = 0.41 (CH_2Cl_2/Methanol 10:1)
^{1}H NMR (360 MHz, MeOD) δ 7.92 (d, *J* = 7.7 Hz, 1H), 7.47 (t, *J* = 7.7 Hz, 1H), 7.32 (d, *J* = 7.7 Hz, 1H), 7.07 (t, *J* = 7.7 Hz, 1H), 6.98 (s, 1H), 5.91 (ddt, *J* = 17.0, 10.5, 7.5 Hz, 1H), 5.07 (d, *J* = 17.0 Hz, 1H), 5.00 (d, *J* = 10.5 Hz, 1H), 3.73 (s, 3H), 3.06 (t, *J* = 7.5 Hz, 2H), 2.25 (q, *J* = 7.5 Hz, 2H), 1.78 (pent, *J* = 7.5 Hz, 2H).
^{13}C NMR (90 MHz, MeOD) δ 162.9, 146.8, 139.5, 138.8, 134.0, 131.7, 128.1, 123.8, 121.5, 120.2, 116.0, 112.3, 102.6, 34.6, 33.0, 29.8, 28.3.
HRMS (m/z): $[M + H]^+$ calcd. for $C_{17}H_{19}N_2O^+$ 267.1492, found 267.1486.

Compound (±)-48: (3a*R*)-11-methyl-2,3,3a,4,5,10-hexahydro-1*H*-10*b*,5-(epiminomethano)cyclopenta[*a*]carbazol-12-one

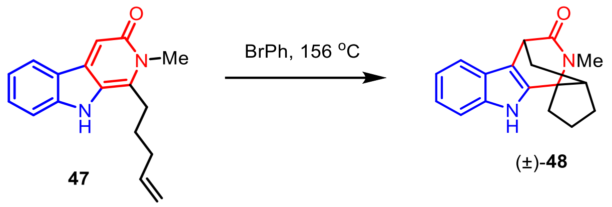

47 (±)-**48**

Inspired from a known procedure.[16]
A solution of pyridone **47** (50 mg, 0.188 mmol) in bromobenzene (5 mL) was heated to 156 °C for about 14 h, then it was allowed to cool down to room temperature and concentrated under reduced pressure. The crude product was purified by preparative TLC (Petroleum ether /EtOAc 1:1) to give 20 mg of (±)-**48** as a white solid (40% yield).

R$_f$ = 0.37 (CH_2Cl_2/MeOH 20:1) and 0.25 (Petroleum ether /EtOAc 1:1)
^{1}H NMR (300 MHz, $CDCl_3$) δ 8.49 (s, 1H), 7.55 – 7.51 (m, 1H), 7.37 – 7.33 (m, 1H), 7.15 – 7.10 (m, 2H), 4.19 (s, 1H), 2.85 (s, 3H), 2.53 – 2.40 (m, 1H), 2.36 – 2.21 (m, 1H), 2.15 – 1.70 (m, 6H), 1.45 – 1.34 (m, 1H).
^{13}C NMR (75 MHz, $CDCl_3$) δ 177.4, 143.2, 136.1, 124.8, 121.4, 120.4, 118.3, 113.3, 111.9, 69.5, 51.2, 42.8, 29.3, 28.6, 28.2, 25.5, 24.1.
HRMS (m/z): $[M + H]^+$ calcd. for $C_{17}H_{19}N_2O^+$ 267.1492, found 267.1482.

Compound (±)-23: 2-ethoxy-5-(methoxycarbonyl)-3-vinyl-3,4-dihydro-2*H*-pyran-4-yl)acetic acid

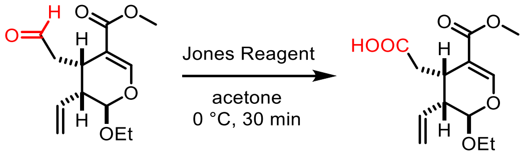

(±)-**16** (±)-**23**

Inspired from a known procedure.[17]
To a solution of aldehyde (±)-**16** (55 mg, 0.216 mmol), which was prepared according to our modification of the Tietze procedure,[2] in acetone (4 mL), a solution of the Jones reagent (0.12 mL of 2.67 M in water) was added dropwise at 0 °C and the reaction mixture was stirred at 0 °C for about 30 min. Upon completion of the reaction (monitoring by TLC), the reaction mixture was quenched with

*iso*propanol at 0 ºC. The resulting mixture was filtered over celite and concentrated under reduced pressure. The crude product was purified by flash chromatography on silica gel (CH_2Cl_2/MeOH 10:1) to give 48 mg of (±)-**23** as a white solid (83% yield).

R_f = 0.37 (CH_2Cl_2/MeOH 10:1)
^{1}H NMR (300 MHz, $CDCl_3$) δ 7.47 (d, J = 1.7 Hz, 1H), 5.59 (dt, J = 17.4, 9.7 Hz, 1H), 5.29 – 5.20 (m, 2H), 4.94 (d, J = 4.1 Hz, 1H), 3.85 (dq, J = 9.6, 7.1 Hz, 1H), 3.70 (s, 3H), 3.62 (dq, J = 9.6, 7.1 Hz, 1H), 3.36 – 3.24 (m, 1H), 2.94 (dd, J = 16.6, 5.2 Hz, 1H), 2.77 – 2.71 (m, 1H), 2.33 (dd, J = 16.6, 8.0 Hz, 1H), 1.22 (t, J = 7.1 Hz, 3H).
^{13}C NMR (100 MHz, $CDCl_3$) δ 178.0, 167.6, 153.0, 133.2, 120.3, 108.2, 100.5, 64.8, 51.4, 44.7, 34.7, 27.8, 15.2.
HRMS (m/z): $[M + Na]^+$ calcd. for $C_{13}H_{18}NaO_6^+$ 293.0996, found 293.0993.

Compound (±)-51: methyl -2-ethoxy-4-((2-methyl-3-oxo-3,9-dihydro-2*H*-pyrido[3,4-*b*]indol-1-yl)methyl)-3-vinyl-3,4-dihydro-2*H*-pyran-5-carboxylate

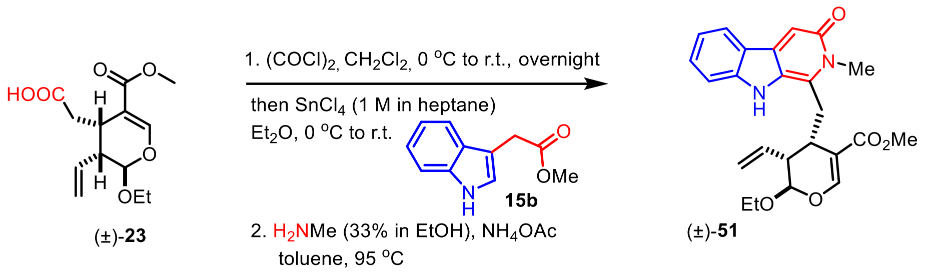

Inspired from known procedure.[13-15]
To a solution of (±)-**23** (170 mg, 0.629 mmol) in CH_2Cl_2 (3 mL) at 0 ºC was added dropwise oxalyl chloride (97 mg, 0.767 mmol) and then the reaction mixture was allowed to stir at room temperature overnight. It was then concentrated to dryness and the crude material was directly treated for next step without isolation. To the crude reaction mixture dissolved in Et_2O (6 mL) was added **15b** (133.8 mg, 0.708 mmol) followed by a dropwise addition of a solution of $SnCl_4$ (0.06 mL of 1 M in heptane, 0.06 mmol) at 0 ºC. The reaction mixture was then allowed to stir at room temperature for 2 h. The reaction mixture was quenched with a saturated aqueous solution of KF and the resulting mixture was extracted 5 times with CH_2Cl_2. The combined organic layers were dried over Na_2SO_4, filtered and concentrated under reduced pressure. The crude product was purified by flash chromatography on silica gel (CH_2Cl_2/Petroleum ether 10:1 to 5:1) to give 120 mg of acylation product in mixture with an unknown compound as a colorless oil.
To a solution of 13 mg of this mixture containing the acylation product in toluene (1 mL) were added H_2NMe (0.2 mL of 33% in EtOH) and NH_4OAc (7 mg, 0.09 mmol) at room temperature and the reaction mixture was stirred at 95 ºC for about 6 h. Upon completion of the reaction (monitoring by TLC), the reaction mixture was allowed to cool down to room temperature and was then concentrated under reduced pressure. The crude product was purified by flash chromatography on silica gel (CH_2Cl_2/MeOH 20:1 to10:1) to give 4 mg of **(±)-50** as a yellow solid (14% yield over 2 steps).

R_f = 0.41 (CH_2Cl_2/MeOH 10:1)
^{1}H NMR (360 MHz, $CDCl_3$) δ 9.08 (br, 1H), 7.89 (dt, J = 8.0, 1.2 Hz, 1H), 7.59 (s, 1H), 7.46 (ddd, J = 8.0, 7.1, 1.2 Hz, 1H), 7.30 (dt, J = 10.2, 1.2 Hz 1H), 7.09 (ddd, J = 8.0, 7.1, 1.2 Hz, 1H), 7.04 (s, 1H), 5.55 (ddd, J = 17.5, 10.3, 7.0 Hz, 1H), 5.25 (d, J = 2.7, 1H), 5.01 (d, J = 17.5 Hz, 1H), 4.92 (d, J = 10.3

Hz, 1H), 4.14 – 4.04 (m, 1H), 3.84 (s, 3H), 3.79 – 3.74 (m, 1H), 3.73 (s, 3H), 3.69 – 3.60 (m, 1H), 3.38 – 3.32 (m, 1H), 3.32 – 3.24 (m, 1H), 2.69 – 2.62 (m, 1H), 1.42 (t, J = 7.1 Hz, 3H).
^{13}C NMR (100 MHz, $CDCl_3$) δ 168.4, 161.9, 153.0, 144.8, 137.8, 132.6, 130.4, 130.3, 126.6, 123.2, 121.2, 119.3, 118.9, 111.1, 109.5, 103.5, 100.2, 66.1, 52.0, 43.2, 32.9, 32.4, 30.6, 15.4.
HRMS (m/z): $[M + H]^+$ calcd. for $C_{24}H_{27}N_2O_5^+$ 423.1914, found 423.1899.

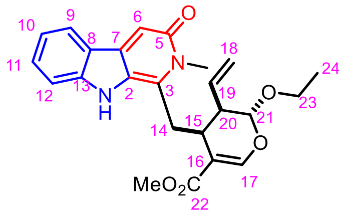

Assignment of ^{1}H (δ_H) and ^{13}C ($\delta_{^{13}C}$) NMR chemical shifts of compound (±)-51.

**Position**	**$\delta_{^1H}$ (ppm)** 360 MHz, $CDCl_3$	**$\delta_{^{13}C}$ (ppm)** 100 MHz, $CDCl_3$
**2**	-	126.6, C
**3**	-	130.4, C
**5**	-	161.9, C
**6**	7.04, s	103.5, CH
**7**	-	137.8, C
**8**	-	121.2, C
**9**	7.89, dt (8.0, 1.2)	123.2, CH
**10**	7.09, ddd (8.0, 7.1, 1.2)	119.3, CH
**11**	7.46, ddd (8.0, 7.1, 1.2)	130.3, CH
**12**	7.30, dt (8.0, 1.2)	111.1, CH
**13**	-	144.8, C
**14a**	3.69 – 3.60, m	30.6, CH_2
**14b**	3.32 – 3.24, m	
**15**	3.38 – 3.32, m	32.4, CH
**16**	-	109.5, C
**17**	7.59, s	153.0, CH
**18a**	5.01, d (17.5)	118.9, CH_2
**18b**	4.92, d (10.3)	
**19**	5.55, ddd (17.7, 10.3, 7.0)	132.6, CH
**20**	2.69 – 2.62, m	43.2, CH
**21**	5.25, d, (2.7)	100.2, CH
**22**	-	168.4, C
**23a**	4.14 – 4.04, m	66.1, CH_2
**23b**	3.79 – 3.74, m	
**24**	1.42, t (7.1)	15.4, CH_3
**N-Me**	3.84, s	32.9, CH_3
**COOMe**	3.73, s	52.0, CH_3
**NH**	9.08, br	-

Compound (±)-53: methyl-4-ethoxy-13-methyl-14-oxo-4,4a,4b,5,6,11,12,12a-octahydro-11b,6-(epiminomethano)pyrano[3',4':3,4]cyclopenta[1,2-*a*]carbazole-1-carboxylate

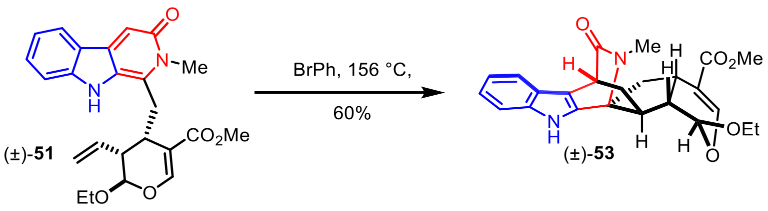

Inspired from a known procedure.[16] A solution of (±)-**51** (5 mg, 0.0118 mmol) in bromobenzene (1 mL) was stirred at 156 ºC for about 5 h. Upon completion of the reaction (monitoring by TLC), the reaction mixture was allowed to cool down to room temperature and was then concentrated under reduced pressure. The crude product was purified by preparative TLC (CH_2Cl_2/MeOH 10:1) to give 3 mg of (±)-**53** as a white solid (60% yield).

R_f = 0.40 (CH_2Cl_2/MeOH 10:1)
^{1}H NMR (360 MHz, $CDCl_3$) δ 8.08 (br, 1H), 7.55 – 7.50 (m, 1H), 7.41 (s, 1H), 7.36 – 7.30 (m, 1H), 7.15 – 7.10 (m, 2H), 4.97 (d, *J* = 2.9 Hz, 1H), 4.18 (t, *J* = 2.6 Hz, 1H), 3.95 – 3.87 (m, 1H), 3.78 (s, 3H), 3.58– 3.44 (m, 1H), 3.43 – 3.33 (m, 1H), 3.21 (dd, *J* = 14.9, 8.4 Hz, 1H), 2.94 (s, 3H), 2.45 – 2.35 (m, 1H), 2.17 – 2.05 (m, 2H), 2.03 – 1.97 (m, 1H), 1.96 – 1.88 (m, 1H), 1.18 (t, *J* = 7.0 Hz, 3H).
^{13}C NMR (100 MHz, $CDCl_3$) δ 177.3, 167.7, 152.3, 142.1, 136.1, 124.8, 121.7, 120.5, 118.5, 113.5, 112.9, 111.9, 102.1, 68.6, 65.9, 51.6, 49.1, 46.1, 42.7, 36.0, 34.9, 28.7, 28.6, 15.2.
HRMS (m/z): $[M + H]^+$ calcd. for $C_{24}H_{27}N_2O_5^+$ 423.1914, found 423.1900.
$[M + Na]^+$ calcd. for $C_{24}H_{26}N_2NaO_5^+$ 445.1734, found 445.1712.

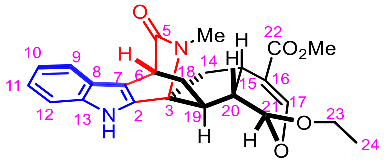

Assignment of ^{1}H (δ_H) and ^{13}C ($\delta_{^{13}C}$) NMR chemical shifts of compound (±)-53.

**Position**	**$\delta_{^1H}$ (ppm)** 360 MHz, $CDCl_3$	**$\delta_{^{13}C}$ (ppm)** 100 MHz, $CDCl_3$
**2**	-	142.1, C
**3**	-	68.6, C
**5**	-	177.3, C
**6**	4.18, t (2.6)	42.7, CH
**7**	-	113.5, C
**8**	-	124.8, C
**9**	7.55 – 7.50, m	118.5, CH
**10**	7.15 – 7.10, m (overlap)	120.5, CH
**11**	7.15 – 7.10, m (overlap)	121.7, CH
**12**	7.36 – 7.30, m	111.9, CH
**13**	-	136.1, C
**14a**	3.21, dd (14.9, 8.4)	34.9, CH_2
**14b**	2.03 – 1.97, m	

**15**	3.43 – 3.33, m	36.0, CH
**16**	-	112.9, C
**17**	7.41, s	152.3, CH
**18a**	2.17 – 2.05, m (overlap)	28.7, CH_2
**18b**	1.96 – 1.88, m	
**19**	2.17 – 2.05, m (overlap)	49.1, CH
**20**	2.45 – 2.35, m	46.1, CH
**21**	4.97, d, (2.9)	102.1, CH
**22**	-	167.7, C
**23a**	3.95 – 3.87, m	65.9, CH_2
**23b**	3.58 – 3.44, m	
**24**	1.18, t (7.0)	15.2, CH_3
**N-Me**	2.94, s	28.6, CH_3
**COOMe**	3.78, s	51.6, CH_3
**NH**	8.08, br	-

Compound (−)-24 : 2-((2*S*,3*R*,4*S*)-5-(methoxycarbonyl)-2-(((2*S*,3*R*,4*S*,5*R*,6*R*)-3,4,5-triacetoxy-6-(acetoxymethyl)tetrahydro-2*H*-pyran-2-yl)oxy)-3-vinyl-3,4-dihydro-2*H*-pyran-4-yl)acetic acid[17]

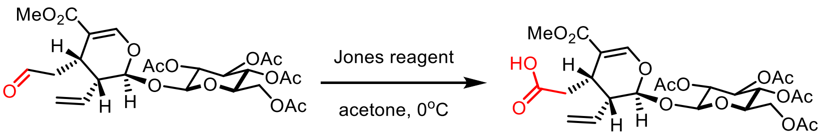

Prepared according to a known procedure.[17] To secologanin tetraacetate (−)-**17** (60 mg, 0.108 mmol, prepared according to our modifications of Ishikawa's procedures[18]) in acetone (2 mL) at 0 ºC was added dropwise a solution of Jones Reagent (0.06 mL of 2.67 M in water) and the reaction mixture was stirred at 0 ºC for about 30 min. Upon completion of the reaction (monitoring by TLC), the mixture was quenched with isopropanol at 0 ºC and was filtered over celite and concentrated under reduced pressure. The crude product was purified by preparative TLC on silica gel (CH_2Cl_2/MeOH 10:1) to give 58 mg of (−)-**24** as a white solid (93% yield).

R_f = 0.44 (CH_2Cl_2/MeOH 10:1)
^{1}H NMR (360 MHz, $CDCl_3$) δ 7.40 (d, *J* = 2.0 Hz, 1H), 5.53 (dt, *J* = 17.3, 9.9 Hz, 1H), 5.31 – 5.23 (m, 3H), 5.20 (d, *J* = 9.5 Hz, 1H), 5.10 (t, *J* = 9.5 Hz, 1H), 5.01 (dd, *J* = 9.5, 8.1 Hz, 1H), 4.88 (d, *J* = 8.1 Hz, 1H), 4.28 (dd, *J* = 12.4, 4.4 Hz, 1H), 4.14 (dd, *J* = 12.4, 2.1 Hz, 1H), 3.74 – 3.71 (m, 1H), 3.69 (s, 3H), 3.20 – 3.12 (m, 1H), 3.05 (dd, *J* = 17.2, 4.7 Hz, 1H), 2.92 – 2.86 (m, 1H), 2.30 (dd, *J* = 17.2, 9.1 Hz, 1H), 2.10 (s, 3H), 2.02 (s, 3H), 2.00 (s, 3H), 1.90 (s, 3H).
^{13}C NMR (90 MHz, $CDCl_3$) δ 177.1, 170.9, 170.4, 169.5, 169.1, 166.9, 151.2, 132.0, 121.3, 109.6, 96.0, 95.7, 72.6, 72.4, 70.7, 68.2, 61.8, 51.5, 43.4, 33.6, 26.2, 20.9, 20.7, 20.7, 20.2.
HRMS (m/z): $[M + Na]^+$ calcd. for $C_{25}H_{32}NaO_{15}^+$ 595.1628, found 595.1618.
$[\alpha]^{25}_D$ = – 78.2 (c 0.55, $CHCl_3$)

Compound (−)-50b: (2*R*,3*R*,4*S*,5*R*,6*S*)-2-(acetoxymethyl)-6-(((2*S*,3*R*,4*S*)-4-(2-(3-(2-methoxy-2-oxoethyl)-1*H*-indol-2-yl)-2-oxoethyl)-5-(methoxycarbonyl)-3-vinyl-3,4-dihydro-2*H*-pyran-2-yl)oxy)tetrahydro-2*H*-pyran-3,4,5-triyl triacetate

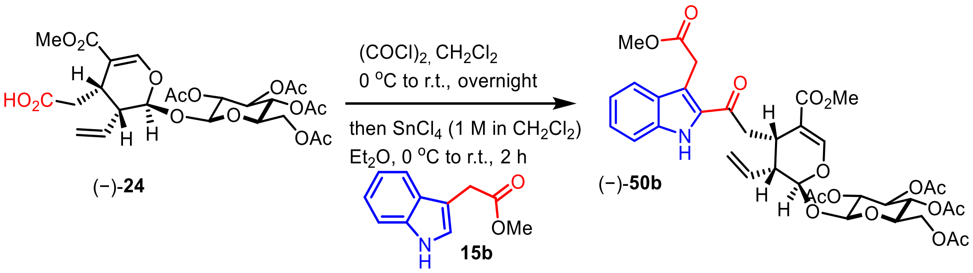

Inspired from known procedures.[13] To a solution of (−)-**24** (47 mg, 0.0821 mmol) in CH_2Cl_2 (0.5 mL) at 0 °C was added dropwise oxalyl chloride (20 mg, 0.16 mmol) and then, the reaction mixture was allowed to stir at room temperature overnight. The reaction mixture was concentrated to dryness under reduced pressure and the crude material was directly treated for next step without isolation. To the crude reaction mixture dissolved in Et_2O (1 mL) at 0 °C were successively added **15b** (30 mg, 0.16 mmol) and dropwise a solution of $SnCl_4$ (0.008 mL of 1 M in CH_2Cl_2, 0.008 mmol). The reaction mixture was allowed to stir at room temperature for 2 h. The reaction mixture was quenched with a saturated aqueous solution of KF. The resulting mixture was extracted 5 times with CH_2Cl_2. The combined organic layers were dried over Na_2SO_4, filtered and concentrated under reduced pressure. The crude product was purified by preparative TLC on silica gel (Dichloromethane/Ethyl acetate 5:1) to give 28 mg of (−)-**50b** as a colorless oil (46% yield).

R_f = 0.42 (Dichloromethane/Ethyl acetate 5:1)
^{1}H NMR (360 MHz, $CDCl_3$) δ 9.68 (br, 1H), 7.67 (dt, *J* = 8.2, 1.0 Hz, 1H), 7.46 (d, *J* = 1.9 Hz, 1H), 7.41 (dt, *J* = 8.2, 1.0 Hz, 1H), 7.34 (ddd, *J* = 8.2, 6.5, 1.0 Hz, 1H), 7.15 (ddd, *J* = 8.2, 6.5, 1.0 Hz, 1H), 5.57 (dt, *J* = 17.0, 10.0 Hz, 1H), 5.32 (d, *J* = 3.4 Hz, 1H), 5.26 – 5.18 (m, 2H), 5.17 – 5.14 (m, 1H), 5.13 – 5.06 (m, 1H), 4.99 (dd, *J* = 9.5, 8.1 Hz, 1H), 4.90 (d, *J* = 8.1 Hz, 1H), 4.28 (dd, *J* = 12.4, 4.4 Hz, 1H), 4.19 – 4.10 (m, 3H), 3.80 – 3.74 (m, 1H), 3.73 (s, 3H), 3.67 (s, 3H), 3.67 – 3.61 (m, 1H), 3.49 – 3.39 (m, 1H), 2.96 (ddd, *J* = 10.0, 5.9, 3.4 Hz, 1H), 2.81 (dd, *J* = 16.3, 10.0 Hz, 1H), 2.10 (s, 3H), 2.02 (s, 3H), 1.99 (s, 3H), 1.91 (s, 3H).
^{13}C NMR (90 MHz, $CDCl_3$) δ 191.3, 171.3, 170.8, 170.3, 169.3, 168.8, 167.5, 151.6, 135.8, 132.3, 132.0, 128.2, 126.2, 121.2, 120.9, 120.7, 114.5, 112.2, 109.9, 96.0, 95.9, 72.5, 72.2, 70.7, 68.1, 61.6, 52.1, 51.6, 43.2, 39.8, 31.1, 27.0, 20.8, 20.6, 20.6, 20.2.
HRMS (m/z): $[M + H]^+$ calcd. for $C_{36}H_{42}NO_{16}^+$ 744.2498, found 744.2463.
$[M + Na]^+$ calcd. for $C_{36}H_{41}NNaO_{16}^+$ 766.2318, found 766.2283.
$[\alpha]^{25}_D$ = − 90.0 (c 0.35, $CHCl_3$)

Compound (−)-50a: (2*R*,3*R*,4*S*,5*R*,6*S*)-2-(acetoxymethyl)-6-(((2*S*,3*R*,4*S*)-5-(methoxycarbonyl)-4-(2-(3-(2-(methylamino)-2-oxoethyl)-1*H*-indol-2-yl)-2-oxoethyl)-3-vinyl-3,4-dihydro-2*H*-pyran-2 yl)oxy)tetrahydro-2*H*-pyran-3,4,5-triyl triacetate

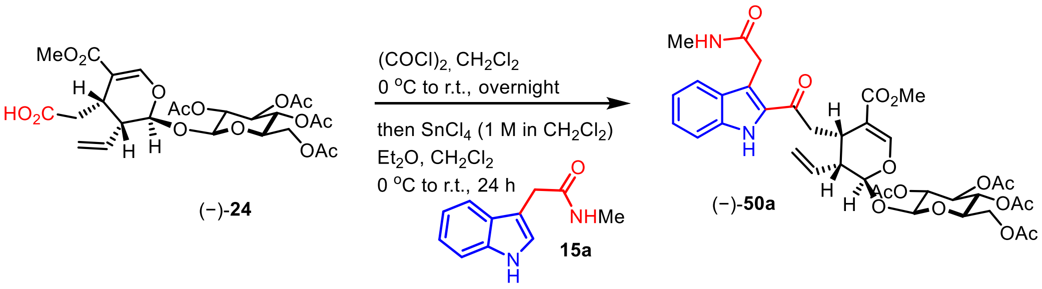

Inspired from a known procedure.[13] To a solution of (−)-**24** (23 mg, 0.04017 mmol) in CH_2Cl_2 (0.5 mL) at 0 °C, was added dropwise oxalyl chloride (7 µL, 0.08 mmol) and then the reaction mixture was allowed to stir at room temperature overnight. The reaction mixture was concentrated to dryness under reduced pressure and the crude material was directly treated for next step without purification. To the crude reaction mixture dissolved in Et_2O (0.4 mL) and CH_2Cl_2 (0.1 mL) at 0 °C, was added **15a** (15 mg,0.08 mmol) followed by the dropwise addition of a solution of tin chloride (0.04 mL, 1 M in CH_2Cl_2, 0.04 mmol). The reaction mixture was allowed to stir at room temperature overnight. The reaction mixture was quenched with a saturated aqueous solution of KF and the resulting mixture was extracted 5 times with CH_2Cl_2. The combined organic layers were dried over Na_2SO_4 and concentrated under reduced pressure. The crude product was purified by preparative TLC on silica gel (EtOAc) to give 13 mg of (−)-**50a** as a colorless oil (44% yield).

R_f = 0.46 (Ethyl acetate)
^{1}H NMR (360 MHz, $CDCl_3$) δ 9.94 (br, 1H), 7.80 (dt, *J* = 8.2, 0.9 Hz, 1H), 7.46 (d, *J* = 1.9 Hz, 1H), 7.43 (dt, *J* = 8.2, 0.9 Hz, 1H), 7.37 (ddd, *J* = 8.2, 6.7, 0.9 Hz, 1H), 7.18 (ddd, *J* = 8.2, 6.7, 0.9 Hz,, 1H), 6.23 (br, 1H), 5.58 (dt, *J* = 17.4, 9.6 Hz, 1H), 5.34 (d, *J* = 3.4 Hz, 1H), 5.26 – 5.16 (m, 3H), 5.09 (t, *J* = 9.5 Hz, 1H), 4.96 (dd, *J* = 9.5, 8.1 Hz, 1H), 4.88 (d, *J* = 8.1 Hz, 1H), 4.28 (dd, *J* = 12.4, 4.4 Hz, 1H), 4.19 – 4.10 (m, 1H), 4.02 (d, *J* = 2.7 Hz, 2H), 3.73 (s, 3H), 3.73 – 3.70 (m, 1H), 3.62 (dd, *J* = 15.2, 2.3 Hz, 1H), 3.44 – 3.37 (m, 1H), 2.89 (ddd, *J* = 9.6, 5.9, 3.4 Hz, 1H), 2.75 – 2.67 (m, 1H), 2.70 (d, *J* = 4.5 Hz, 3H), 2.10 (s, 3H), 2.02 (s, 3H), 1.98 (s, 3H), 1.88 (s, 3H).
^{13}C NMR (90 MHz, $CDCl_3$) δ 192.3, 171.3, 170.9, 170.4, 169.5, 168.9, 167.8, 152.1, 136.4, 132.4, 131.7, 128.1, 126.9, 121.5, 121.5, 121.2, 117.1, 112.4, 109.6, 95.9 (2C), 72.5, 72.4, 70.7, 68.2, 61.7, 51.9, 43.5, 40.1, 33.7, 27.6, 26.5, 20.9, 20.7 (2C), 20.3.
HRMS (m/z): $[M + H]^+$ calcd. for $C_{36}H_{43}N_2O_{15}^+$ 743.2658, found 744.2651.
$[M + Na]^+$ calcd. for $C_{36}H_{42}N_2NaO_{15}^+$ 765.2477, found 765.2469.
$[\alpha]^{25}_D$ = − 77.0 (c 1.00, $CHCl_3$)

Compound (−)-7a: ophiorrhine G[19]

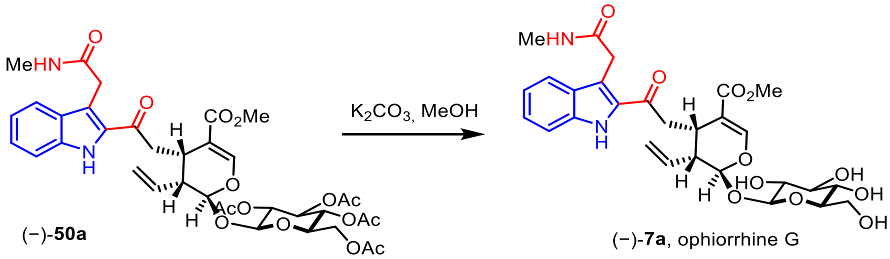

(−)-**50a**
(−)-**7a**, ophiorrhine G

To a solution of (−)-**50a** (13 mg, 0.0175 mmol) of methanol (1 mL) at 0 °C, was added K_2CO_3 (7 mg, 0.05 mmol). The reaction mixture was stirred at 0 °C for 20 min and was then directly purified by silica gel preparative TLC (CH_2Cl_2[saturated with ammonium hydroxide]/MeOH 5:1) to give 10 mg of ophiorrhine G (−)-**7a** as a white solid (quant.).

R_f = 0.26 (CH_2Cl_2 [saturated with ammonium hydroxide]/MeOH 5:1)
^{1}H NMR (400 MHz, MeOD) δ 7.68 (dt, *J* = 8.3, 1.1 Hz, 1H), 7.53 (d, *J* = 1.9 Hz, 1H), 7.44 (dt, *J* = 8.3, 1.1 Hz, 1H), 7.31 (ddd, *J* = 8.3, 6.8, 1.1 Hz, 1H), 7.11 (ddd, *J* = 8.3, 6.8, 1.1 Hz, 1H), 5.67 (dt, J = 17.0, 10.0 Hz, 1H), 5.54 (d, *J* = 3.8 Hz, 1H), 5.16 (dd, J = 17.0, 1.8 Hz, 1H), 5.14 (dd, *J* = 10.0, 1.8 Hz, 1H), 4.69 (d, *J* = 7.8 Hz, 1H), 4.08 (d, *J* = 15.5 Hz, 1H), 4.00 (d, *J* = 15.5 Hz, 1H), 3.91 (dd, *J* = 12.0, 2.0 Hz, 1H), 3.68 (dd, *J* = 12.0, 5.8 Hz, 1H), 3.68 – 3.64 (m, 1H), 3.61 (s, 3H), 3.55 (dd, *J* = 17.1, 5.2 Hz, 1H),

3.42 – 3.35 (m, 1H), 3.34 – 3.28 (m, 2H), 3.25 (dd, J = 9.2, 7.8 Hz, 1H), 2.97 (dd, J = 17.1, 8.0 Hz, 1H), 2.91 – 2.86 (m, 1H), 2.68 (s, 3H).

^{13}C NMR (100 MHz, MeOD) δ 194.5, 174.5, 169.1, 153.8, 138.1, 134.8, 133.6, 129.2, 127.0, 121.5 (2C), 120.5, 116.9, 113.5, 110.4, 100.0, 97.6, 78.4, 78.0, 74.6, 71.6, 62.8, 51.7, 45.4, 41.0, 33.7, 27.8, 26.6.

HRMS (m/z): $[M + H]^+$ calcd. for $C_{28}H_{35}N_2O_{11}^+$ 575.2235, found 575.2219.

$[M + Na]^+$ calcd. for $C_{28}H34_4N_2NaO_{11}^+$ 597.2055, found 597.2039.

$[\alpha]^{27}_D$ = − 105.0 (c 0.4, MeOH)

Compound (−)-6a: ophiorrhiside E [20]

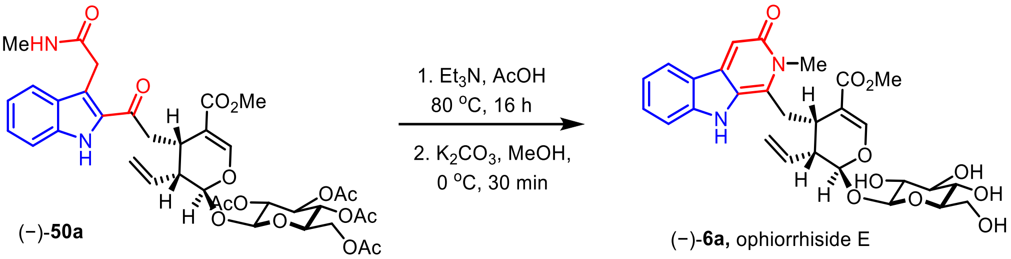

(−)-**50a**

(−)-**6a**, ophiorrhiside E

Inspired from known procedures.[14,15] To (−)-**50a** (18 mg, 0.0242 mmol) in AcOH (1.0 mL) at room temperature was added Et_3N (50 µL, 0.364 mmol) and then the reaction mixture was stirred at 80 °C for 1 h, after which an addition of Et_3N (50 µL, 0.364 mmol) was effected followed by a third addition of Et_3N (50 µL, 0.364 mmol) after an additional 1 h and the reaction mixture was stirred for an additional 14 h. Then the mixture was cooled to room temperature, diluted with CH_2Cl_2, and then quenched with a solution of ammonium hydroxide until it reached pH ≥ 7. The resulting mixture was extracted 5 times with CH_2Cl_2 in. The combined organic layers were dried over Na_2SO_4, filtered and concentrated under reduced pressure. The crude product was used directly in the next step without purification. It was dissolved in MeOH (1.0 mL) onto which K_2CO_3 (10 mg, 0.073 mmol) was added at 0 °C. The reaction mixture was stirred at 0 °C for 30 min and was then directly purified by silica gel preparative TLC (CH_2Cl_2[saturated with ammonium hydroxide]/EtOAc[saturated with ammonium hydroxide] /MeOH 3:2:1) to give 8 mg of ophiorrhine G (−)-**7a** as a white solid (57%) and 4 mg of ophiorrhiside E (−)-**6a** as a yellow oil (30%).

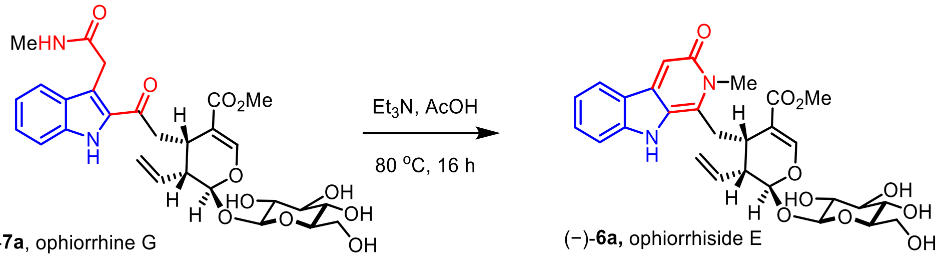

(−)-**7a**, ophiorrhine G

(−)-**6a**, ophiorrhiside E

To ophiorrhine G (−)-**7a** (6 mg, 0.0104 mmol) in AcOH (0.5 mL) at room temperature, was added Et_3N (22 µL, 0.15 mmol) and then the reaction mixture was stirred at 80 °C for 1 h, after which an addition of Et_3N (22 µL, 0.15 mmol) was effected followed by a third addition of Et_3N (22 µL, 0.15 mmol) after an additional 1 h and the reaction mixture was stirred for an additional 14 h. Then the mixture was cooled to room temperature, diluted with CH_2Cl_2, and then quenched with a solution of ammonium hydroxide until it reached pH ≥ 7. The resulting mixture was extracted 5 times with CH_2Cl_2. The combined organic layers were dried over Na_2SO_4, filtered and concentrated under reduced pressure. The crude product was purified by silica gel preparative TLC (CH_2Cl_2[saturated with ammonium

hydroxide]/EtOAc[saturated with ammonium hydroxide]/MeOH 3:2:1) to give 1 mg of ophiorrhiside E (−)-**6a** as a yellow oil (20%).

R_f = 0.12 (CH_2Cl_2[saturated with ammonium hydroxide]/MeOH 5:1)
^{1}H NMR (400 MHz, MeOD) δ 7.95 (d, *J* = 8.0 Hz, 1H), 7.49 (t, *J* = 8.0 Hz, 1H), 7.45 (s, 1H), 7.33 (d, *J* = 8.0 Hz, 1H), 7.09 (t, *J* = 8.0 Hz, 1H), 7.05 (s, 1H), 6.02 (d, *J* = 9.2 Hz, 1H), 6.00 (ddd, *J* = 17.5, 10.7, 7.7 Hz 1H), 5.39 (d, *J* = 17.5 Hz, 1H), 5.31 (d, *J* = 10.7 Hz, 1H), 4.85 (d, *J* = 8.0 Hz, 1H), 4.00 (dd, *J* = 11.7, 2.0 Hz, 1H), 3.82 (s, 3H), 3.71 (dd, *J* = 11.7, 6.5 Hz, 1H), 3.46 –3.20 (m, 7H), 3.07 (s, 3H), 2.79 – 2.72 (m, 1H).
^{13}C NMR (100 MHz, MeOD) δ 168.4, 162.8, 154.7, 146.7, 139.1, 135.8, 131.8, 131.7, 129.4, 123.7, 121.3, 120.2, 119.5, 112.3, 109.8, 102.9, 100.5, 97.0, 78.8, 78.1, 74.7, 71.7, 63.1, 51.5, 45.2, 35.7, 33.6, 32.4.
HRMS (m/z): $[M + H]^+$ calcd. for $C_{28}H_{33}N_2O_{10}^+$ 557.2130, found 557.2104.
$[M + Na]^+$ calcd. for $C_{28}H_{32}N_2NaO_{10}^+$ 579.1949, found 579.1923.
$[\alpha]^{25}_D$ = − 265.0 (c 0.6, MeOH)

Compound (−)-54: (2*R*,3*R*,4*S*,5*R*,6*S*)-2-(acetoxymethyl)-6-(((4*S*,4a*S*,4b*S*,6*R*,11b*S*,12a*S*)-1-(methoxycarbonyl)-13-methyl-14-oxo-4,4a,4b,5,6,11,12,12a-octahydro-11b,6-(epiminomethano)pyrano[3',4':3,4]cyclopenta[1,2-a]carbazol-4-yl)oxy)tetrahydro-2*H*-pyran-3,4,5-triyl triacetate [21]

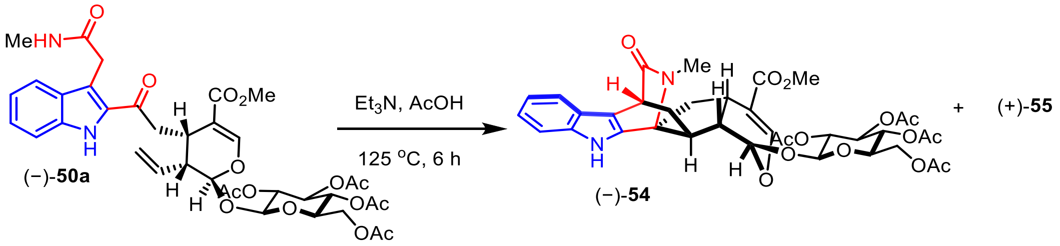

To (−)-**50a** (7 mg, 0.00943 mmol) in AcOH (0.5 mL at room temperature) was added Et_3N (59 µL, 0.425 mmol) and the reaction mixture was stirred at 125 °C for about 6 h. Then the mixture was cooled down to room temperature, diluted with CH_2Cl_2, and then quenched with a solution of ammonium hydroxide until pH ≥ 7. The resulting mixture was extracted 5 times with CH_2Cl_2. The combined organic layers were dried over Na_2SO_4 and concentrated under reduced pressure. The crude product was purified by silica gel preparative TLC (EtOAc) to give 2.0 mg of (+)-**55** (29%) as a white solid and 4.0 mg of (−)-**54** as a white solid (58%).

R_f = 0.56 (Ethyl acetate)
^{1}H NMR (400 MHz, $CDCl_3$) δ 8.13 (br, 1H), 7.57 – 7.53 (m, 1H), 7.38 – 7.34 (m, 1H), 7.30 (s, 1H), 7.19 – 7.13 (m, 2H), 5.35 (d, *J* = 1.5 Hz, 1H), 5.23 (t, *J* = 9.6 Hz, 1H), 5.13 (t, *J* = 9.6 Hz, 1H), 4.99 (dd, *J* = 9.6, 8.1 Hz, 1H), 4.84 (d, *J* = 8.1 Hz, 1H), 4.31 (dd, *J* = 12.4, 4.3 Hz, 1H), 4.24 – 4.22 (m, 1H), 4.16 (dd, *J* = 12.4, 2.4 Hz, 1H), 3.78 (s, 3H), 3.73 (ddd, *J* = 9.6, 4.3, 2.4 Hz, 1H), 3.32 – 3.20 (m, 2H), 2.96 (s, 3H), 2.33 (dd, *J* = 11.2, 9.0 Hz, 1H), 2.13 (s, 3H), 2.18 – 2.09 (m, 1H), 2.05 (s, 3H), 2.03 (s, 3H), 1.95 (s, 3H), 1.94 – 1.87 (m, 3H).
^{13}C NMR (100 MHz, $CDCl_3$) δ 176.2, 170.8, 170.4, 169.5, 169.1, 167.1, 148.9, 141.9, 136.0, 124.7, 121.9, 120.7, 118.5, 114.1, 113.5, 111.9, 96.1, 94.3, 72.6, 72.3, 70.8, 68.2, 67.8, 61.7, 51.7, 50.8, 45.1, 42.3, 33.5, 31.2, 28.7, 26.6, 20.9, 20.7, 20.7, 20.3.
HRMS (m/z): $[M + H]^+$ calcd. for $C_{36}H_{41}N_2O_{14}^+$ 725.2552, found 725.2534.
$[M + Na]^+$ calcd. for $C_{36}H_{40}N_2NaO_{14}^+$ 747.2371, found 747.2355.

$[\alpha]^{25}_{D}$ = − 85.0 (c 1.0, MeOH)

Compound (+)-55: (2R,3R,4S,5R,6S)-2-(acetoxymethyl)-6-(((4S,4aS,12aS)-1-(methoxycarbonyl)-6-(methylcarbamoyl)-4a,11,12,12a-tetrahydro-4H-pyrano[3',4':3,4]cyclopenta[1,2-a]carbazol-4-yl)oxy)tetrahydro-2H-pyran-3,4,5-triyl triacetate

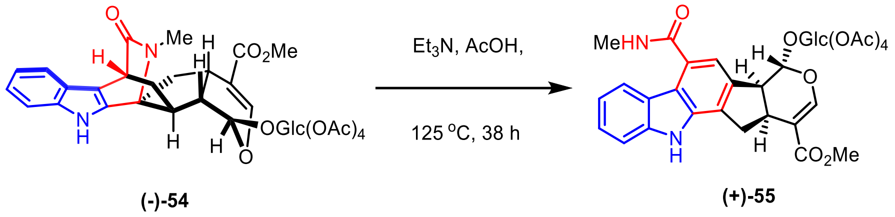

To (−)-**54** (5 mg, 0.00691 mmol) in AcOH (0.4 mL at room temperature) was added Et_3N (43 µL, 0.31 mmol) and the reaction mixture was stirred at 125 °C for about 38 h. Then the mixture was cooled down to room temperature, diluted with CH_2Cl_2, and then quenched with a solution of ammonium hydroxide until pH ≥ 7. The resulting mixture was extracted 5 times with CH_2Cl_2. The combined organic layers were dried over Na_2SO_4 and concentrated under reduced pressure. The crude product was purified by silica gel preparative TLC (EtOAc/petroleum ether 7:3) to give 3.0 mg of (+)-**55** as a pale solid (60%) and trace of (−)-**54** as a white solid.

R_f = 0.43 (EtOAc/ petroleum ether 7:3)
^{1}H NMR (360 MHz, $CDCl_3$) δ 8.37 (d, *J* = 8.1 Hz, 1H), 8.13 (s, 1H), 7.42 (s, 1H), 7.42 – 7.38 (m, 2H), 7.31 (s, 1H), 7.21 (dt, *J* = 8.1, 4.1 Hz, 1H), 6.39 (q, *J* = 4.8 Hz, 1H), 5.41 (d, *J* = 4.3 Hz, 1H), 5.25 (t, *J* = 9.4 Hz, 1H), 5.13 – 5.05 (m, 2H), 4.92 (d, *J* = 7.9 Hz, 1H), 4.25 (dd, *J* = 12.4, 4.8 Hz, 1H), 4.05 (dd, *J* = 12.4, 2.4 Hz, 1H), 3.76 (s, 3H), 3.71 (ddd, *J* = 10.0, 5.0, 2.4 Hz, 1H), 3.63 – 3.51 (m, 3H), 3.16 (d, *J* = 4.8 Hz, 3H), 3.10 – 2.98 (m, 1H), 2.03 (s, 6H), 2.02 (s, 3H), 1.89 (s, 3H).
^{13}C NMR (90 MHz, $CDCl_3$) δ 170.8, 170.40, 170.36, 169.6, 169.5, 167.4, 151.8, 140.1, 137.4, 136.8, 130.1, 126.5, 126.4, 123.9, 122.4, 120.2, 120.1, 115.9, 111.6, 110.8, 96.6, 95.5, 72.5, 72.4, 70.9, 68.4, 61.7, 51.6, 46.9, 36.0, 35.1, 27.0, 20.8, 20.7, 20.7, 20.6.
HRMS (m/z): $[M + H]^+$ calcd. for $C_{36}H_{39}N_2O_{14}^+$ 723.2396, found 723.2377.
$[M + Na]^+$ calcd. for $C_{36}H_{38}N_2NaO_{14}^+$ 745.2215, found 745.2199.
$[\alpha]^{27}_{D}$ = + 6.7 (c 0.75, $CHCl_3$)

Compound (+)-56: methyl (4S,12aS)-6-(methylcarbamoyl)-4-(((2S,3R,4S,5S,6R)-3,4,5-trihydroxy-6-(hydroxymethyl)tetrahydro-2H-pyran-2-yl)oxy)-4a,11,12,12a-tetrahydro-4H-pyrano[3',4':3,4]cyclopenta[1,2-a]carbazole-1-carboxylate

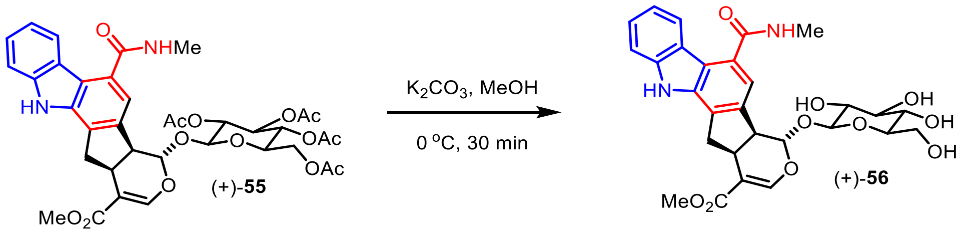

To a solution of (+)-**55** (2 mg, 0.00277 mmol) in MeOH (0.5 mL) at 0 °C was added K_2CO_3 (1 mg, 0.07 mmol). The reaction mixture was stirred at 0 °C for 30 min and was then directly purified by silica gel preparative TLC (CH_2Cl_2 [saturated with ammonium hydroxide]/MeOH 5:1) to give 1.5 mg of (+)-**56** as a white solid (98%).

R_f = 0.21 (CH_2Cl_2 [saturated with ammonium hydroxide]/MeOH 5:1)
^{1}H NMR (400 MHz, MeOD) δ 8.14 (d, J = 8.0 Hz, 1H), 7.55 (s, 1H), 7.47 (d, J = 8.0 Hz, 1H), 7.38 (t, J = 8.0 Hz, 1H), 7.35 (s, 1H), 7.12 (t, J = 8.0 Hz, 1H), 5.52 (d, J = 6.2 Hz, 1H), 4.74 (d, J = 7.6 Hz, 1H), 3.77 (s, 3H), 3.73 – 3.69 (m, 1H), 3.66 – 3.63 (m, 1H), 3.62 – 3.58 (m, 1H), 3.58 – 3.55 (m, 1H), 3.55 – 3.52 (m, 1H), 3.40 – 3.35 (m, 1H), 3.34 – 3.28 (m, 1H), 3.27 – 3.23 (m, 1H), 3.23 – 3.16 (m, 1H), 3.05 (s, 3H), 3.03 – 2.96 (m, 1H).
^{13}C NMR (100 MHz, MeOD) δ 174.1, 169.5, 153.8, 142.2, 139.0, 138.3, 130.4, 127.9, 126.8, 123.7, 123.1, 120.5, 119.9, 116.8, 112.2, 112.0, 100.5, 97.5, 78.4, 78.1, 74.8, 71.4, 62.4, 51.8, 48.1, 37.5, 36.8, 26.9.
HRMS (m/z): $[M + Na]^+$ calcd. for $C_{28}H_{30}N_2NaO_{10}^+$ 577.1793, found 577.1774.
$[\alpha]^{27}_D$ = + 55.00 (c 0.4, MeOH)

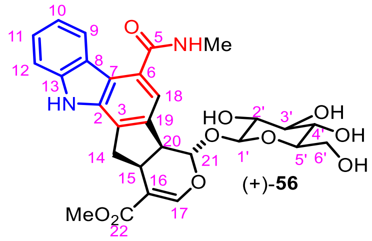

(+)-**56**

Assignment of ^{1}H ($\delta_{^1H}$) and ^{13}C ($\delta_{^{13}C}$) NMR chemical shifts of compound (+)-56.

**Position**	**$\delta_{^1H}$ (ppm)** 400 MHz, CD_3OD	**$\delta_{^{13}C}$ (ppm)** 100 MHz, CD_3OD
**2**	-	138.3, C
**3**	-	127.9, C
**5**	-	174.1, C
**6**	-	130.4, C
**7**	-	120.5, C
**8**	-	123.1, C
**9**	8.14, d (8.0)	123.7, CH
**10**	7.12, t (8.0)	119.9, CH
**11**	7.38, t (8.0)	126.8, CH
**12**	7.47, d, (8.0)	112.0, CH
**13**	-	142.2, C
**14a**	3.62 – 3.58, m	37.5, CH_2
**14b**	3.03 – 2.96, m	
**15**	3.66 – 3.63, m	36.8, CH
**16**	-	112.2, C
**17**	7.55, s	153.8, CH
**18**	7.35, s	116.8, CH
**19**	-	139.0, C
**20**	3.58 – 3.55, m	48.1, CH
**21**	5.52, d, (6.2)	97.5, CH
**22**	-	169.5, C
**1’**	4.74, d (7.6)	100.5, CH
**2’**	3.34 – 3.28, m (overlap)	74.8, CH
**3’**	3.40 – 3.35, m	78.1, CH

**4’**	3.27 – 3.23, m	71.4, CH
**5’**	3.23 – 3.16, m	78.4, CH
**6’a**	3.73 – 3.69, m	62.4, CH_2
**6’b**	3.55 – 3.52, m	
**N-Me**	3.05, s	26.9, CH_3
**COOMe**	3.77, s	51.8, CH_3

Compound (−)-1a: ophiorrhine A

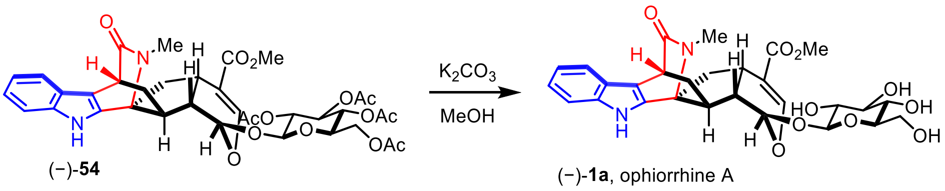

To (−)-**54** (11 mg, 0.0148 mmol) dissolved in 1 mL methanol at 0 °C was added K_2CO_3 (6 mg, 0.0445 mmol). The reaction mixture was stirred at 0 °C for 20 min and was then directly purified by silica gel preparative TLC (CH_2Cl_2[saturated with ammonium hydroxide]/MeOH 5:1) to give 8.0 mg of ophiorrhine A (−)-**1a** as a white solid (quant.).

R*f* = 0.29 (CH_2Cl_2[saturated with ammonium hydroxide]/MeOH 5:1)
^{1}H NMR (400 MHz, MeOD) δ 7.47 (d, *J* = 7.3, 1H), 7.37 (s, 1H), 7.33 (d, *J* = 7.3 Hz, 1H), 7.07 – 6.99 (m, 2H), 5.52 (d, *J* = 1.5 Hz, 1H), 4.59 (d, *J* = 7.9 Hz, 1H), 4.14 (t, *J* = 2.3 Hz, 1H), 3.87 (dd, *J* = 11.9, 1.7 Hz, 1H), 3.77 (s, 3H), 3.64 (dd, *J* = 11.9, 5.3 Hz, 1H), 3.39 – 3.23 (m, 5H), 3.17 (dd, *J* = 9.1, 7.9 Hz, 1H), 2.96 (s, 3H), 2.30 – 2.17 (m, 2H), 1.97 – 1.89 (m, 1H), 1.88 – 1.83 (m, 2H).
^{13}C NMR (100 MHz, MeOD) δ 179.7, 169.1, 151.0, 144.1, 137.9, 125.5, 122.0, 120.7, 118.5, 114.7, 113.1, 112.5, 100.0, 95.7, 78.3, 77.9, 74.6, 71.5, 69.8, 62.6, 52.3, 51.9, 46.9, 43.4, 34.5, 32.5, 28.7, 28.0.
HRMS (m/z): $[M + H]^+$ calcd. for $C_{28}H_{33}N_2O_{10}^+$ 557.2130, found 557.2111.
$[M + Na]^+$ calcd. for $C_{28}H_{32}N_2NaO_{10}^+$ 579.1949, found 579.1931.
$[\alpha]^{27}_D$ = − 68.6 (c 0.35, MeOH)

4. Comparison of ^{1}H, ^{13}C NMR data and optical rotations for natural and synthetic products

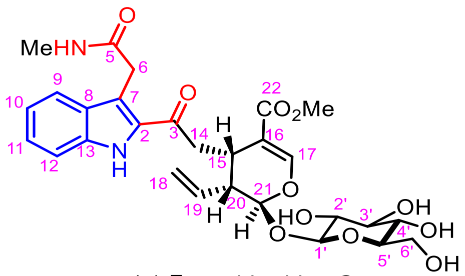

(−)-**7a**, ophiorrhine G

Comparison of the ^{1}H NMR chemical shifts (δ_H) of natural and synthetic ophiorrhine G

Position	*Natural product* **Reported $\delta_{^1H}$ (ppm)**[19] (Methanol-d4, 600 MHz)	*Natural product* **Recalibrated $\delta_{^1H}$ (ppm)**[a] vs Methanol-d4 at 3.31 ppm (Methanol-d4, 600 MHz)	*Our Synthetic product* **$\delta_{^1H}$ (ppm)** vs Methanol-d4 at 3.31 ppm (Methanol-d4, 400 MHz)
**6a**	3.88, d (15.5)	3.98, d (15.5)	4.00, d (15.5)
**6b**	3.98, d (15.5)	4.08, d (15.5)	4.08, d (15.5)
**9**	7.58, d (8.2)	7.68, d (8.2)	7.68 dt (8.3, 1.1)
**10**	7.02, t (8.2)	7.12, t (8.2)	7.11, ddd (8.3, 6.8, 1.1)
**11**	7.22, t (8.2)	7.32, t (8.2)	7.31, ddd (8.3, 6.8, 1.1)
**12**	7.34, d (8.2)	7.44, d (8.2)	7.44, dt (8.3, 1.1)
**14a**	3.44, dd (17.1, 5.2)	3.54, dd (17.1, 5.2)	3.55, dd (17.1, 5.2)
**14b**	2.86, dd (17.1, 8.0)	2.96, dd (17.1, 8.0)	2.97, dd (17.1, 8.0)
**15**	3.54, m	3.64, m	3.68 – 3.64, m (overlap)
**17**	7.45, d (1.8)	7.55, d (1.8)	7.53, d (1.9)
**18a**	5.04, m	5.14, m	5.16, dd (17.0, 1.8)
**18b**			5.14, dd (9.7, 1.8)
**19**	5.56, m	5.66, m	5.67, ddd (17.0, 10.1, 9.7)
**20**	2.76, m	2.86, m	2.91 – 2.86, m
**21**	5.43, d (3.7)	5.53, d (3.7)	5.54, d (3.8)
**1'**	4.58, d (7.9)	4.68, d (7.9)	4.69, d (7.8)
**2'**	3.15, dd (9.1, 7.9)	3.25, dd (9.1, 7.9)	3.25, m (9.2, 7.8)
**3'**	3.27, m	3.37, m	3.42 – 3.35, m
**4'**	3.19, m	3.29, m	3.34 – 3.28, m (overlap)
**5'**	3.22 m	3.32 m	3.34 – 3.30, m (overlap)
**6a'**	3.58, dd (12.0, 6.0)	3.68, dd (12.0, 6.0)	3.68, dd (12.0, 5.8)
**6b'**	3.81, dd (12.0, 2.0)	3.91, dd (12.0, 2.0)	3.91, dd (12.0, 2.0)
**N-Me**	2.58, s	2.68, s	2.68, s
**COOMe**	3.51, s	3.61, s	3.61, s

a) the methanol-d4 peak was at 3.21 ppm on the provided ^{1}H NMR of natural ophiorrhine G[19]

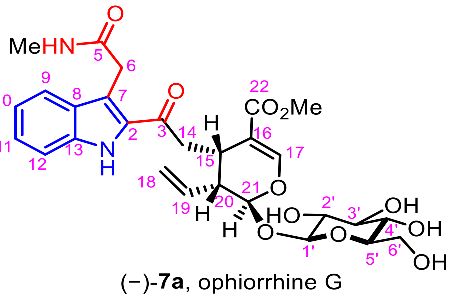

(−)-**7a**, ophiorrhine G

Comparison of the ^{13}C NMR chemical shifts ($\delta_{^{13}C}$) of natural and synthetic ophiorrhine G

**Position**	*Natural product* **Reported $\delta_{^{13}C}$ (ppm)**[19] (Methanol-d4, 150 MHz)	*Natural product* **Recalibrated $\delta_{^{13}C}$ (ppm)**[a] vs Methanol-d4 at 49.0 ppm (Methanol-d4, 150 MHz)	*Our Synthetic product* **$\delta_{^{13}C}$ (ppm)** vs Methanol-d4 at 49.0 ppm (Methanol-d4, 100 MHz)
**2**	132.2, C	133.8, C	133.6, C
**3**	193.1, C	194.7, C	194.5, C
**5**	173.1, C	174.7, C	174.5, C
**6**	32.3, CH_2	33.9, CH_2	33.7, CH_2
**7**	115.5, C	117.1, C	116.9, C
**8**	127.5, C	129.1, C	129.2, C
**9**	120.2, CH	121.8, CH	121.5, CH
**10**	120.1, CH	121.7, CH	121.5, CH
**11**	125.8, CH	127.4, CH	127.0, CH
**12**	112.1, CH	113.7, CH	113.5, CH
**13**	136.7, C	138.3, C	138.1, C
**14**	39.5, CH_2	41.1, CH_2	41.0, CH_2
**15**	26.4, CH	28.0, CH	27.8, CH
**16**	109.0, C	110.6, C	110.4, C
**17**	152.5, CH	154.1, CH	153.8, CH
**18**	119.2, CH_2	120.8, CH_2	120.5, CH_2
**19**	133.4, CH	135.0, CH	134.8, CH
**20**	44.0, CH	45.6, CH	45.4, CH
**21**	96.2, CH	97.8, CH	97.6, CH
**22**	167.8, C	169.4, C	169.1, C
**1'**	98.6, CH	100.0, CH	100.0, CH
**2'**	73.2, CH	74.8, CH	74.6, CH
**3'**	76.5, CH	78.1, CH	78.0, CH
**4'**	70.1, CH	71.7, CH	71.6, CH
**5'**	77.0, CH	78.6, CH	78.4, CH
**6'**	61.3, CH_2	62.9, CH_2	62.8, CH_2
**N-Me**	25.2, CH_3	26.8, CH_3	26.6, CH_3
**COOMe**	50.4, CH_3	52.0, CH_3	51.7, CH_3

a) the methanol-d4 peak was at 47.4 ppm on the provided ^{13}C NMR of natural ophiorrhine G[19]

Comparison of the optical rotation of natural and synthetic ophiorrhine G

Natural product **Reported**[19] **$[\alpha]^{27}_D$**	*Our Synthetic product* **Measured $[\alpha]^{27}_D$**
− 260.0 (c 0.05, MeOH)	− 105.0 (c 0.4, MeOH)

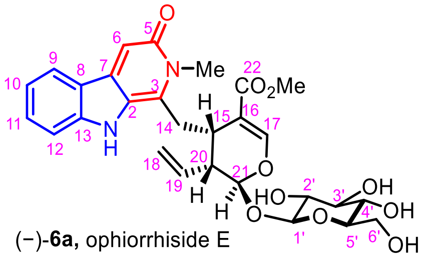

(−)-**6a**, ophiorrhiside E

Comparison of the ^{1}H NMR chemical shifts (δ_{H}) of natural and synthetic Ophiorrhiside E [1]

**Position**	***Natural product*** **Reported δ^{1}H (ppm)**[20,a] (Methanol-d4, 500 MHz)	***Our Synthetic product*** **δ^{1}H (ppm)** vs Methanol-d4 at 3.31 ppm (Methanol-d4, 400 MHz)
**6**	7.01, s	7.05, s
**9**	7.93, br d (7.8)	7.95, d (8.0)
**10**	7.07, dd (7.8, 7.8)	7.09, t (8.0)
**11**	7.47, ddd (7.8, 7.8, 1.2)	7.49, t (8.0, 8.0)
**12**	7.31, d (7.8)	7.33, d (8.0)
**14a**	3.40 – 3.35, m (overlap)	3.45 – 3.28, m (overlap)
**14a**	3.30 – 3.15, m (overlap)	3.45 – 3.28, m (overlap)
**15**	3.40 – 3.35, m (overlap)	3.42 – 3.33, m (overlap)
**17**	7.44, s	7.45, s
**18a**	5.38, d (17.5)	5.39, d (17.5)
**18b**	5.30, d (10.7)	5.31, d (10.4)
**19**	5.99, ddd (17.5, 10.7, 8.5)	6.00, ddd (17.5, 10.7, 7.7)
**20**	2.74, m	2.79 – 2.72, m
**21**	6.02, d (9.5)	6.02, d (9.2)
**1’**		4.85, d (8.0)
**2’**	3.30 – 3.15, m (overlap)	3.29 – 3.20, m (overlap)
**3’**	3.40 – 3.35, m (overlap)	3.35 – 3.30, m (overlap)
**4’**	3.30 – 3.15, m (overlap)	3.31 – 3.26, m (overlap)
**5’**	3.40 – 3.35, m (overlap)	3.46 – 3.39, m (overlap)
**6’a**	4.01, dd (11.8, 2.1)	4.00, dd (11.7, 2.0)
**6’b**	3.70, dd (11.8, 6.9)	3.71, dd (11.7, 6.5)
**N-Me**	3.79, s	3.82, s
**COOMe**	3.07, s	3.08, s

a) No reference for the chemical shifts (δ^{1}H) or copy of the ^{1}H NMR spectra were provided.[20]

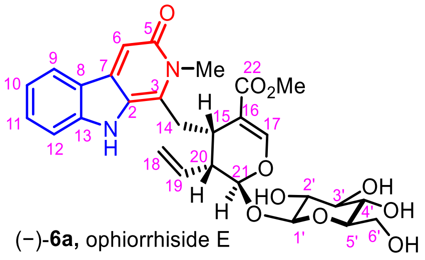

(−)-**6a**, ophiorrhiside E

Comparison of the ^{13}C NMR chemical shifts ($\delta_{^{13}C}$) of natural and synthetic ophiorrhiside E

**Position**	***Natural product*** **Reported $\delta_{^{13}C}$ (ppm)**[20,a] (Methanol-d4, 125 MHz)	***Our Synthetic product*** **$\delta_{^{13}C}$ (ppm)** vs Methanol-d4 at 49.0 ppm (Methanol-d4, 100 MHz)
**2**	129.2, C	129.4, C
**3**	131.5, C	131.7, C
**5**	163.2, C	162.8, C
**6**	103.1, CH	102.9, CH
**7**	139.2, C	139.1, C
**8**	121.3, C	121.3, C
**9**	123.7, CH	123.7, CH
**10**	120.1, CH	120.2, CH
**11**	131.7, CH	131.8, CH
**12**	112.2, CH	112.3, CH
**13**	146.7, C	146.7, C
**14**	32.4, CH_2	32.4, CH_2
**15**	35.7, CH	35.7, CH
**16**	109.8, C	109.8, C
**17**	154.7, CH	154.7, CH
**18**	119.5, CH_2	119.5, CH_2
**19**	135.8, CH	135.8, CH
**20**	45.2, CH	45.2, CH
**21**	97.0, CH	97.0, CH
**22**	168.4, C	168.4, C
**1'**	100.5, CH	100.5, CH
**2'**	74.7, CH	74.7, CH
**3'**	78.0, CH	78.1, CH
**4'**	71.7, CH	71.7, CH
**5'**	78.8, CH	78.8, CH
**6'**	63.0, CH_2	63.1, CH_2
**N-Me**	33.4, CH_3	33.6, CH_3
**COOMe**	51.6, CH_3	51.5, CH_3

a) No reference for the chemical shifts ($\delta_{^{13}C}$) or copy of the ^{1}H NMR spectra were provided.[20]

Comparison of the optical rotation of natural and synthetic ophiorrhiside E

Natural product **Reported**[20] **$[\alpha]^{27}_D$**	***Our Synthetic product*** **Measured $[\alpha]^{25}_D$**
− 223.3 (c 0.09, MeOH)	− 265.0 (c 0.6, MeOH)

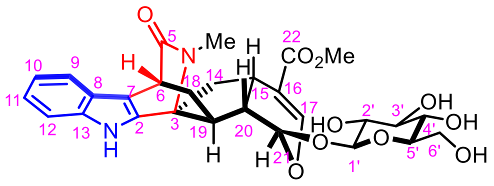

(−)-**1a**, ophiorrhine A

Comparison of the ^{1}H NMR chemical shifts (δ_H) of natural and synthetic ophiorrhine A

**Position**	***Natural product*** **Reported**[21] **$\delta_{^1H}$ (ppm)** (Methanol-d4, 600 MHz)	***Natural product*** **Recalibrated**[a] **$\delta_{^1H}$ (ppm)** vs Methanol-d4 at 3.31 ppm (Methanol-d4, 600 MHz)	***Our Synthetic product*** **$\delta_{^1H}$ (ppm)** vs Methanol-d4 at 3.31 ppm (Methanol-d4, 400 MHz)
**6**	4.15, t (2.6)	4.15, t (2.6)	4.15, t (2.3)
**9**	7.47, d (7.8)	7.47, d (7.8)	7.47, d (7.3)
**10**	7.01, t (7.8)	7.01, t (7.8)	7.01, d (7.3)
**11**	7.04, t (7.8)	7.04, t (7.8)	7.05, d (7.3)
**12**	7.32, d (7.8)	7.32, d (7.8)	7.33, d (7.3)
**14a**	3.25, m	3.25, m	3.25 – 3.32, m
**14b**	2.22, m	2.22, m	2.28 – 2.22, m
**15**	3.33, m	3.33, m	3.34-3.32, m
**17**	7.37, s	7.37, s	7.37, s
**18a,b**	1.85, ddd (6.8, 2.5, 2.1)	1.85, ddd (6.8, 2.5, 2.1)	1.88 – 1.84, m
**19**	1.91, m	1.91, m	1.96 – 1.88, m
**20**	2.20, m	2.20, m	2.25 – 2.19, m
**21**	5.52, d (1.7)	5.52, d (1.7)	5.52, d (1.5)
**1’**	4.59, d (8.0)	4.59, d (8.0)	4.59, d (7.9)
**2’**	3.16, dd (9.2, 8.0)	3.16, dd (9.2, 8.0)	3.17, dd (9.1, 7.9)
**3’**	3.35, m	3.35, m	3.37 – 3.31, m
**4’**	3.25, m	3.25, m	3.30 – 3.24, m
**5’**	3.28, m	3.28, m	3.28 – 3.30, m
**6a’**	3.88, dd (11.8, 1.8)	3.88, dd (11.8, 1.8)	3.88, dd (11.7, 1.7)
**6b’**	3.65, dd (11.8, 5.7)	3.65, dd (11.8, 5.7)	3.65, dd (11.7, 5.3)
**N-Me**	2.95, s	2.95, s	2.96, s
**COOMe**	3.76, s	3.76, s	3.77, s

a) the methanol-d4 peak was at 3.31 ppm on the provided ^{1}H NMR of natural ophiorrhine A[21]

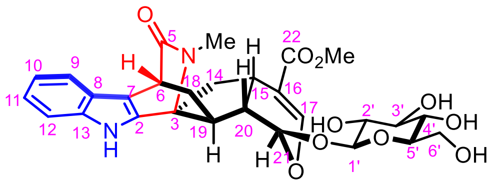

(−)-**1a**, ophiorrhine A

Comparison of the ^{13}C NMR chemical shifts ($\delta_{^{13}C}$) of natural and synthetic ophiorrhine A

**Position**	*Natural product* **Reported $\delta_{^{13}C}$ (ppm)**[21] (Methanol-d4, 150 MHz)	*Natural product* **Recalibrated $\delta_{^{13}C}$ (ppm)**[a] vs Methanol-d4 at 49.0 ppm (Methanol-d4, 150 MHz)	*Our Synthetic product* **$\delta_{^{13}C}$ (ppm)** vs Methanol-d4 at 49.0 ppm (Methanol-d4, 100 MHz)
**2**	142.7, C	144.1, C	144.1, C
**3**	68.3, C	69.7, C	69.8, C
**5**	178.3, C	179.7, C	179.7, C
**6**	41.9, CH	43.3, CH	43.4, CH
**7**	111.1, C	112.5, C	112.5, C
**8**	124.1, C	125.5, C	125.5, C
**9**	117.1, CH	118.5, CH	118.5, CH
**10**	119.3, CH	120.7, CH	120.7, CH
**11**	120.6, CH	122.0, CH	122.0, CH
**12**	111.7, CH	113.1, CH	113.1, CH
**13**	136.5, C	137.9, C	137.9, C
**14**	33.1, CH_2	34.5, CH_2	34.5, CH_2
**15**	31.1, CH	32.5, CH	32.5, CH
**16**	113.3, C	114.7, C	114.7, C
**17**	149.6, CH	151.0, CH	151.0, CH
**18**	26.6, CH_2	28.0, CH_2	28.0, CH_2
**19**	50.8, CH	52.2, CH	52.3, CH
**20**	45.5, CH	46.9, CH	46.9, CH
**21**	94.2, CH	95.6, CH C	95.7, CH
**22**	167.7, C	169.1,	169.1, C
**1’**	98.5, CH	99.9, CH	100.0, CH
**2’**	73.2, CH	74.6, CH	74.6, CH
**3’**	76.5, CH	77.9, CH CH	77.9, CH
**4’**	70.1, CH	71.5, CH	71.5, CH
**5’**	76.9, CH	78.3, CH	78.3, CH
**6’**	61.2, CH_2	62.6, CH_2	62.6, CH_2
**N-Me**	27.4, CH_3	28.6, CH_3	28.7, CH_3
**COOMe**	50.5, CH_3	51.9, CH_3	51.9, CH_3

a) the methanol-d4 peak was at 47.6 ppm on the provided ^{13}C NMR of natural ophiorrhine A[21]

Comparison of the optical rotation of natural and synthetic ophiorrhine A

Natural product **Reported**[21] **$[\alpha]^{27}_D$**	*Our Synthetic product* **Measured $[\alpha]^{27}_D$**
− 55.6 (c 0.14, MeOH)	− 68.6 (c 0.35, MeOH)

5. NMR spectra of all compounds

^{1}H NMR (360 MHz, CD_3OD), **28a**

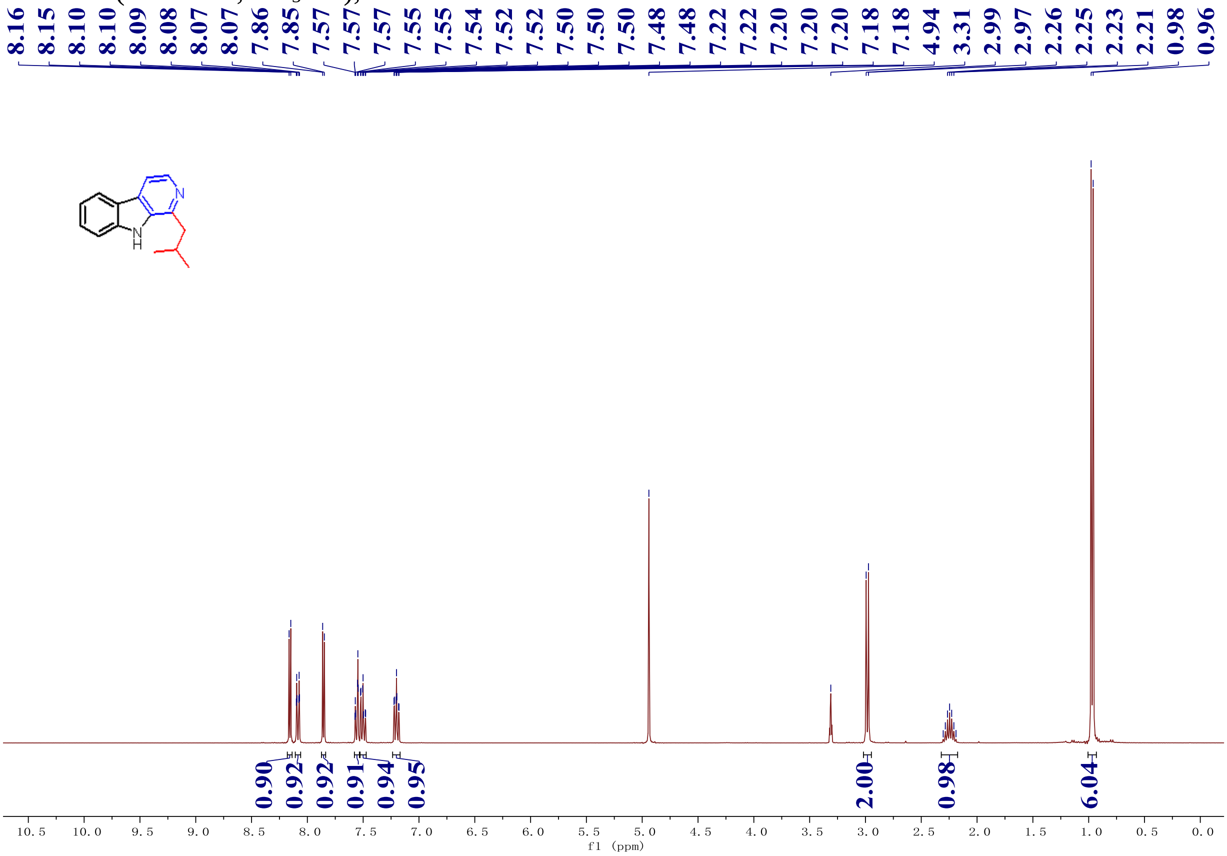

^{13}C NMR (90 MHz, CD_3OD), **28a**

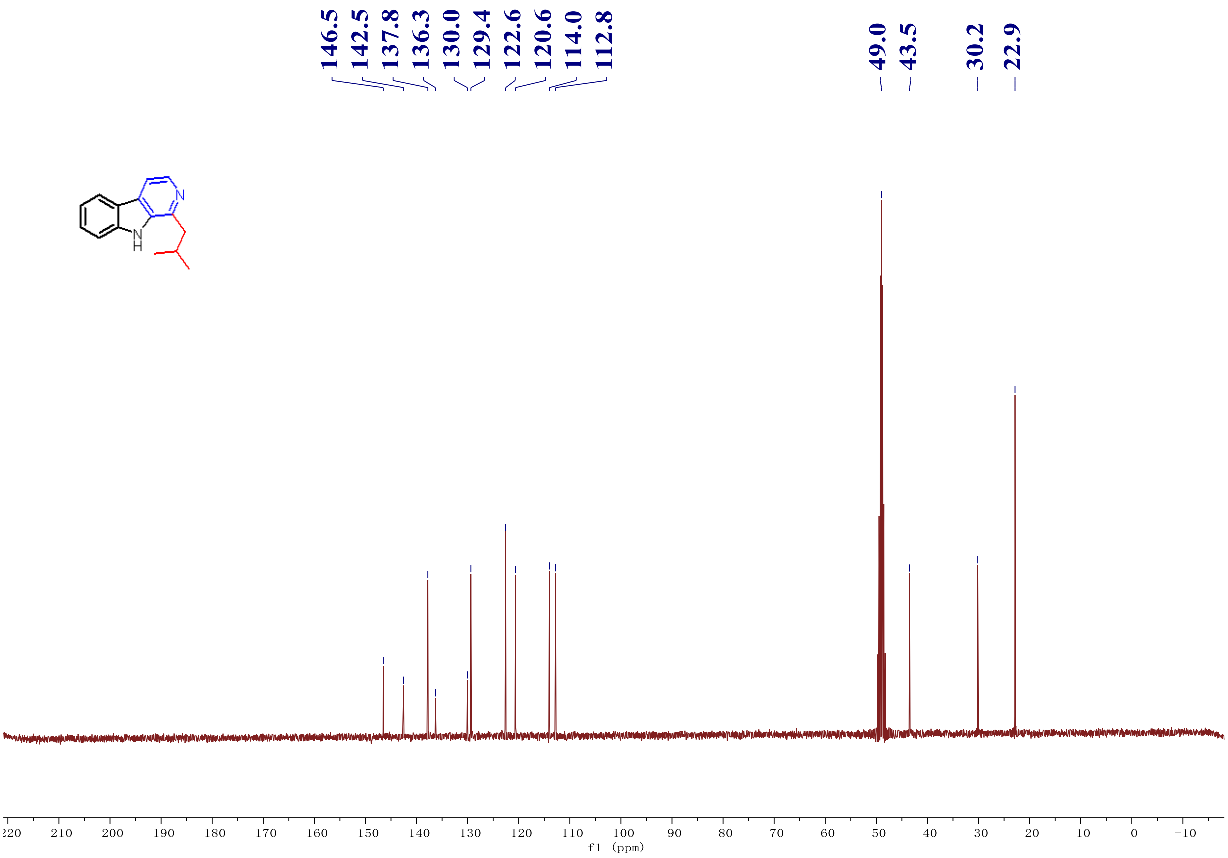

^{1}H NMR (360 MHz, CD_3OD), **29a**

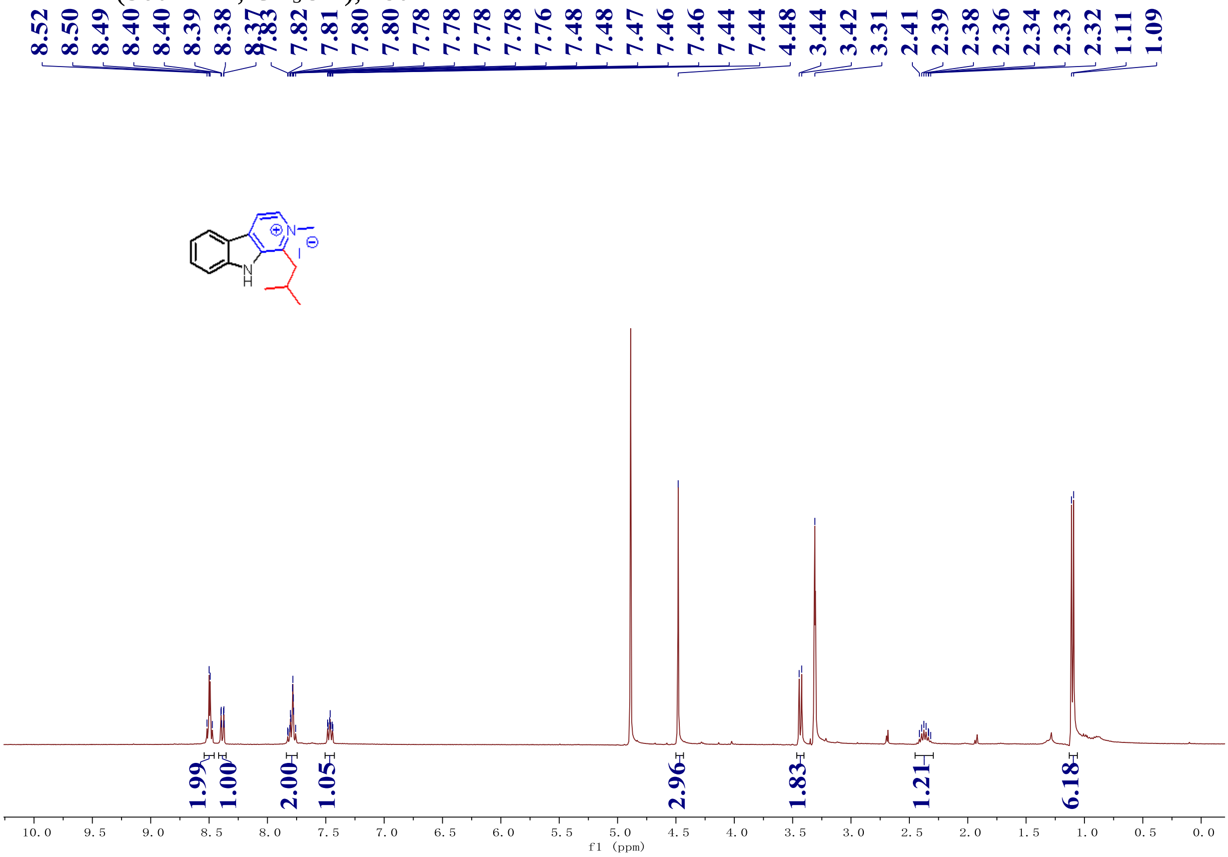

^{13}C NMR (90 MHz, CD_3OD), **29a**

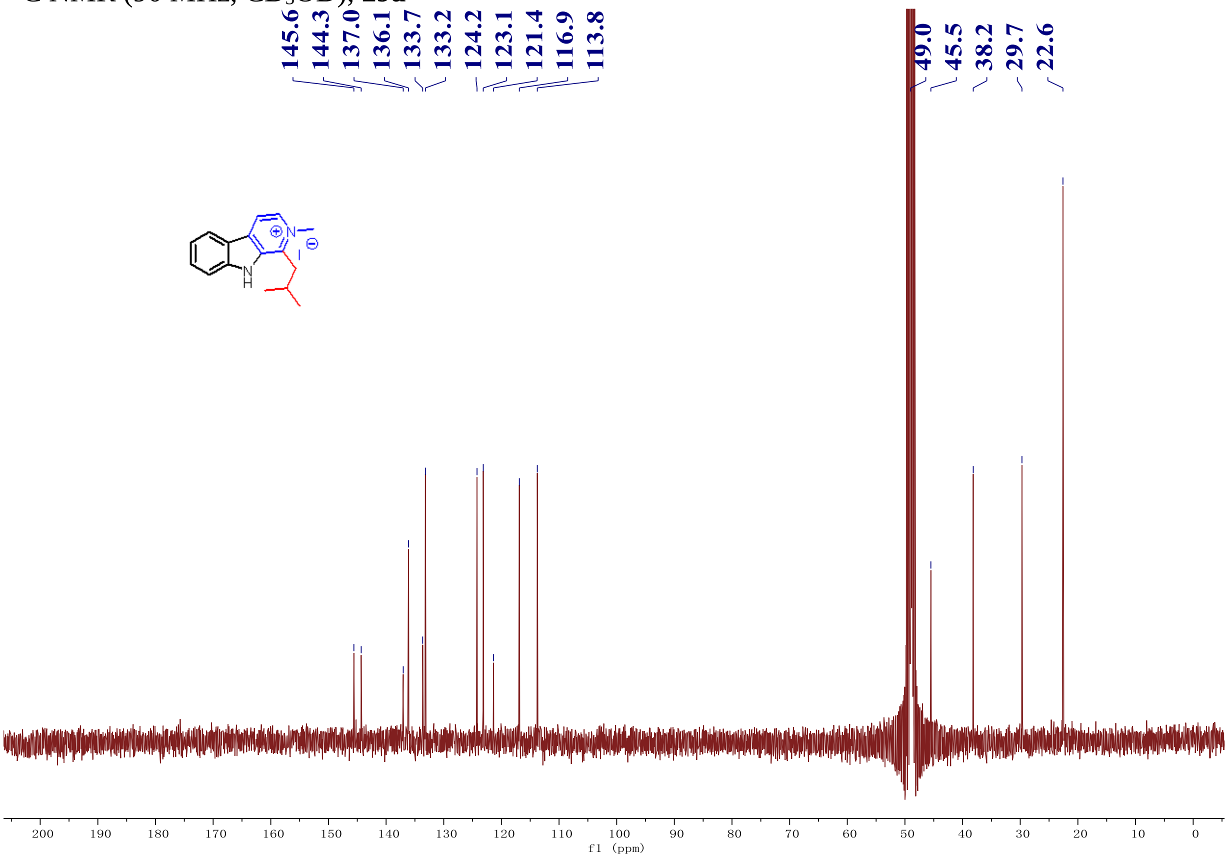

^{1}H NMR (360 MHz, CD_3OD), **31**

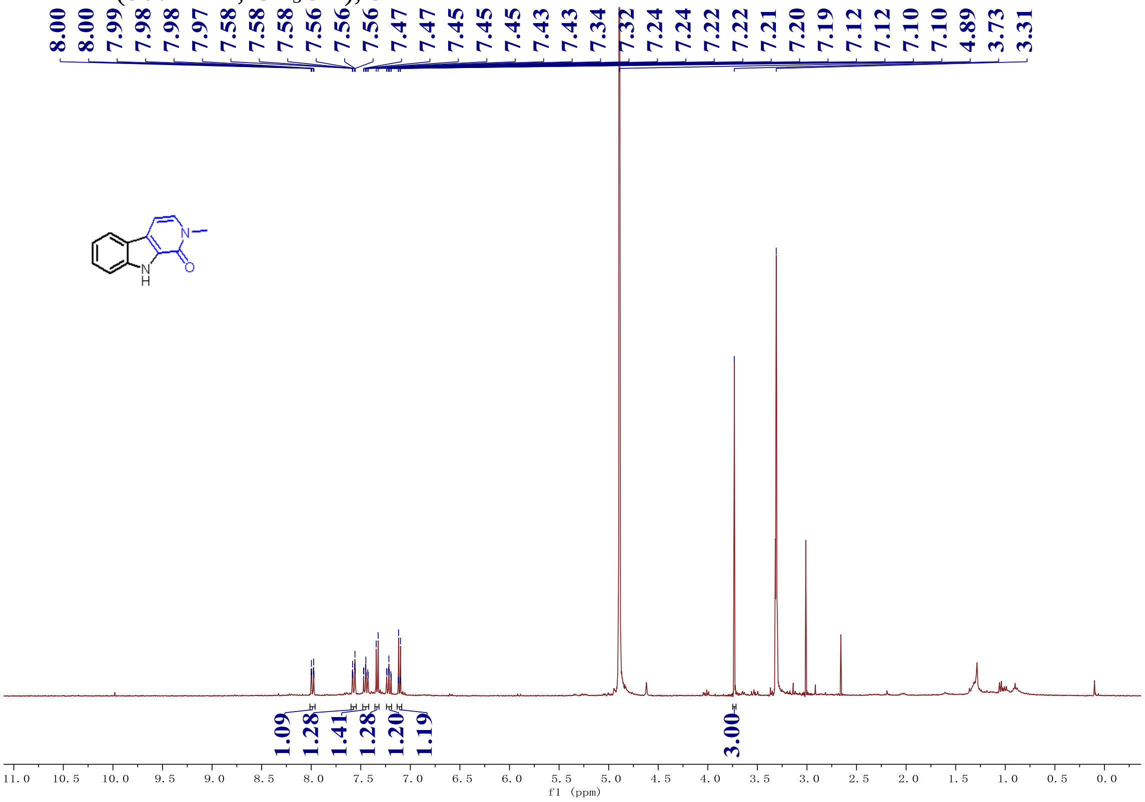

^{13}C NMR (90 MHz, CD_3OD), **31**

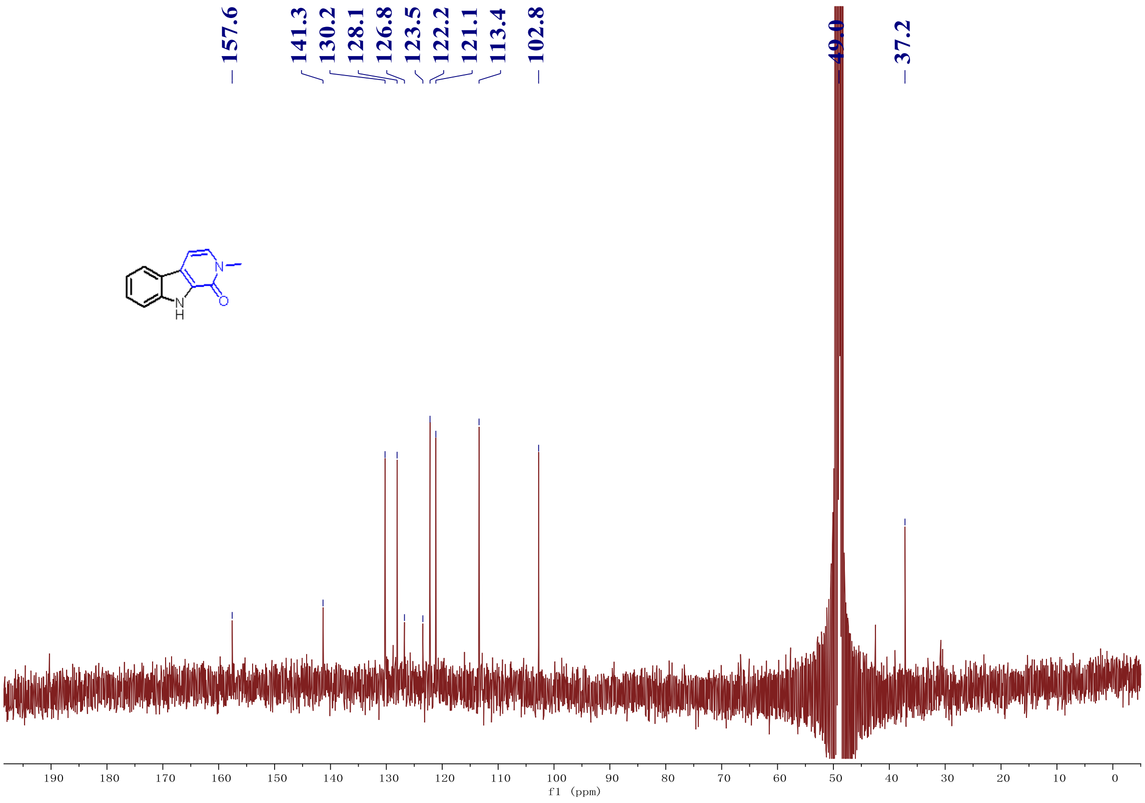

^{1}H NMR (300 MHz, $CDCl_3$), **(±)-28b**

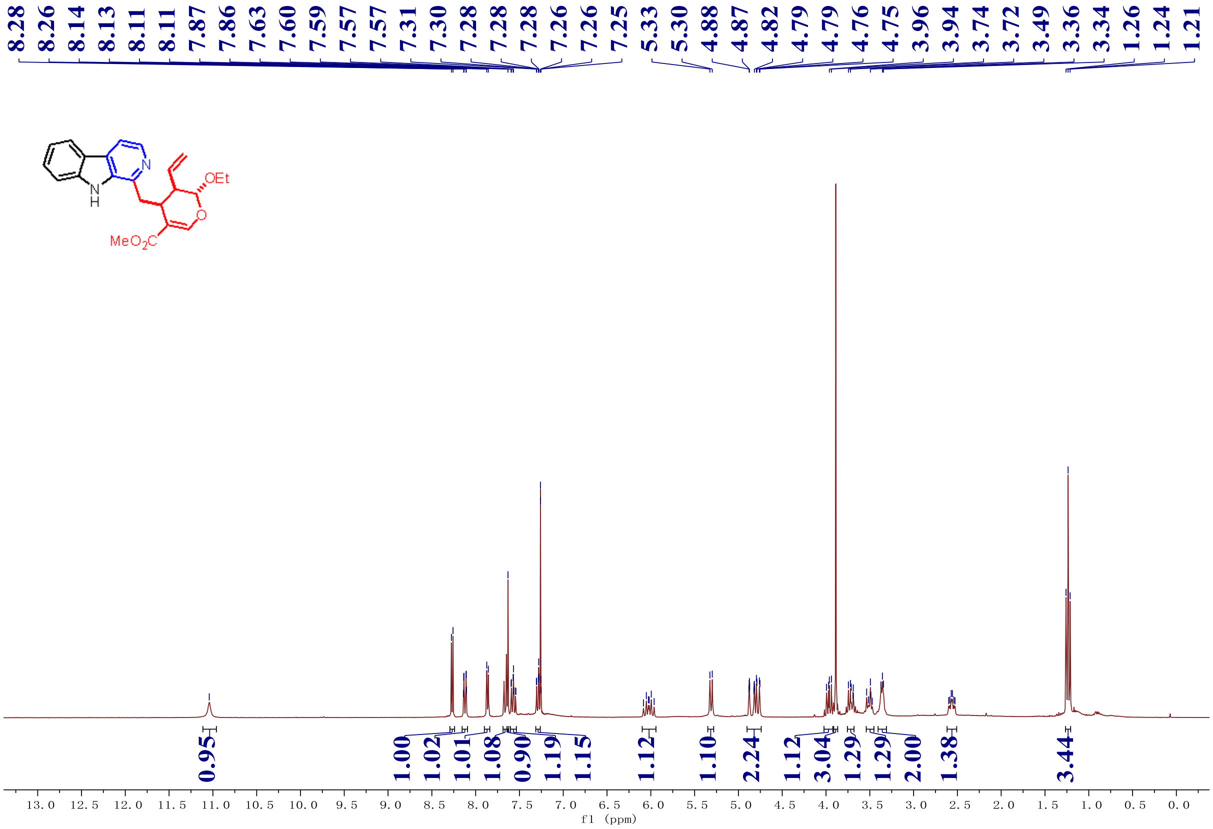

^{13}C NMR (75 MHz, $CDCl_3$), **(±)-28b**

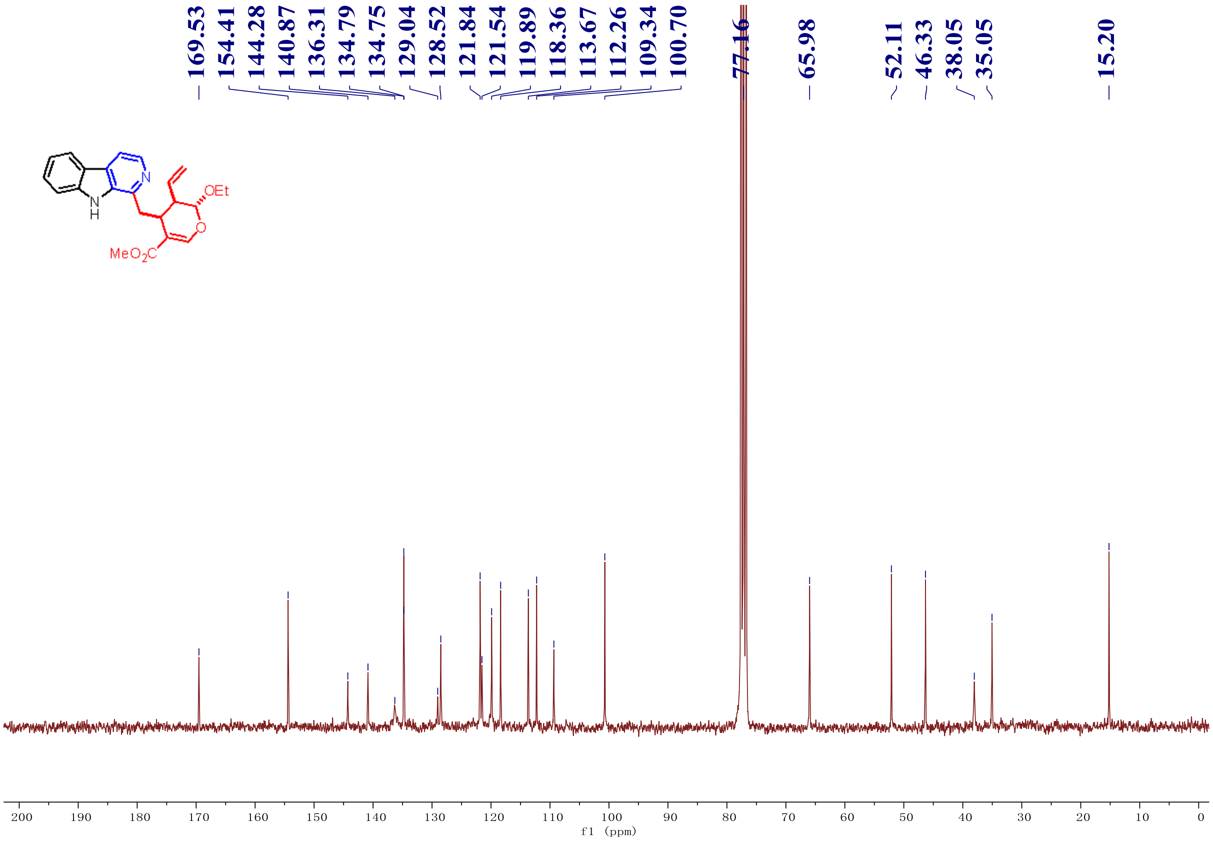

^{1}H NMR (300 MHz, CDCl$_3$), **(±)-29b**

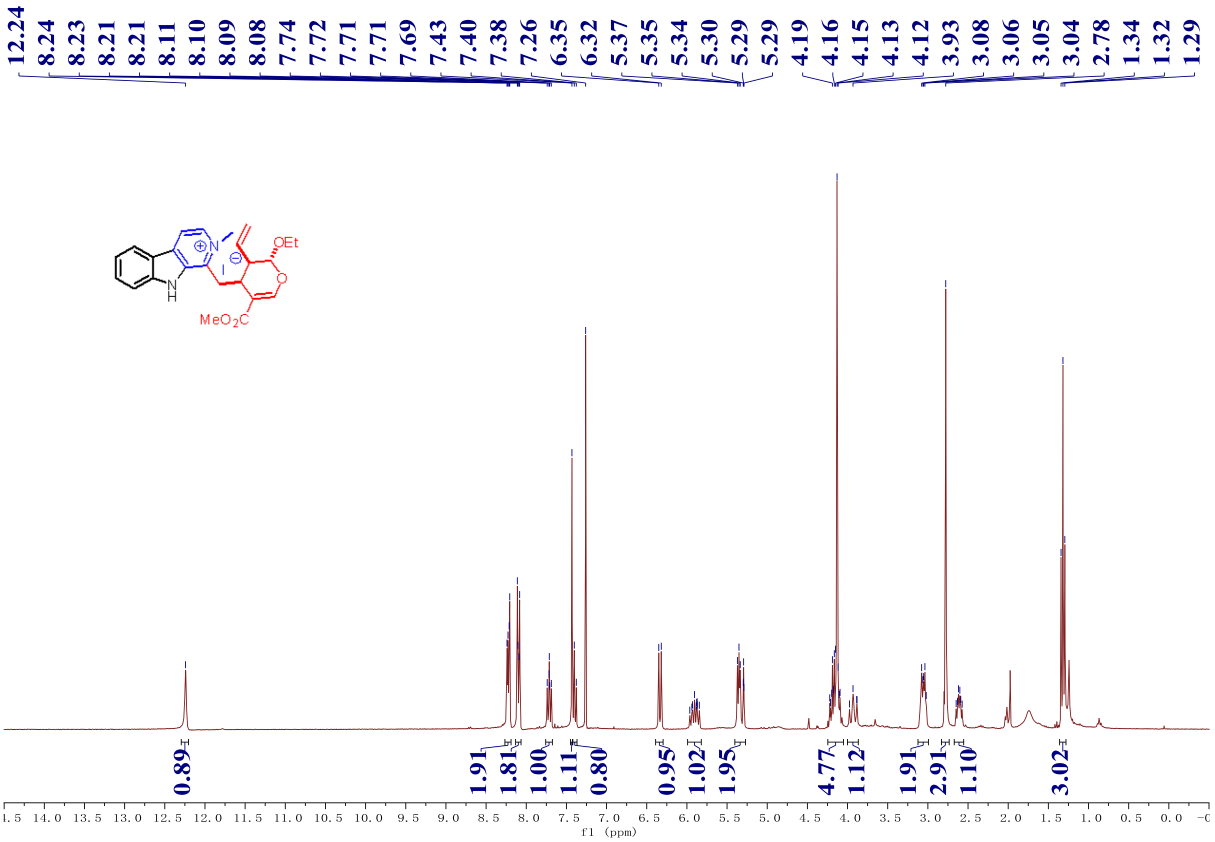

^{13}C NMR (75 MHz, CDCl$_3$), **(±)-29b**

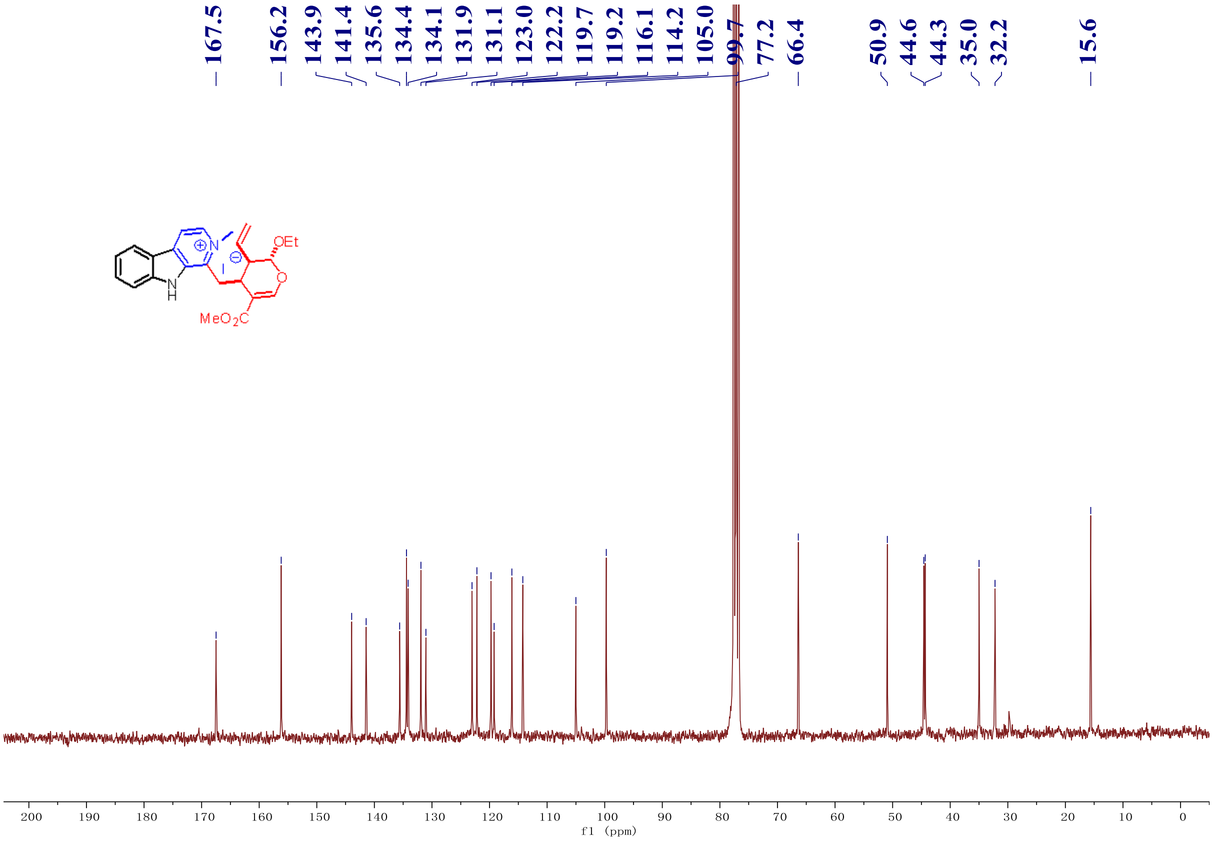

^{1}H NMR (360 MHz, CD_3OD), **33a**

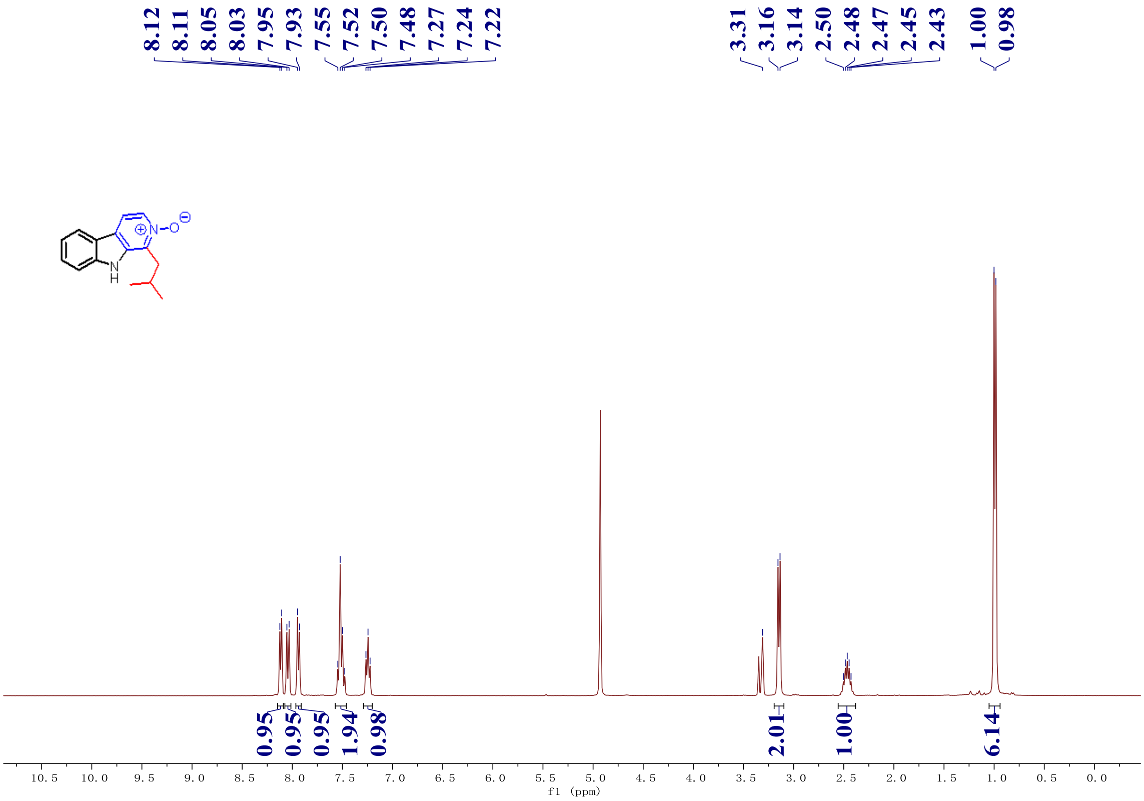

^{13}C NMR (100 MHz, CD_3OD), **33a**

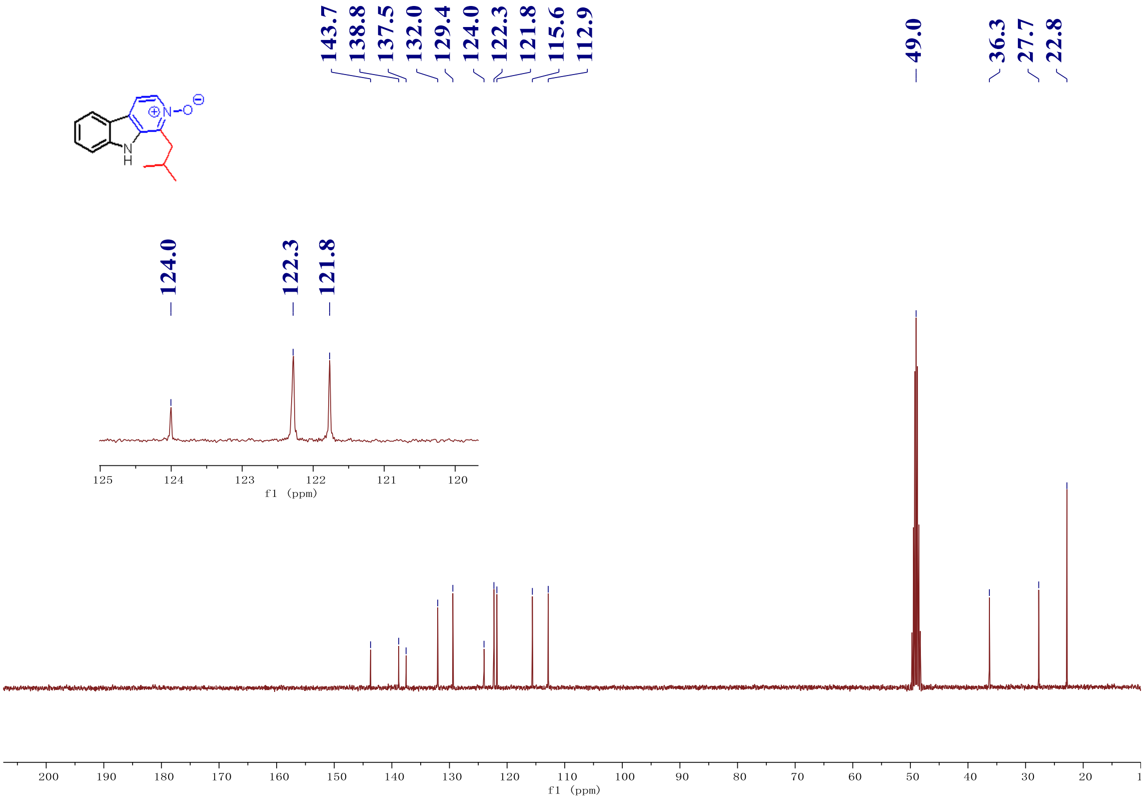

^{1}H NMR (250 MHz, CD_3OD), **(±)-34a**

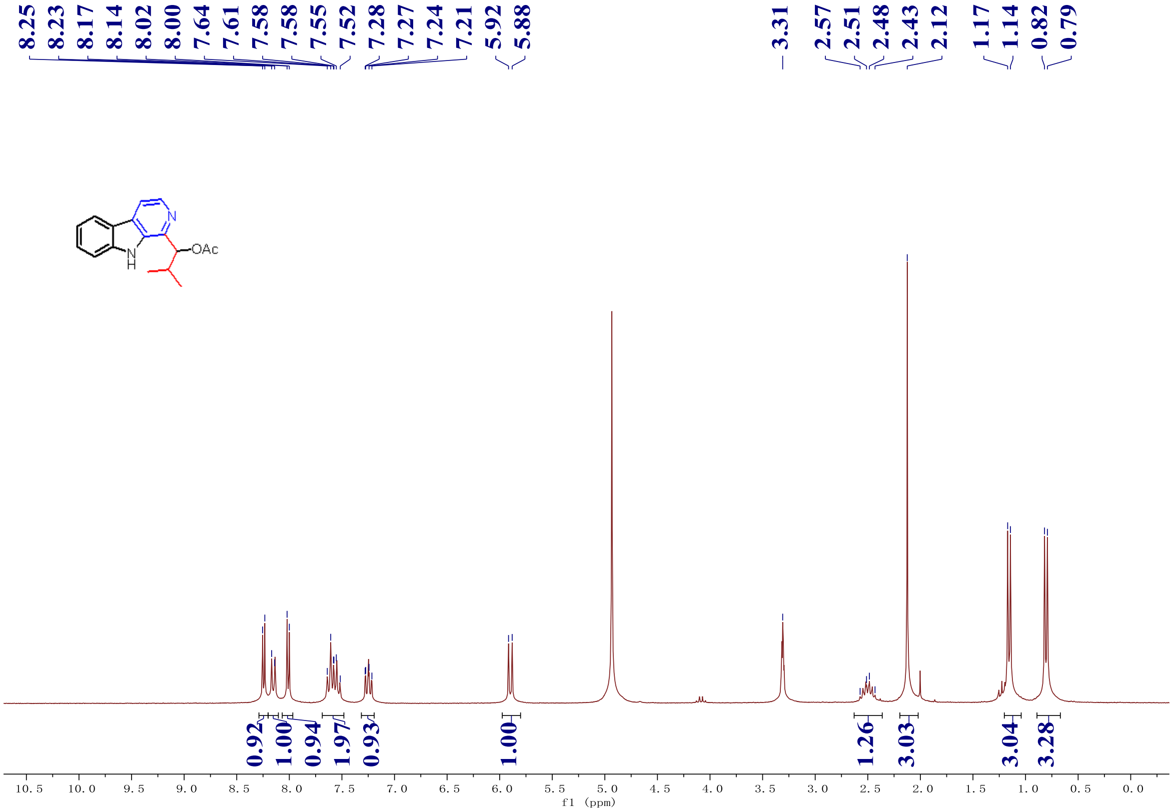

^{13}C NMR (62.5 MHz, CD_3OD), **(±)-34a**

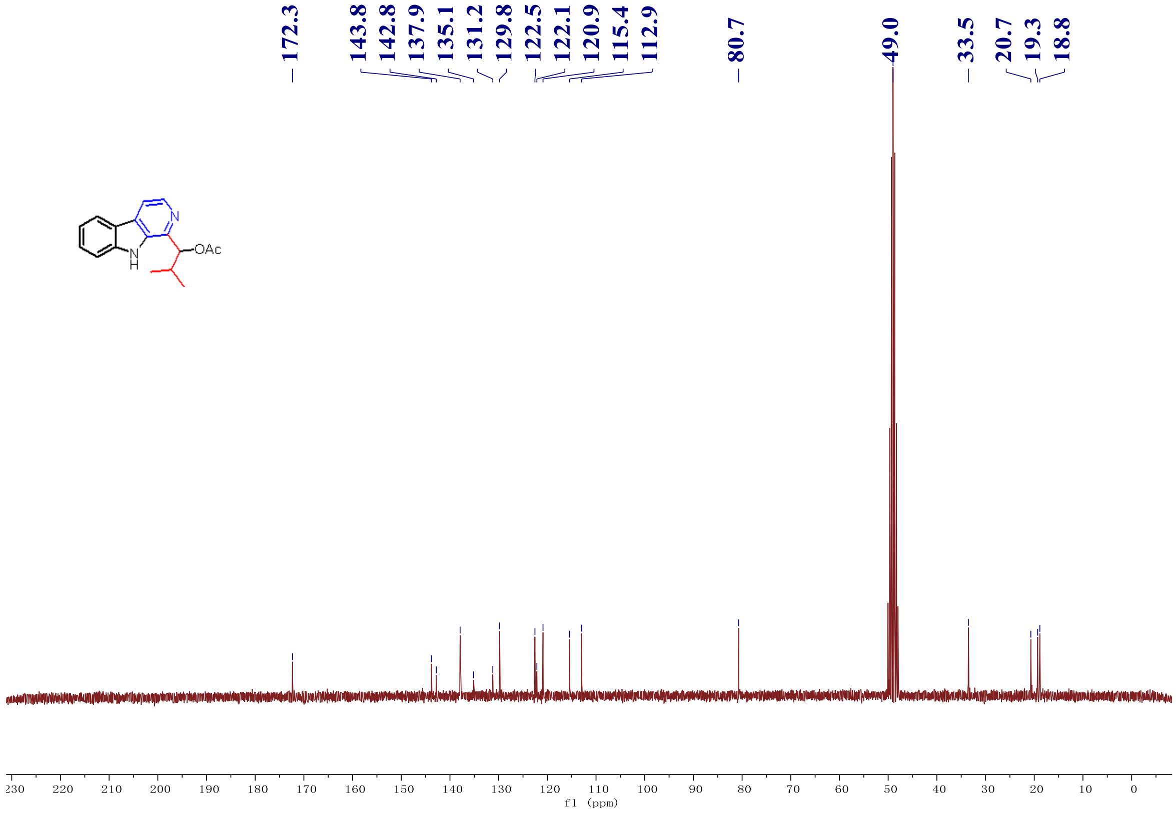

^{1}H NMR (360 MHz, CD_3OD), **(±)-35a**

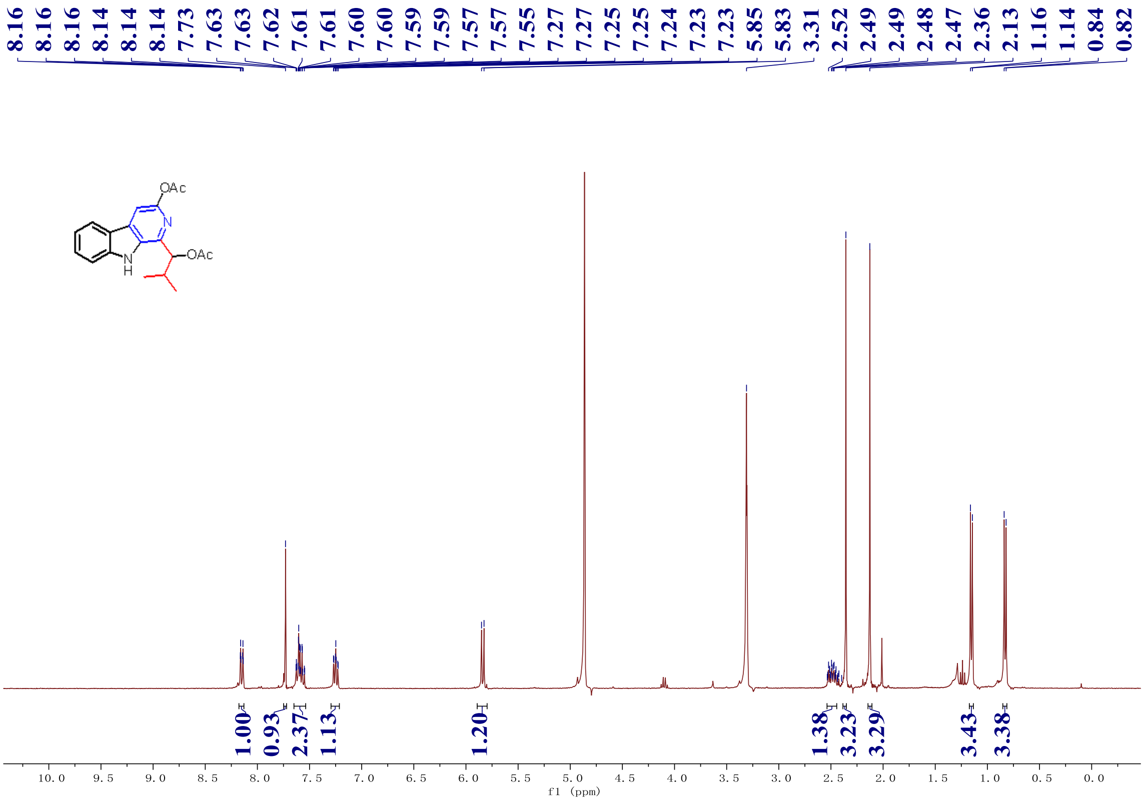

^{13}C NMR (100 MHz, CD_3OD), **(±)-35a**

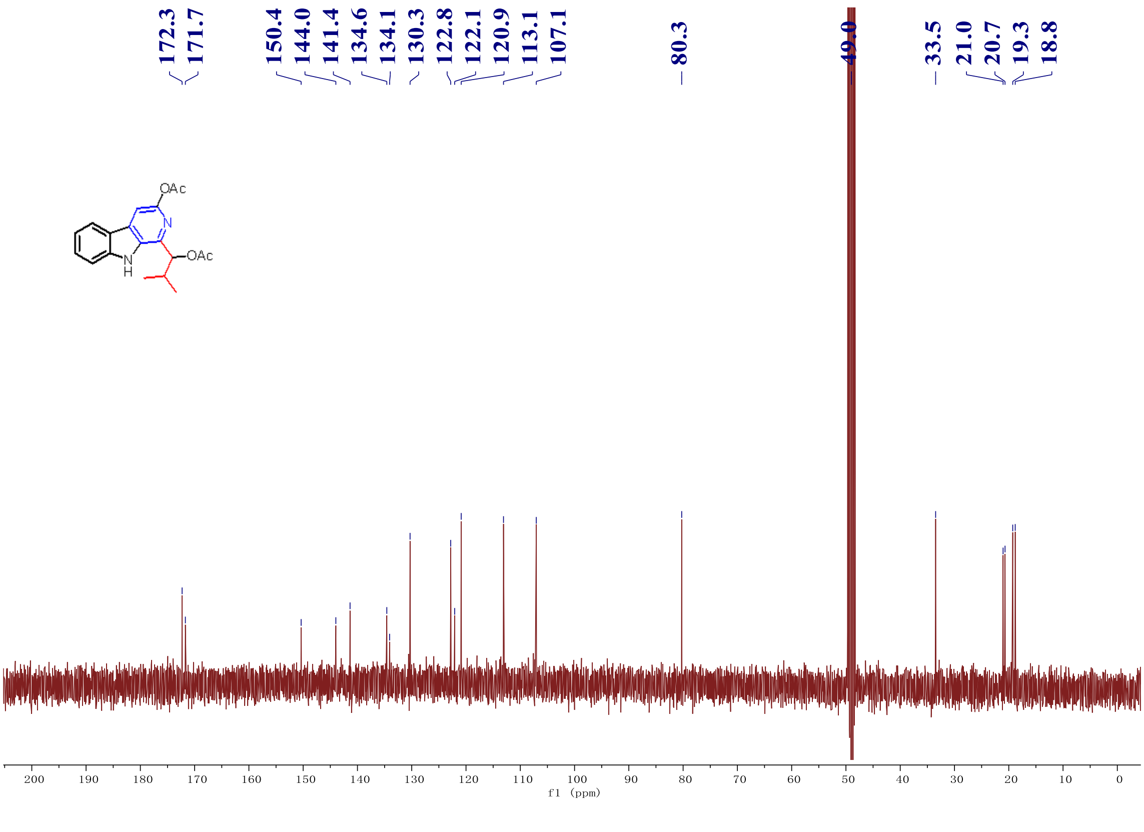

^{1}H NMR (360 MHz, CD_3OD), **(±)-36a**

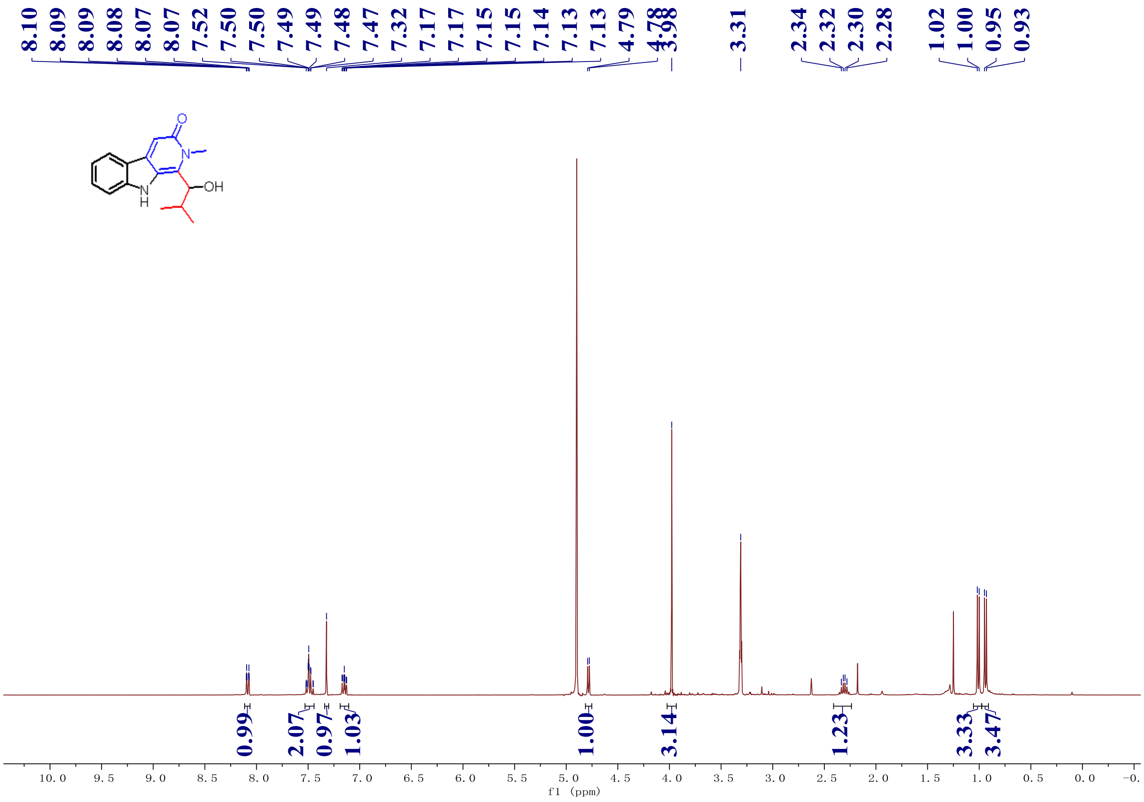

^{13}C NMR (100 MHz, CD_3OD), **(±)-36a**

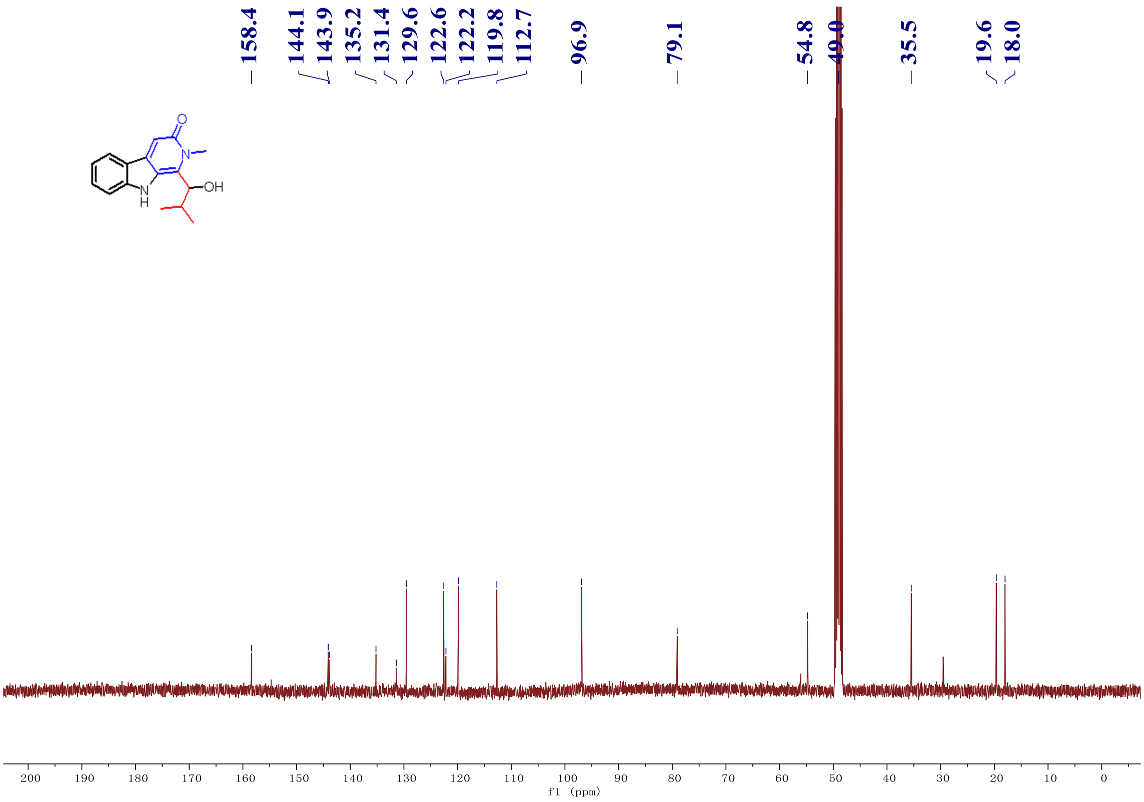

^{1}H NMR (400 MHz, $CDCl_3$), **37a**

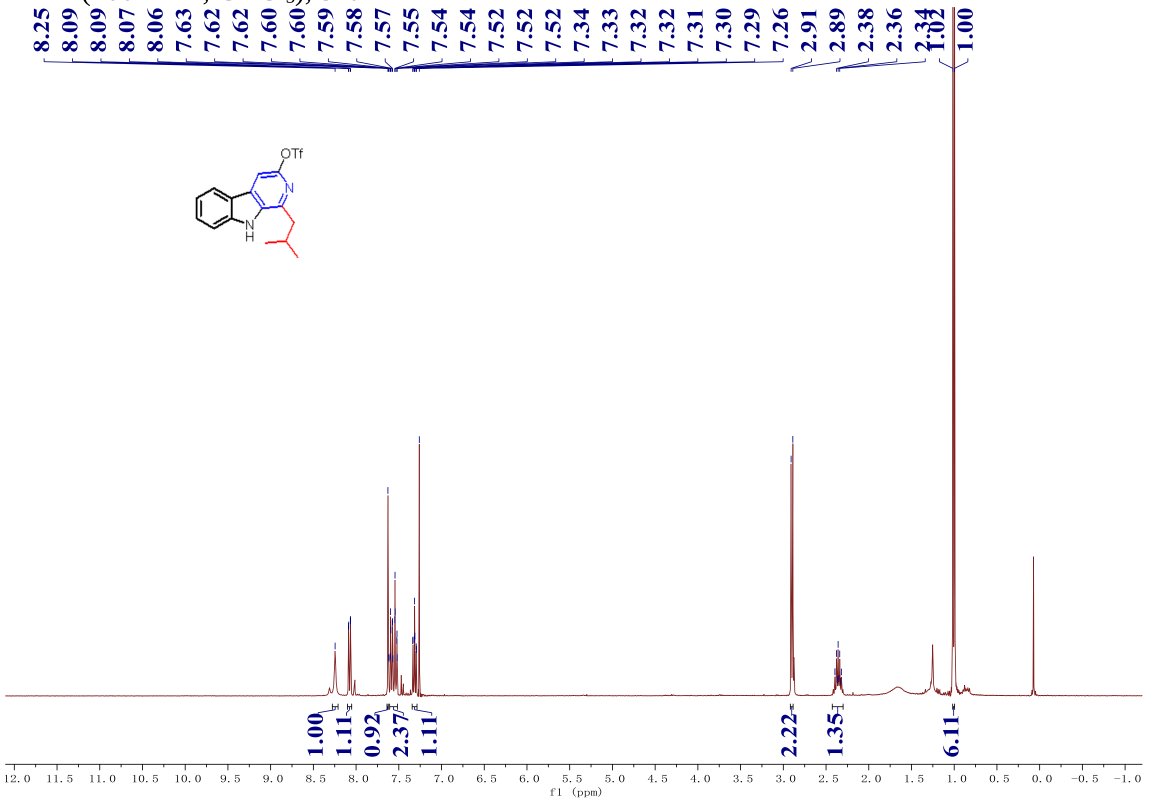

^{13}C NMR (100 MHz, $CDCl_3$), **37a**

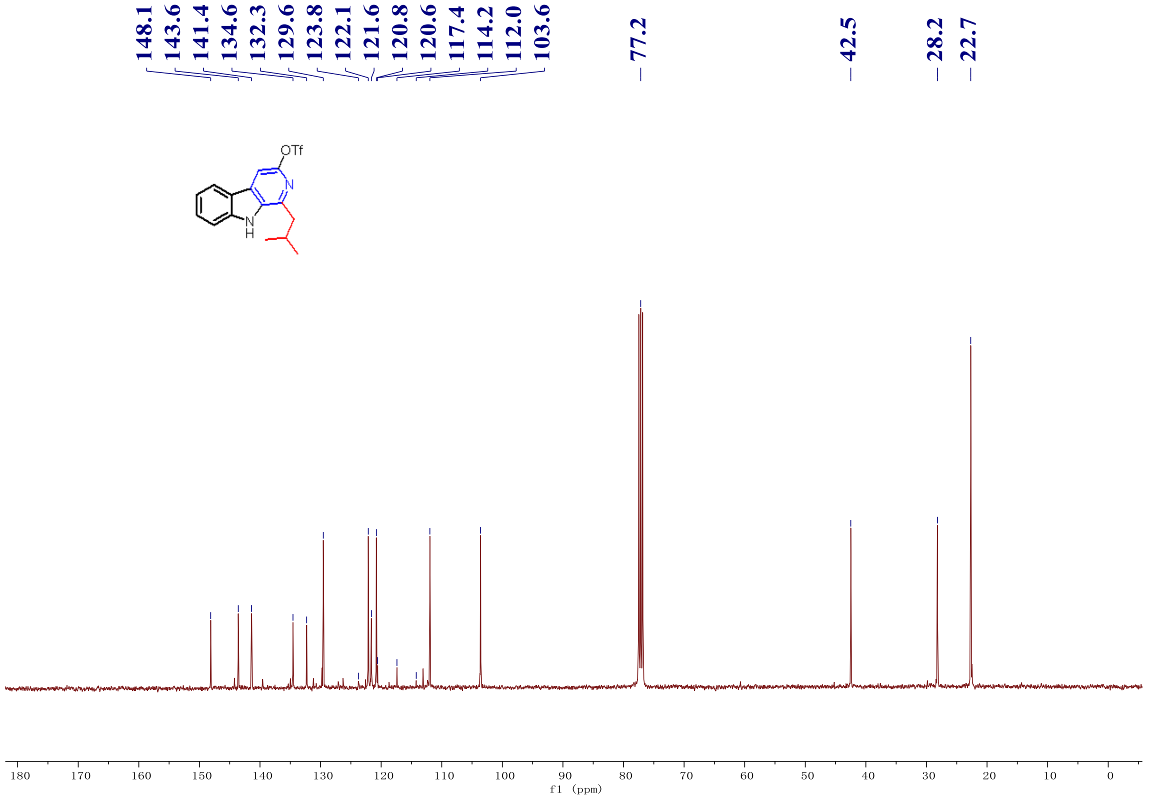

^{19}F NMR (235 MHz, $CDCl_3$), **37a**

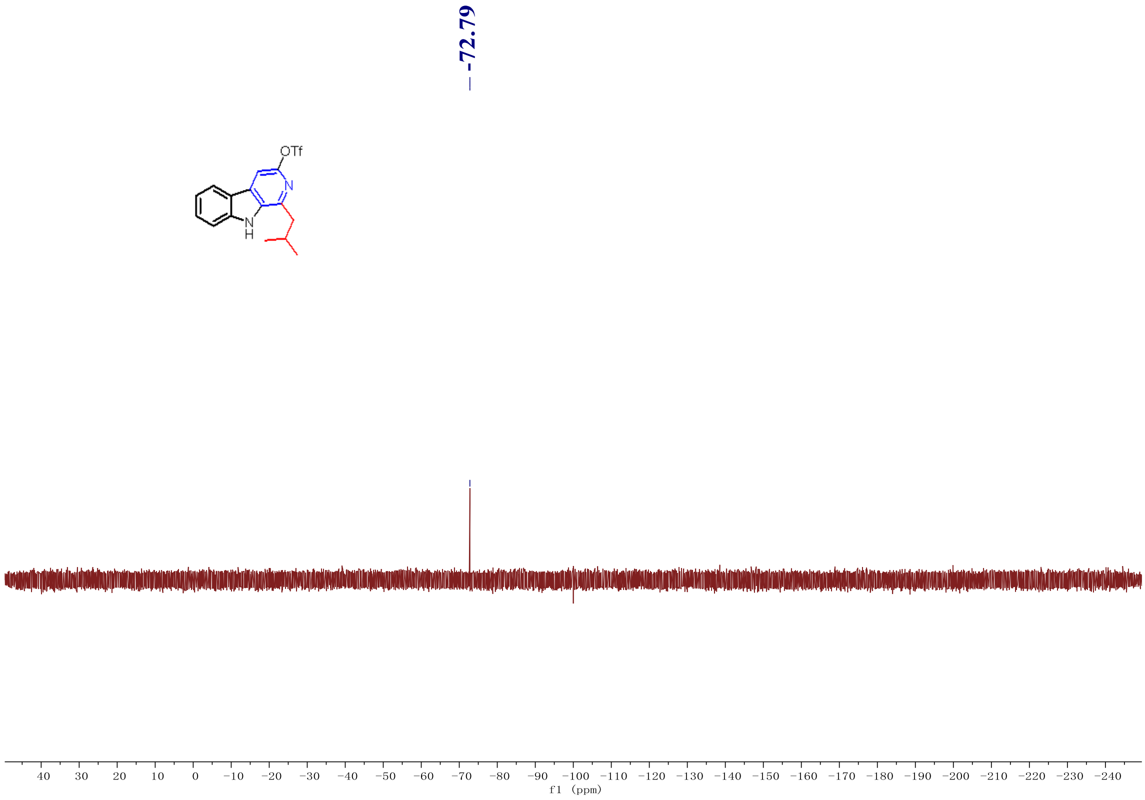

^{1}H NMR (300 MHz, $CDCl_3$), **38a**

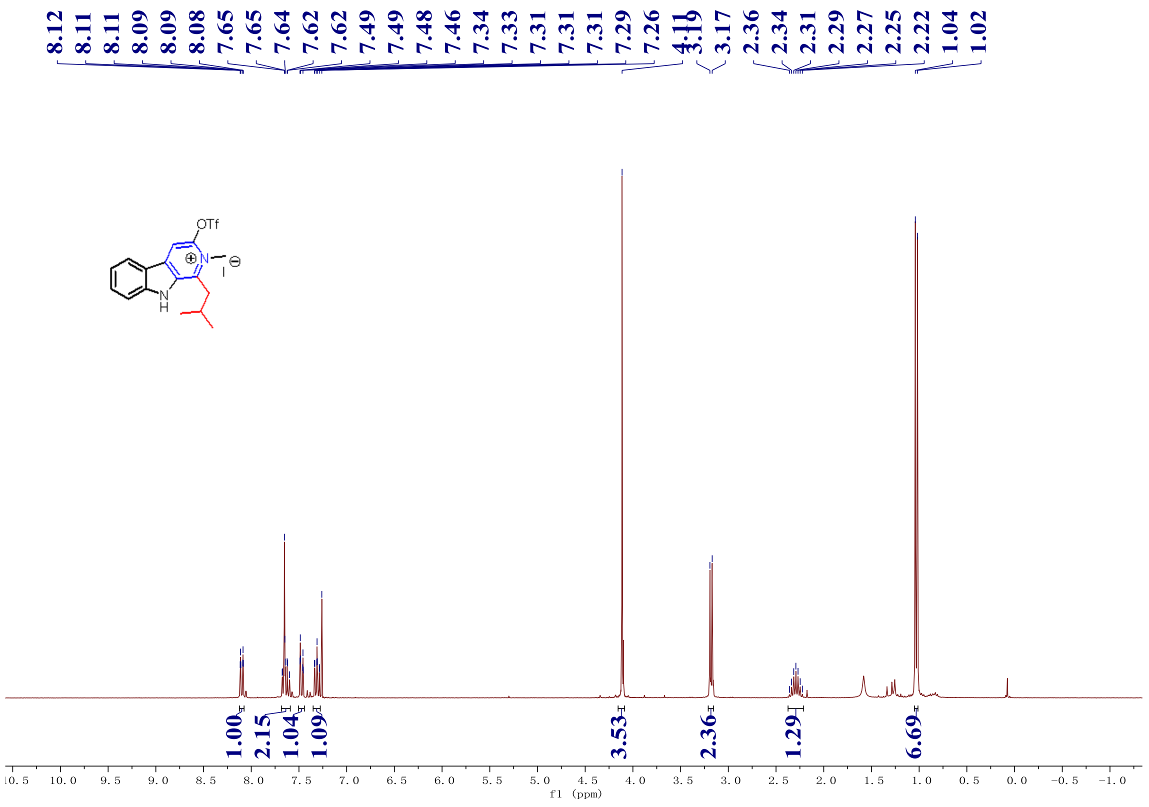

^{13}C NMR (62.5 MHz, $CDCl_3$), **38a**

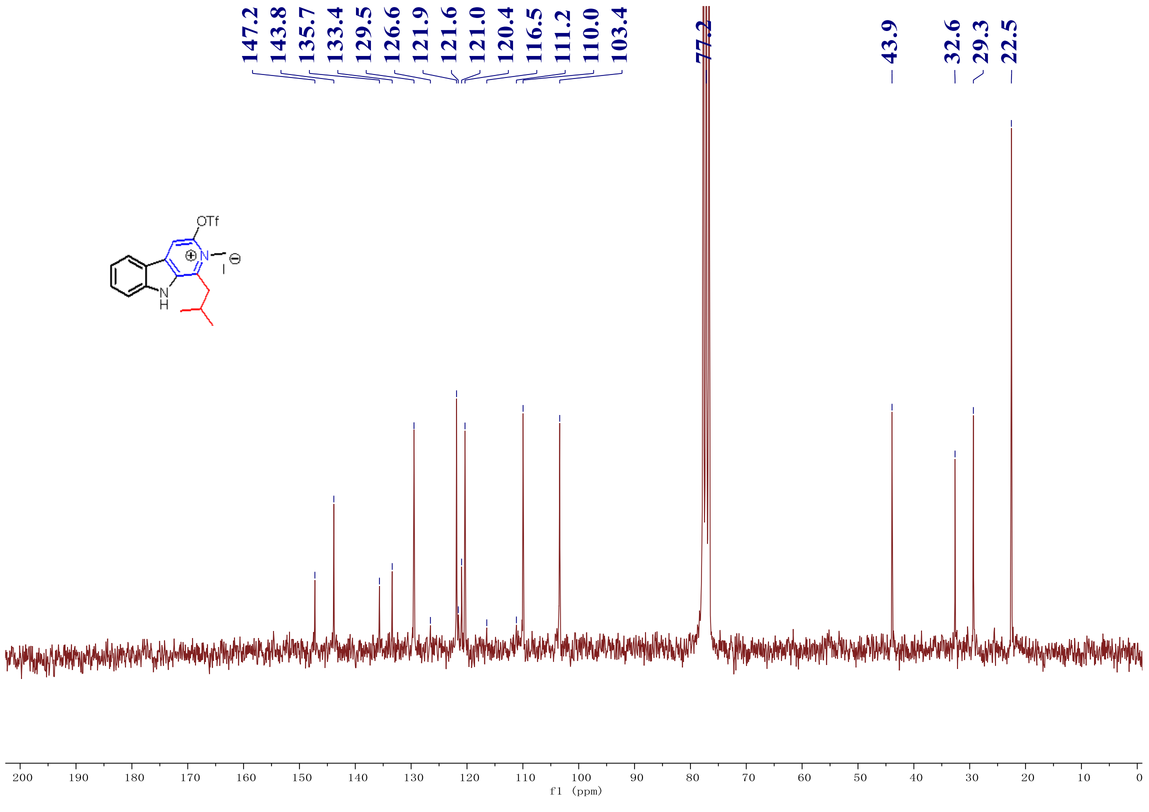

^{19}F NMR (235 MHz, $CDCl_3$), **38a**

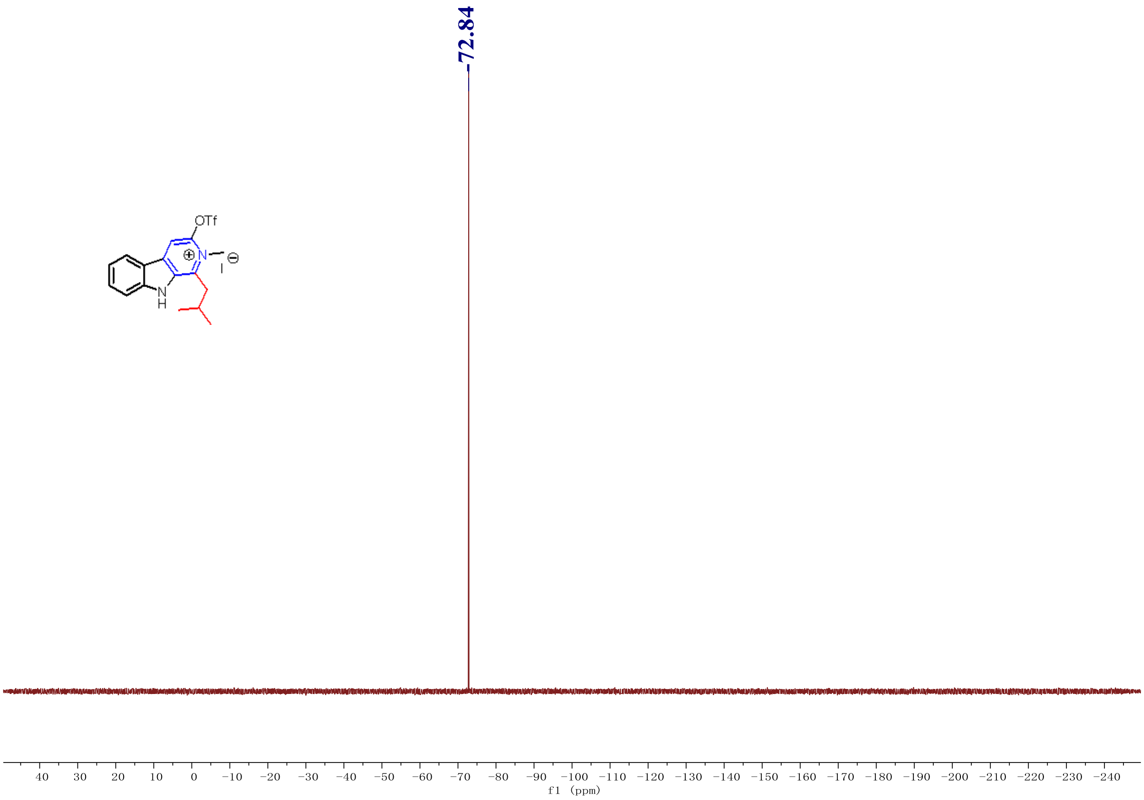

^{1}H NMR (400 MHz, $CDCl_3$), **39a**

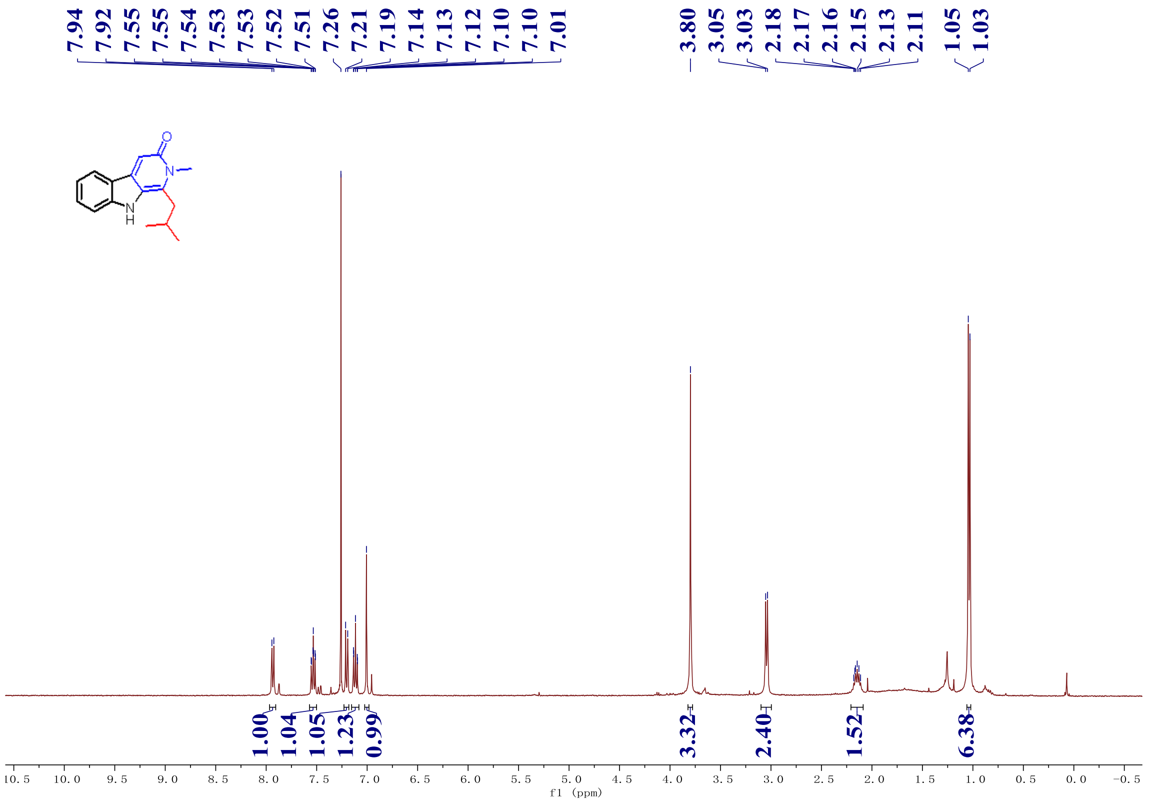

^{13}C NMR (100 MHz, $CDCl_3$), **39a**

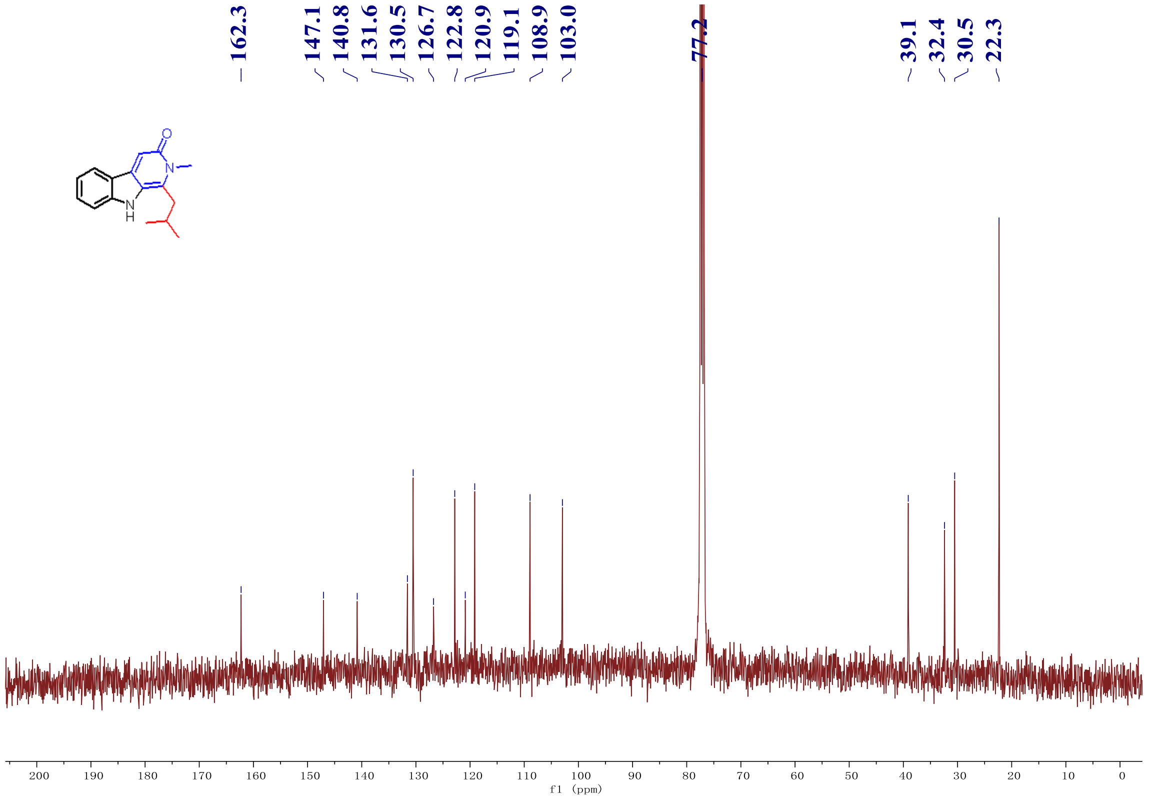

^{1}H NMR (300 MHz, $CDCl_3$), **(±)-33b**

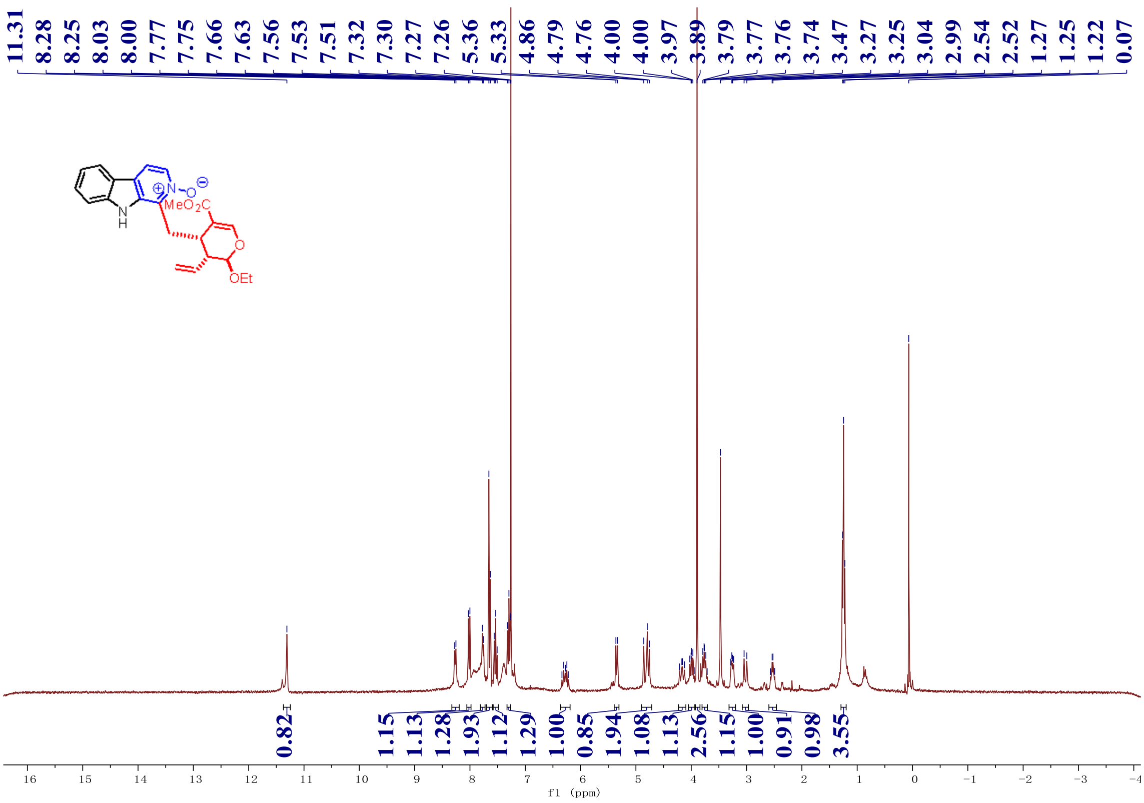

^{13}C NMR (90 MHz, $CDCl_3$), **(±)-33b**

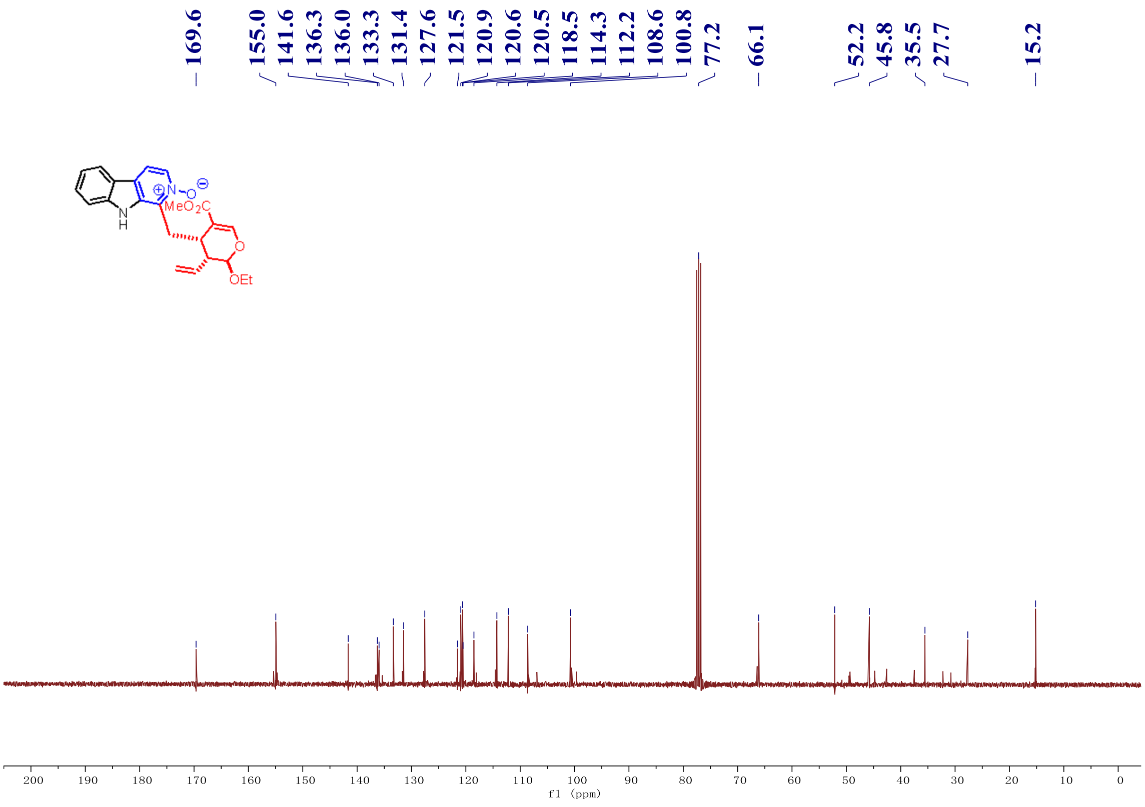

^{1}H NMR (300 MHz, $CDCl_3$), **(±)-37b**

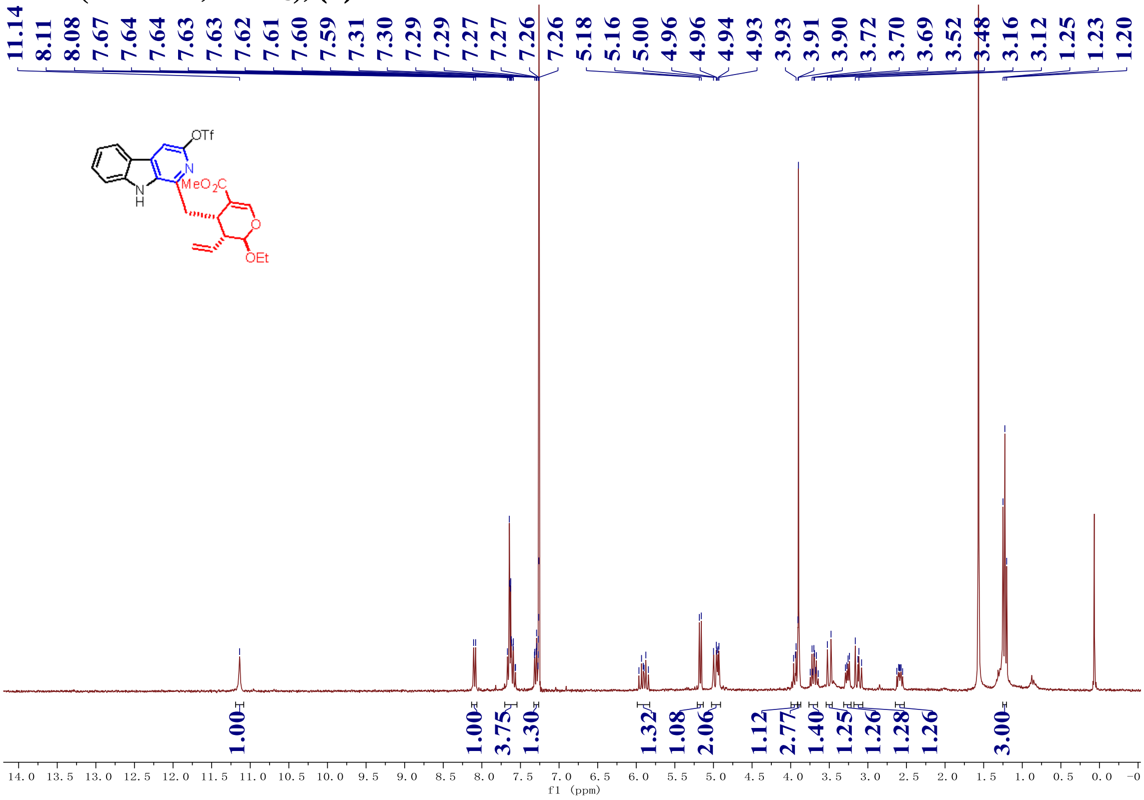

^{13}C NMR (100 MHz, $CDCl_3$), **(±)-37b**

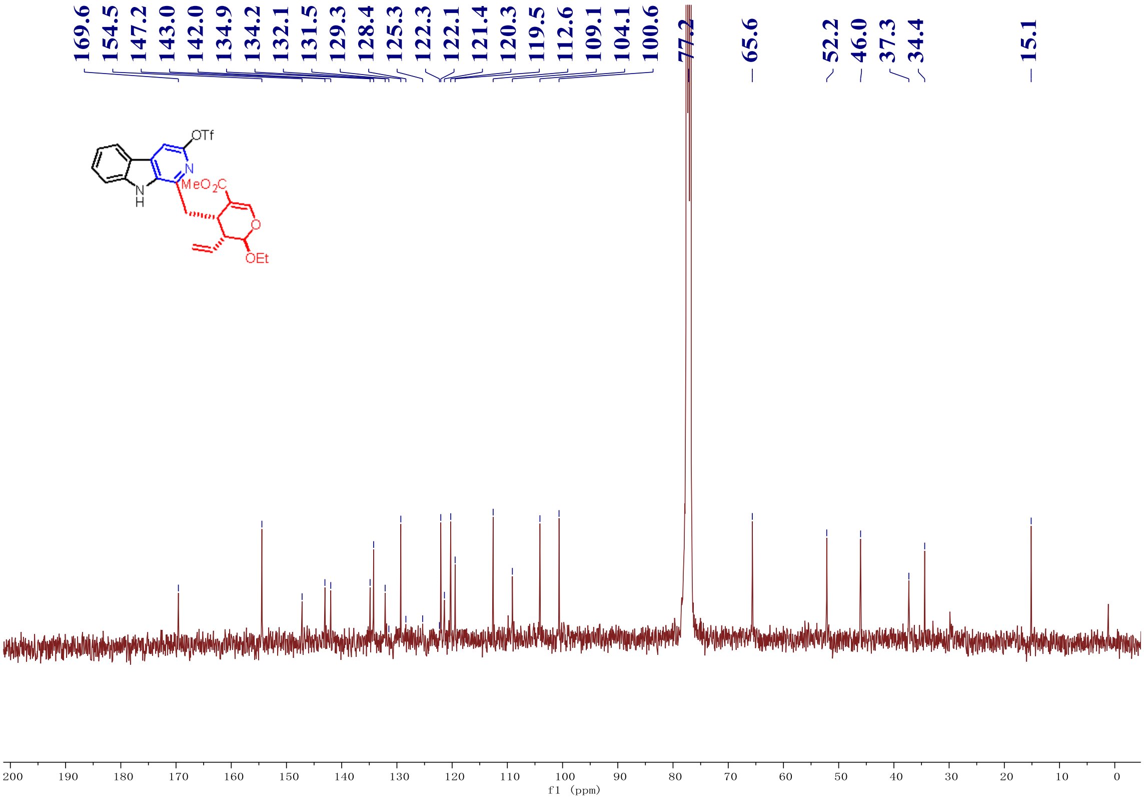

COSY NMR (300 MHz, $CDCl_3$), **(±)-37b**

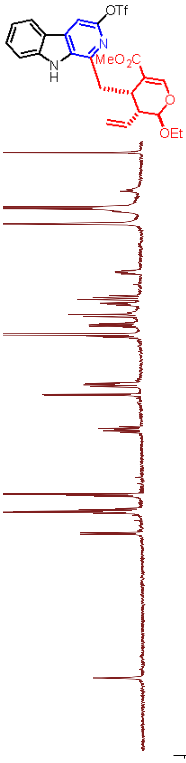

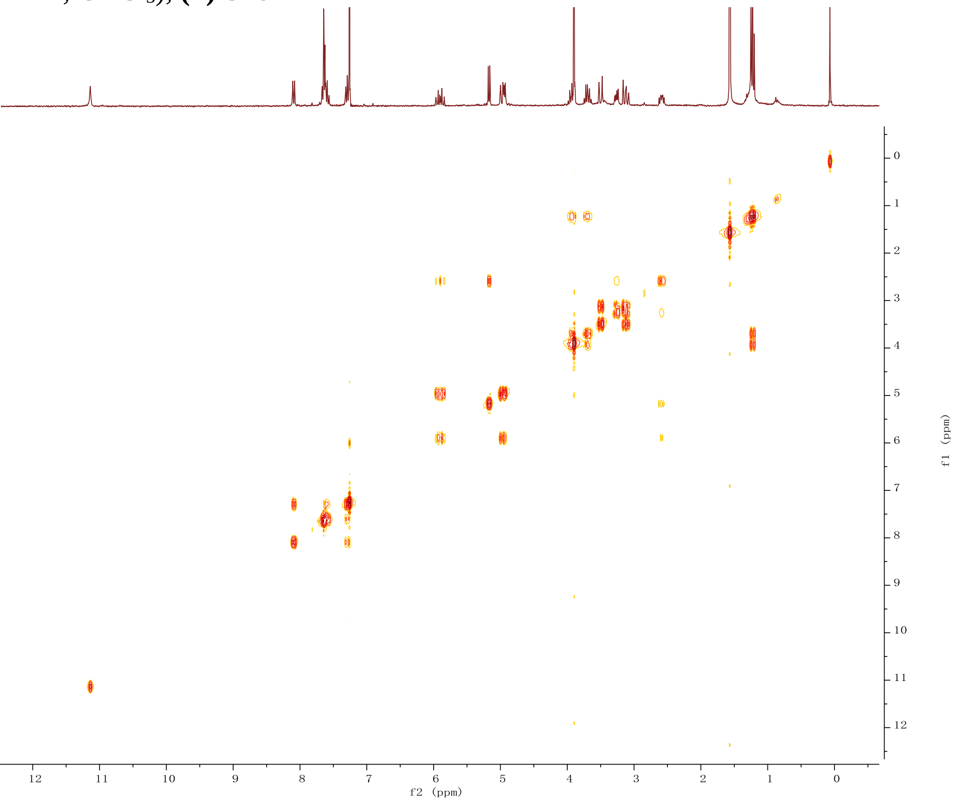

^{19}F NMR (235 MHz, $CDCl_3$), **(±)-37b**

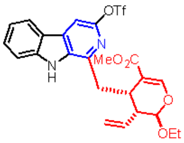

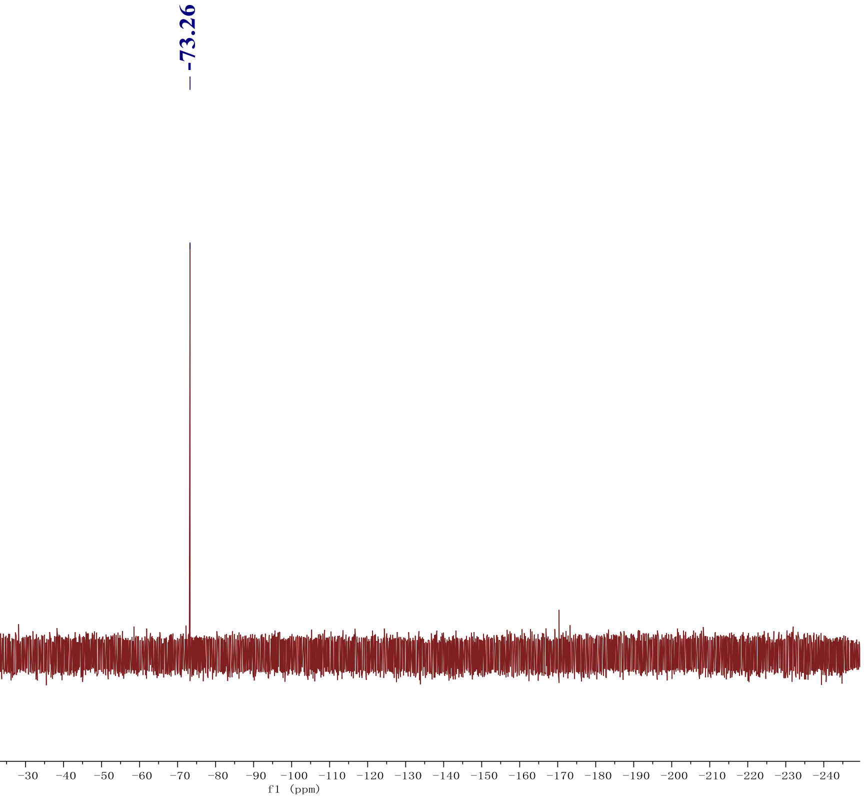

^{1}H NMR (360 MHz, $CDCl_3$), **15b**

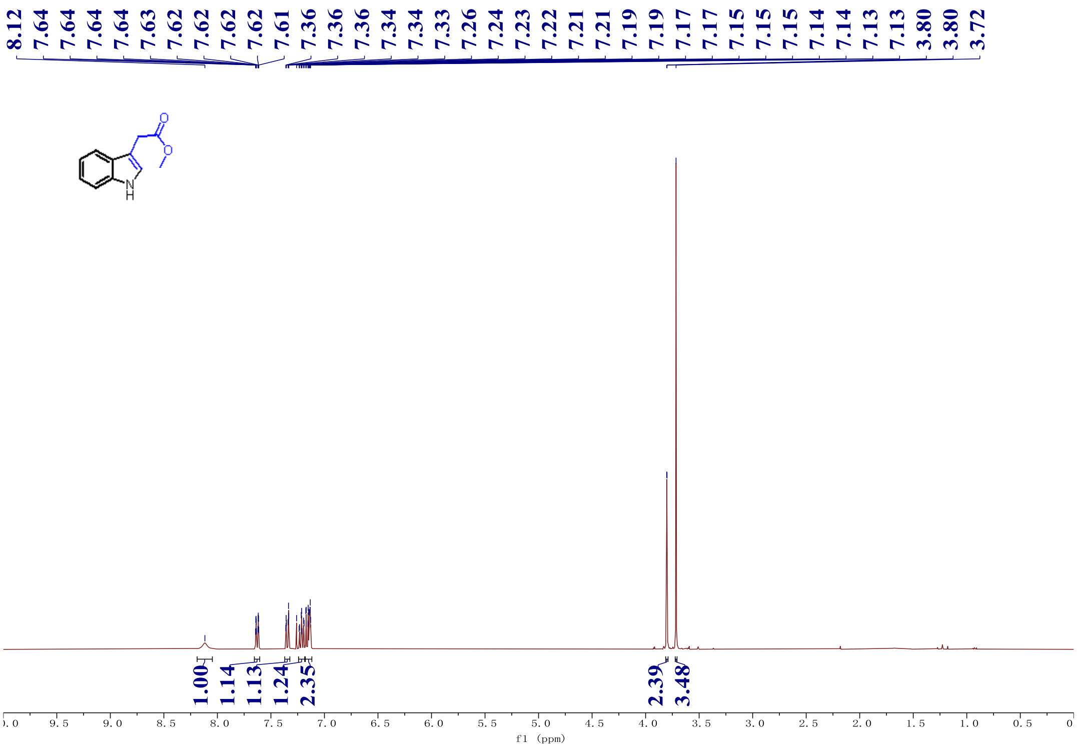

^{13}C NMR (90 MHz, $CDCl_3$), **15b**

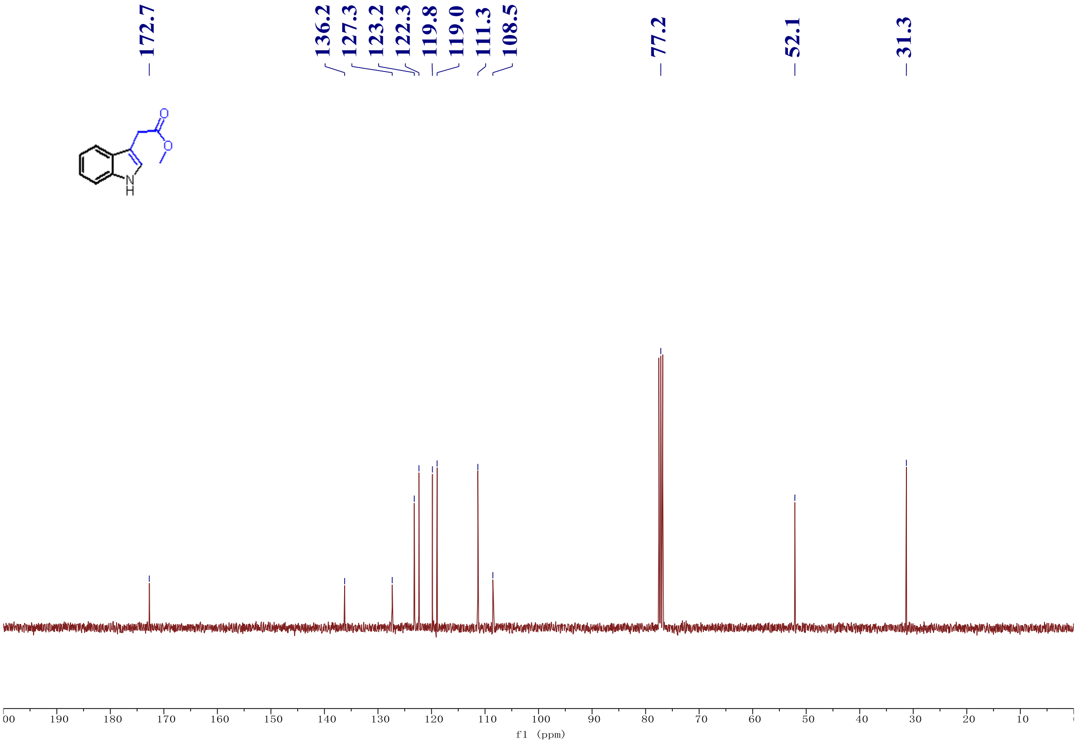

^{1}H NMR (360 MHz, $CDCl_3$), **15a**

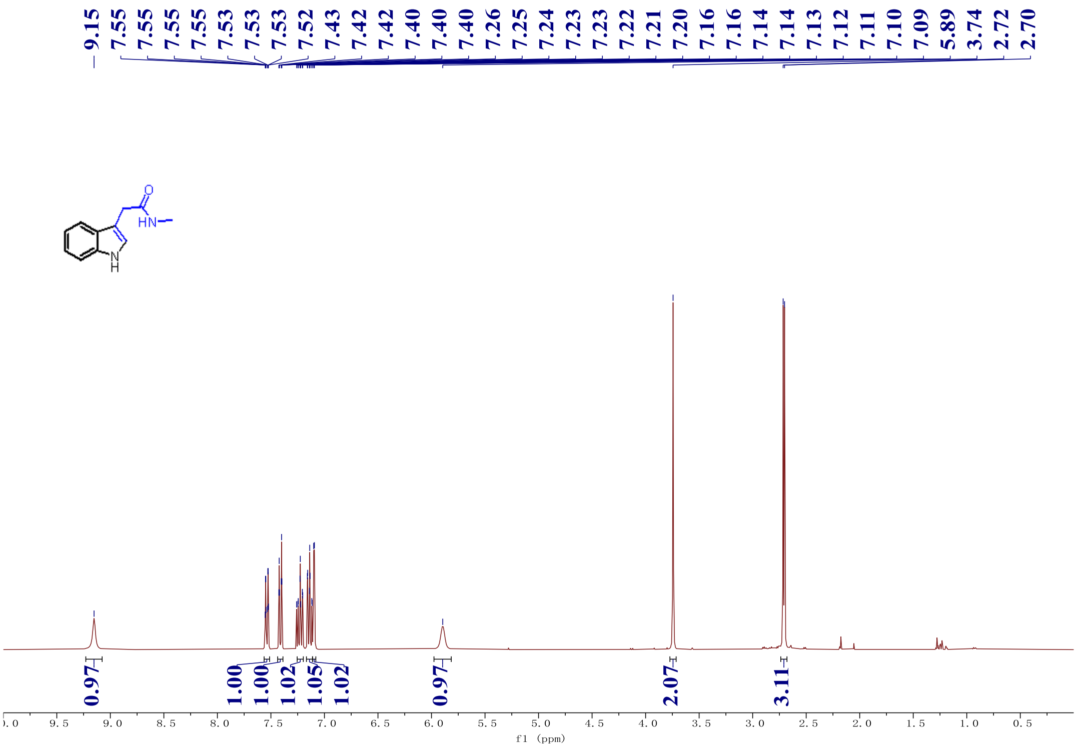

^{13}C NMR (90 MHz, $CDCl_3$), **15a**

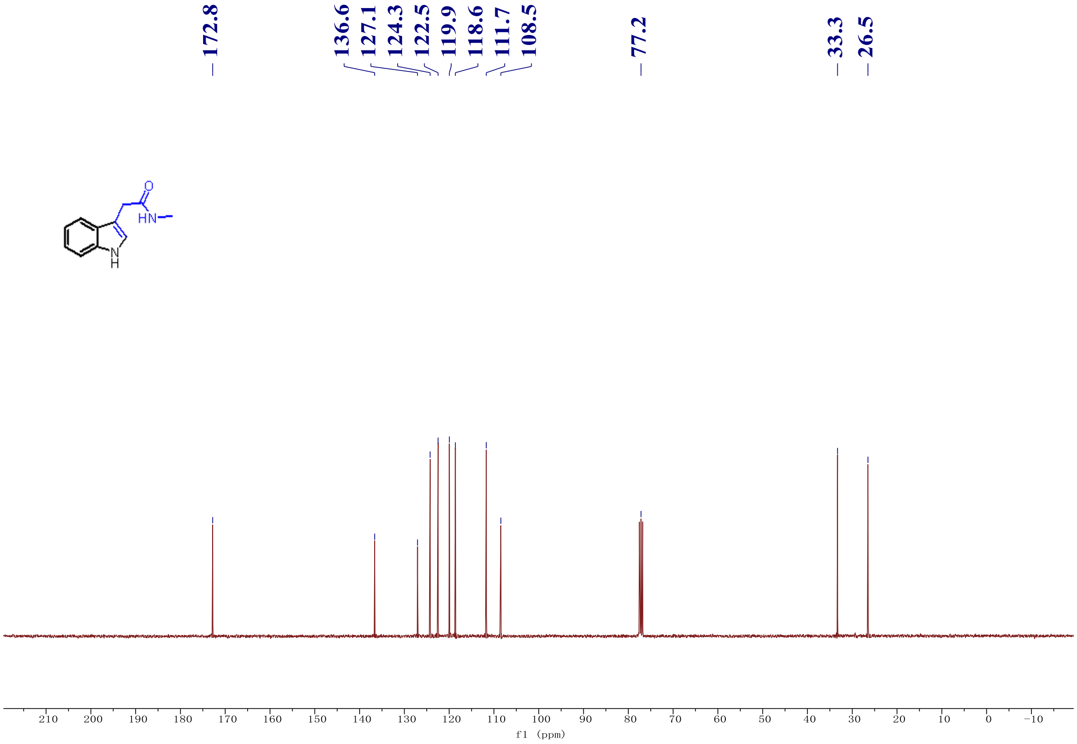

^{1}H NMR (300 MHz, $CDCl_3$), **(±)-40**

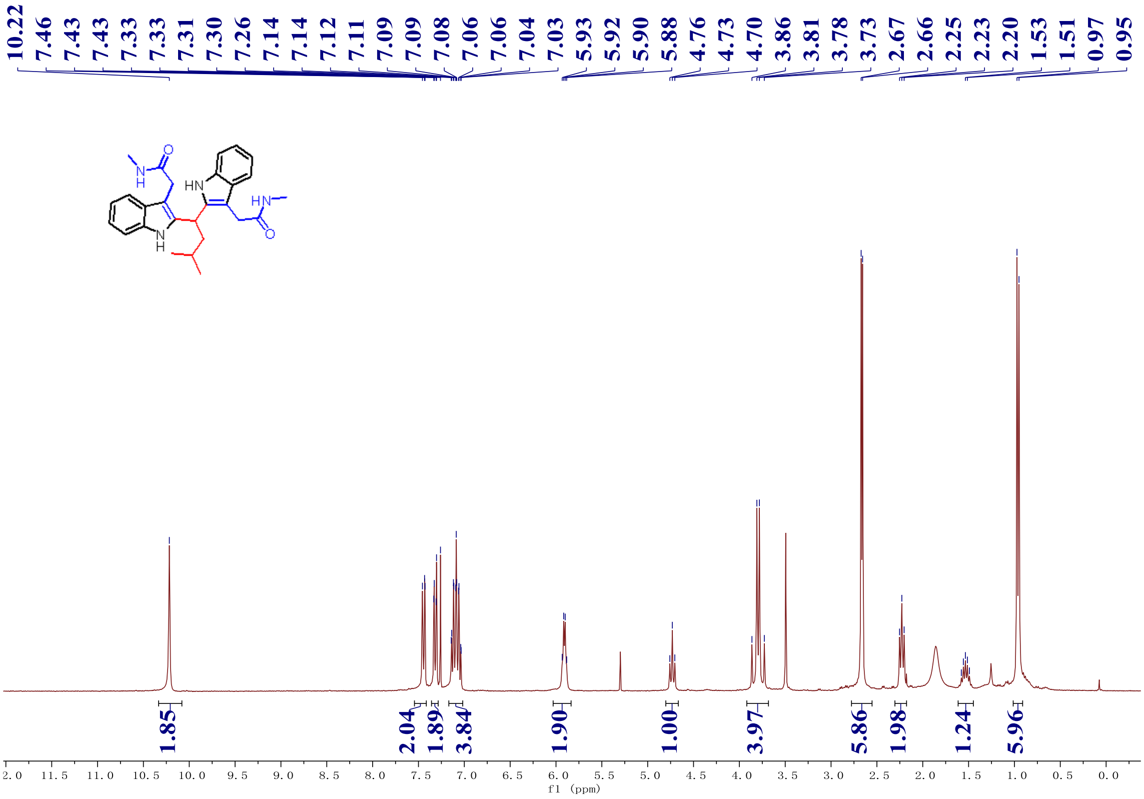

^{13}C NMR (100 MHz, $CDCl_3$), **(±)-40**

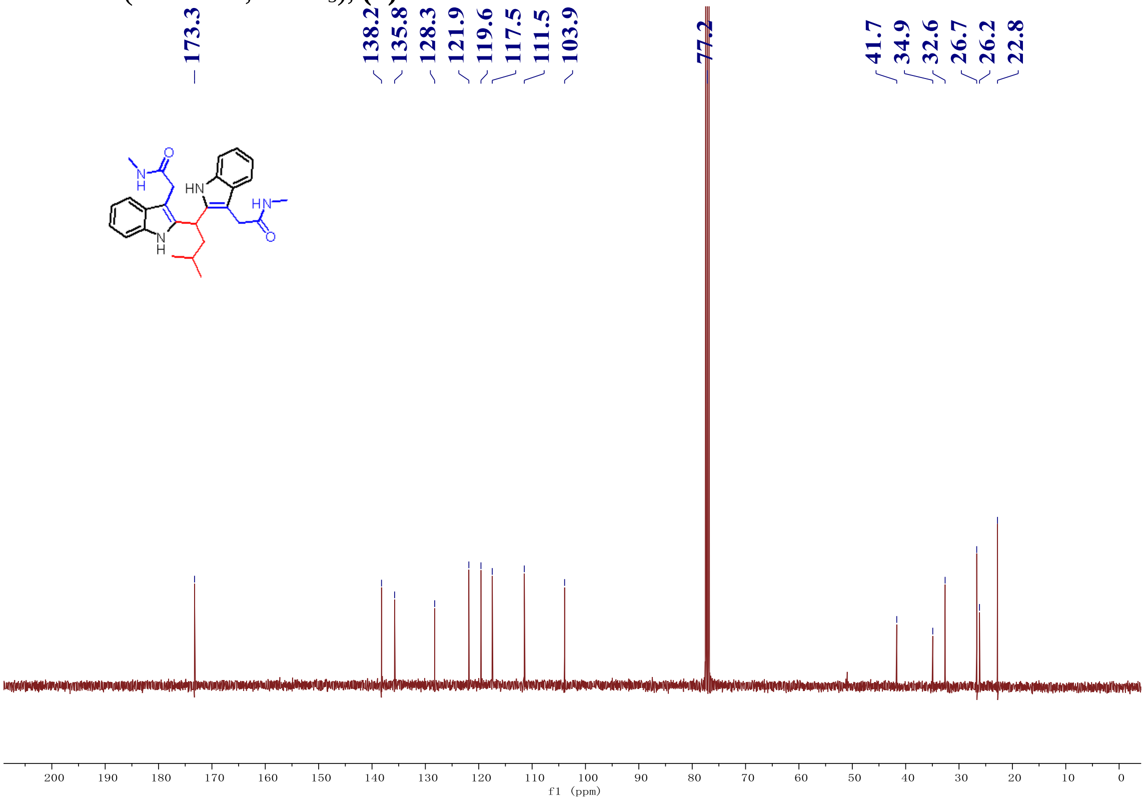

^{1}H NMR (300 MHz, CD_3OD), **43a**

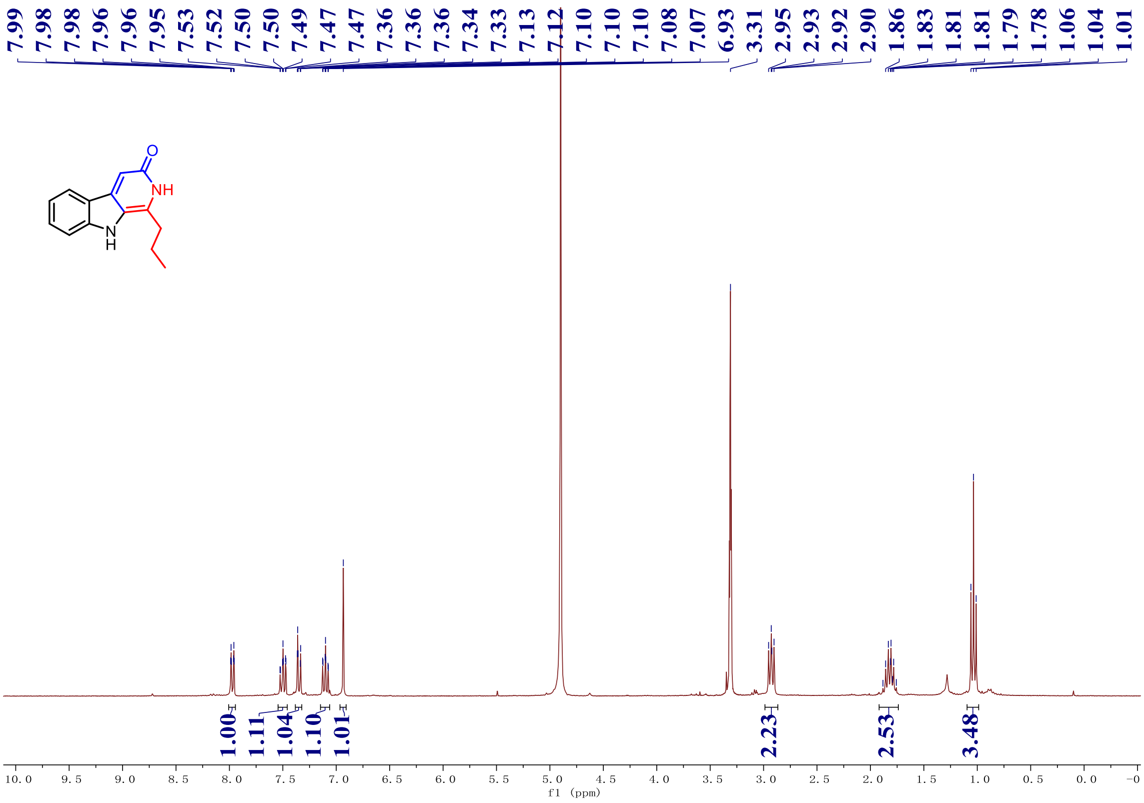

^{13}C NMR (100 MHz, CD_3OD), **43a**

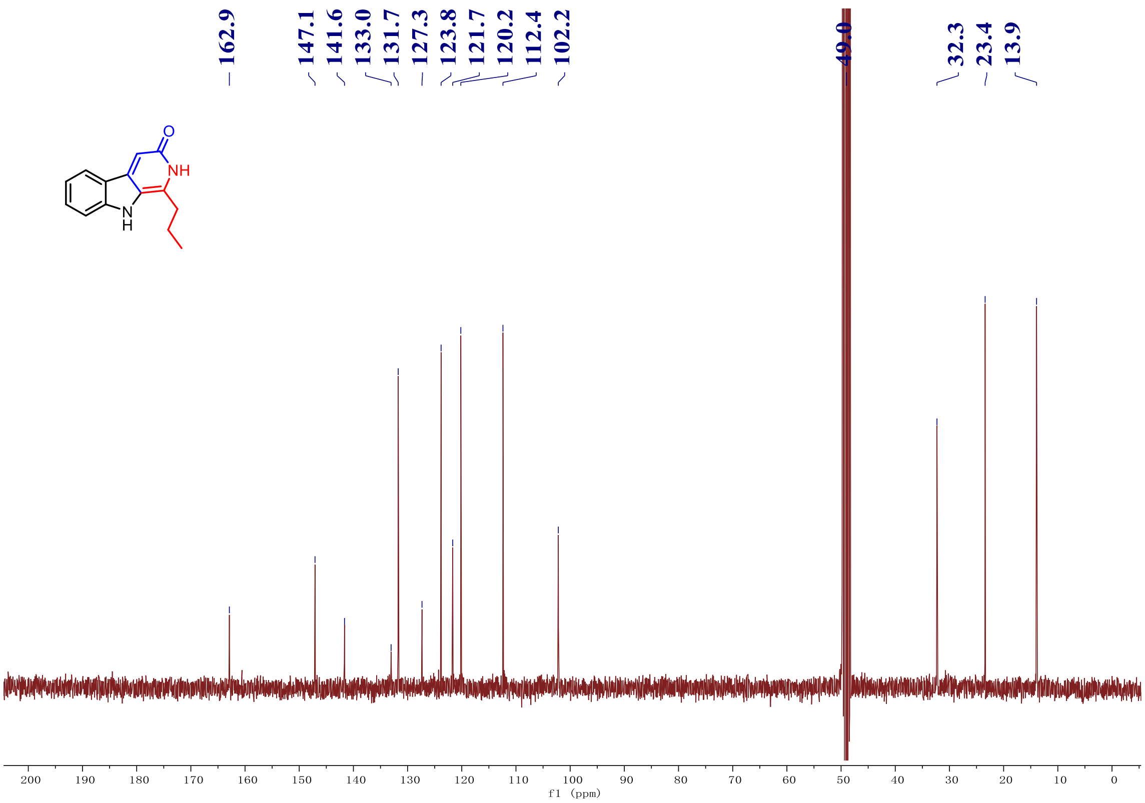

^{1}H NMR (300 MHz, $CDCl_3$), **26**

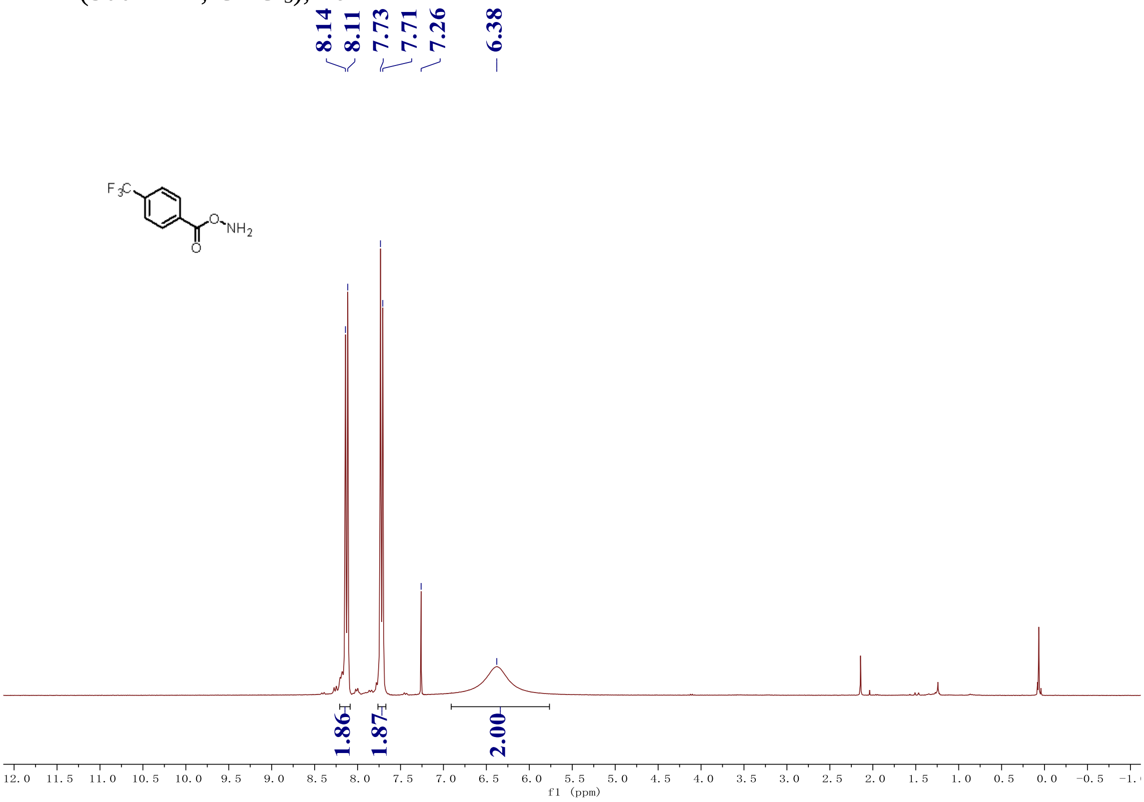

^{13}C NMR (90 MHz, $CDCl_3$), **26**

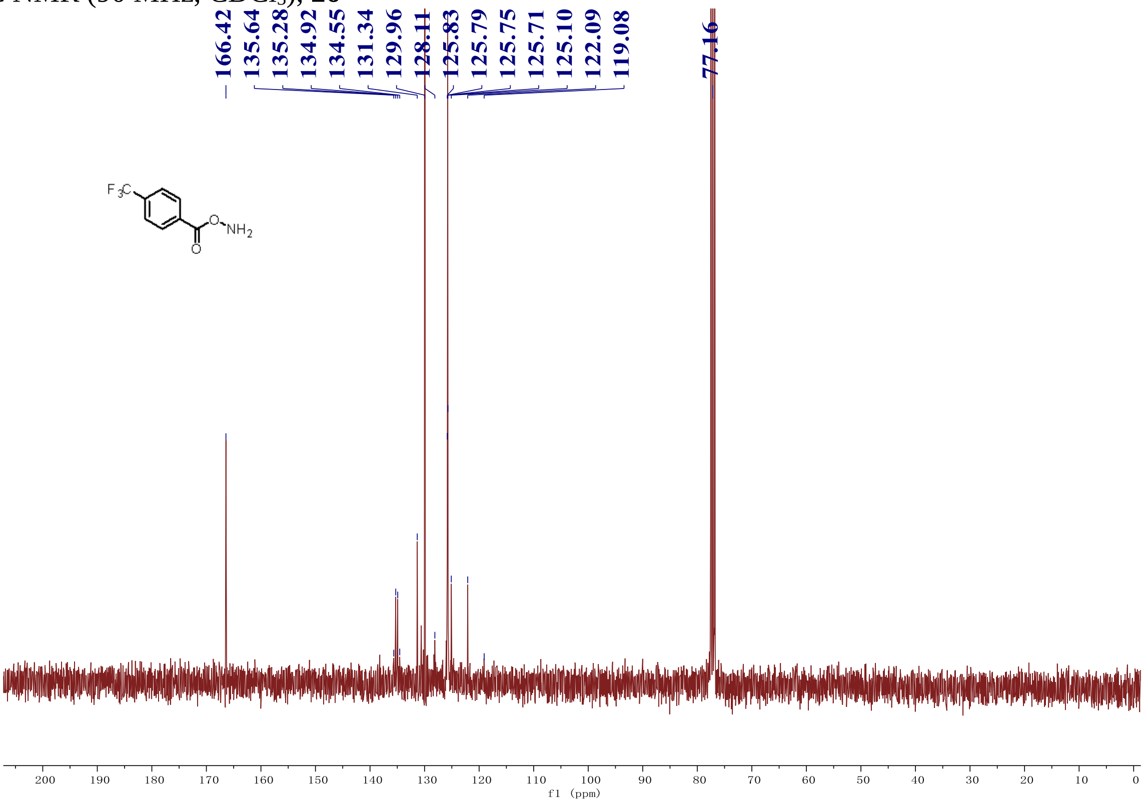

^{19}F NMR (235 MHz, $CDCl_3$), **26**

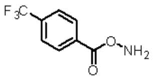

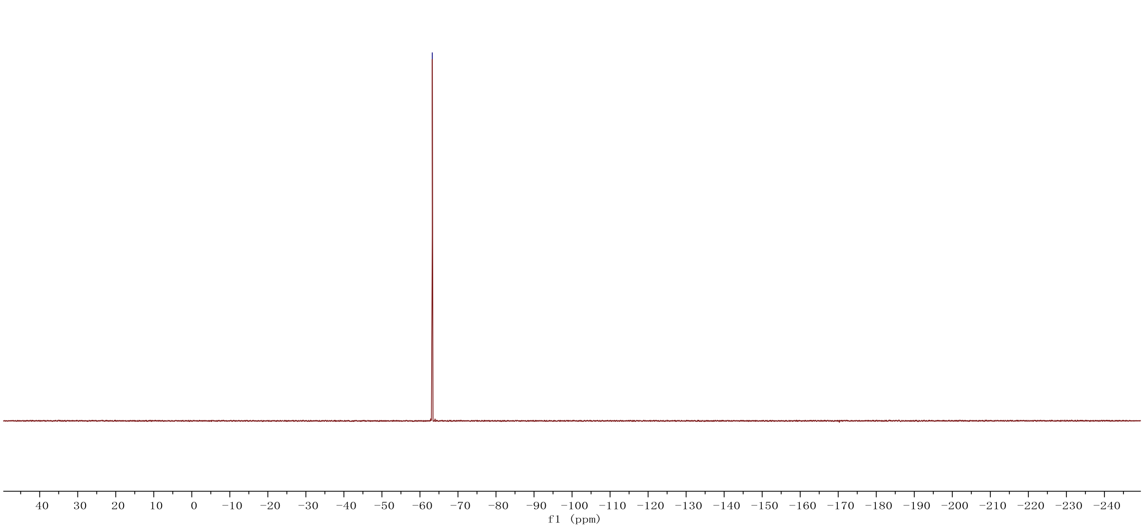

^{1}H NMR (300 MHz, $CDCl_3$), **(±)-25**

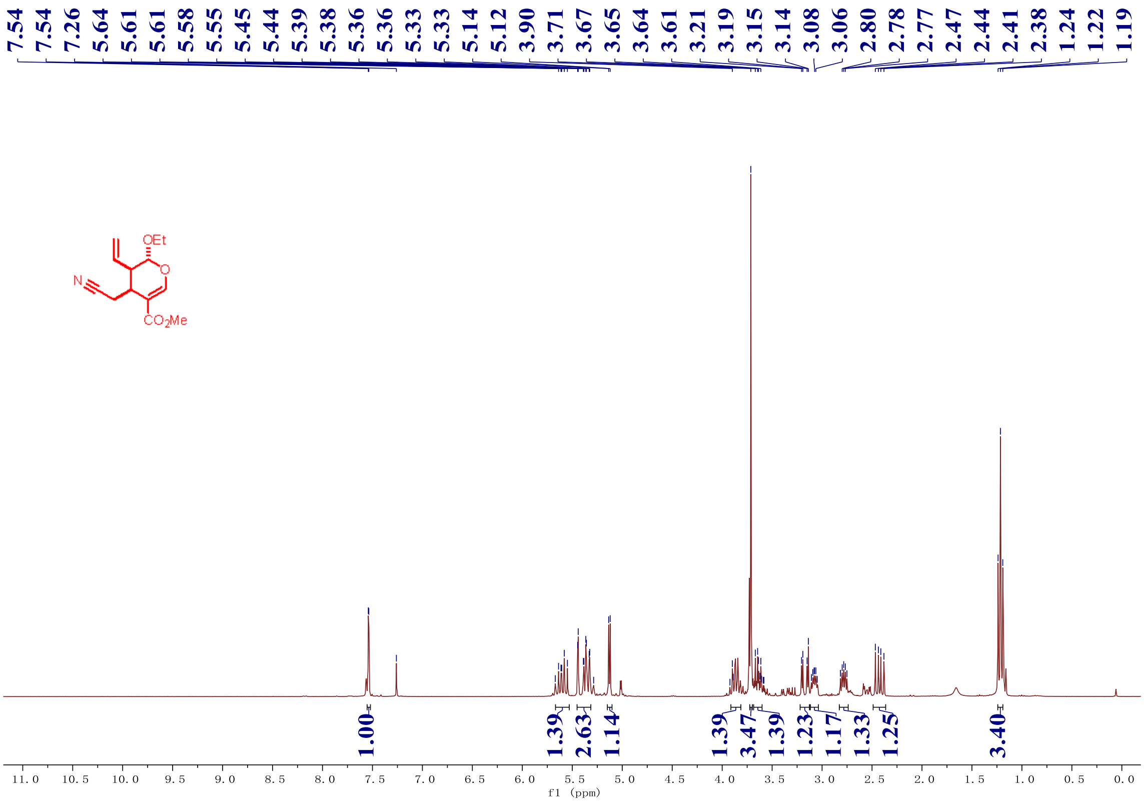

^{13}C NMR (100 MHz, $CDCl_3$), **(±)-25**

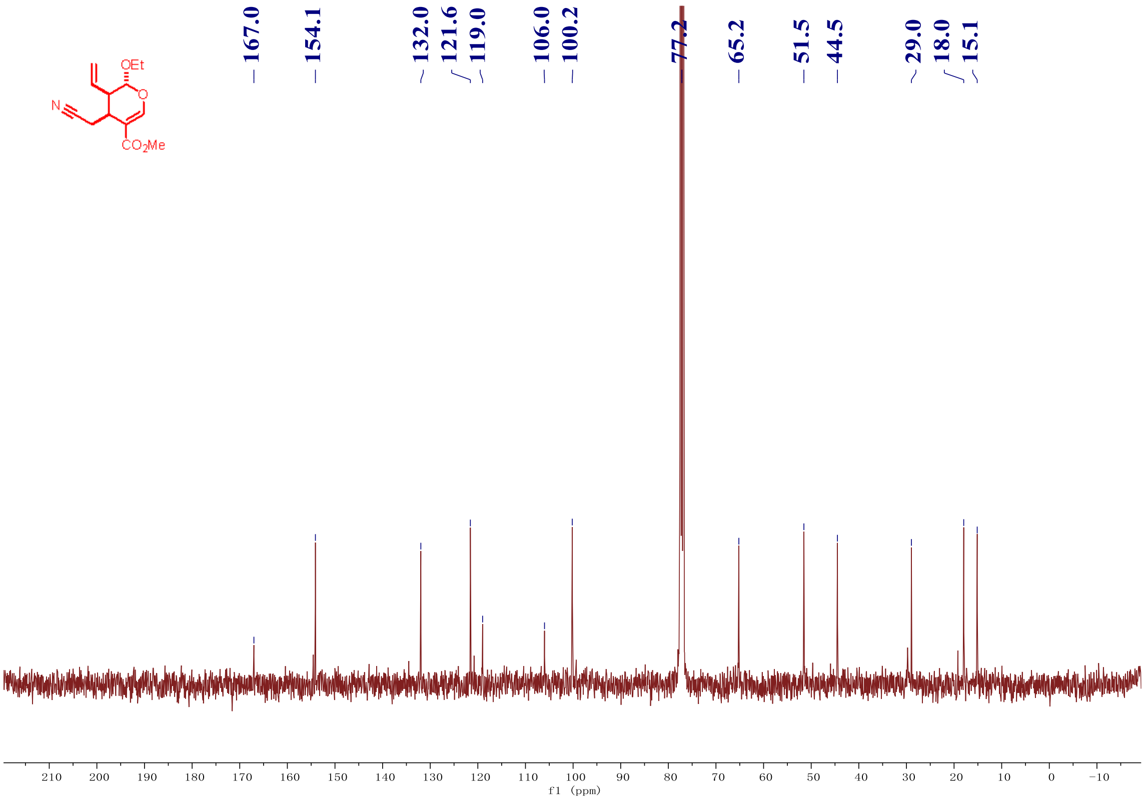

^{1}H NMR (360 MHz, $CDCl_3$), **46b**

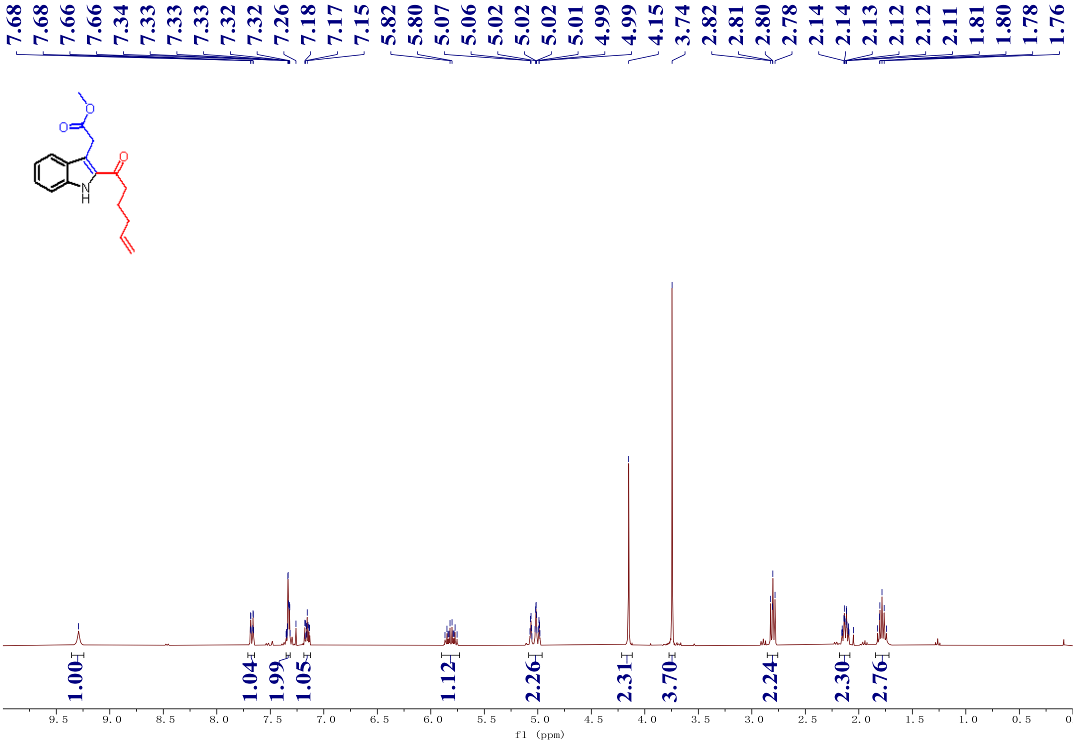

^{13}C NMR (90 MHz, $CDCl_3$), **46b**

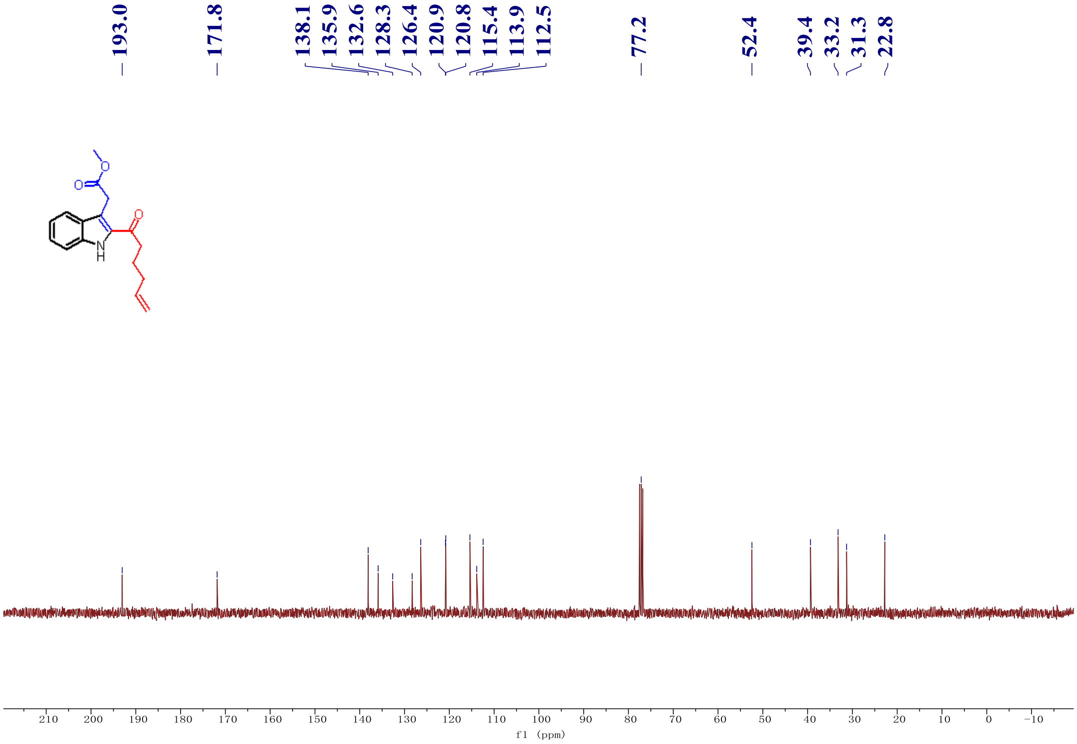

^{1}H NMR (360 MHz, CD_3OD), **46a**

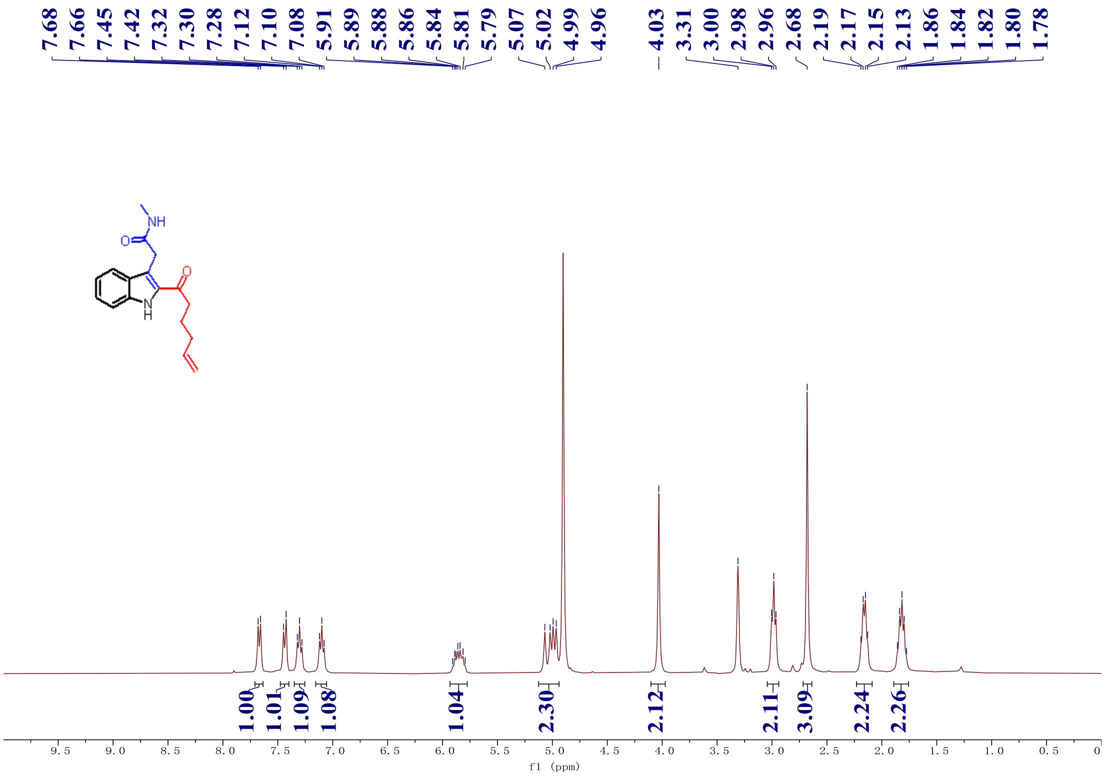

^{13}C NMR (90 MHz, CD_3OD), **46a**

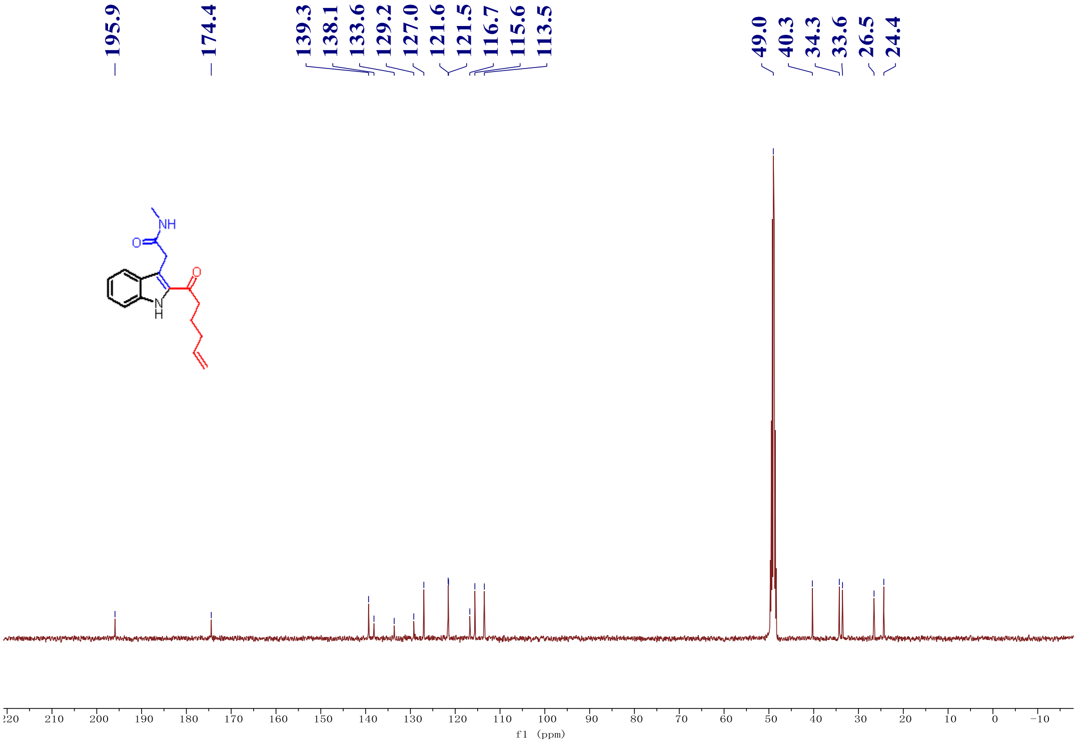

^{1}H NMR (360 MHz, CD_3OD), **47**

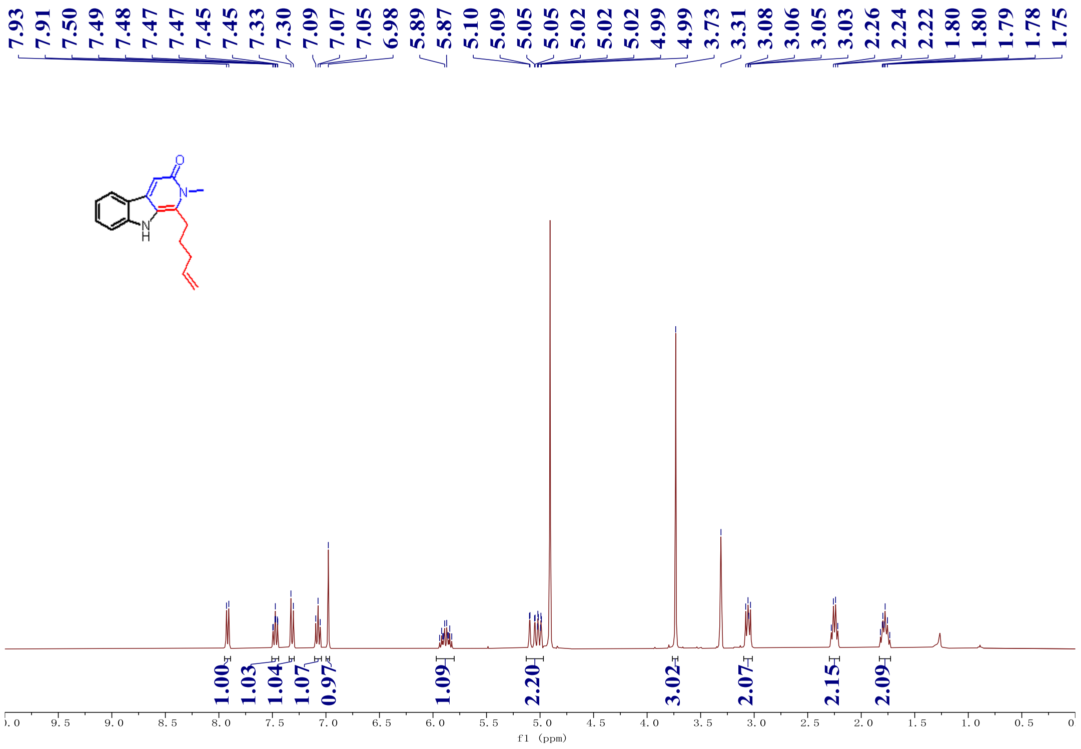

^{13}C NMR (90 MHz, CD_3OD), **47**

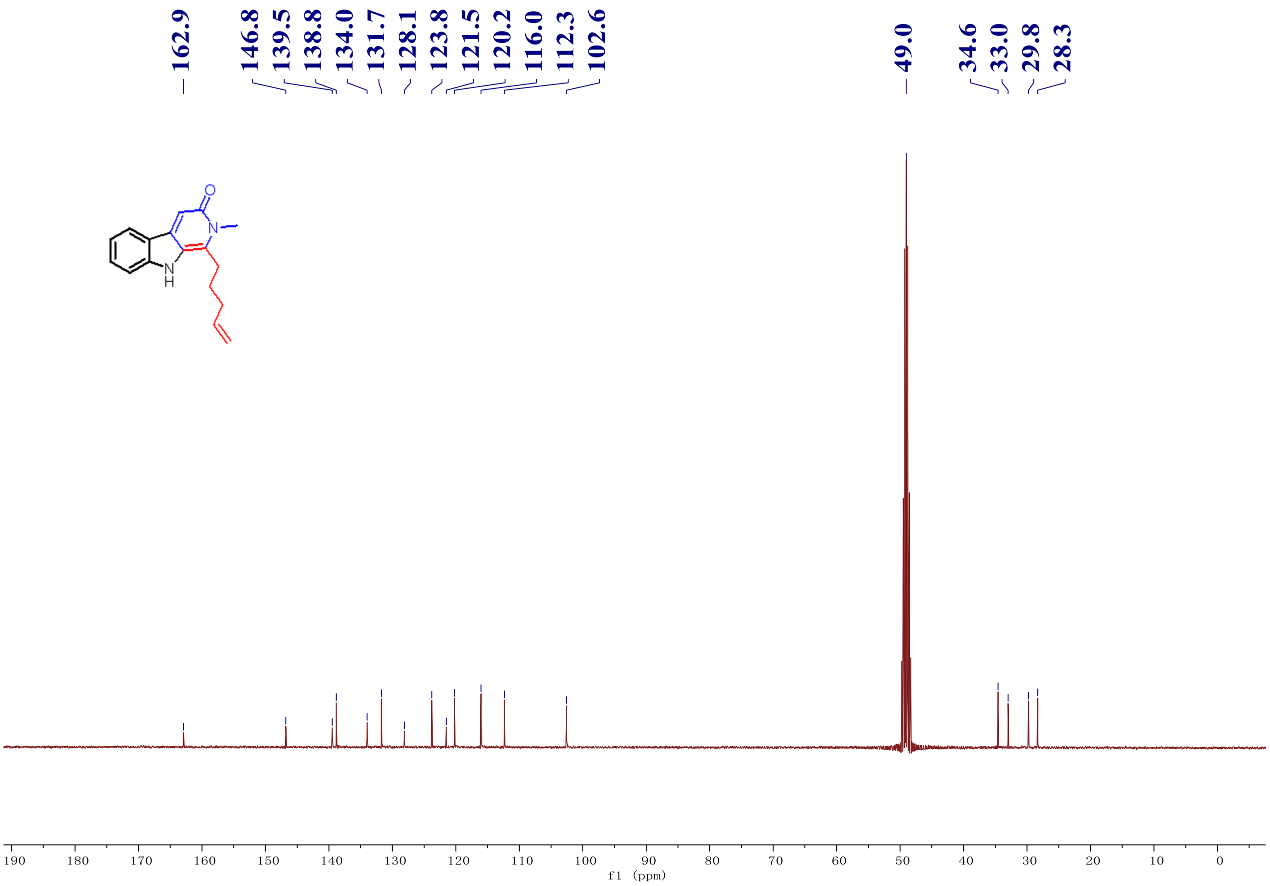

^{1}H NMR (300 MHz, $CDCl_3$), (±)-**48**

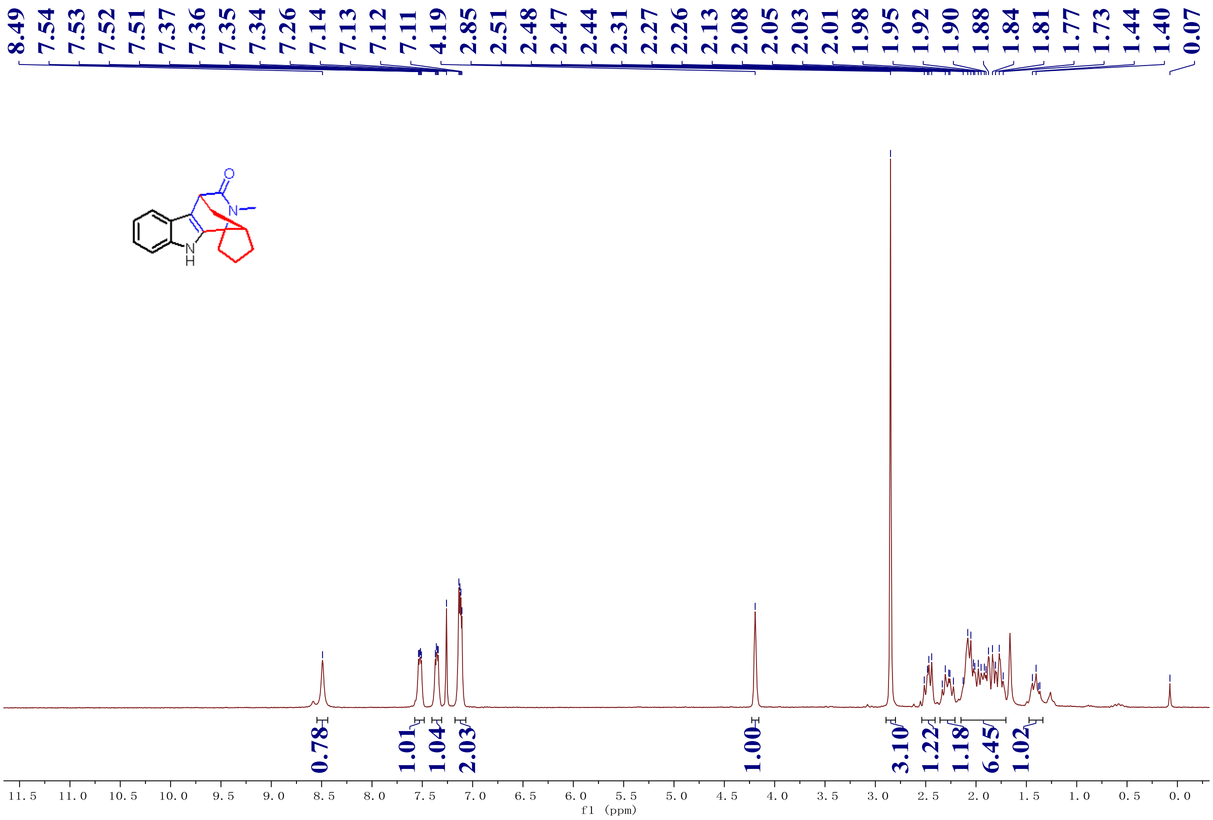

^{13}C NMR (75 MHz, $CDCl_3$), (±)-**48**

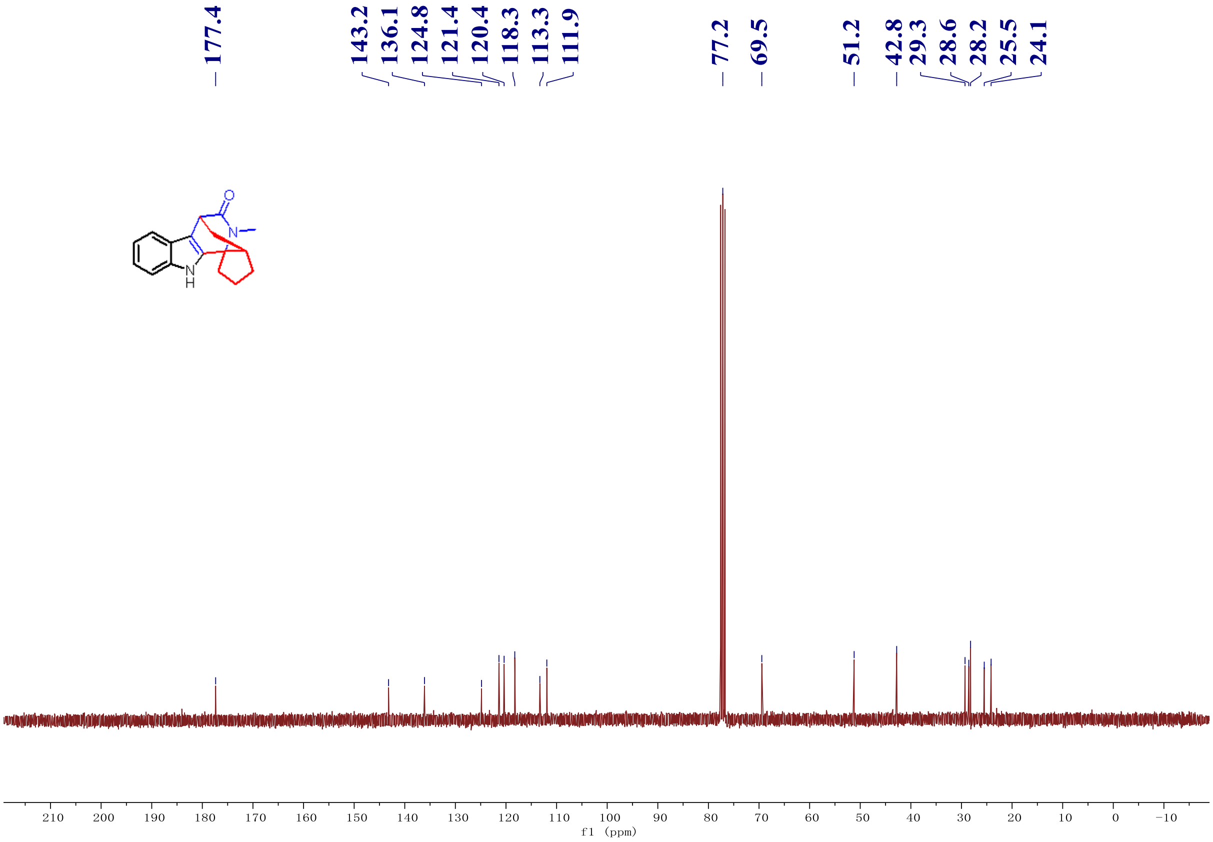

^{1}H NMR (300 MHz, $CDCl_3$), **(±)-23**

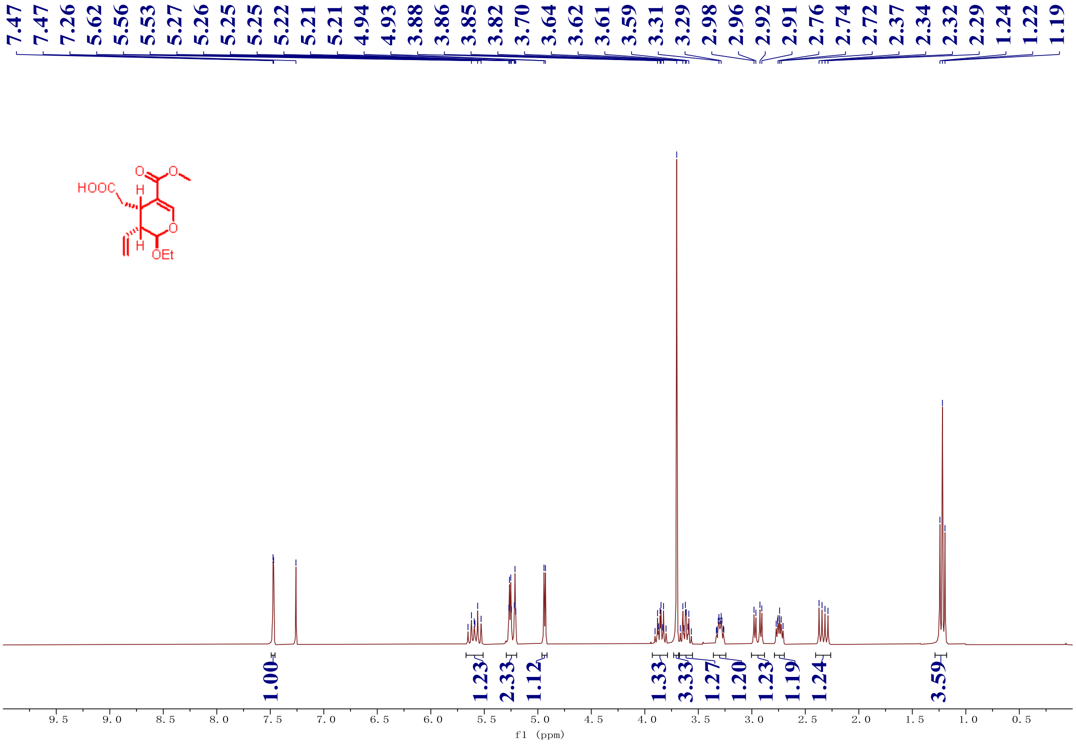

^{13}C NMR (100 MHz, $CDCl_3$), **(±)-23**

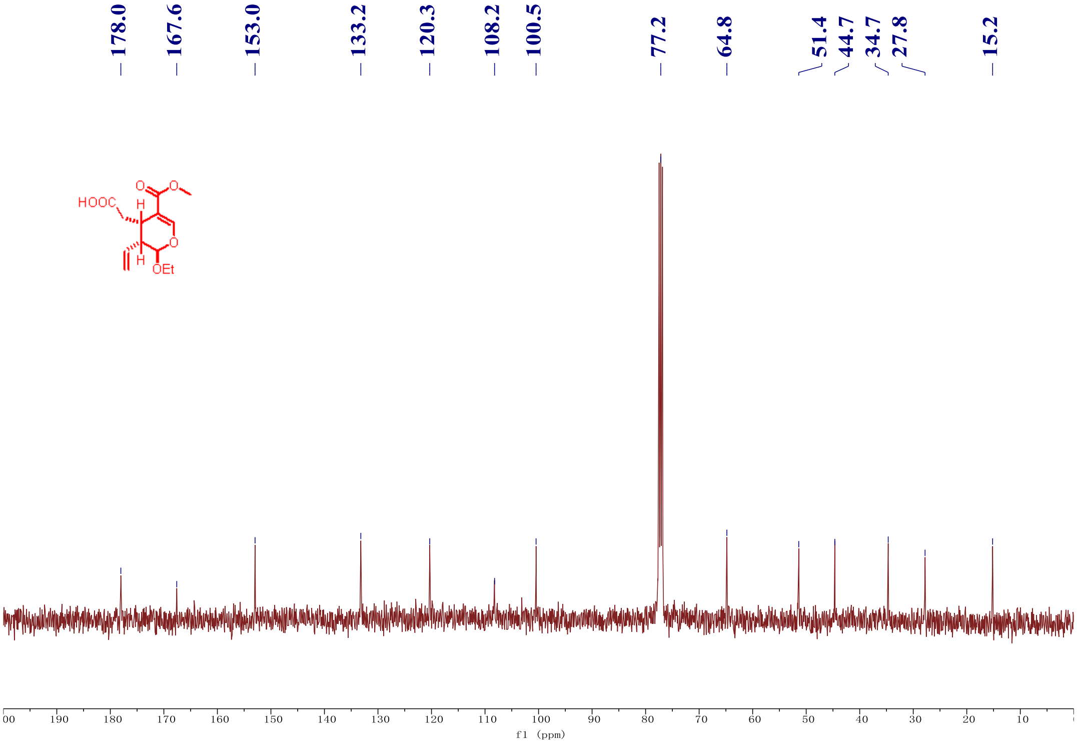

^{1}H NMR (360 MHz, $CDCl_3$), **(±)-51**

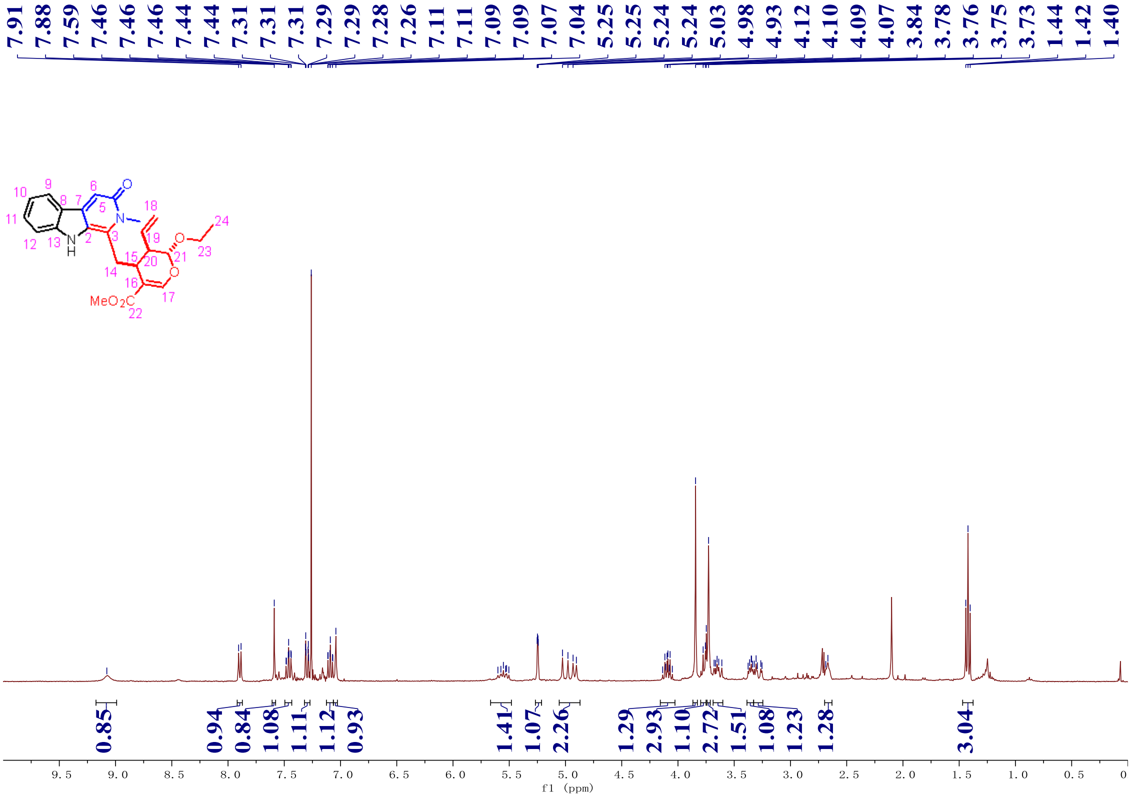

^{13}C NMR (100 MHz, $CDCl_3$), **(±)-51**

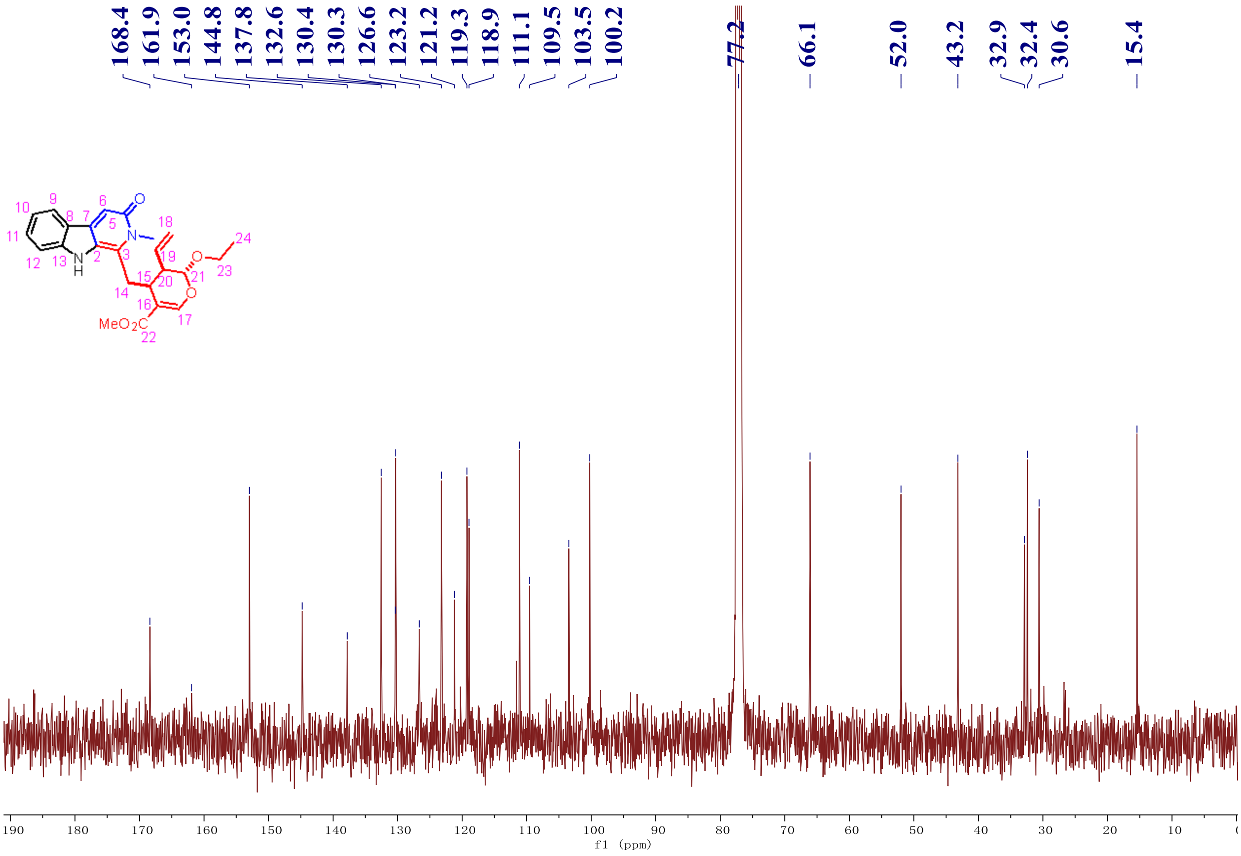

COSY (360 MHz, $CDCl_3$), **(±)-51**

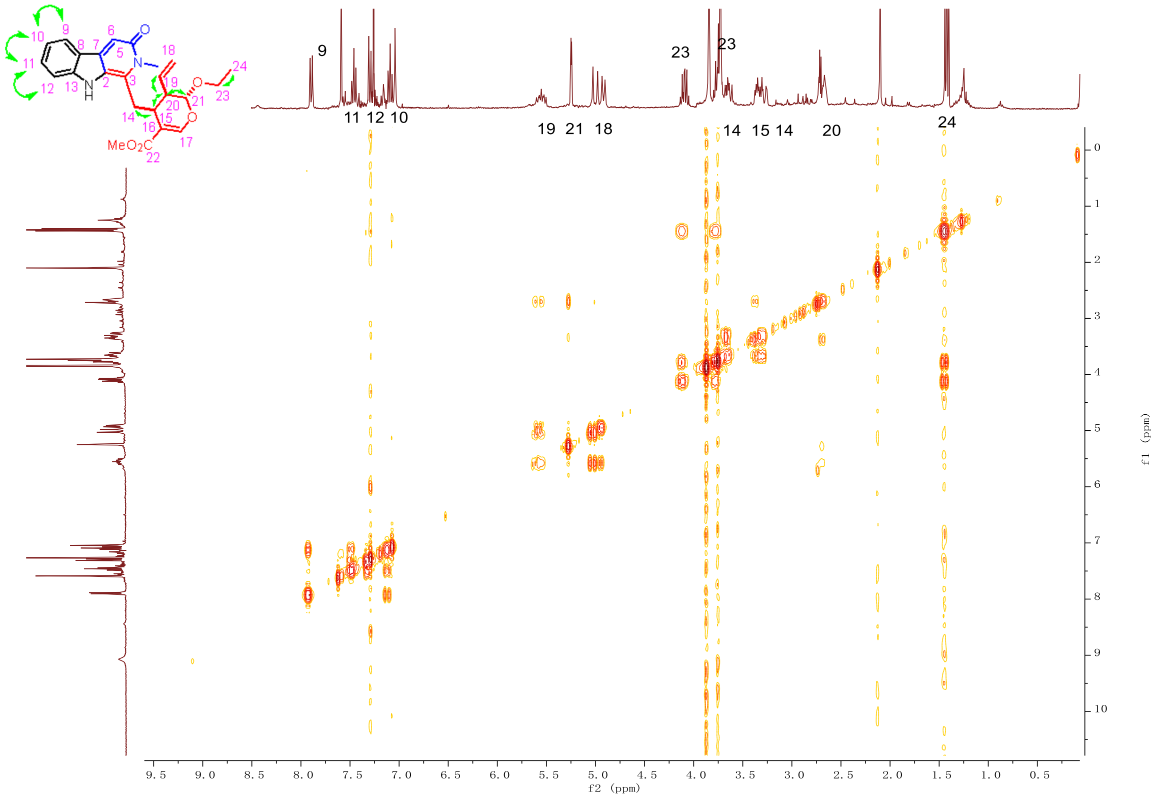

HSQC (400 MHz, $CDCl_3$), **(±)-51**

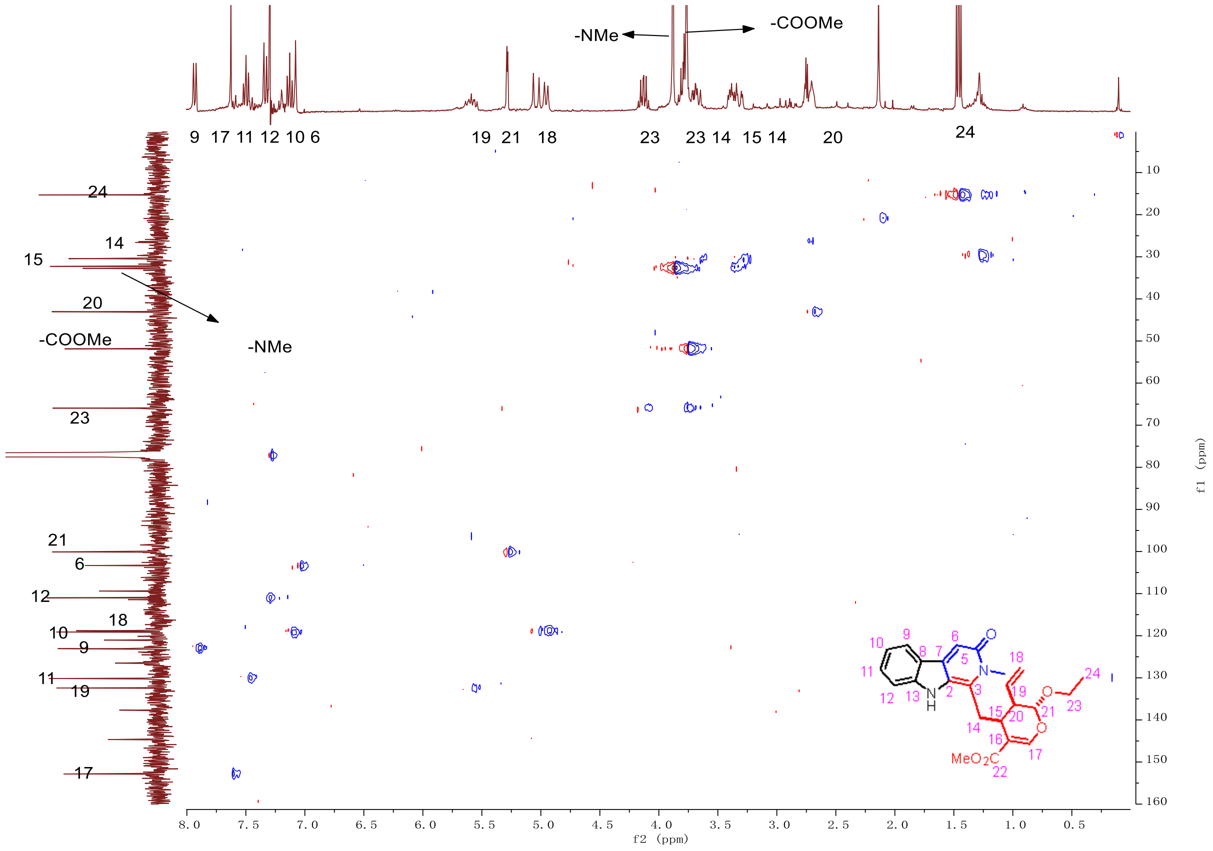

HMBC (400 MHz, $CDCl_3$), **(±)-51**

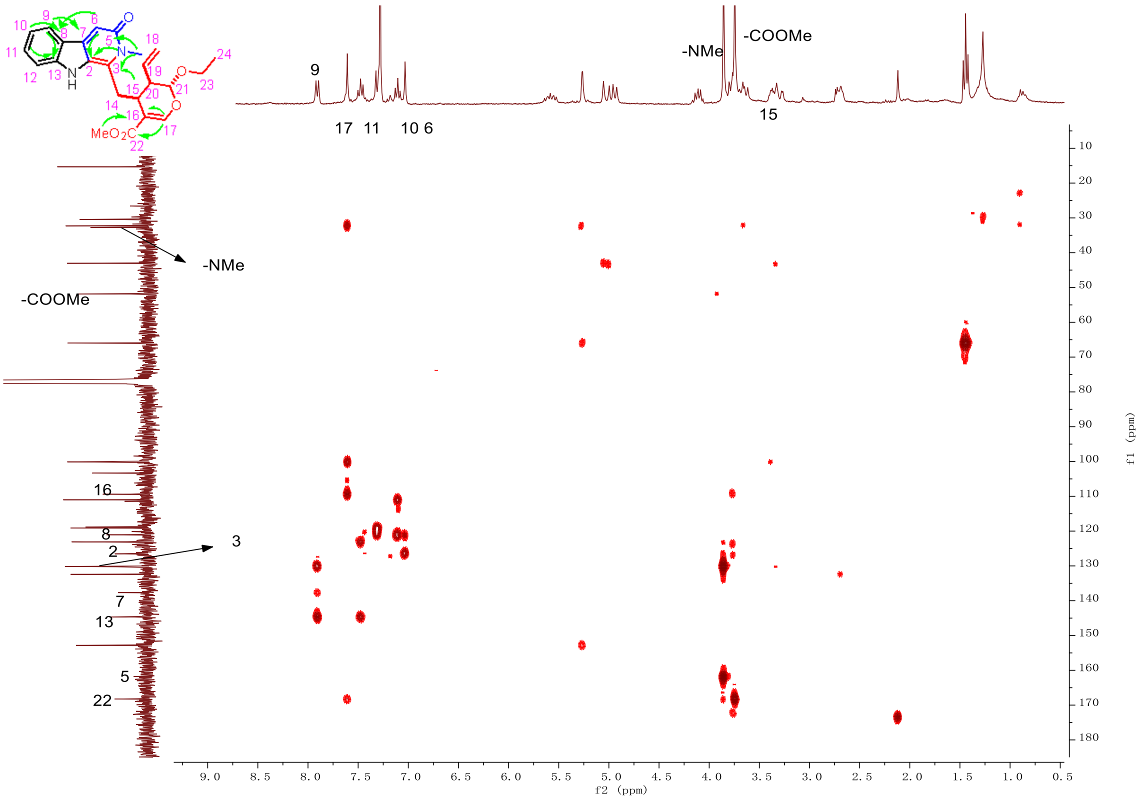

DEPT (400 MHz, $CDCl_3$), **(±)-51**

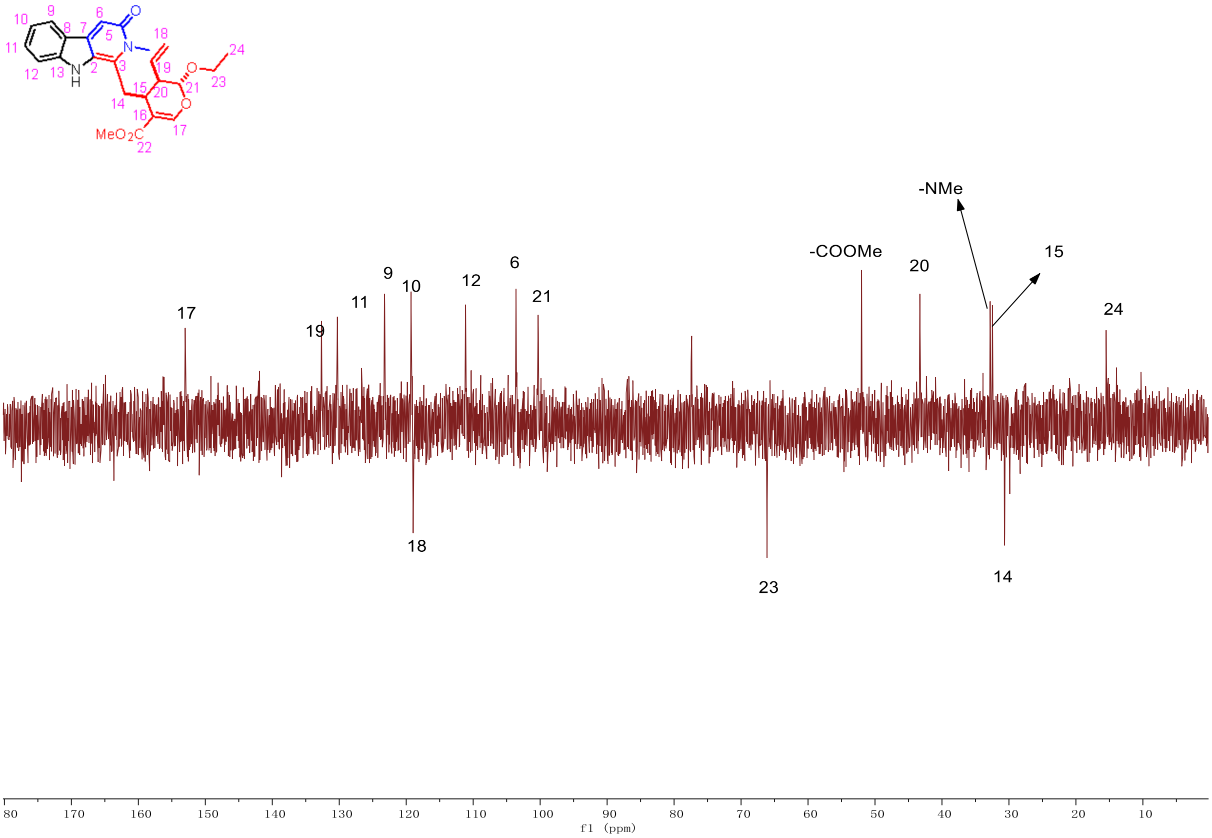

NOESY (400 MHz, $CDCl_3$), **(±)-51**

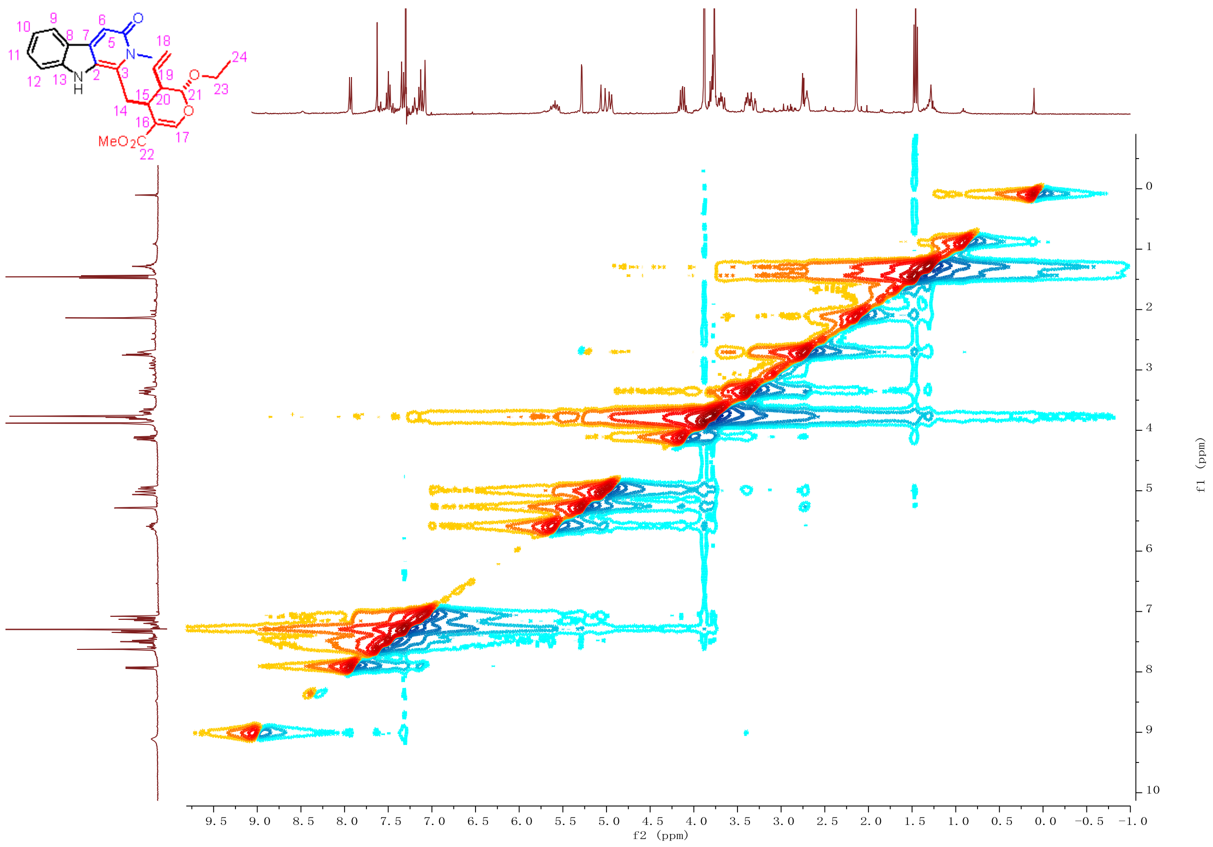
f2 (ppm)
f1 (ppm)
MeO2C

^{1}H NMR (360 MHz, $CDCl_3$), **(±)-53**

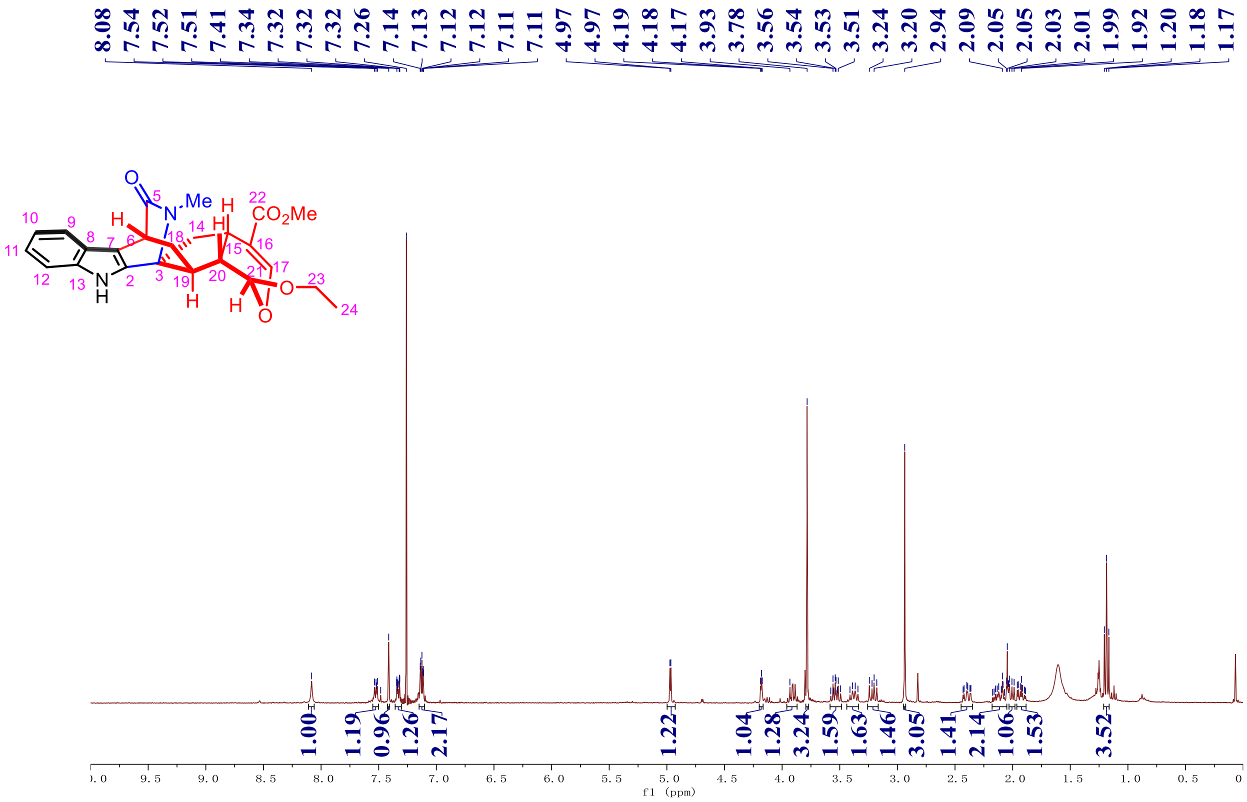

^{13}C NMR (100 MHz, $CDCl_3$), **(±)-53**

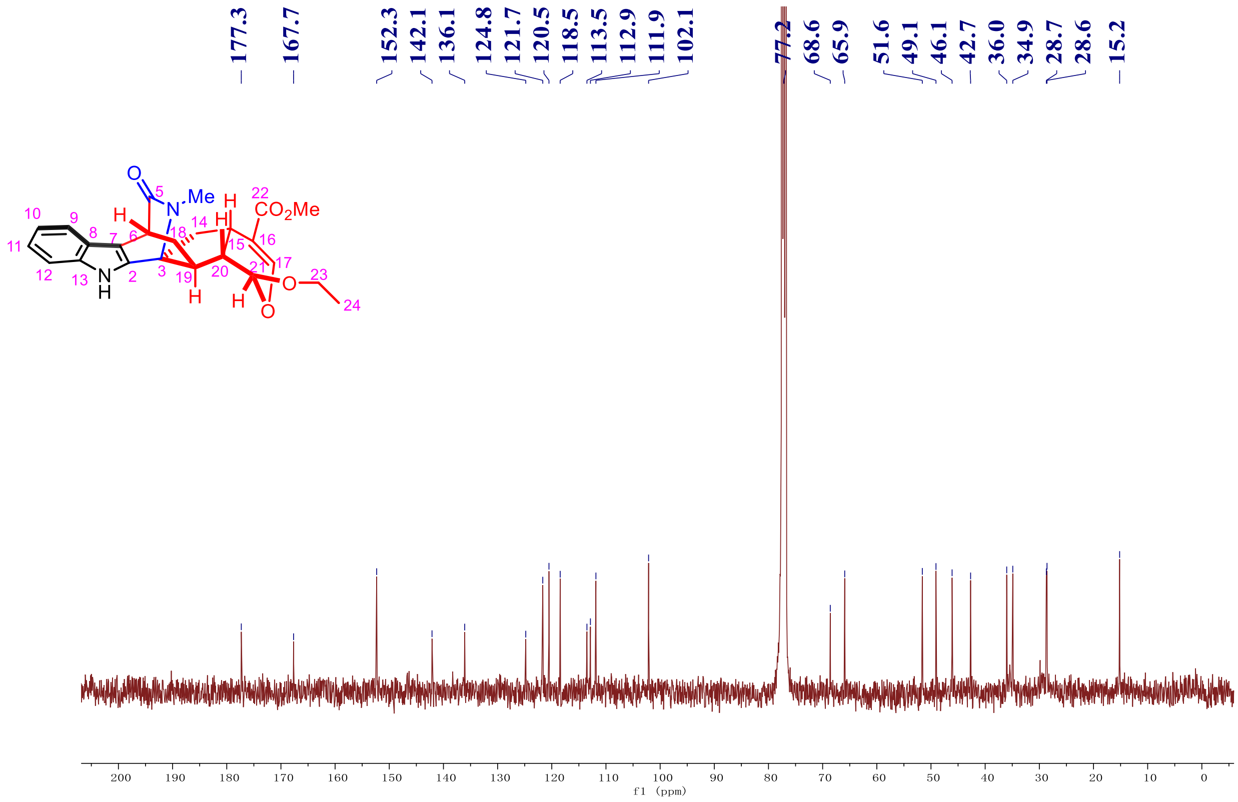

COSY (360 MHz, $CDCl_3$), **(±)-53**

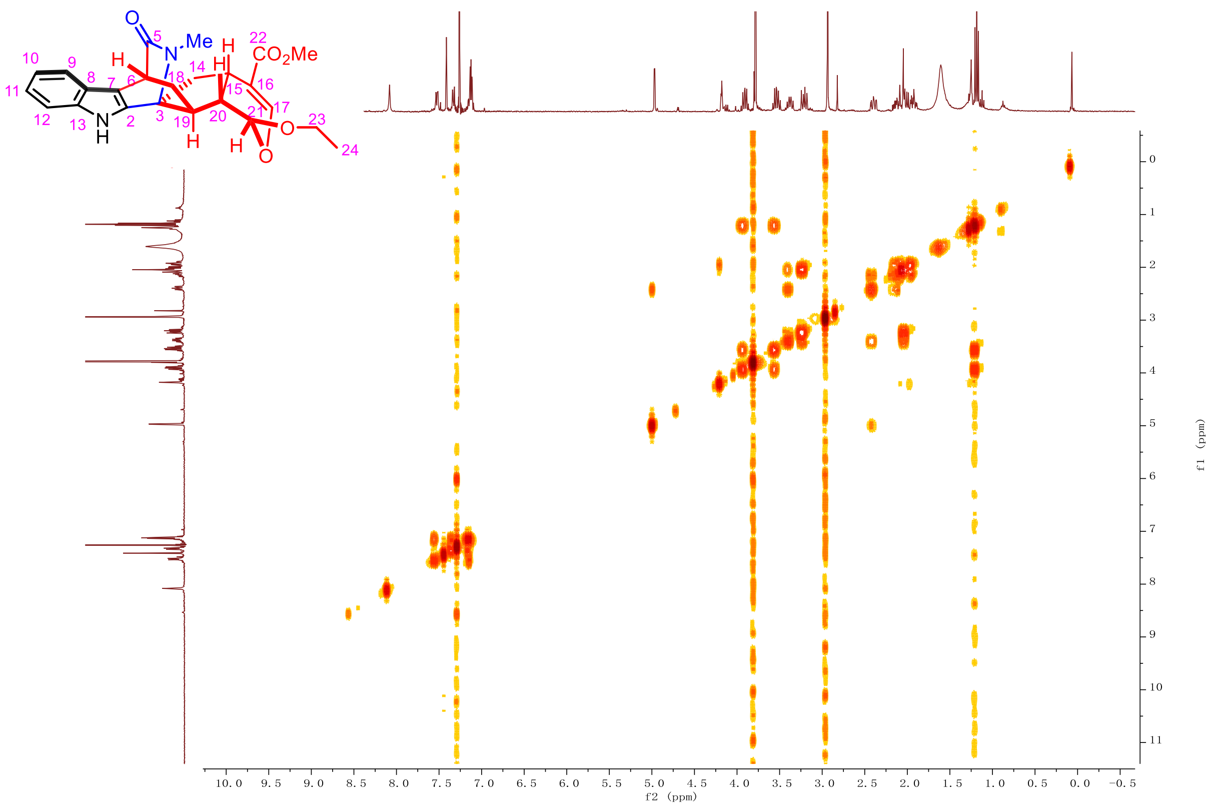

HSQC (360 MHz, $CDCl_3$), **(±)-53**

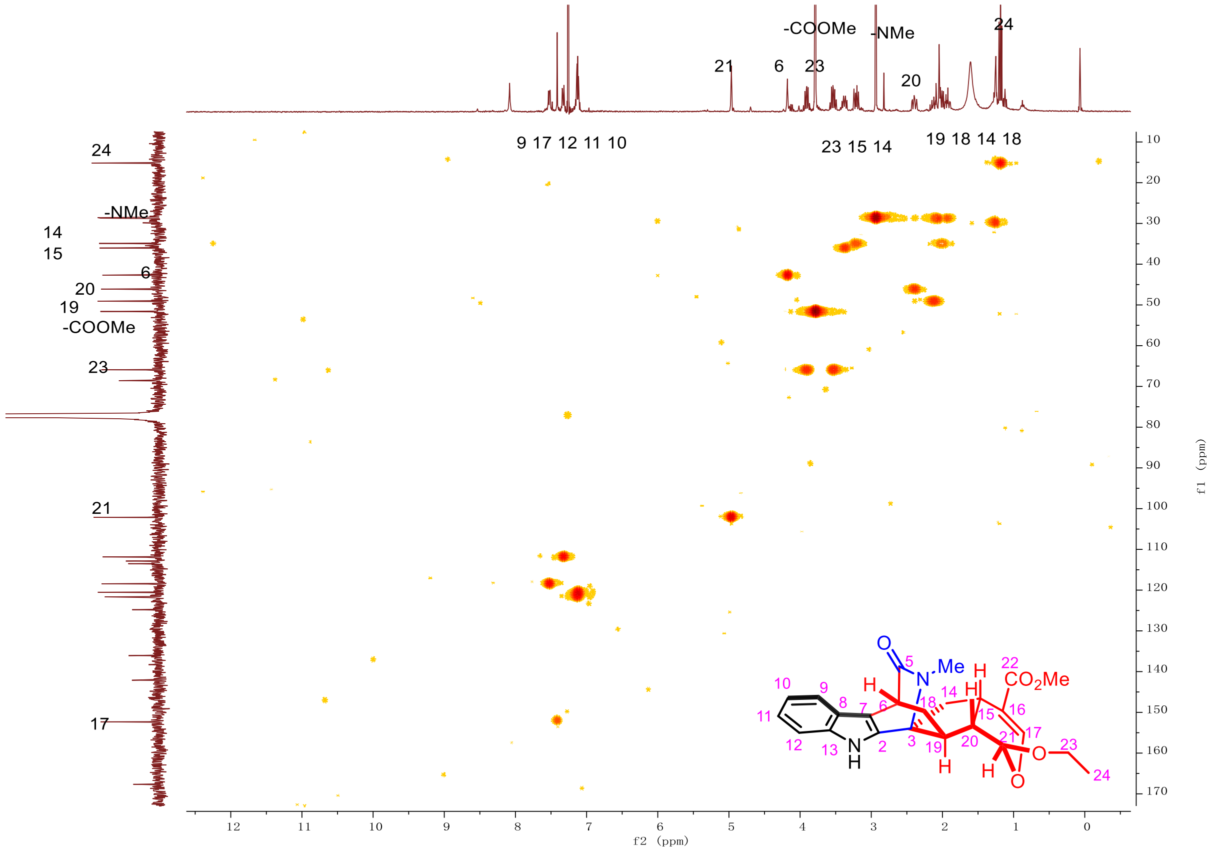

DEPT (360 MHz, $CDCl_3$), **(±)-53**

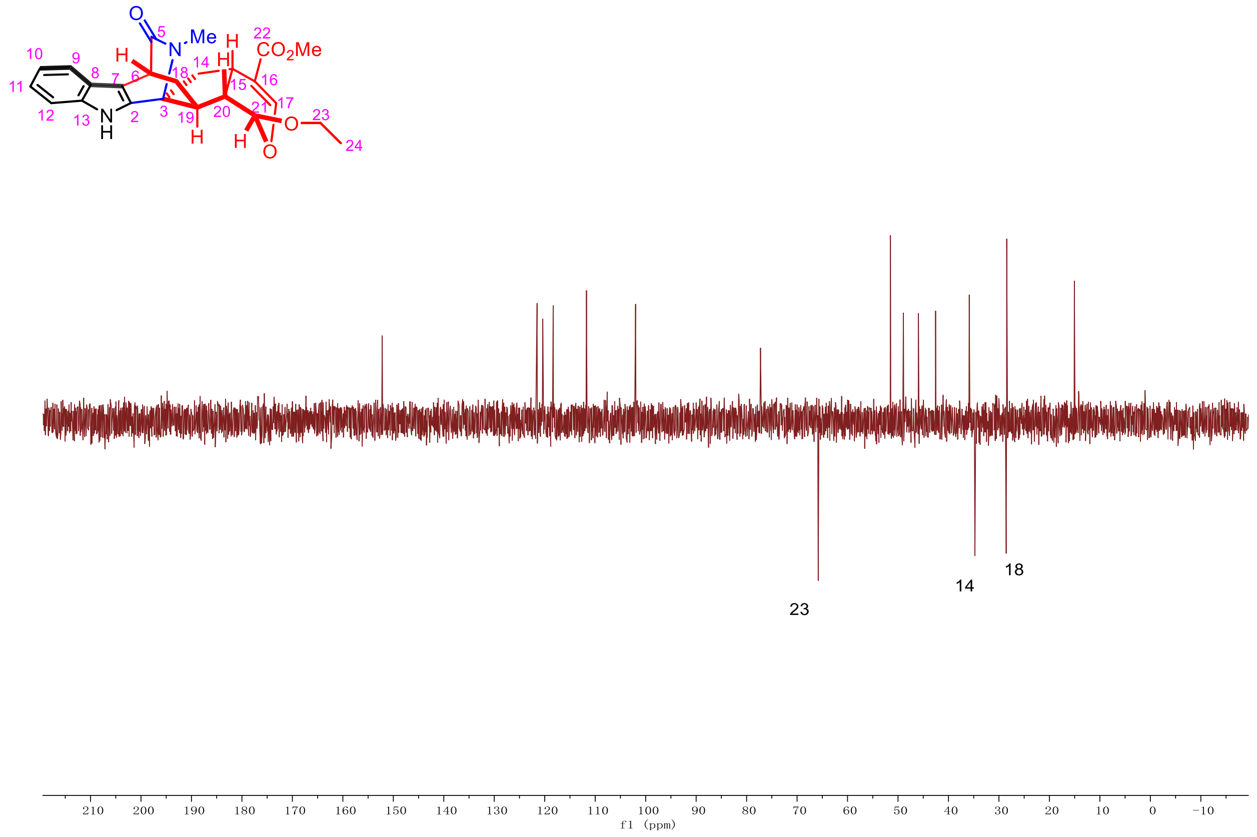

HMBC (400 MHz, $CDCl_3$), **(±)-53**

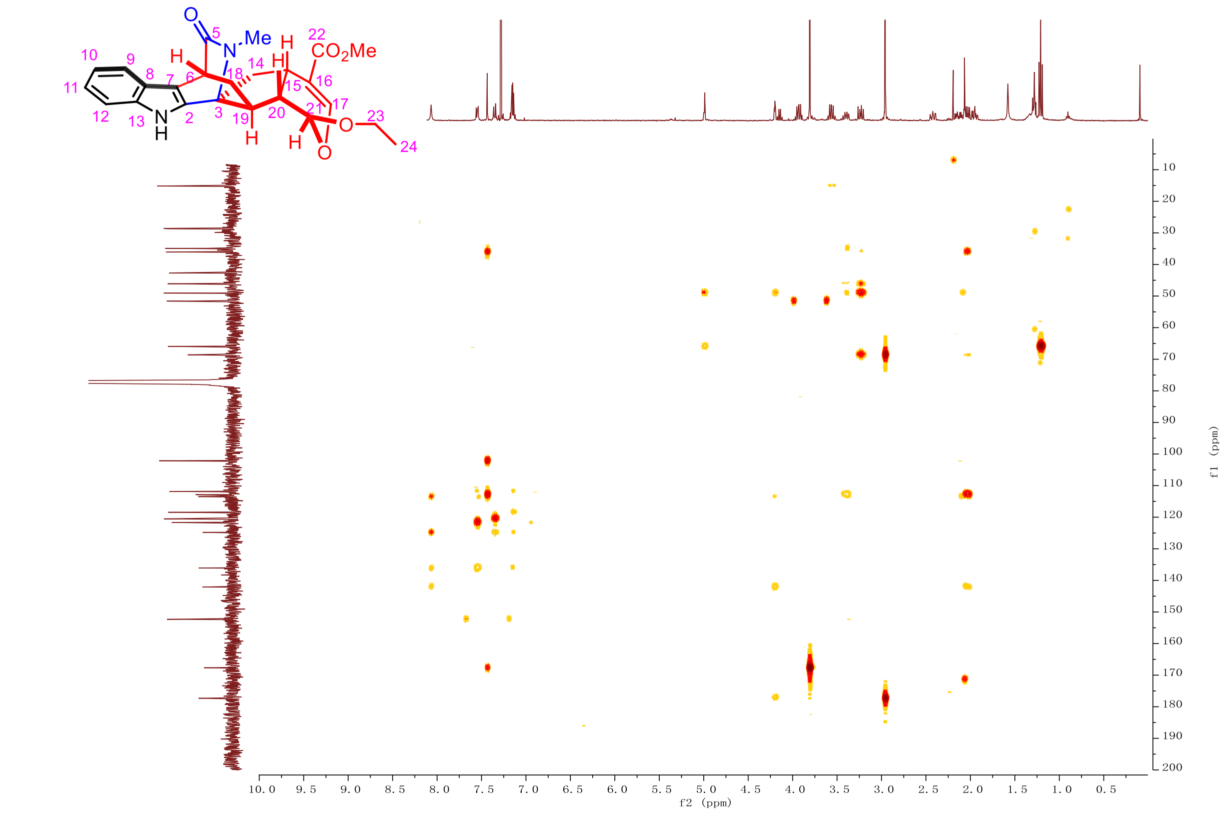

ROESY (600 MHz, $CDCl_3$), **(±)-53**

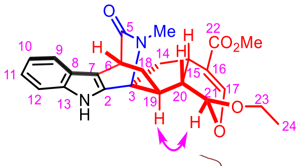

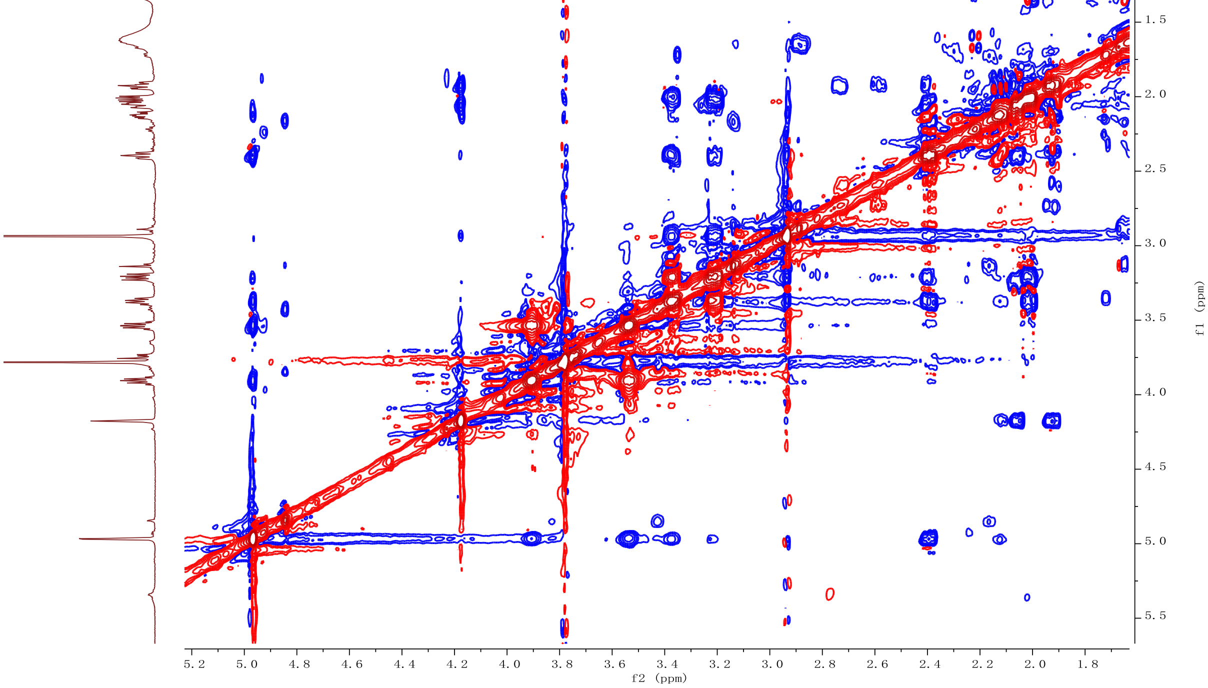

^{1}H NMR (360 MHz, $CDCl_3$), **(−)-24**

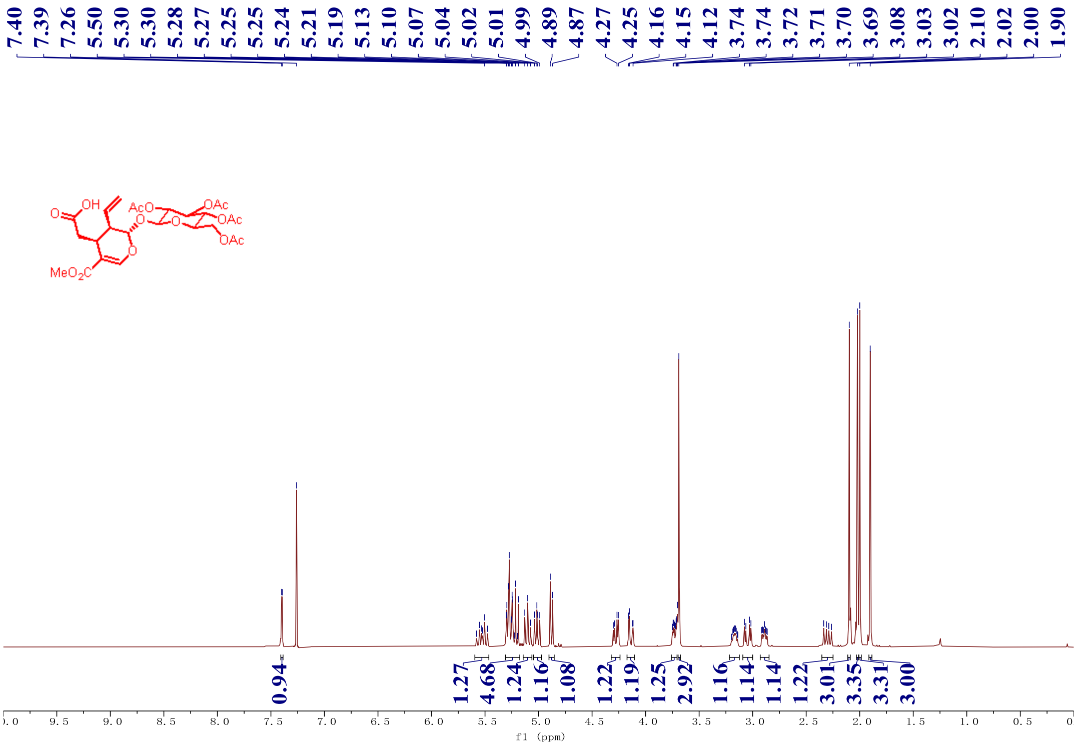

^{13}C NMR (90 MHz, $CDCl_3$), **(−)-24**

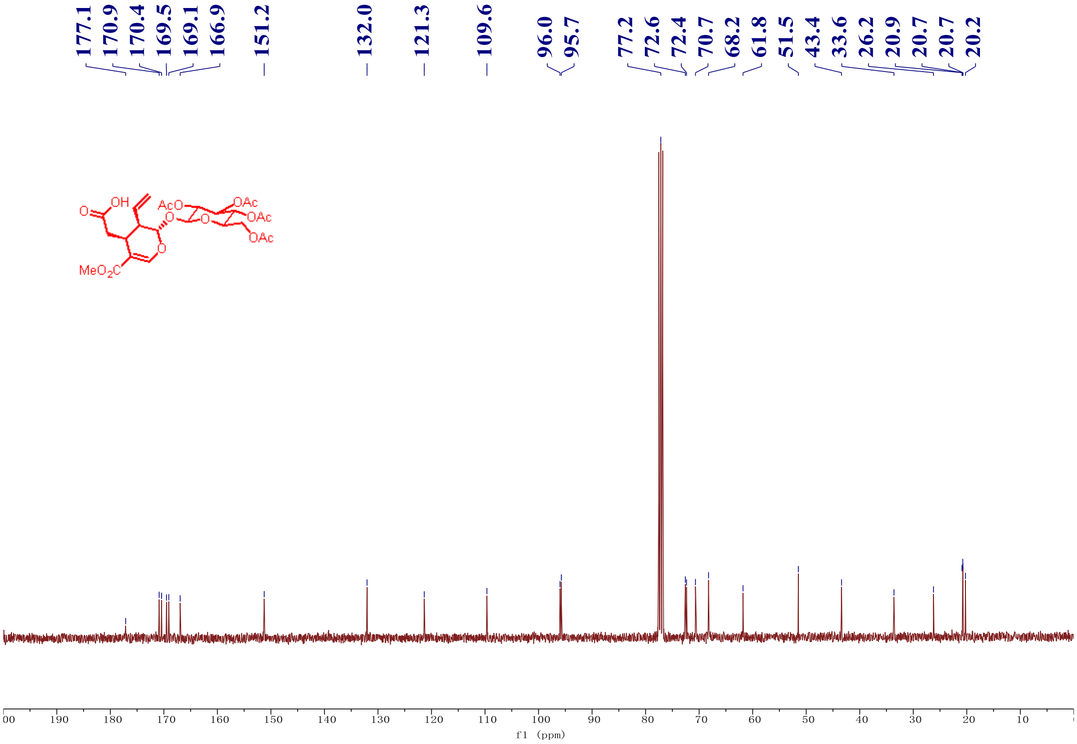

^{1}H NMR (360 MHz, $CDCl_3$), **(−)-50b**

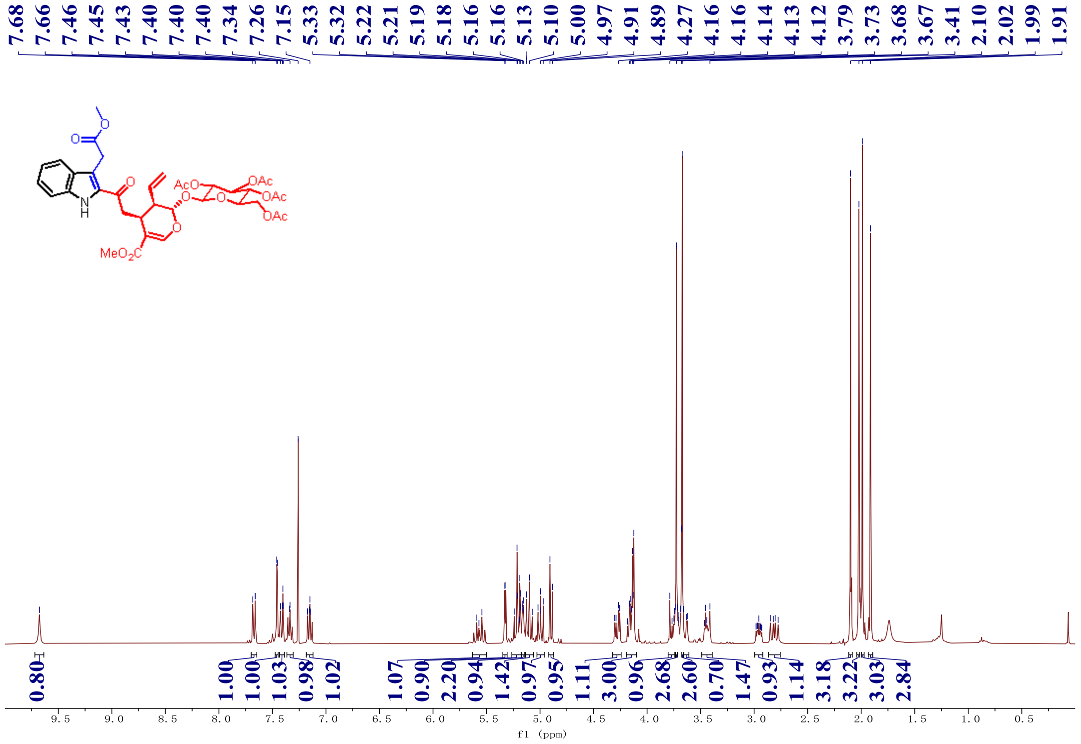

^{13}C NMR (90 MHz, $CDCl_3$), **(−)-50b**

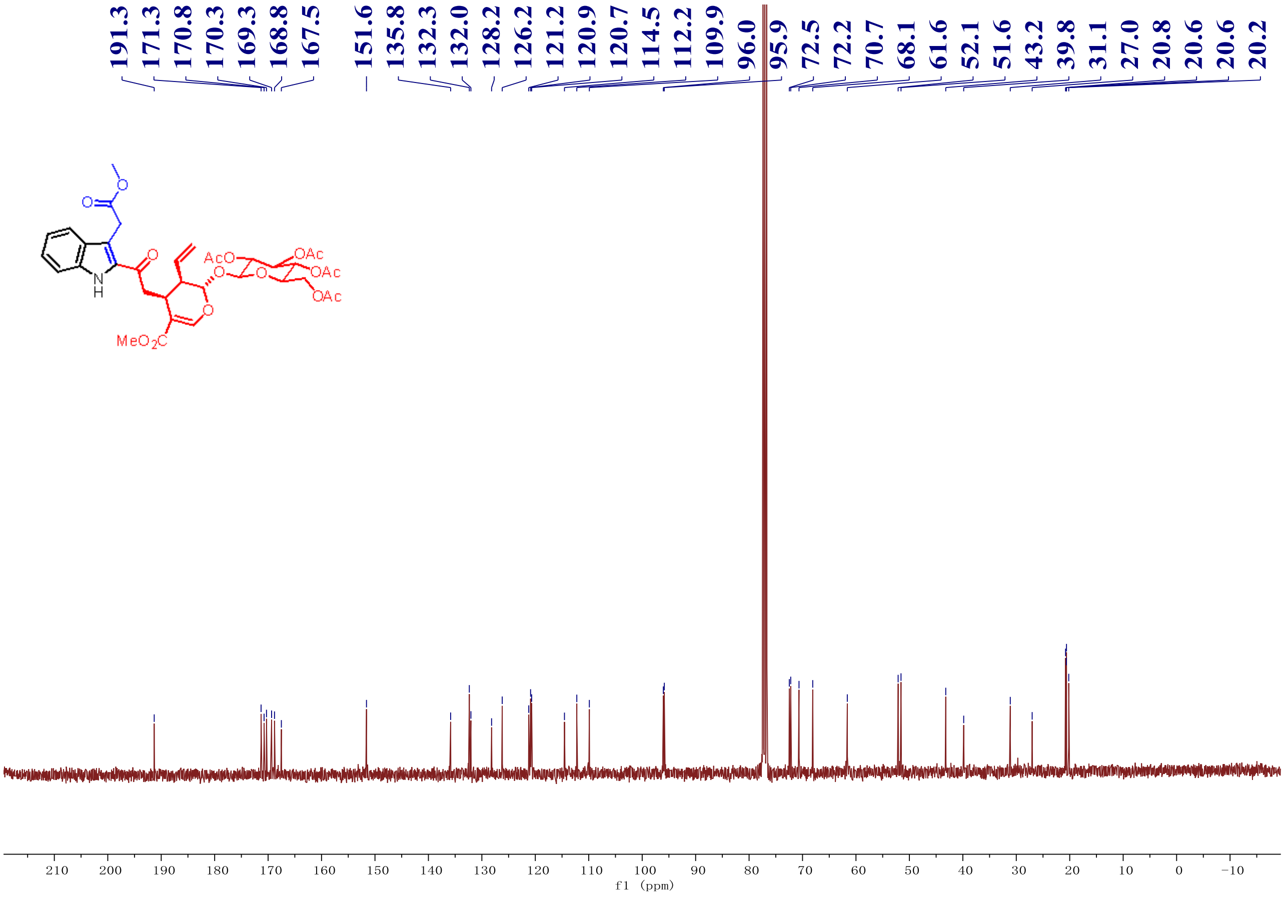

^{1}H NMR (360 MHz, $CDCl_3$), **(−)-50a**

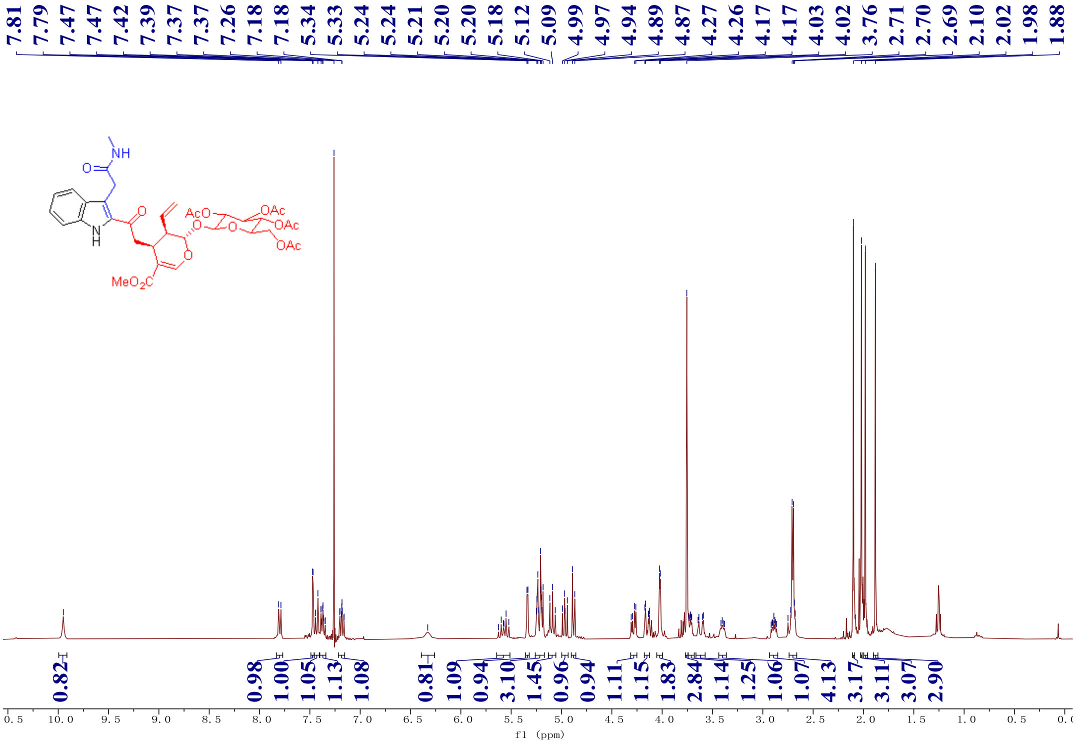

^{13}C NMR (90 MHz, $CDCl_3$), **(−)-50a**

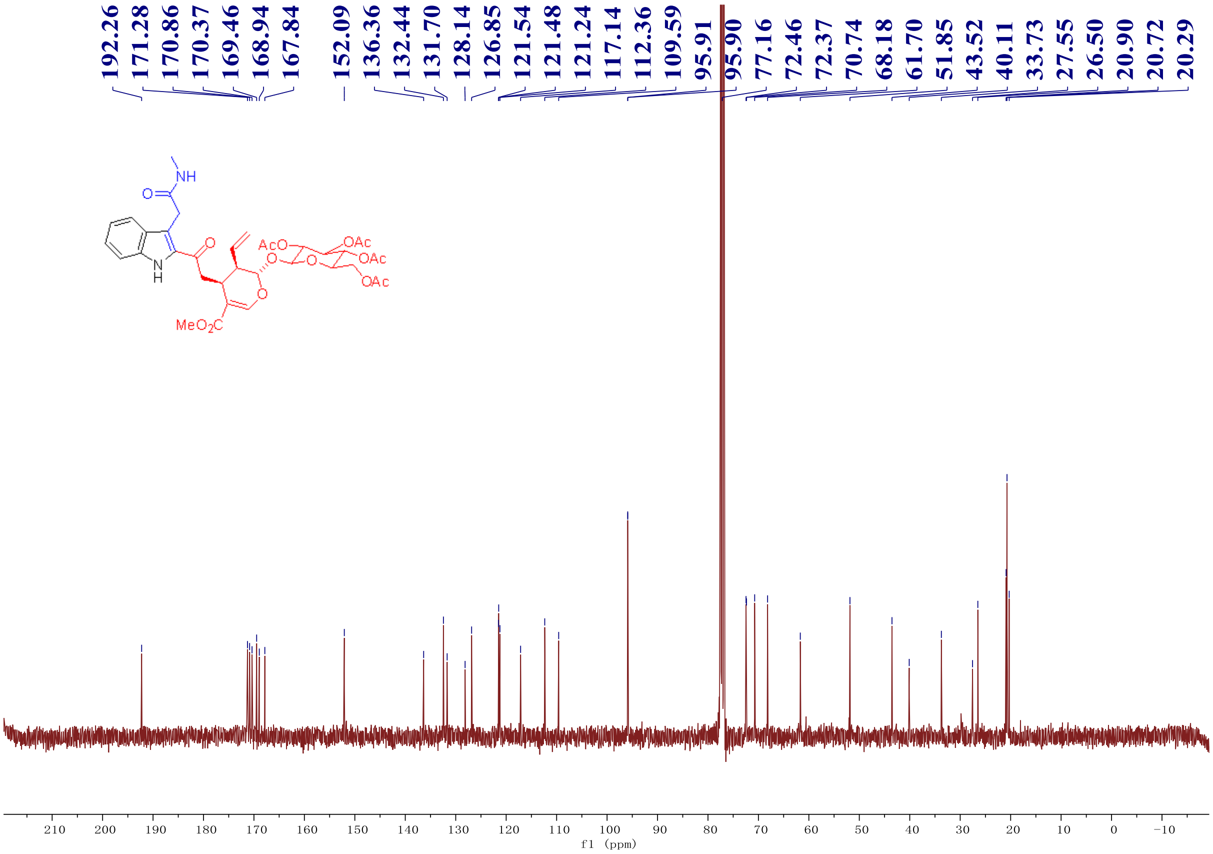

^{1}H NMR (400 MHz, CD_3OD), (−)-**7a**

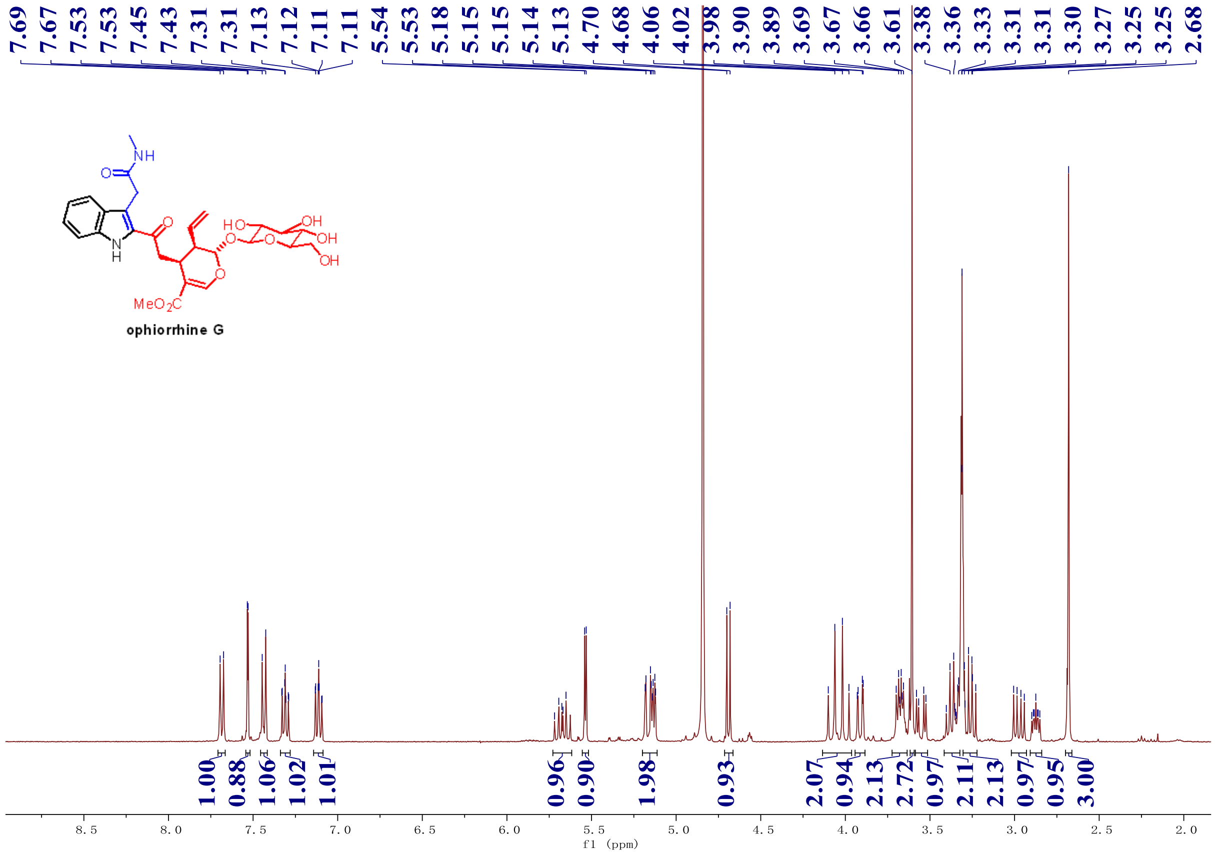

^{13}C NMR (100 MHz, CD$_3$OD), (−)-**7a**

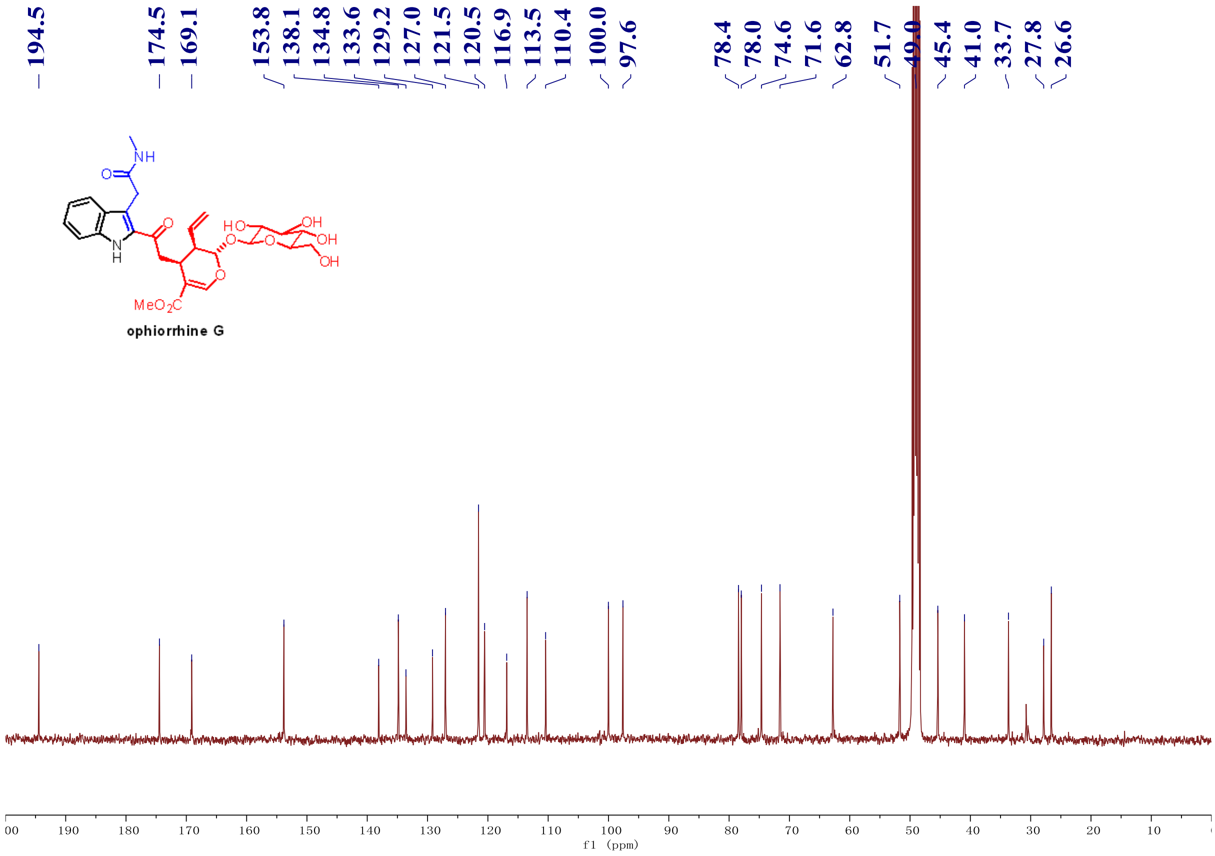

DEPT (400 MHz, CD$_3$OD), (−)-**7a**

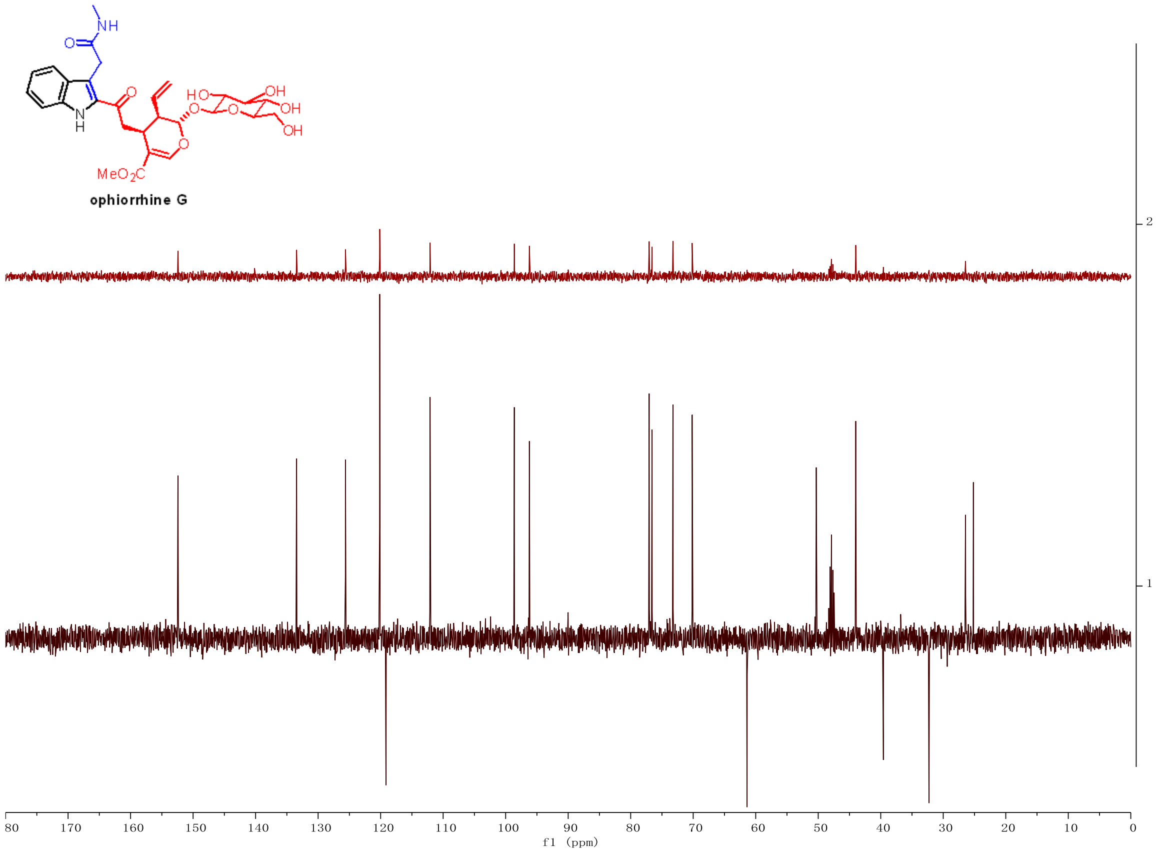

COSY (400 MHz, CD_3OD), (−)-**7a**

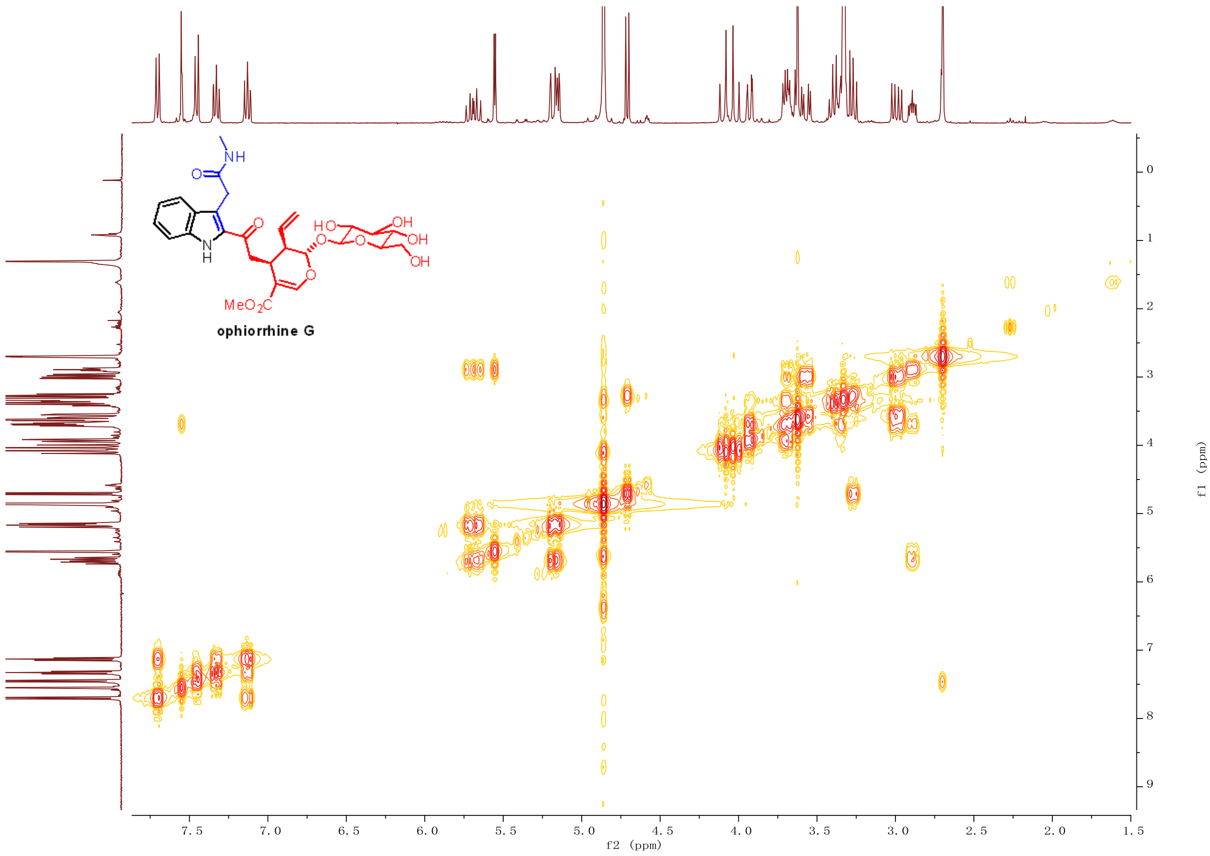

HSQC (400 MHz, CD_3OD), (−)-**7a**

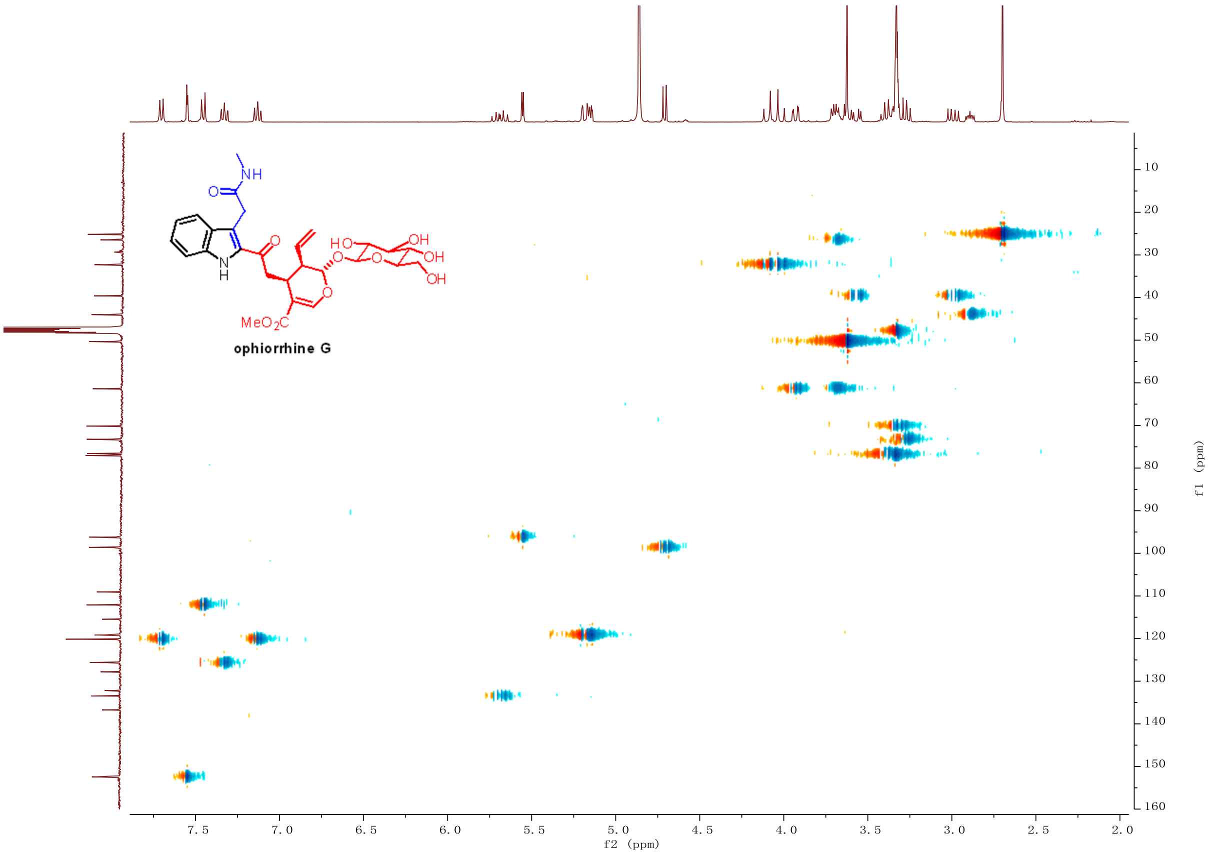

HMBC (400 MHz, CD3OD), (−)-**7a**

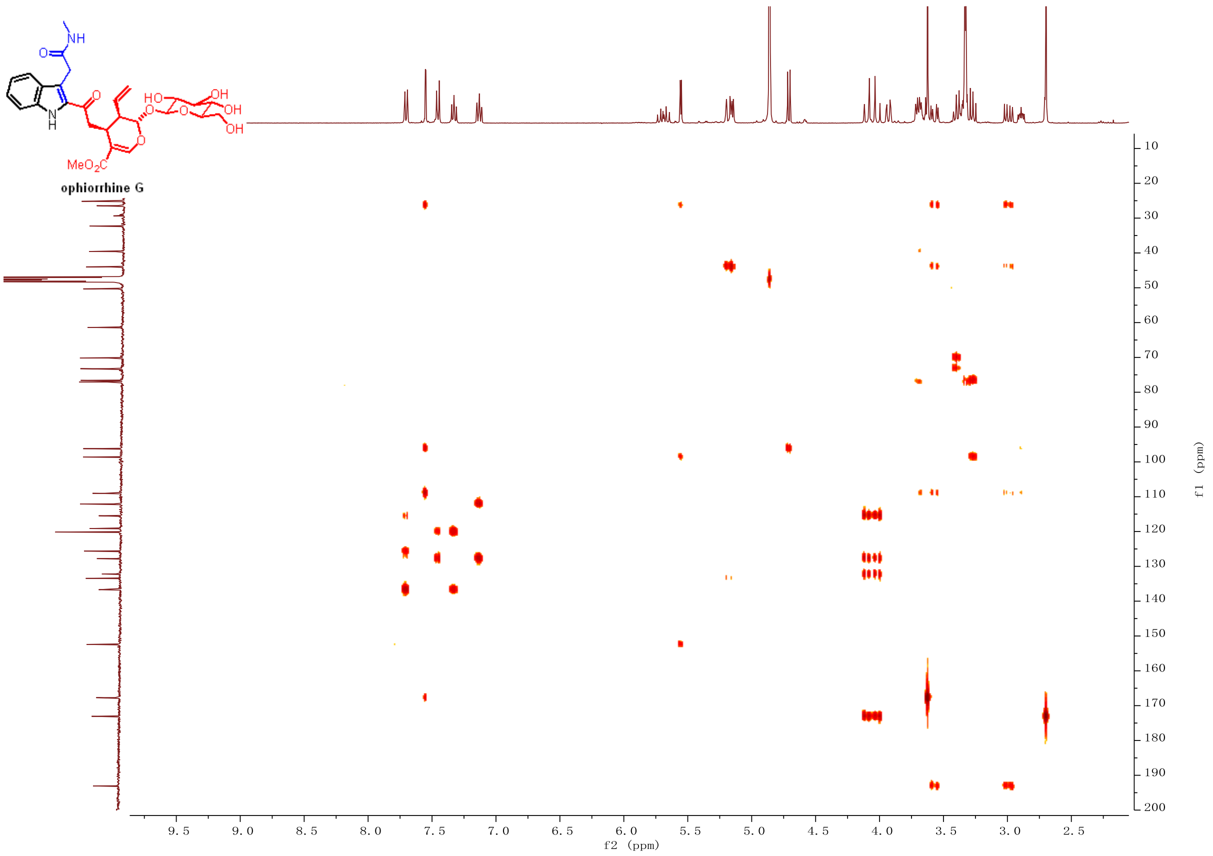

NOESY (400 MHz, CD3OD), (−)-**7a**

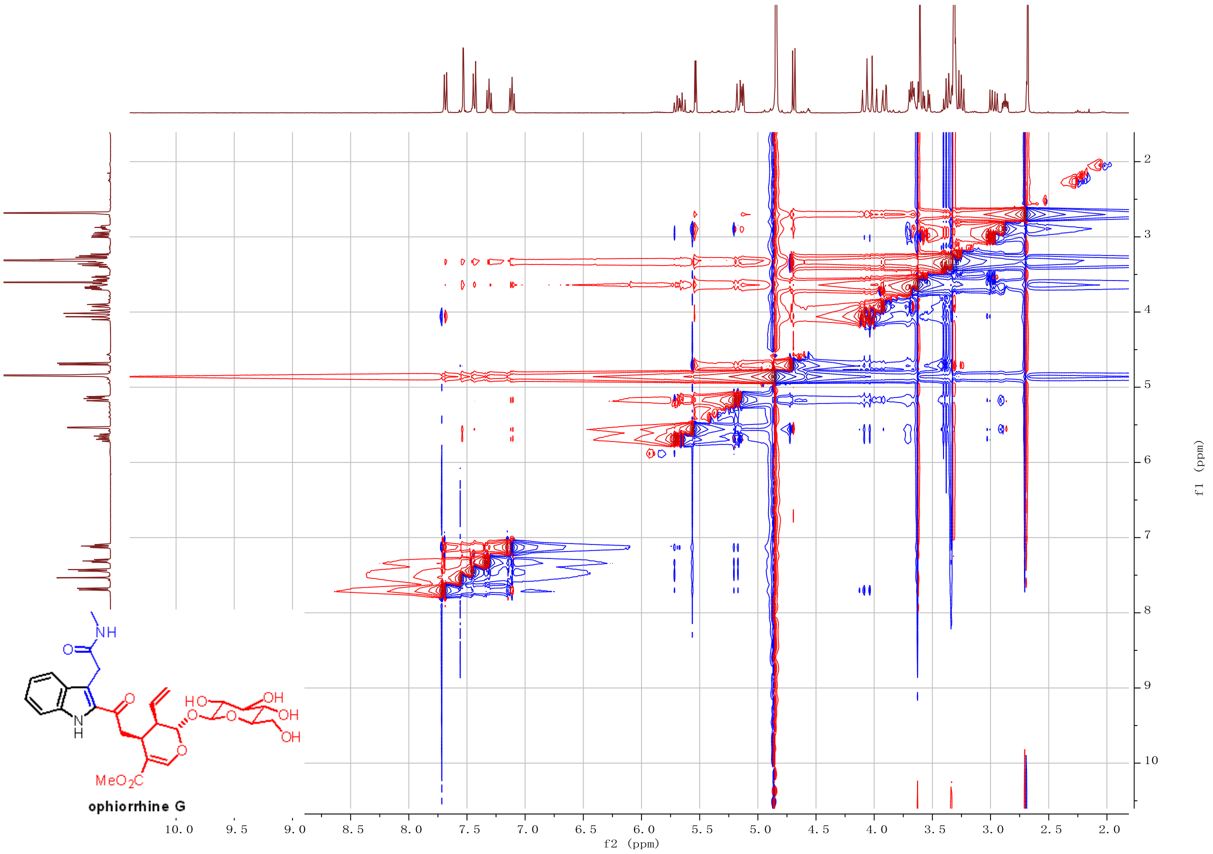

ophiorrhine G
f2 (ppm)
f1 (ppm)

^{1}H NMR (400 MHz, CD_3OD), (−)-**6a**

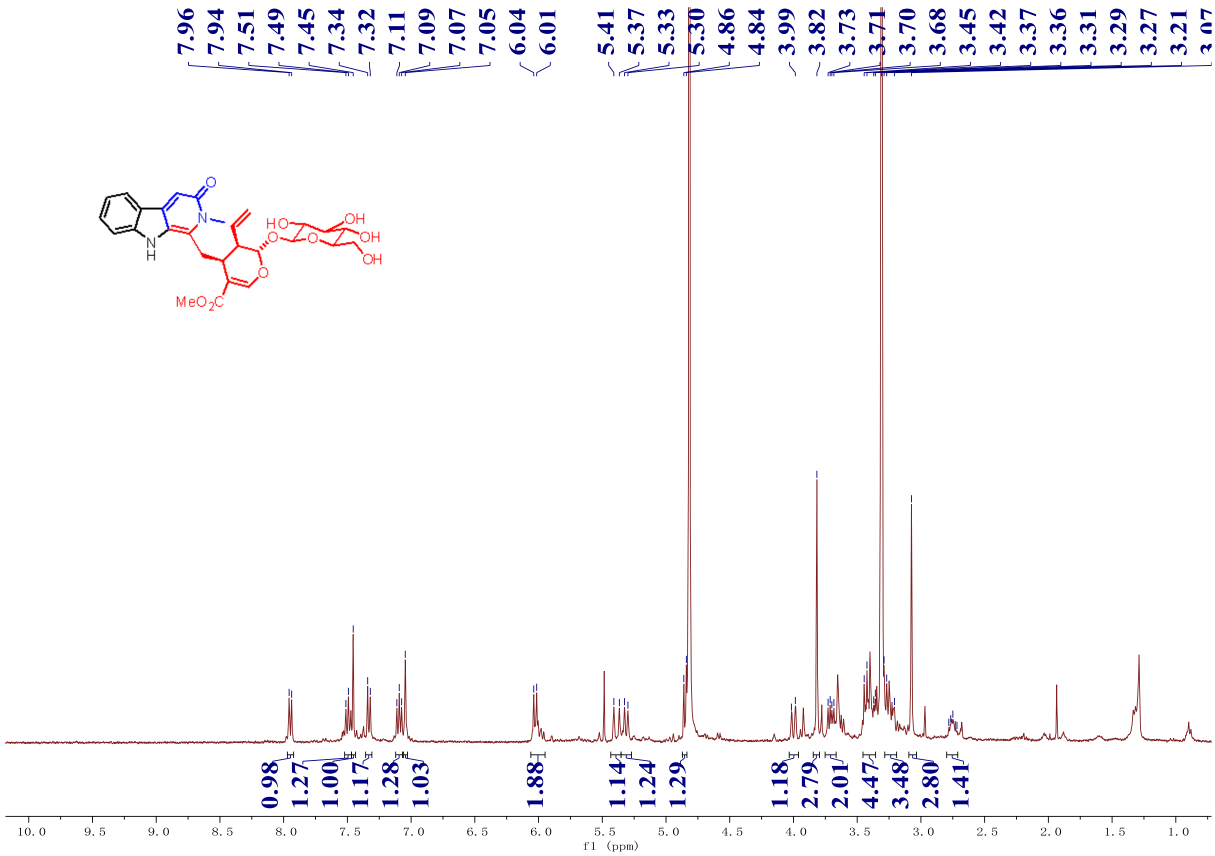

^{13}C NMR (100 MHz, CD_3OD), (−)-**6a**

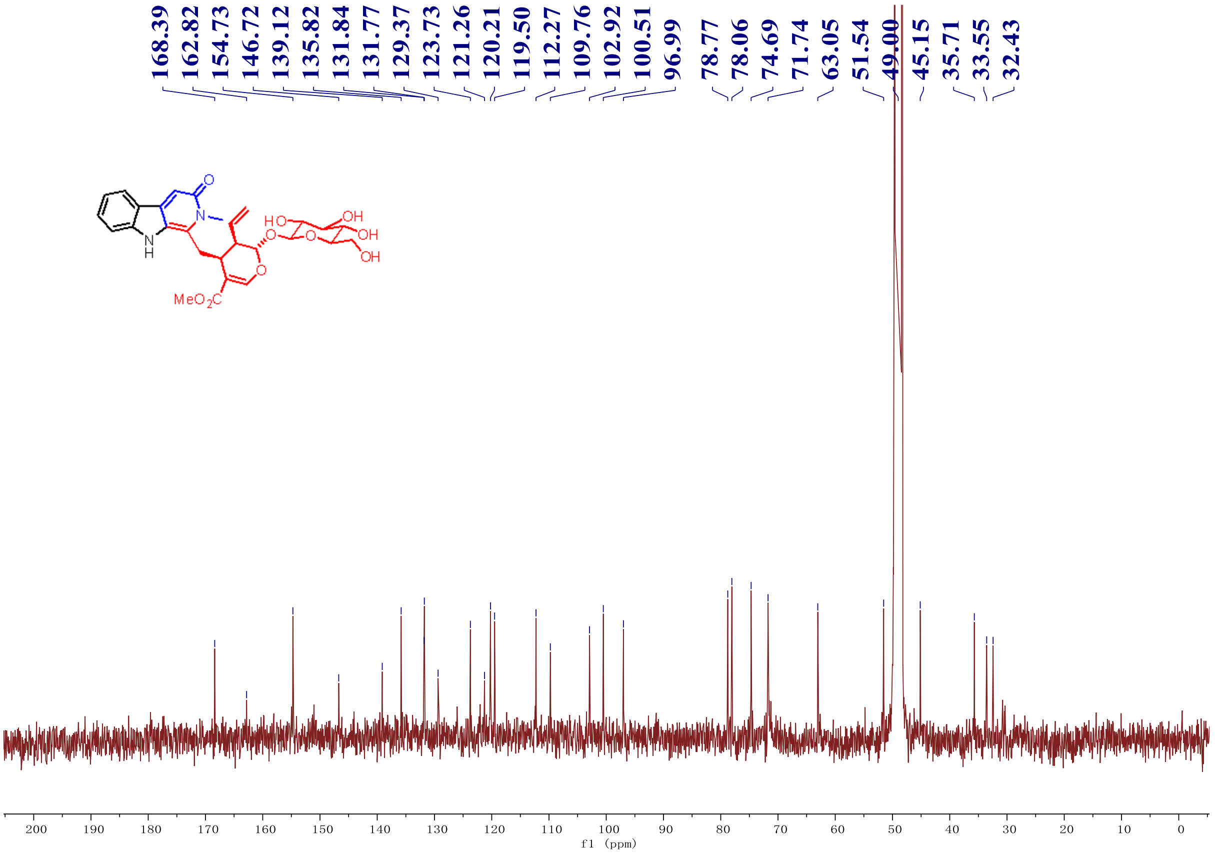

DEPT (400 MHz, CD3OD), (−)-**6a**

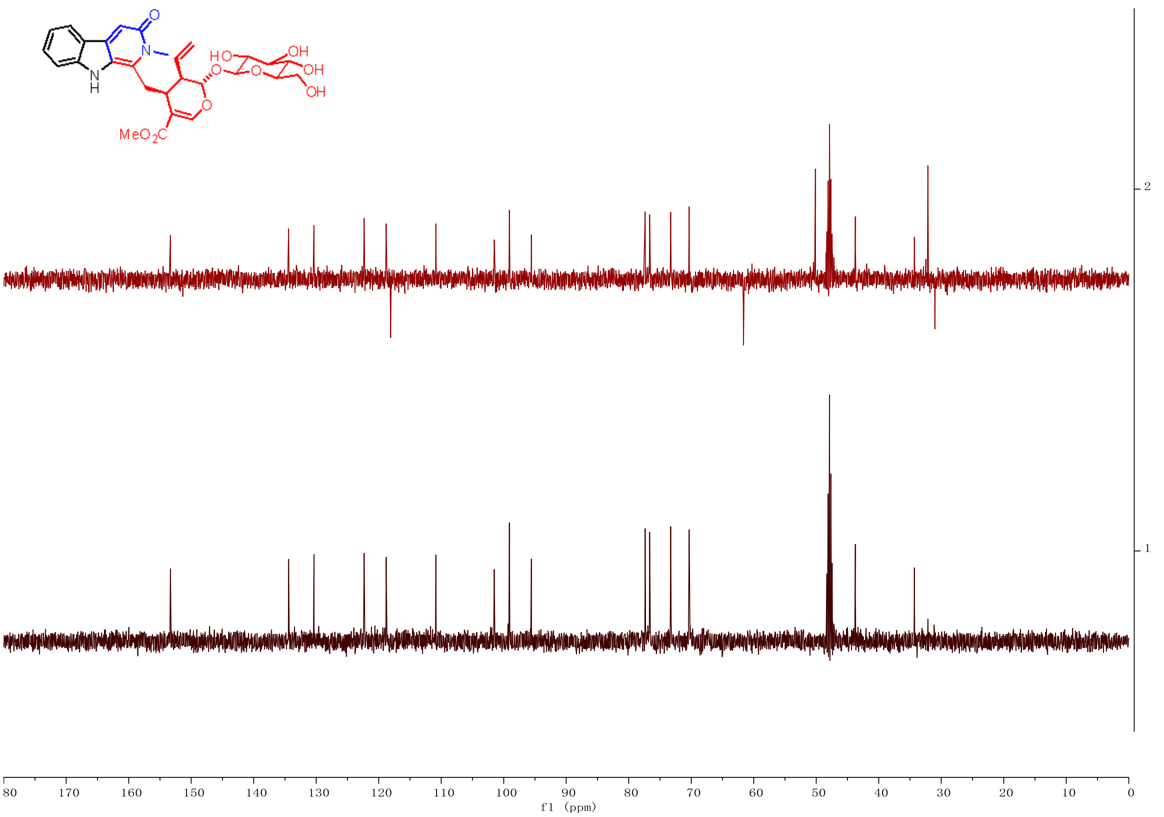

COSY (400 MHz, CD3OD), (−)-**6a**

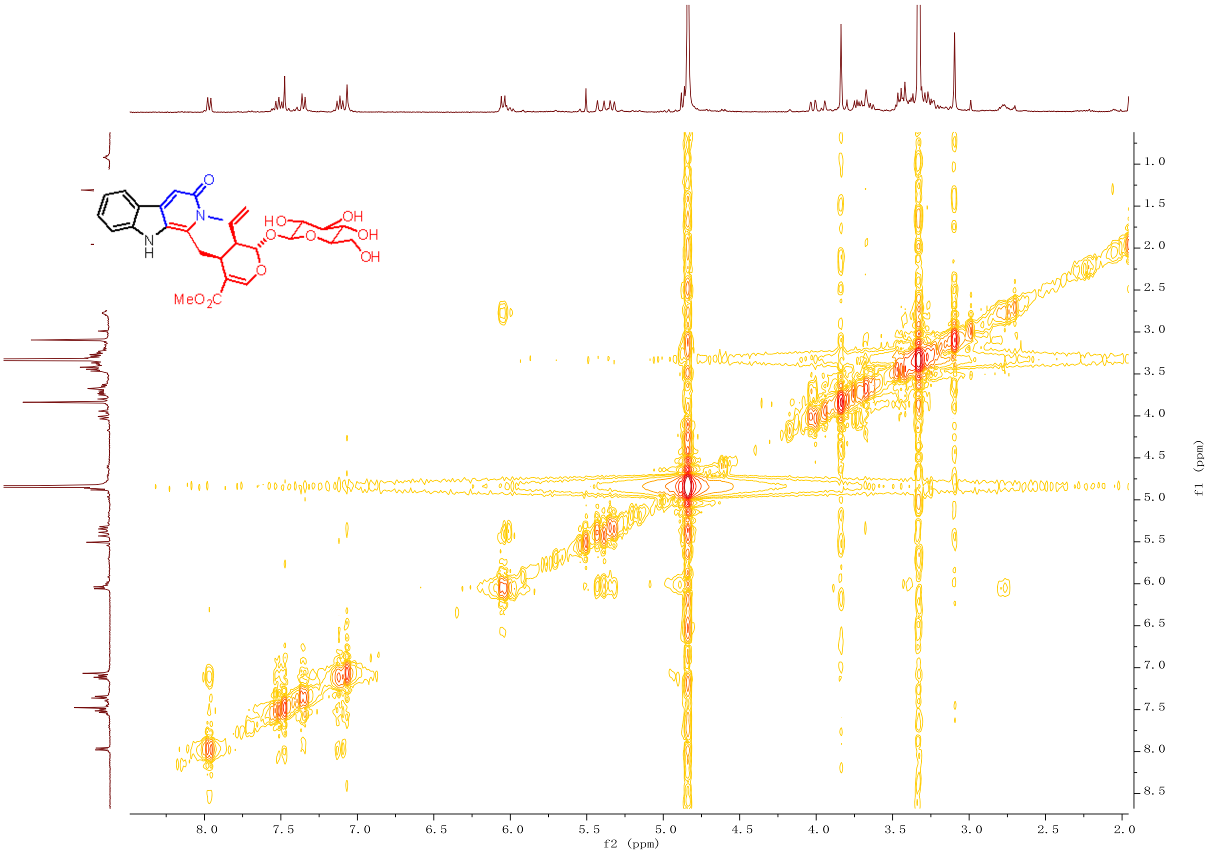

HSQC (360 MHz, CD_3OD), (−)-**6a**

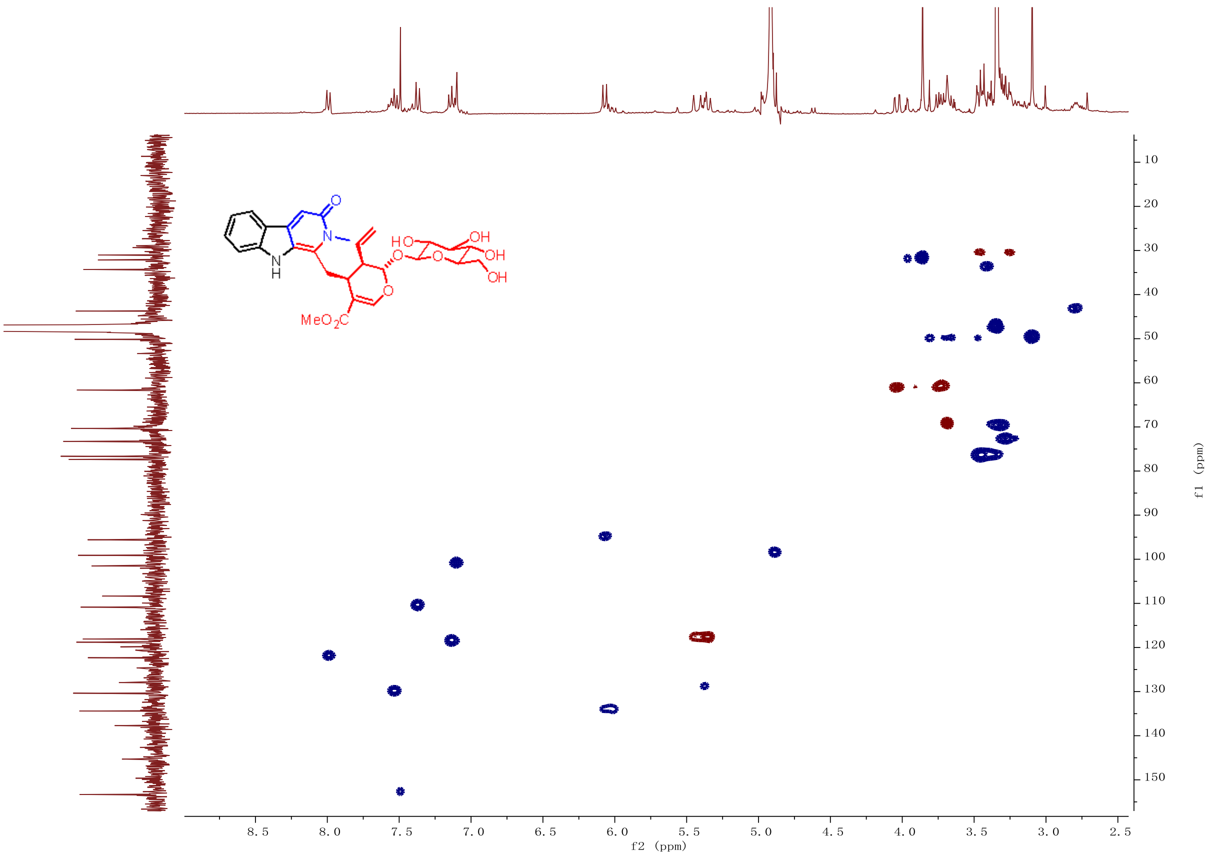

HMBC (400 MHz, CD_3OD), (−)-**6a**

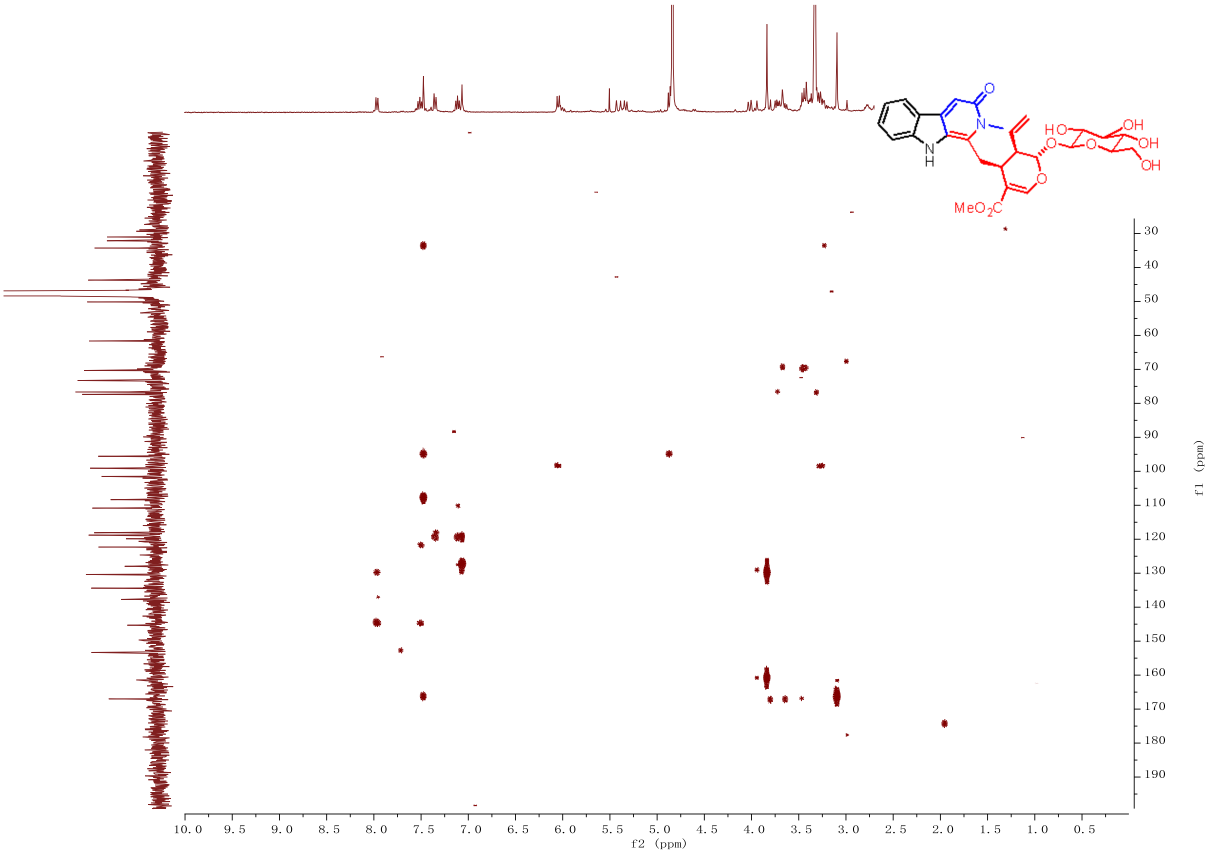

^{1}H NMR (400 MHz, CDCl3), **(−)-54**

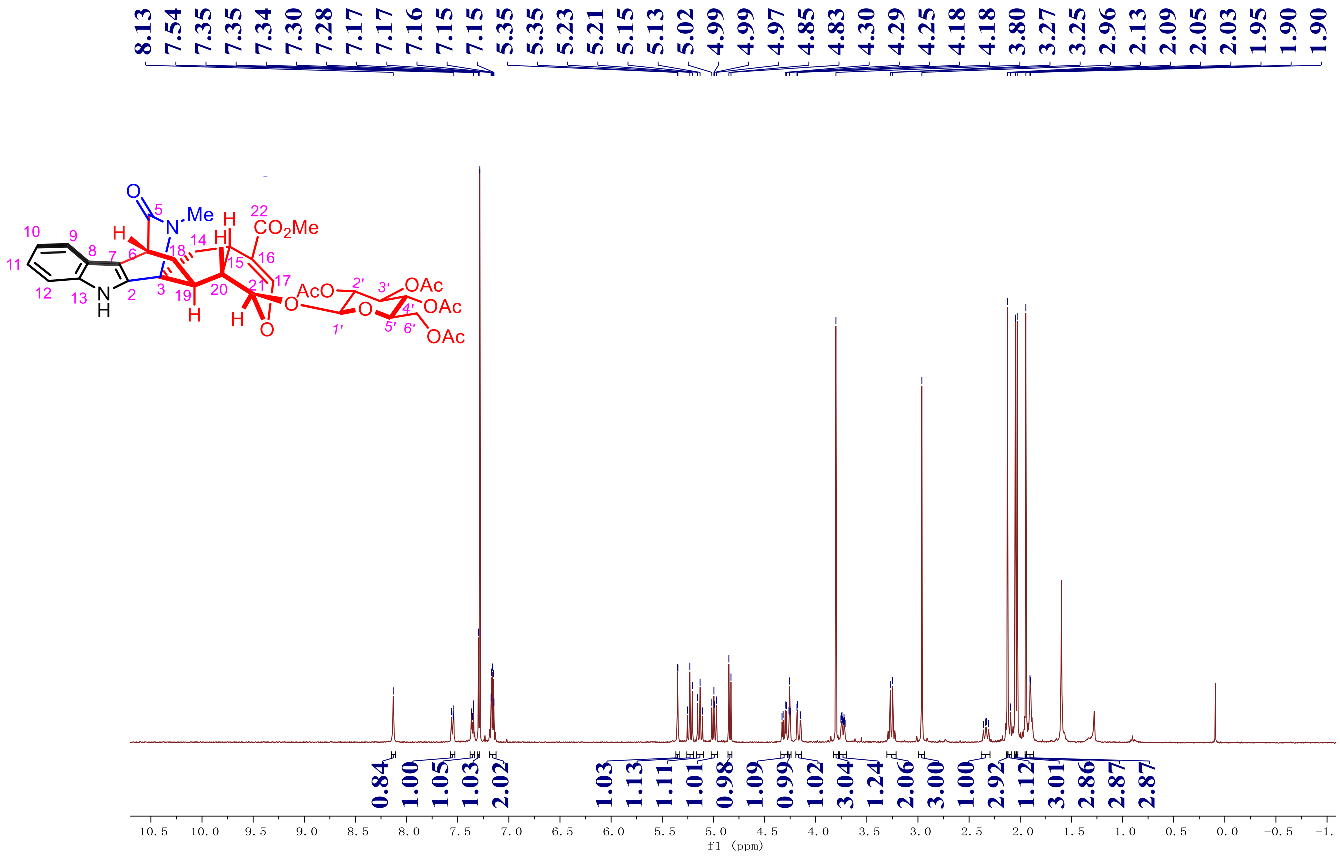

^{13}C NMR (100 MHz, CDCl3), **(−)-54**

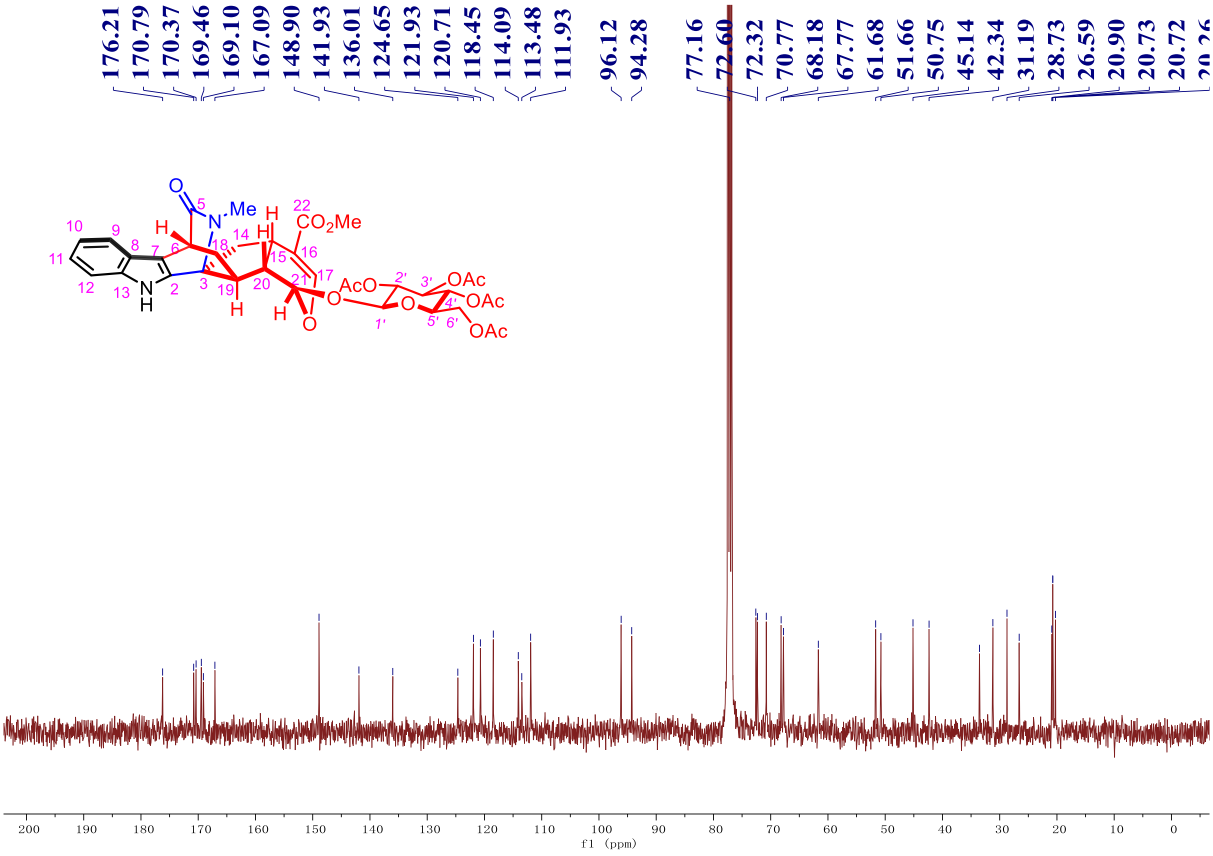

^{1}H NMR (360 MHz, $CDCl_3$), **(+)-55**

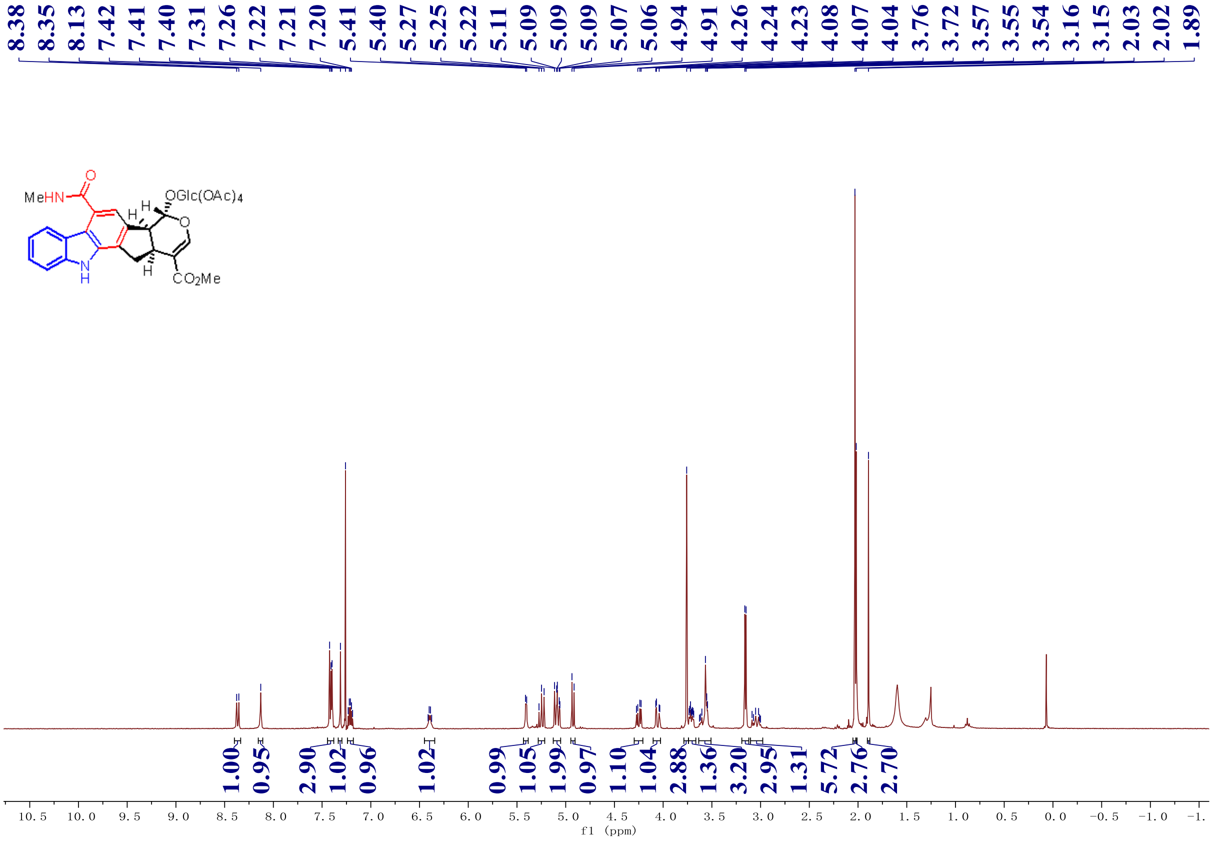

^{13}C NMR (360 MHz, $CDCl_3$), **(+)-55**

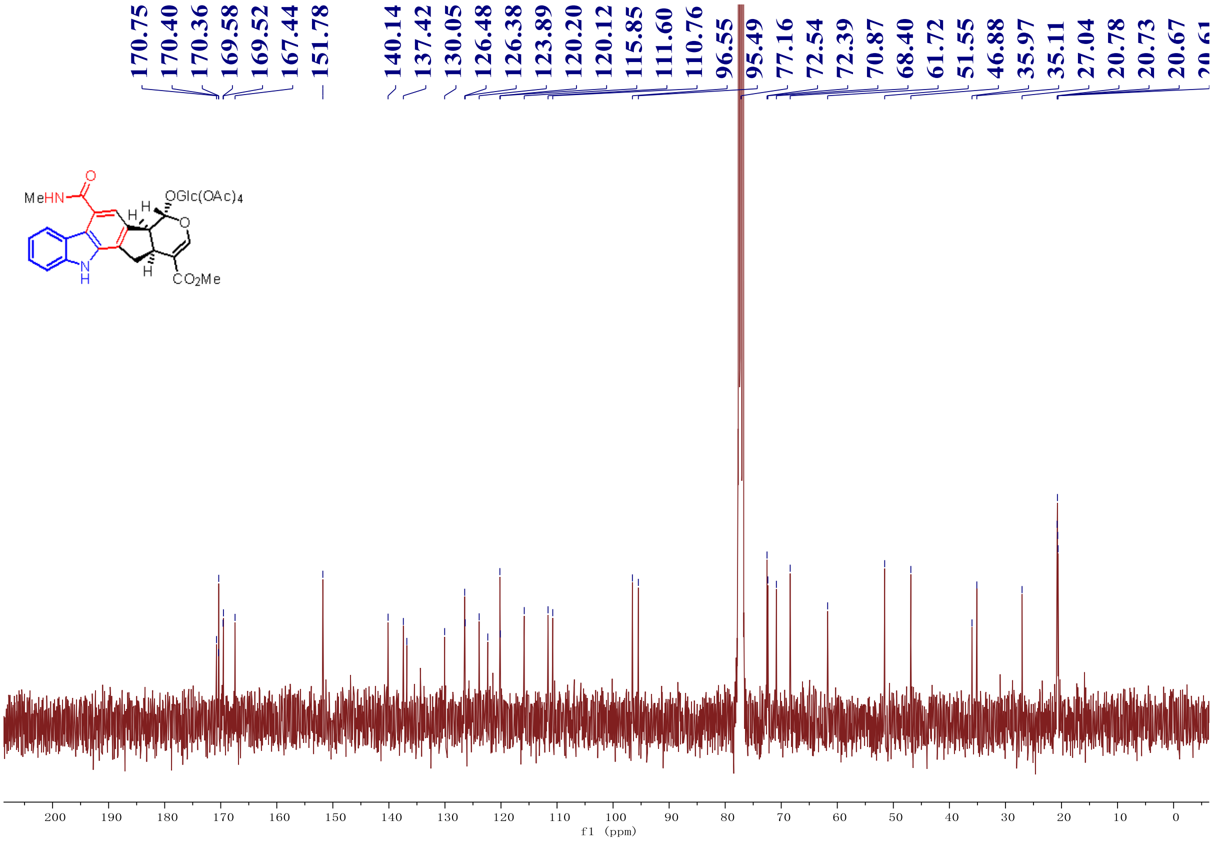

^{1}H NMR (400 MHz, CD_3OD), **(+)-56**

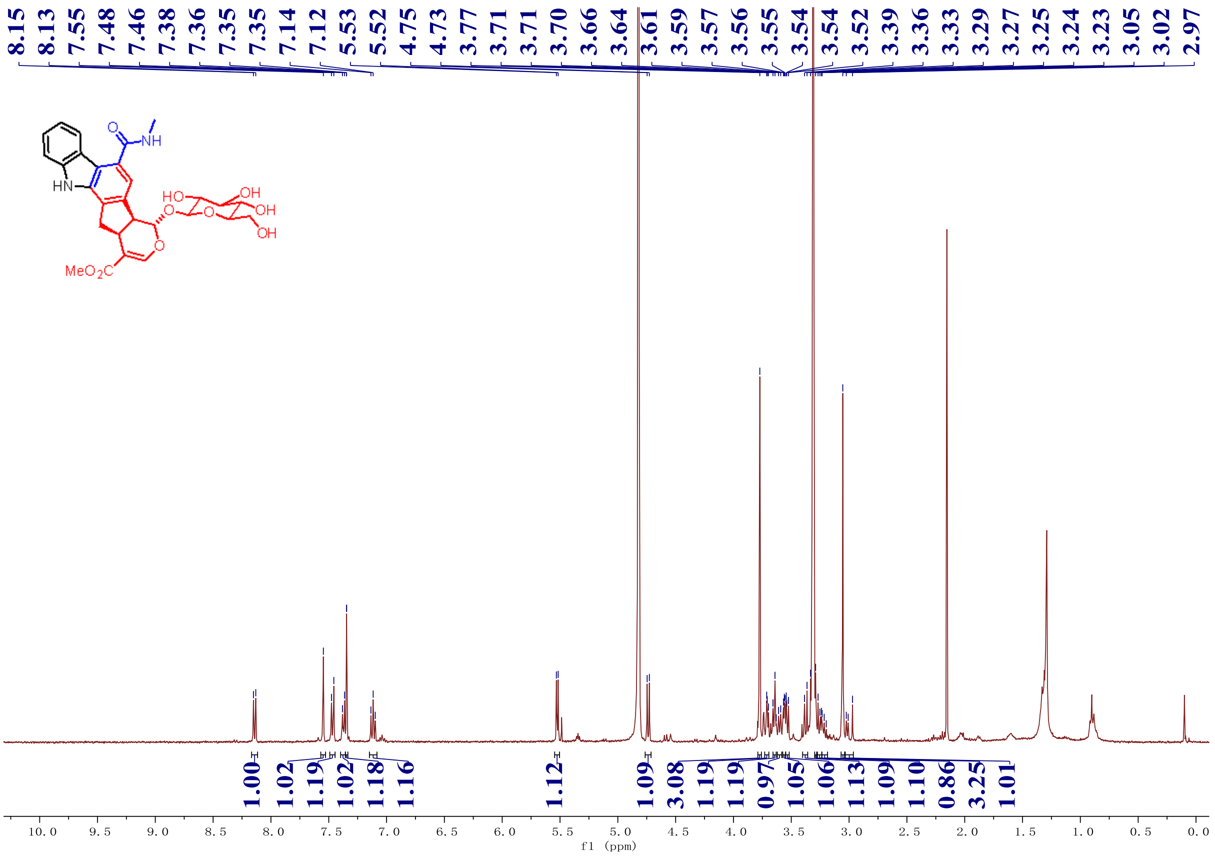

^{13}C NMR (100 MHz, CD_3OD), **(+)-56**

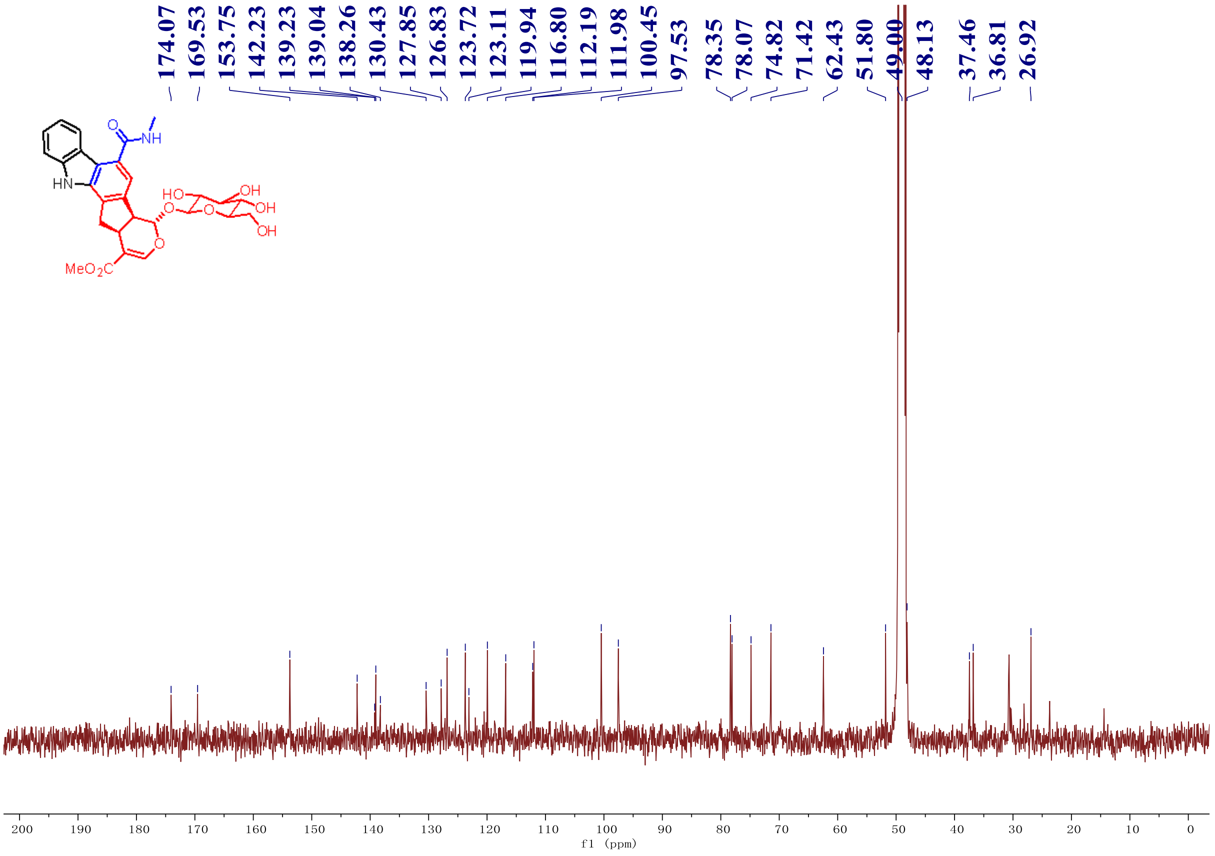

DEPT (400 MHz, CD3OD), **(+)-56**

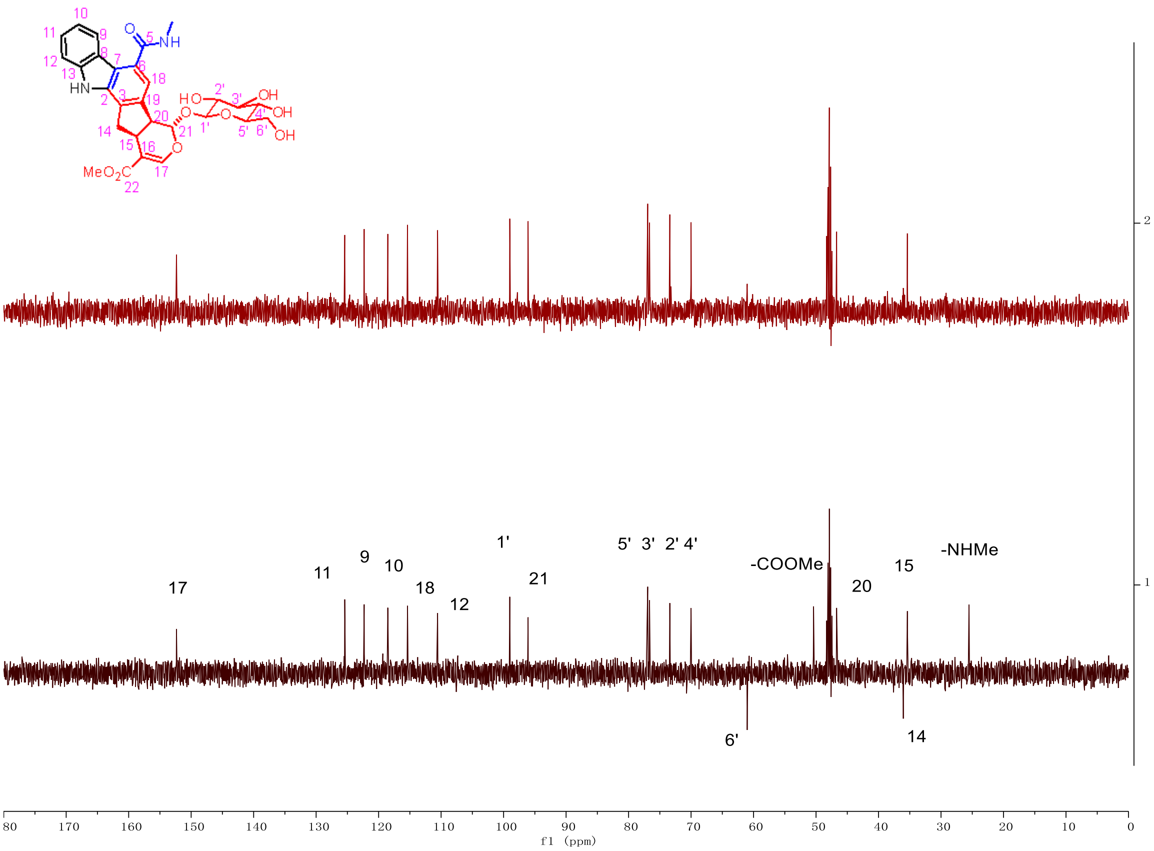

COSY (360 MHz, CD3OD), **(+)-56**

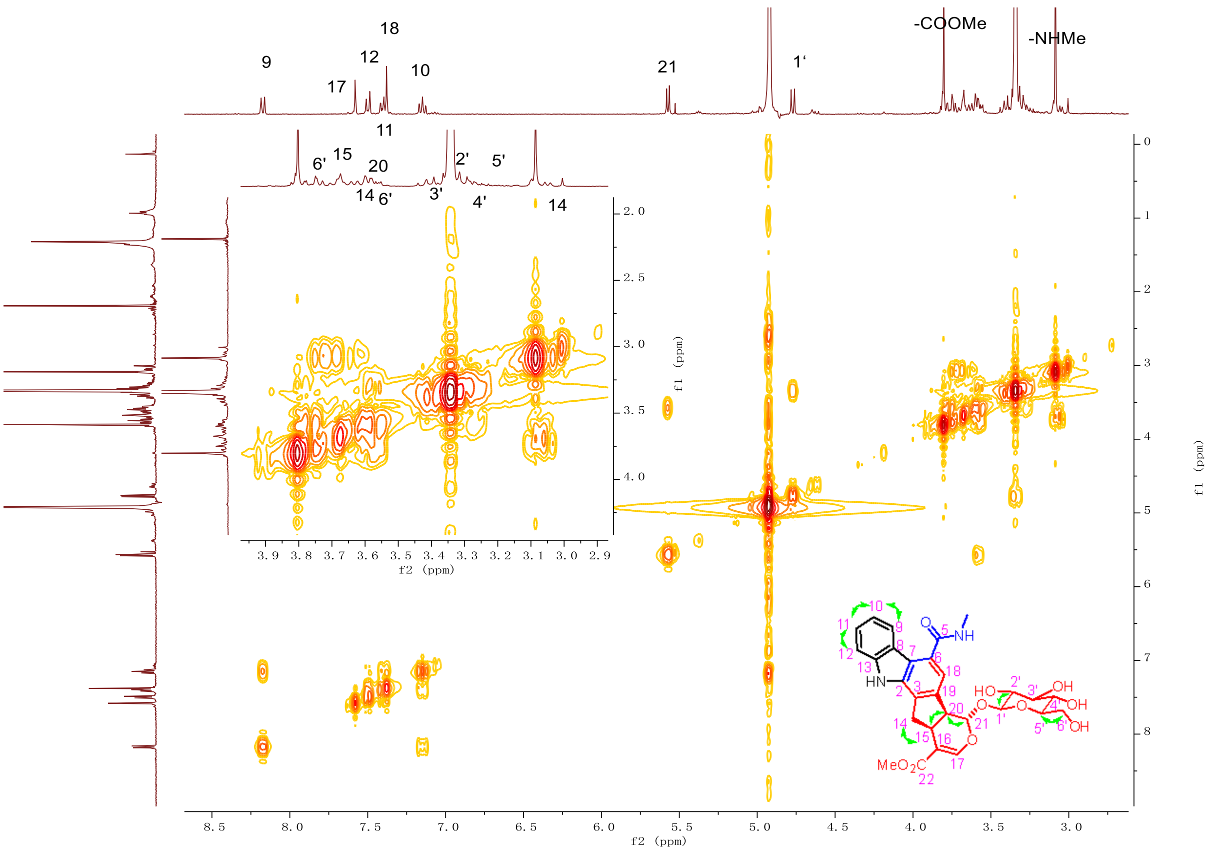

HSQC (360 MHz, CD_3OD), **(+)-56**

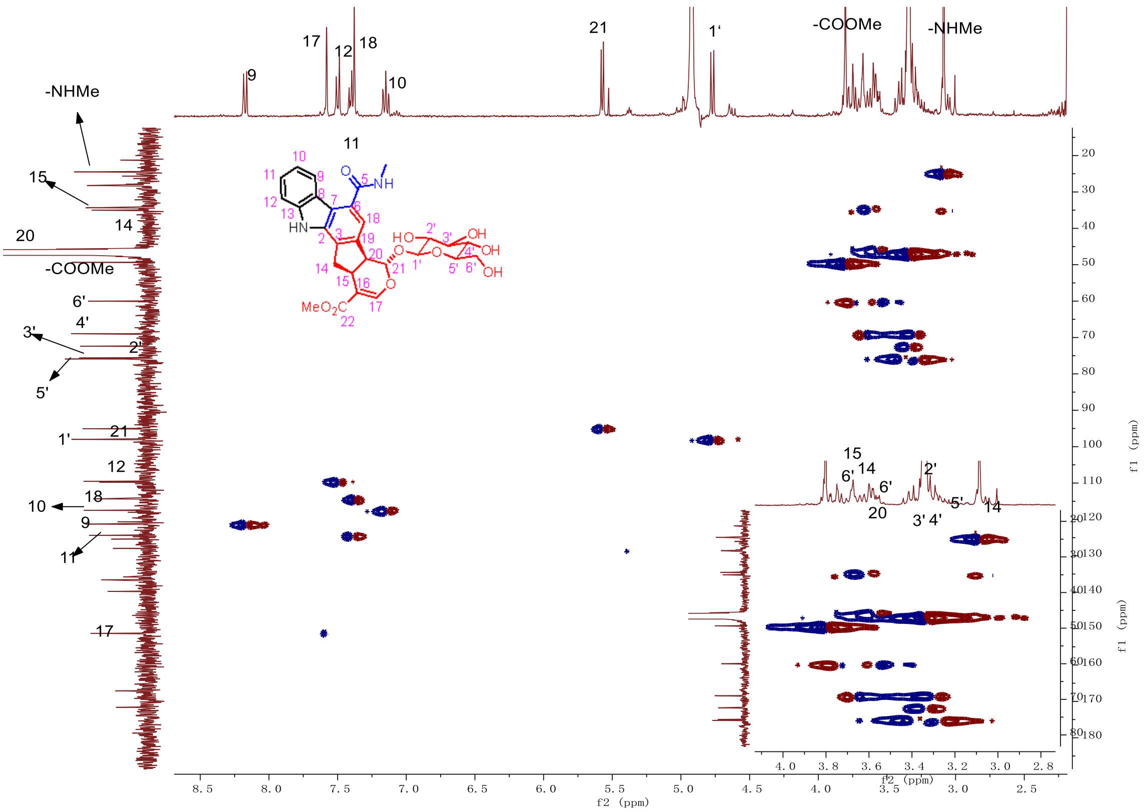

NOESY (360 MHz, CD_3OD), **(+)-56**

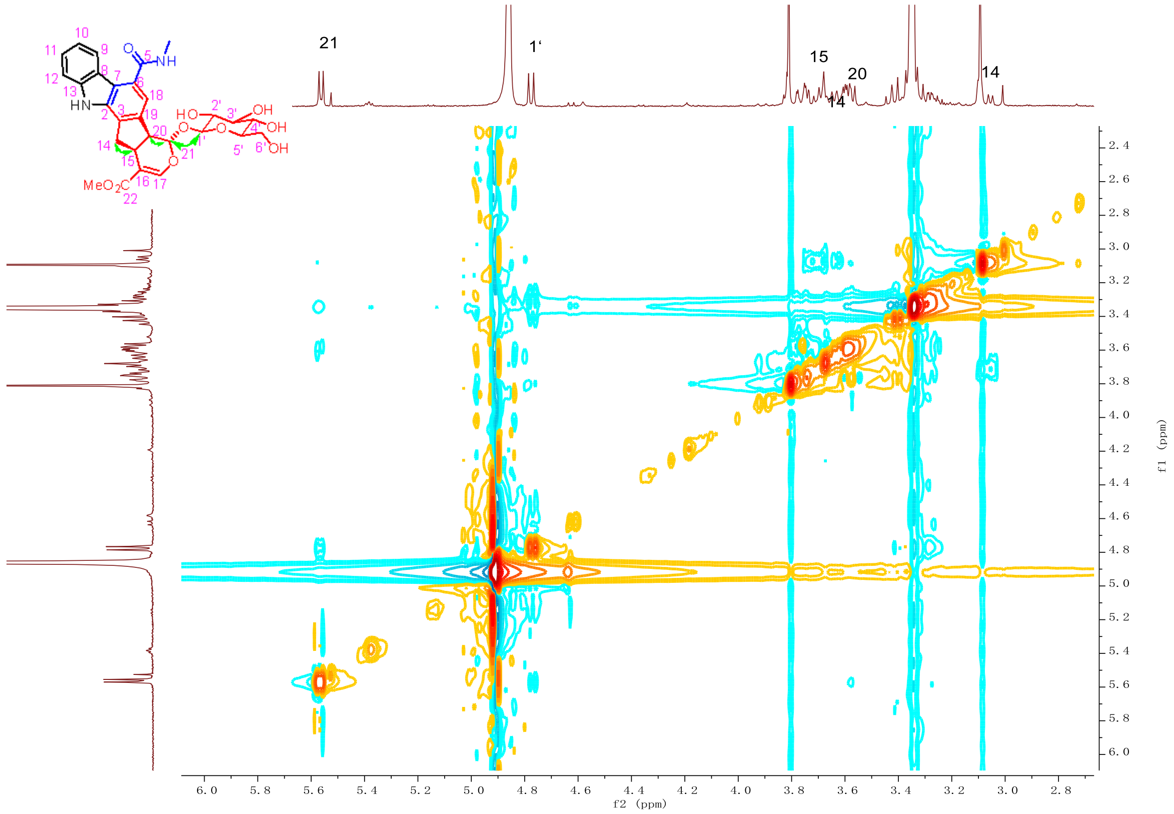

HMBC (360 MHz, CD_3OD), **(+)-56**

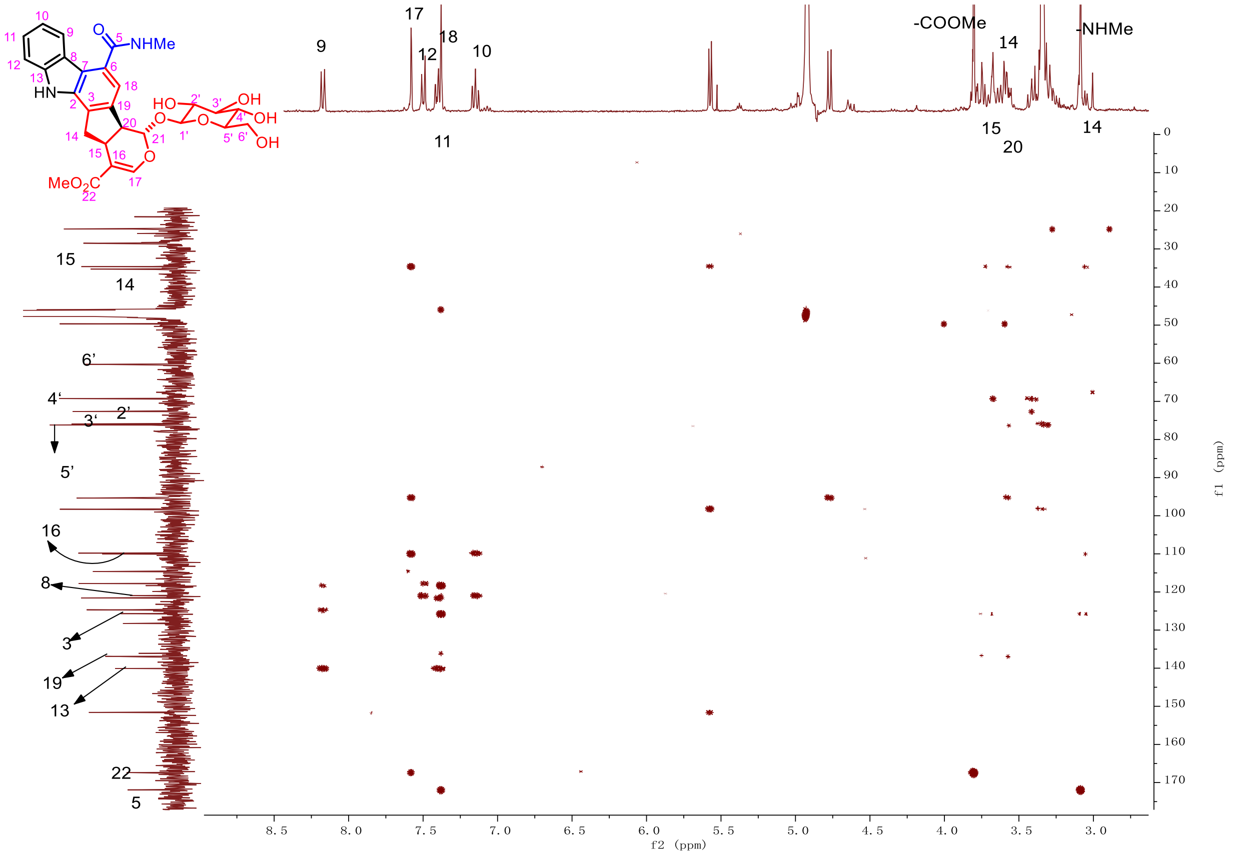

Key HMBC correlations

H14a,b (3.65 and 3.00 ppm) – **C3** (127.9 ppm)

H18 (7.35 ppm) – **C3** (127.9 ppm)

H18 (7.35 ppm) - **C5** (174.1 ppm)

H18 (7.35 ppm) - **C7** (120.5 ppm); **H9** (8.14 ppm) - **C7** (120.5 ppm)

H18 (7.35 ppm) - **C20** (48.1 ppm)

H20 (3.56 ppm) – **C19** (139.2 ppm)

^{1}H NMR (400 MHz, CD_3OD), (−)-**1a**

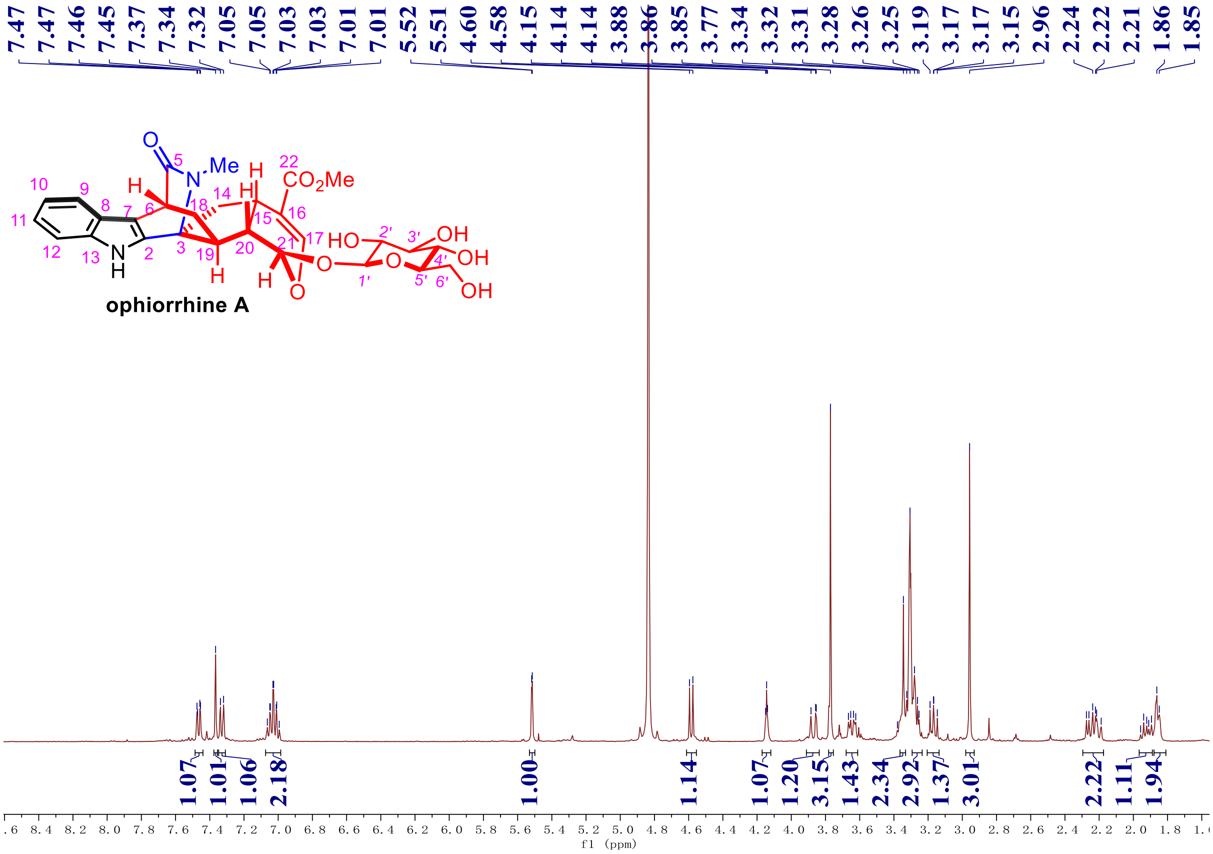

^{13}C NMR (100 MHz, CD_3OD), (−)-**1a**

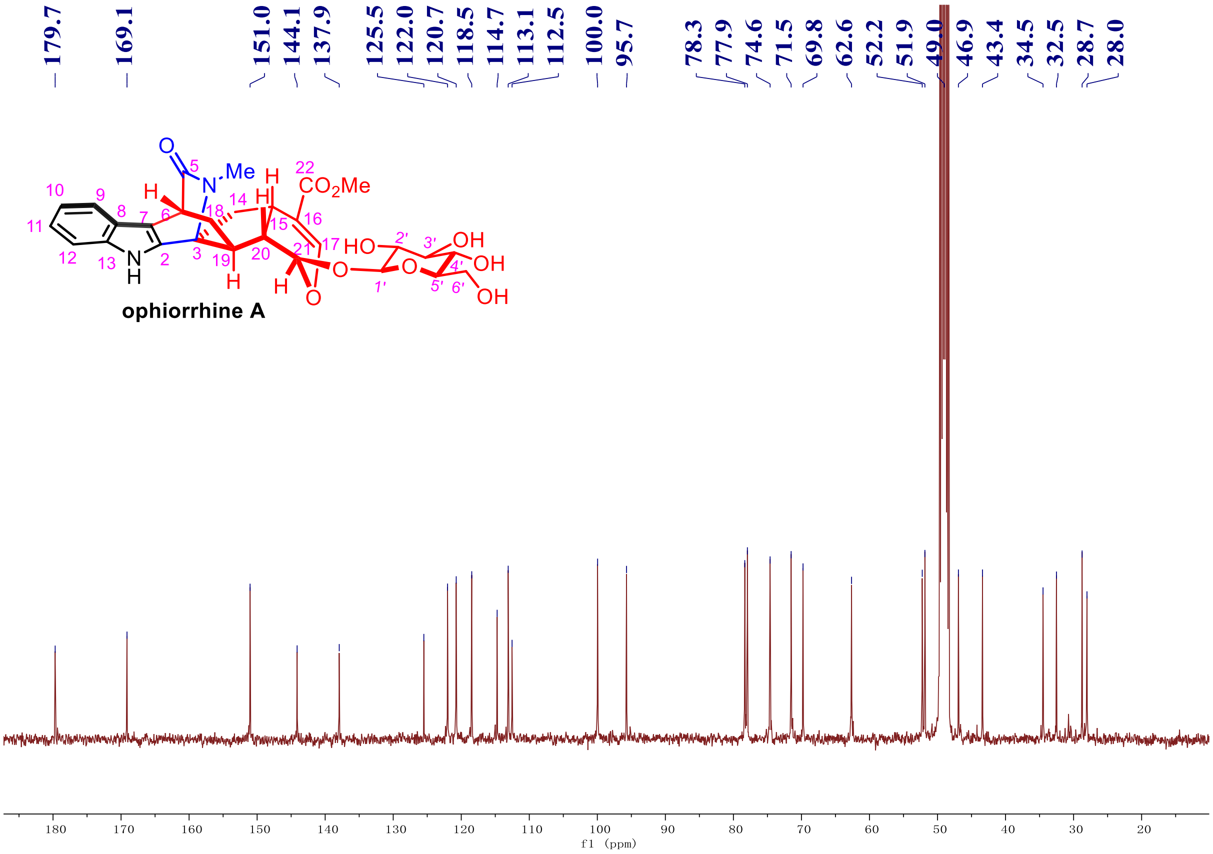

DEPT (400 MHz, CD_3OD), (−)-**1a**

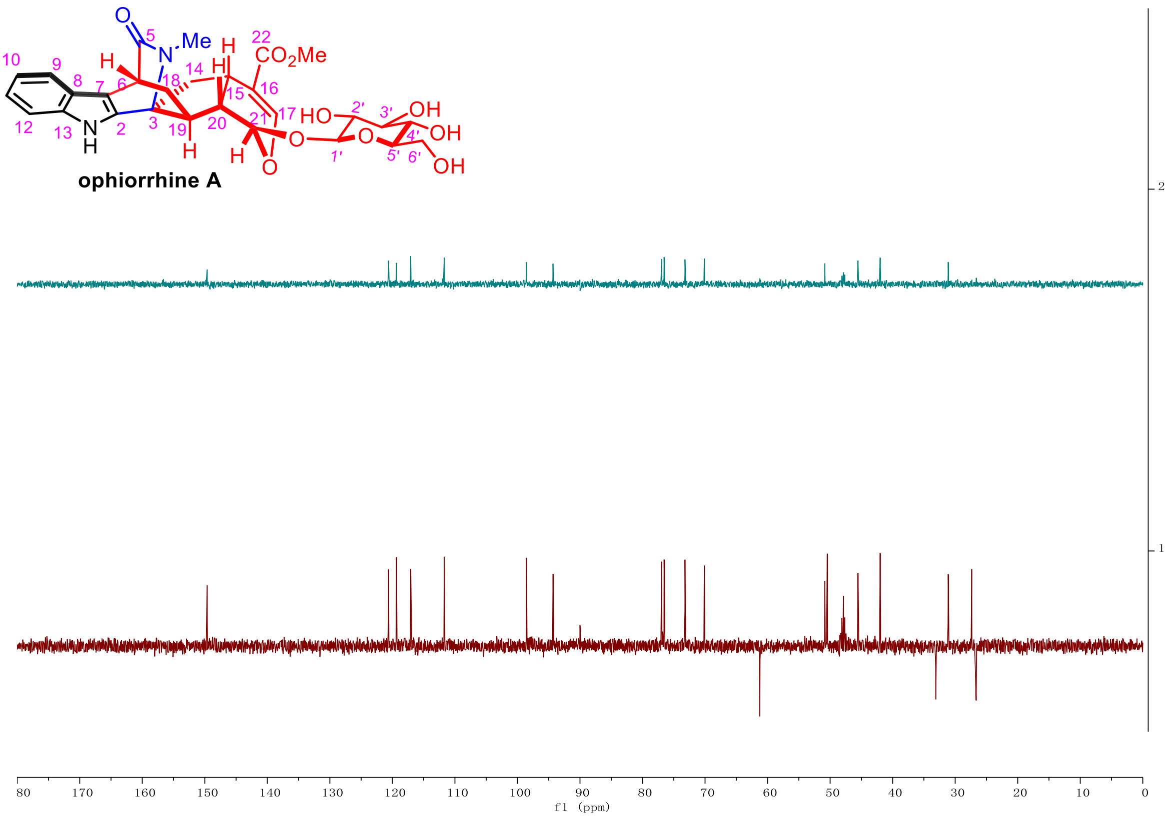

COSY (400 MHz, CD_3OD), (−)-**1a**

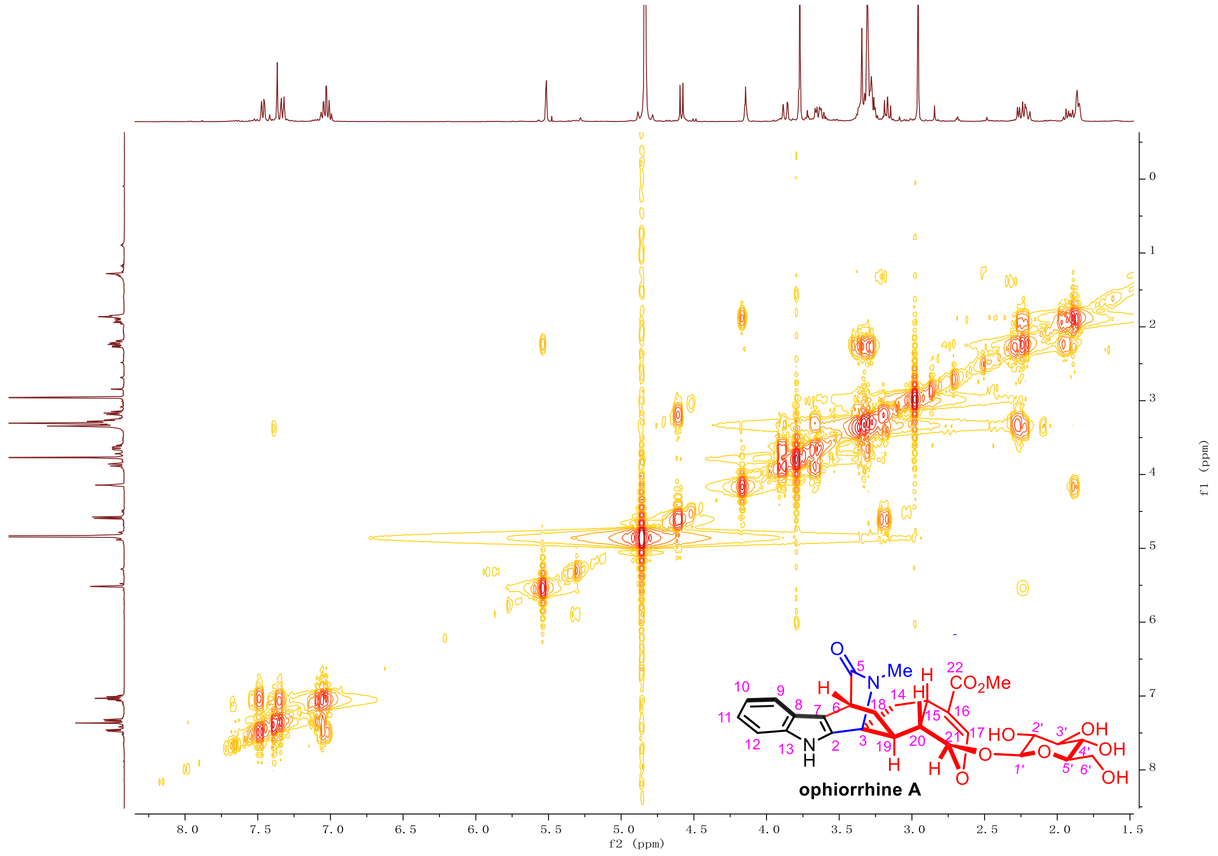

HSQC (400 MHz, CD_3OD), (−)-**1a**

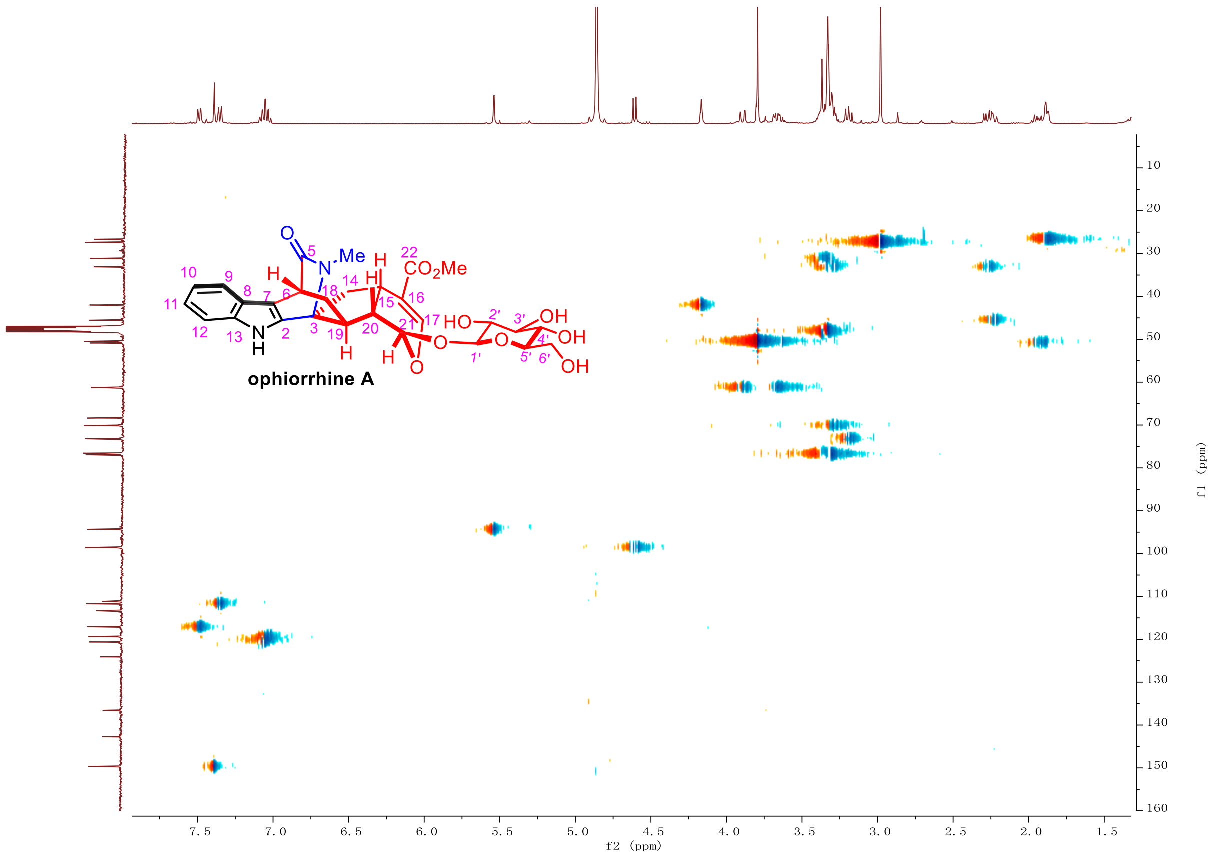

HMBC (400 MHz, CD_3OD), (−)-**1a**

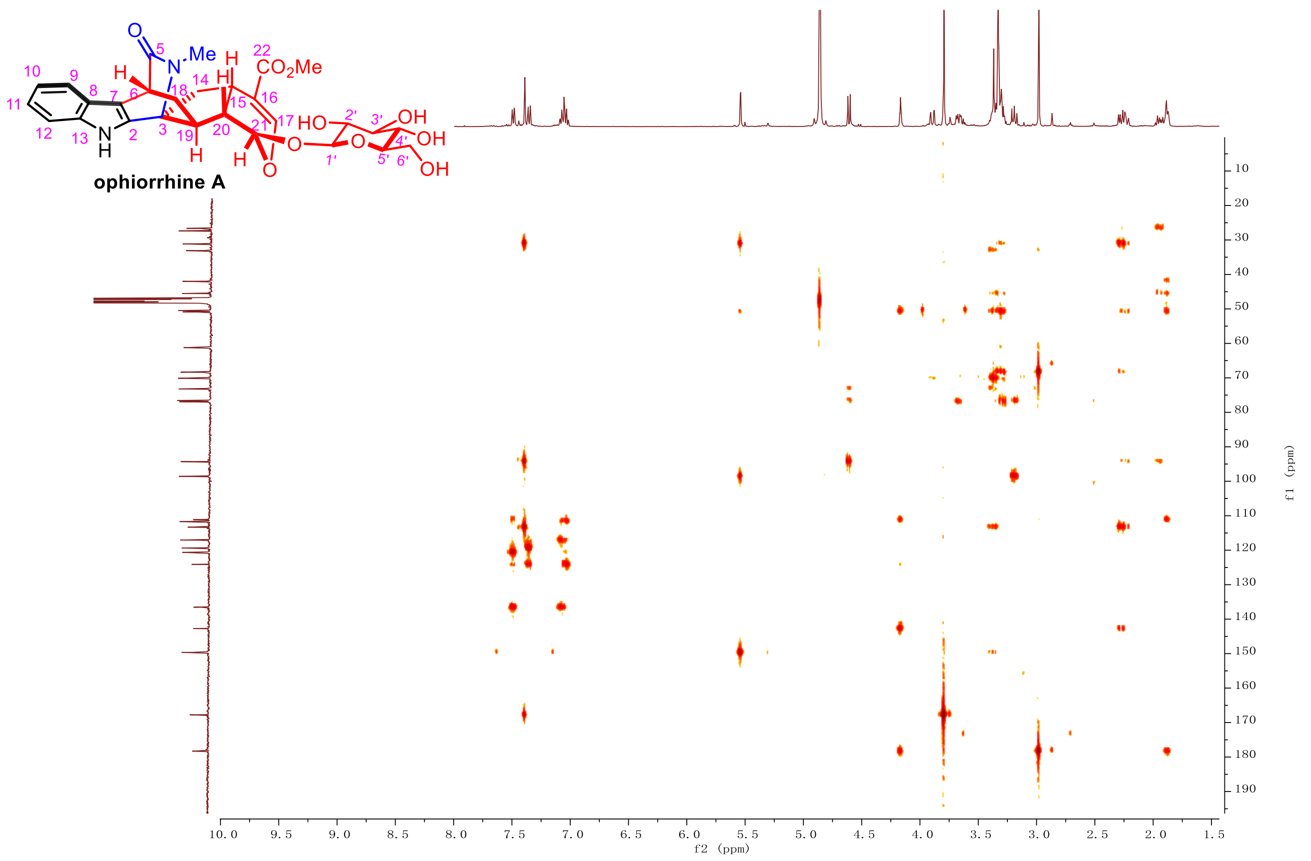

NOESY (400 MHz, CD_3OD), (−)-**1a**

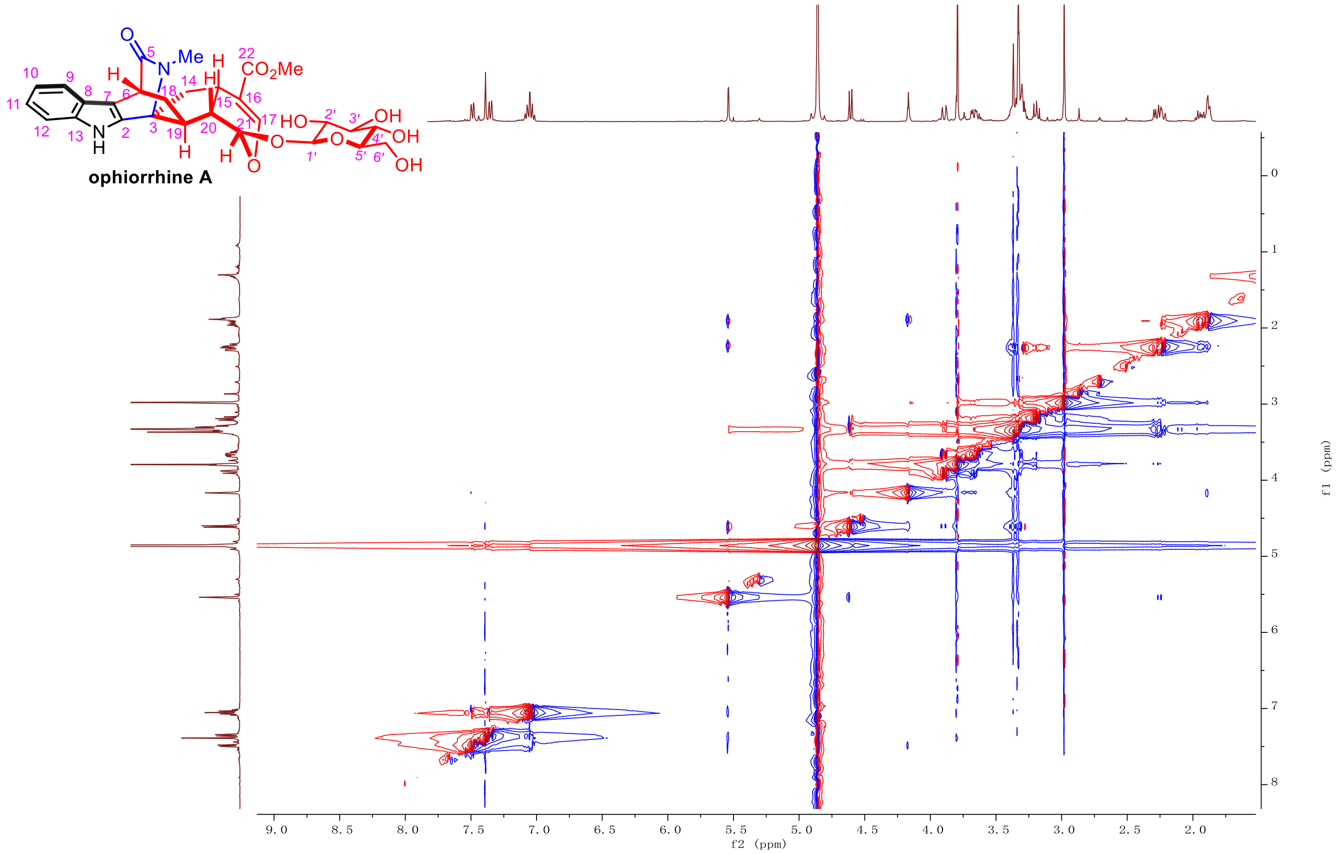

6. References

1. A. Kamal, M. Sathish, A.V.G. Prasanthi, J. Chetna, Y. Tangella, V. Srinivasulu, N. Shankaraiah, A. Alarifi, *RSC Adv*., **2015**, 5, 90121–90126.

2. X. Chen, W.J. Li, S. Li, J. Tang, X. Du, X.L. Zheng, M.L. Yuan, H.Y. Fu, R.X. Li, H. Chen, *J. Org. Chem.*, **2020**, 85, 622–632.

3. Y. Jin, L. Ou, H. Yang, H. Fu, *J. Am. Chem. Soc.*, **2017**, 139, 14237−14243.

4. (a) Y. C. Dou, C. Kouklovsky, V. Gandon, G. Vincent, *Angew. Chem. Int. Ed.,* **2020**, 59, 1527–1531; (b) L. F. Tietze, H. Meier, H. Nutt. *Liebigs Ann. Chem.* **1990**, 253–260.

5. (a) G. Lin, Y. Wang, Q. Zhou, W. Tang, J. Wang, T. Lu, *Molecules*, **2010**, 15, 5680–5691. (b) T. Sasaki, W. Li, T. Ohmoto, K. Koike, *Bioorg. Med. Chem. Lett.*, **2016**, 26, 4992–4995.

6. H.M. Haley, S.E. Payer, S.M. Papidocha, S. Clemens, J. Nyenhuis, R. Sarpong, *J. Am. Chem. Soc.*, **2021**, 143, 4732−4740.

7. S. T. Hilton, T. C. T. Ho, G. Pljevaljcic, K. Jones, *Org. Lett.*, **2000**, 2, 2639–2641.

8. N. Kumar, A. Maity, V.-R. Gavit, A. Bisai, *Chem. Commun*., **2018**, 54, 9083–9086.

9. D.R. IJzendoorn, P.N. Botman, R.H. Blaauw, *Org. Lett.*, **2006**, 8, 239–242.

10. T.T. Wang, D. Zhang, W.W. Liao, *Chem. Commun.*, **2018**, 54, 2048–2051.

11. X.D. An, S. Yu, *Org. Lett.*, **2015**, 17, 2692–2695.

12. X.D. An, S. Yu, *Org. Lett*., **2015**, 17, 5064–5067.

13. (a) M. Ihara, N. Taniguchi, K. Noguchi, K. Fukumoto, T. Kametani, *J. Chem. Soc.*, *Perkin Trans.*, **1988**, 1, 1277–1281; (b) A.V. Muratov, A.B. Eresko, V.S. Tolkunov, Synthesis and Functionalization. *Russ. J. Org. Chem.*, **2019**, 55, 345–350.

14. (a) S. V. Tolkunov, V. S. Tolkunov, V. I. Dulenko, *Chem. Heterocycl. Comp.*, **2004**, 40, 481–489. (b) V. S. Tolkunov, Yu. B. Vysotsky, O. A. Gorban', S. V. Shishkina, O. V. Shishkin, V. I. Dulenko, *Chem. Heterocycl. Comp.*, **2005**, 41, 515–525.

15. G. N. Dorofeenko, V. G. Korobkova, E. A. Gizhina, *Chem. Heterocycl. Compd.*, **1971**, 7, 319–323.

16. H., Erfanian-Abdoust, U. Pindur, *Heterocycles*, **1989**, 29, 1709–1719.

17. R. T. Brown, C. L. Chapple, D. M. Duckworth, R. Platt, *J. Chem. Soc., Perkin Trans.*, **1976**, 1,160–162.

18. (a) Y. C. Dou, C. Kouklovsky, V. Gandon, G. Vincent, *Chem. Eur. J.*, **2020**, 26, 17190–17194; (b) (b) K. Rakumitsu, J. Sakamoto, H. Ishikawa, *Chem. – Eur. J.* **2019**, *25*, 8996–9000.

19. B.B. Shi, H.L. Ai, K.T. Duan, T. Feng, J.K. Liu, *J. Nat. Prod.*, **2022**, 85, 453−457.

20. M. Kitajima, S. Ohara, N. Kogure, D. Santiarworn, H. Takayama, *Tetrahedron*, **2013**, 69, 9451–9456.

21. T. Feng, K.-T. Duan, S.-J. He, B. Wu, Y.-S. Zheng, H.-L. Ai, Z.-H. Li, J. He, J.-P. Zuo, J.-K. Liu, *Org. Lett.*, **2018**, 20, 7926−7928.
